# Supplementary material for: Curated multiple sequence alignment for the Adenomatous Polyposis Coli (APC) gene and accuracy of in silico pathogenicity predictions
Source: PLoS One. 2020 Aug 4;15(8):e0233673. doi: 10.1371/journal.pone.0233673 (PMC7402488; doi:10.1371/journal.pone.0233673)
Supplement: S1 Fig — PMSA was generated from the program Clustal Omega. Exon boundaries are labeled in red with a black background. The domains are highlighted throughout the alignment. Grey is oligomerization domain, red is Armadillo repeats, yellow is Beta Catenin Repeats, green is a sequence with homology to the herpes virus (PHA03307), turquoise is the Basic domain, and purple is the EB1 and HDLG binding site. (PDF) [file pone.0233673.s001.pdf]

<u>**APC ClustO Alignment**</u>

Oligomerization Domain
Exon boundary
Armadillo repeats
Beta Catenin Repeats
PHA03307
Basic Domain (Microtubule Binding)
EB1 and HDLG Binding Site

```
Sea Squirt          ESNRNGETNKELSRYDQLLQQVECLKLENTTLKKELSNNSSQISKLESEALNLKEMVVML      60
Sea Urchin          -----------MSSYDQLLHQVESLKAENSHLKMELKDNSSQLNKLDSVVGHLQSGKP--      47
Zebrafish           ---------MAAASYDQLLKQVEALKMENSNLRQELEDNSNHLNKLETEASNMKEVLKQL      51
Frog                ---------MAAASYDQLVKQVEALTMENTNLRQELEDNSNHLTKLETEATNMKEVLKQL      51
Chicken             ---------MAAASYDQLLKQVEALKMENSNLRQELEDNSNHLTKLETEASNMKEVLKQL      51
Opossum             ---------MAAASYDQLLKQVEALKMENSNLRQELEDNSNHLTKLETEASNMKEVLKQL      51
Mouse               ---------MAAASYDQLLKQVEALKMENSNLRQELEDNSNHLTKLETEASNMKEVLKQL      51
Cow                 ---------MAAASYDQLLKQVEALKMENSNLRQELEDNSNHLTKLETEASNMKEVLKQL      51
Monkey              ---------MAAASYDQLLKQVEALKMENSNLRQELEDNSNHLTKLETEASNMKEVLKQL      51
Human               ---------MAAASYDQLLKQVEALKMENSNLRQELEDNSNHLTKLETEASNMKEVLKQL      51
                                : ****::***.*. **: *: **.:**.::.**:: . :::.

Sea Squirt          GNSIKPPTEASEPNDPRATGFVNPPNRPTALFPPHVYSPDMYY-RYPMPNHHML-------      113
Sea Urchin          -GMGEDDLRA-QITIHGLAMTTAAASATSSSAATSG---AGPASSFQVNGG-AM-------      95
Zebrafish           QGSIDEDSKDSQGQIEFLERIKE-MSLDPSGFSGVKLRSKASLQGSA--------------      97
Frog                QGSIEDEAMASSGPIDLLERFKD-LNLDSSNIPAGKARPKMSMRSYGSREG-SL-------      103
Chicken             QGSIEDEAIASSGQIDLLERLKE-LNLESTSFPGVKLRQKVSVRSYGSREG-SV-------      103
Opossum             QGSIEDEAMASSGQIDLLERLKE-LNLDSSNFPGVKLRPKMSIRSYGSREG-SV-------      103
Mouse               QGSIEDETMT-SGQIDLLERLKE-FNLD-SNFPGVKLRSKMSLRSYGSREG-SV-------      101
Cow                 QGSIEDEAMASSGQIDLLERLKE-LNLDSSNFPGVKLRSKMSLRSYGSREG-SV-------      103
Monkey              QGSIEDEAMASSGQIDLLERLKE-LNLDSSNFPGVKLRSKMSLRSYGSREG-SV-------      103
Human               QGSIEDEAMASSGQIDLLERLKE-LNLDSSNFPGVKLRSKMSLRSYGSREG-SV-------      103
                     .                  :    .   :

Sea Squirt          DVGHTPLSPSPSST-----IA---VEIRRHLSNLHLQRTALLESLKREELAKDGYMRQLN      165
Sea Urchin          LPGKPHSLPTSLGTSGTDGSLRQTKDHFIRHQELERDRLLILNEQAKEENLRSQCLAQIQ      155
Zebrafish           ---DSSPSPSPVSSCPRRGASSGGRDSAGYLEELEKERSLLVAELEKEEKEKDWYYAQLQ      154
Frog                SGHSGECSPVPVGSFQRRGLLNGSRESAGYMEELEKERLLLIAEHEKEEKEKRWYYAQLQ      163
Chicken             SSRSGECSPVPMGSFPRRGFMNGSRESTGYLEELEKERSLLLAELEKEEKEKDWYYAQLQ      163
Opossum             SSRSGECSPVPMGSFSRRGFMNGSRESTGYLEELEKERSLLLAELEKEEKEKDWYYAQLQ      163
Mouse               SSRSGECSPVPMGSFPRRTFVNGSRESTGYLEELEKERSLLLADLDKEEKEKDWYYAQLQ      161
Cow                 SSRSGECSPVPMGSFPRRGFVNGSRENTGYLEELEKERSLLLADLDKEEKEKDWYYAQLQ      163
Monkey              SSRSGECSPVPMGSFPRRGFVNGSRESTGYLEELEKERSLLLADLDKEEKEKDWYYAQLQ      163
Human               SSRSGECSPVPMGSFPRRGFVNGSRESTGYLEELEKERSLLLADLDKEEKEKDWYYAQLQ      163
                            *   .:           :     .:*. :*  :: .  :**  :     *::

Sea Squirt          DLSEQLKIMHDTDKPFSPTSEKTRLQLEAGIREIQDQMEKNLGSPEMMSHRVQ-------      218
Sea Urchin          SLTRRIEDLPIT-DNYSLQTDMSRRQLEYEARQLRELMQERLGSMEDIAARHRMRMQRLS      214
Zebrafish           NLTKRIDSLPLT-ENFSLQTDMTRRQLEYEARQIRAAMEDQLGTCQDMEKRAQGRVARIQ      213
Frog                NLTKRIDSLPLT-ENFSMQTDMTRRQLEYEARQIRAAMEEQLGTCQDMEKRVQTRVGKIH      222
Chicken             NLTKRIDSLPLT-ENFSLQTDMTRRQLEYEARQIRAAMEEQLGTCQDMEKRAQVRVARIQ      222
Opossum             NLTKRIDSLPLT-ENFSLQTDMTRRQLEYEARQIRAAMEEQLGTCQDMEKRAQLRVARIQ      222
Mouse               NLTKRIDSLPLT-ENFSLQTDMTRRQLEYEARQIRAAMEEQLGTCQDMEKRAQRRIARIQ      220
Cow                 NLTKRIDSLPLT-ENFSLQTDMTRRQLEYEARQIRVAMEEQLGTCQDMEKRAQRRITRIQ      222
Monkey              NLTKRIDSLPLT-ENFSLQTDMTRRQLEYEARQIRVAMEEQLGTCQDMEKRAQRRIARIQ      222
Human               NLTKRIDSLPLT-ENFSLQTDMTRRQLEYEARQIRVAMEEQLGTCQDMEKRAQRRIARIQ      222
                    .*:.::. :  * . :*  :: :* ***   *:::  *:..**: : :  * :

Sea Squirt          ------------------------------------------------------------      218
Sea Urchin          AIEYELRQVQEQQQQHQQQQQQQQQQQQQQQQQQMLAKVWEQGAQ---------------      259
Zebrafish           QIEKDMLRIRTRLQAQSAESESSGKR-YRERVKHEPLSQTEGSHAAGDAGAAAAASVCSQ      272
Frog                QIEEEILRIRQLLQSQVAEAAERTPQSKHDAGSRDAEKLPDGQGTSE--ITASGNVGSGQ      280
Chicken             QIEKDILRIRQLLQSQAAE-AERAPQGKHDAASHDTERQSEGQGAPE--ISMS-TSNTGQ      278
Opossum             QIEKDILRIRQLLQSQPAE-AERASQSKPDHASHEAERPNEGPGAAD--VSVA-PPAGSQ      278
Mouse               QIEKDILRVRQLLQSQAAE-AERSSQSRHDAASHEAGRQHEGHGVAE--SNTA-ASSSGQ      276
Cow                 QIEKDILRIRQLLQSQATE-AERSSQSKHEAGSHEAERQNEGQGVAE--INMA-TSGSGQ      278
Monkey              QIEKDILRIRQLLQSQATE-AERSSQNKHETGSHDAERQNEGQGVAE--INMA-TSGNGQ      278
Human               QIEKDILRIRQLLQSQATE-AERSSQNKHETGSHDAERQNEGQGVGE--INMA-TSGNGQ      278

Sea Squirt          ---------------------------------TG---EVCSLLGSHDRHDMSCTFQRLS      242
```

```
Sea Urchin          ---------------------------------VGFVYSLLSMLGSHDRDDMASTLLMMS   275
Zebrafish           GSASRVDHDSASEMSSAGSYSVPRRLTSHLGTKVEMVYSLLSMLGTHDKDDMSRTLLAMS   332
Frog                GSSSRADHDTTSVMSSNSTYSVPRRLTSHLGTKVEMVYSLLSMLGTHDKDDMSRTLLAMS   340
Chicken             GSAARMDHETASVMSSSNNYSVPRRLTSHLGTKVEMVYSLLSMLGTHDKDDMSRTLLAMS   338
Opossum             GSVAQVDQETASGGSANGAYSVPRRLTSHLGTKVEMVYSLLSMLGTHDKDDMSRTLLAMS   338
Mouse               SPATRVDHETASVLSSSGTHSAPRRLTSHLGTKVEMVYSLLSMLGTHDKDDMSRTLLAMS   336
Cow                 GSTTRIDHETASVLSSSSTHSAPRRLTSHLGTKVEMVYSLLSMLGTHDKDDMSRTLLAMS   338
Monkey              GSTTRMDHETASVLSSSSTHSAPRRLTSHLGTKVEMVYSLLSMLGTHDKDDMSRTLLAMS   338
Human               GSTTRMDHETASVLSSSSTHSAPRRLTSHLGTKVEMVYSLLSMLGTHDKDDMSRTLLAMS   338
                                                    . .: *:**:**:.**: *:  :*

Sea Squirt          QSEDSCIVMRQDGYLPSMLRLLHDGS---ILPGGANPTPEEVKLRLEIRSKTSQALTNIV   299
Sea Urchin          RSADSCLAMRQSGCIPLLIHILHGTDQESV-LGNF-------RGSQKARDCASTALHNIV   338
Zebrafish           SSQDSCIAMRQSGCLPLLIQLLHGNDKDSVLLGNS-------RGSKEARARASAALHNII   385
Frog                SSQDSCIAMRQSGCLPLLIQLLHGNDKDSVLLGNS-------RGSKEARASGSAALDNII   393
Chicken             SSQDSCIAMRQSGCLPLLIQLLHGNDKDSVLLGNS-------RGSKEARARASAALHNII   391
Opossum             SSQDSCISMRQSGCLPLLIQLLHGNDKDSVLLGNS-------RGSKEARARASAALHNII   391
Mouse               SSQDSCISMRQSGCLPLLIQLLHGNDKDSVLLGNS-------RGSKEARARASAALHNII   389
Cow                 SSQDSCISMRQSGCLPLLIQLLHGNDKDSVLLGNS-------RGSKEARARASAALHNII   391
Monkey              SSQDSCISMRQSGCLPLLIQLLHGNDKDSVLLGNS-------RGSKEARARASAALHNII   391
Human               SSQDSCISMRQSGCLPLLIQLLHGNDKDSVLLGNS-------RGSKEARARASAALHNII   391
                     * ***: ***.* :* ::::**. .   :  *.        :   : *   * ** **:

Sea Squirt          SSNTDGDQKRIESHVLRHLEMIRRVSEDLFESLVEKNHTGASSESAESPTRHPVAETLKN   359
Sea Urchin          HLNPDEKRRKQEGRVLRLLEQIRTYCDSLVEAETKES---------ASSQNALPIDH----   386
Zebrafish           HSQPDDKRGRREIRVLHLLEQIRAYCETCWEWQESHE-RGV--DQDKNPMPSPVEH----   438
Frog                HSQPDDKRGRREIRVLHLLEQIRAYCETCWEWQEAHE-QGM--DQDKNPMPAPVDH----   446
Chicken             HSQPDDKRGRREIRVLHLLEQIRAYCETCWEWQEAHE-QGM--DQDKNPMPAPVDH----   444
Opossum             HSQPDDKRGRREIRVLHLLEQIRAYCETCWQWQEAHE-QGM--DQDKNPMPAPVEH----   444
Mouse               HSQPDDKRGRREIRVLHLLEQIRAYCETCWEWQEAHE-QGM--DQDKNPMPAPVEH----   442
Cow                 HSQPDDKRGRREIRVLHLLEQIRAYCETCWEWQEAHE-QGM--DQDKNPMPAPVEH----   444
Monkey              HSQPDDKRGRREIRVLHLLEQIRAYCETCWEWQEAHE-QGM--DQDKNPMPAPVEH----   444
Human               HSQPDDKRGRREIRVLHLLEQIRAYCETCWEWQEAHE-PGM--DQDKNPMPAPVEH----   444
                      : *  .: :  * :**:  **  **  .:   :    ..         ..    *: .

Sea Squirt          AESIIHSLREIMELVNYKVQRLAINELGGLFCVAEILILHCSSKHDEEAQEETGSRLRQY   419
Sea Urchin          --NPGPAMAALMKLSFDEEHRSAICHLGGLHAIAELLQVDYEVHGS--SNDQYTVTLRRY   442
Zebrafish           --QICPAVCVLMKLSFDEEHRHAMNELGGLQAIGELLQVDCEIYGL--TNDHYSVTLRRY   494
Frog                --QICPAVCVLMKLSFDEEHRHAMNELGGLQAIAELLQVDCEMYGL--INDHYSVTLRRY   502
Chicken             --QICPAVCVLMKLSFDEEHRHAMNELGGLQAIAELLQVDCEMYGL--TNDHYSVTLRRY   500
Opossum             --QICPAVCVLMKLSFDEEHRHAMNELGGLQAIAELLQVDCEMYGL--TSDHYSVTLRRY   500
Mouse               --QICPAVCVLMKLSFDEEHRHAMNELGGLQAIAELLQVDCEMYGL--TNDHYSVTLRRY   498
Cow                 --QICPAVCVLMKLSFDEEHRHAMNELGGLQAIAELLQVDCEMYGL--TNDHYSITLRRY   500
Monkey              --QICPAVCVLMKLSFDEEHRHAMNELGGLQAIAELLQVDCEMYGL--TNDHYSITLRRY   500
Human               --QICPAVCVLMKLSFDEEHRHAMNELGGLQAIAELLQVDCEMYGL--TNDHYSITLRRY   500
                      .   ::  :*:*   : :* *:  .**** .:.*:* :. .       .:.    **:*

Sea Squirt          SGRILTNLTYADNLNKVLLMNMRGLLETVRDQLQHESEEIQQAMASILRNLSWQADKEGR   479
Sea Urchin          AGMALTNLTFGDVTNKALLCSMKGCMKALVALLSAESEDLRQVAASVLRNLSWRADMASK   502
Zebrafish           AGMALTNLTFGDVANKATLCSMKGCMRAMVAQLKSESEDLQQVIASVLRNLSWRADVNSK   554
Frog                AGMALTNLTFGDVANKATLCSMKSCMRALVAQLKSESEDLQQVIASVLRNLSWRADVNSK   562
Chicken             AGMALTNLTFGDVANKATLCSMKGCMRALVAQLKSESEDLQQVIASVLRNLSWRADVNSK   560
Opossum             AGMALTNLTFGDVANKATLCSMKGCMRALVAQLKSESEDLEQVIASVLRNLSWRADVNSK   560
Mouse               AGMALTNLTFGDVANKATLCSMKGCMRALVAQLKSESEDLQQVIASVLRNLSWRADVNSK   558
Cow                 AGMALTNLTFGDVANKATLCSMKGCMRALVAQLQSESEDLQQVIASVLRNLSWRADVNSK   560
Monkey              AGMALTNLTFGDVANKATLCSMKGCMRALVAQLKSESEDLQQVIASVLRNLSWRADVNSK   560
Human               AGMALTNLTFGDVANKATLCSMKGCMRALVAQLKSESEDLQQVIASVLRNLSWRADVNSK   560
                    :*  *****:.*  **. * .*:. :.::   *. ***::.*. **:******:**  .:

Sea Squirt          DLLRQKGVVRTLTECVICAKGEGTLKAMLSALWNLSGHCPANREDICSVEGSLAFLVSTL   539
Sea Urchin          KALREAGAVVALMTCSLEVKKESTLKSVLSALWNLSAHCTENKADICAVNGALEFLVSSL   562
Zebrafish           KILREVGSVRALMECALEVQKESTLKSVLSALWNLSAHCTENKADICTVPGALAFLVSTL   614
Frog                KTLREVGSVKALMECALDVKKESTLKSVLSALWNLSAHCTENKADICSVDGALAFLVSTL   622
Chicken             KTLREVGSVKALMECALEVKKESTLKSVLSALWNLSAHCTENKADICAVDGALAFLVGTL   620
Opossum             KTLREVGSVKALMECALEVKKESTLKSVLSALWNLSAHCTENKADICAVDGALAFLVGTL   620
Mouse               KTLREVGSVKALMECALEVKKESTLKSVLSALWNLSAHCTENKADICAVDGALAFLVGTL   618
Cow                 KTLREVGSVKALMECALEVKKESTLKSVLSALWNLSAHCTENKADICAVDGALAFLVGTL   620
Monkey              KTLREVGSVKALMECALEVKKESTLKSVLSALWNLSAHCTENKADICAVDGALAFLVGTL   620
Human               KTLREVGSVKALMECALEVKKESTLKSVLSALWNLSAHCTENKADICAVDGALAFLVGTL   620
                    . **: * * :*  * : .: *.***::********.**  *: ***:* *:* ***.:*
```

```
Sea Squirt      TYRSPSKSAMVIESGGGILRNISSIVATNETFRQILREHNCLQTLLQQLTSASLTIVSNA   599
Sea Urchin      TYRSPTRNSAVVENGGGILRNVSSHIATSEKYRQLLRKHNCLQILLHHLKSSSLTIVSNA   622
Zebrafish       TYRSQTNTLAIIESGGGILRNVSSLIATNEEHRQILRENSCLQTLLQHLKSHSLTIVSNA   674
Frog            TYRSQTNTLAIIESGGGILRNVSSLIATNEDHRQILRENNCLQTLLQHLKSHSLTIVSNA   682
Chicken         TYRSQTNTLAIIESGGGILRNVSSLIATNEDHRQILRENSCLQTLLQHLKSHSLTIVSNA   680
Opossum         TYRSQTNTLAIIESGGGILRNVSSLIATNEDHRQILRENSCLQTLLQHLKSHSLTIVSNA   680
Mouse           TYRSQTNTLAIIESGGGILRNVSSLIATNEDHRQILRENNCLQTLLQHLKSHSLTIVSNA   678
Cow             TYRSQTNTLAIIESGGGILRNVSSLIATNEDHRQILRENNCLQTLLQHLKSHSLTIVSNA   680
Monkey          TYRSQTNTLAIIESGGGILRNVSSLIATNEDHRQILRENNCLQTLLQHLKSHSLTIVSNA   680
Human           TYRSQTNTLAIIESGGGILRNVSSLIATNEDHRQILRENNCLQTLLQHLKSHSLTIVSNA   680
                **** :..  ::*.*******:** :**.* .**:**::.*** **::*.* ********

Sea Squirt      CGTLWNLSARDETDQQTLRELGAVNKLQKLIHSKHTVIAQGSAAALRNLLANRGDRLNTV   659
Sea Urchin      CGTLWNLSARNKADQDLLWELGAVSMLKNLISSKHKMIAMGSSAALRNLMASRPDVLATA   682
Zebrafish       CGTLWNLSARNAKDQEALWDMGAVSMLKNLIHSKHKMIAMGSAAALRNLMANRPAKYKDA   734
Frog            CGTLWNLSARNAKDQEGLWDMGAVSMLKNLIHSKHKMIAMGSAAALRNLMANRPAKYKDA   742
Chicken         CGTLWNLSARNAKDQEALWDMGAVSMLKNLIHSKHKMIAMGSAAALRNLMANRPAKYKDT   740
Opossum         CGTLWNLSARNTKDQEALWDMGAVSMLKNLIHSKHKMIAMGSAAALRNLMANRPAKYKDA   740
Mouse           CGTLWNLSARNPKDQEALWDMGAVSMLKNLIHSKHKMIAMGSAAALRNLMANRPAKYKDA   738
Cow             CGTLWNLSARNPKDQEALWDMGAVSMLKNLIHSKHKMIAMGSAAALRNLMANRPAKYKDA   740
Monkey          CGTLWNLSARNPKDQEALWDMGAVSMLKNLIHSKHKMIAMGSAAALRNLMANRPAKYKDA   740
Human           CGTLWNLSARNPKDQEALWDMGAVSMLKNLIHSKHKMIAMGSAAALRNLMANRPAKYKDA   740
                **********:  **: * ::***. *::** ***.:** **:******:*.*      .

Sea Squirt      FQNETKGDMPTLQTRKLRQLEADIQLASK-NAPEISENFSPRHHAHITT-----------   707
Sea Urchin      DGQK--EGTPGLHVRKQRALQAEIDKN-LKDTYAEMEGRTDQHGLLQSQQRASLRNRGHR   739
Zebrafish       NIMSPGSSLPSLHVRKQKALIEELDAQHLSETFDNIDNLSPKA--------------SHR   780
Frog            NIMSPGSSVPSLHVRKQKALEAELDAQHLSETFDNIDNLSPKT--------------THR   788
Chicken         NIMSPGSSLPSLHVRKQKALEAELDAQHLSETFDNIDNLSPKA--------------SHR   786
Opossum         NIMSPGSSLPSLHVRKQKALEAELDAQHLSETFDNIDNLSPKT--------------SHR   786
Mouse           NIMSPGSSLPSLHVRKQKALEAELDAQHLSETFDNIDNLSPKA--------------SHR   784
Cow             NIMSPGSSLPSLHVRKQKALEAELDAQHLSETFDNIDNLSPKA--------------SHR   786
Monkey          NIMSPGSSLPSLHVRKQKALEAELDAQHLSETFDNIDNLSPKA--------------SHR   786
Human           NIMSPGSSLPSLHVRKQKALEAELDAQHLSETFDNIDNLSPKA--------------SHR   786
                    .   . * *:.** : *  :::     ::    :. : :

Sea Squirt      -----NQN--------------SMARLSLQEPGFV--QKSGVNSHLSPGIHYSGSLGNLP   746
Sea Urchin      SRQQQHGP---DYSPV-------PQRIPIWNPNATPLPDSLMNSQQRAHSPSNRHHGNMI   789
Zebrafish       VKPRHKHNVYGDY--------DAVCRSDGYNPNGVGVRSPYMNTPVLSSPSSRDNRGNAE   832
Frog            NKQRHKQNLCSEYALDSSRHDDSICRSDNFSIGNLTVLSPYINTTVLPGSS-S-PRPTMD   846
Chicken         NKQRHKQNIYGEYVLDSSRHDDGVCRTESFNTGNMTVLSPYLNSTVLPGSA-SSSRGNIE   845
Opossum         PKQRHKQSVYGEYALDASRHDDS--RPDAFSTGNLTVLSPYLNSTVLPGSS--SSRTSLE   842
Mouse           SKQRHKQNLYGDYAFDANRHDDS--RSDNFNTGNMTVLSPYLNTTVLPSSS--SSRGSLD   840
Cow             SKQRHKQNLYGDYVFDTNRHDDN--RSDNFNTGNMTVLSPYLNTTVLPSSS--SSRGSLD   842
Monkey          SKQRHKQSLYGDYVFDTNRHEDN--RSDNFNAGNMTVLSPYLNTTVLPSSS--SSRGSLD   842
Human           SKQRHKQSLYGDYVFDTNRHDDN--RSDNFNTGNMTVLSPYLNTTVLPSSS--SSRGSLD   842
                     :                   *    . .     .  :*:             .

Sea Squirt      QQTY--------SPSPGLPFRA----WEDSGS---KNMLRAPTSNSGVFQN--HPLSSHL   789
Sea Urchin      NSPETCSDDQSFQEGKTKD-SEGQSTSSQPGSVE--NSPGRPHGASRIAQIMQEVAQDGL   846
Zebrafish       SVRA--ERDRSLDRERRGFLPD---------GEAAKRMMQIPTSAAQIAVVMEEVQNMHL   881
Frog            GSRP--EK----DRERTAGLGNYHSTTESSGNSSKRIGIQLSTT-AQISKVMDEVSNIHL   899
Chicken         NCLS--EKDRSLDRDRAVGLNAYHPATENSGNSSKRIGMQISTAAAQIAKVMEEVTSMHI   903
Opossum         SSRS--EKDRSLDRERAVALSTFHPAADSPGNPSKRLGMQLSTTTAQIAKVMEEVSAIHA   900
Mouse           SSRS--EKDRSLERERGIGLSAYHPTTENAGTSSK-RGLQITTTAAQIAKVMEEVSAIHT   897
Cow             SSRS--EKDRSLERERGISLGNYHPATENPGTSSK-RGLQISTTAAQIAKVMEEVSAIHT   899
Monkey          SSRS--EKDRSLERERGIGLGNYHPATENPGTSSK-RGLQISTTAAQIAKVMEEVSAIHT   899
Human           SSRS--EKDRSLERERGIGLGNYHPATENPGTSSK-RGLQISTTAAQIAKVMEEVSAIHT   899
                            .                                : :     .

Sea Squirt      QFNNNIS----------------RAT---SFADQNQNPFSNSLPSSRTKSGNLYGKL-PQ   829
Sea Urchin      PTDSS-SGSESPRRESLESRLQSRNGHQQKPTCPRSSSFTHM----PPEGSNLSSRSNSY   901
Zebrafish       GMDDRSAGSTPDPHSVQDD--MIRRQ---TAVHGHQNIYSYS----KTDPSG------RP   926
Frog            VQENRSSGSASEMHCMSDERNSQRKP---SSNHPQSNPFTFT----KAESST------RG   946
Chicken         PQEDRSSGSTSEMHCLTEDRNTTRRA---ATAHTHSNTY-FP----KSENSS------RP   949
Opossum         Q-EDQSSASTTDLHCVAEERSTLRRA---SAAHAHSNTYNFP----KPDNSN------RT   946
Mouse           SQDDRSSASTTEFHCVADDRSAARRS---SASHTHSNTYNFT----KSENSN------RT   944
Cow             SQEDRSSGSTTELHCGTDERNALRRS---STTHTHANTYNFT----KSENSN------RT   946
Monkey          SQEDRSSGSTTELHCVTDERNALRRS---SAAHTHSNTYNFT----KSENSN------RT   946
Human           SQEDRSSGSTTELHCVTDERNALRRS---SAAHTHSNTYNFT----KSENSN------RT   946
                  :.  :                *          . . :         . .
```

```
Sea Squirt     CDFKAAKTWGHYHSVNNNSNNT---ELW---------------NPNQ--SGNGNPEKMLS     869
Sea Urchin     CFGFDGGHGLVARRSSTESINSISSDIFPAGIHERLAQNRSQMDHSQ---SADSSLNMHG    958
Zebrafish      CPM--PKL--EY-RASNDSLNSVNSTD---GYG-----KRGQMKPSVDSYSEDDEGKCCV    973
Frog           CPV--AFMKMEYKMASNDSLNSVSSTE---GYG-----KRGQVKPSVESYSEDDESKFFS    996
Chicken        CPV--PYTKMEYKRASNDSLNSVSSSD---GYG-----KRGQMKPSIESYSEDDESKFCS    999
Opossum        CAM--PYAKVEYKRSSNDSLNSVSSSD---GYG-----KRGQMKPSIESYSEDDESKFCS    996
Mouse          CSM--PYAKVEYKRSSNDSLNSVTSSD---GYG-----KRGQMKPSVESYSEDDESKFCS    994
Cow            CPI--PYAKVEYKRSSNDSLNSVSSSD---GYG-----KRGQMKPSIESYSEDDESKFCS    996
Monkey         CSM--PYAKLEYKRSSNDSLNSVSSSD---GYG-----KRGQMKPSIESYSEDDESKFCS    996
Human          CSM--PYAKLEYKRSSNDSLNSVSSSD---GYG-----KRGQMKPSIESYSEDDESKFCS    996
               *              ..:* *:                    . .    . ..  :

Sea Squirt     PSRMPDDLEEKLRHMN--DDYGMDQDDLPVNYSLQFTDEQSQTPGIQSPRTEEIYEKSAT    927
Sea Urchin     TGRSLQNTTALVHSAD--EAFG-TNMDSTTNYSLKYSEEDL-PPGMHSPKRTAPVEH---    1011
Zebrafish      YRKYPADLAHKIHNANHMEDDN-GDLDTPINYSLKYSDEQL-NSGRQSPSQNERWARPK-    1030
Frog           YGQYPAGLAHKIQSANHMDDND-TELDTPINYSLKYSDEQL-NSGRQSPTQNERWSRPKH    1054
Chicken        YGQYPADLAHKIHSANHMDDND-GELDTPINYSLKYSDEQL-NSGRQSPSQNERWARPKH    1057
Opossum        YGQYPADLAHKIHSANHMDDND-EELDTPINYSLKYSDEQL-NSGRQSPSQNDRWARPKH    1054
Mouse          YGQYPADLAHKIHSANHMDDND-GELDTPINYSLKYSDEQL-NSGRQSPSQNERWARPKH    1052
Cow            YGQYPADLAHKIHSANHMDDND-GELDTPINYSLKYSDEQL-NSGRQSPSQNERWARPKH    1054
Monkey         YGQYPADLAHKIHSANHMDDND-GELDTPINYSLKYSDEQL-NSGRQSPSQNERWARPKH    1054
Human          YGQYPADLAHKIHSANHMDDND-GELDTPINYSLKYSDEQL-NSGRQSPSQNERWARPKH    1054
                 :   .     ::  :  :  .  : *   ****::::*:    * :**         :

Sea Squirt     RMPKKSSAIA---------------TK--------------PE------VP-GKSMGNAVVG  953
Sea Urchin     RCKDGNSQH-----------SSVDGEQSEEG--------LSQPCQKCNSPHRPREGLAIG    1052
Zebrafish      LLDDEMKRPDQKPPRSQSPGYPMYTEGSSEGEDKPKKYQPRFVQQD-LPAFRSR----GS    1085
Frog           IIDSEMKQSEQRQPRTTKTTYSSYTEN---KEEKHKKFPPHFNQSENVPAYTRSRGANNQ    1111
Chicken        IIDDEMKQNDQRQSRSQSATYPVYTES---GDDKHMKYQSPFGQQDCVPSFRSR-GS-NG    1112
Opossum        VIEDEIKQNEQRQARGQNTPFSAYSES---TDDKHMKFQSRFGQQECVSPYRSR-GA-SG    1109
Mouse          VIEDEIKQNEQRQARSQNTSYPVYSEN---TDDKHLKFQPHFGQQECVSPYRSR-GT-SG    1107
Cow            ILEDEIKPNEQRQSRSQSTAYPVYPES---TDDKHLKFQPHFGQQECVSPYRSR-AA-NG    1109
Monkey         IIEDEIKQSEQRQSRSQSTTYPVYTES---TDDKHLKFQPHFGQQECVSPYRSR-GA-NG    1109
Human          IIEDEIKQSEQRQSRNQSTTYPVYTES---TDDKHLKFQPHFGQQECVSPYRSR-GA-NG    1109
                  .  .                  :

Sea Squirt     NKALKIAPR--------------RHKPPAKVDFS------------VEQRILYADEESFD    987
Sea Urchin     NSYPASQQQRPFPQQYSDQSVSSHHNVID----------------PMQGSSMYSNMNDHE    1096
Zebrafish      NEQ---------------ISSGSSHGLNKKISQTICSVDDYADDKPTNYSERYSEEEQLE    1130
Frog           VDQ---------------SRVSSNLSNNSKASKPHCQVDDYDDDKTTNFSERYSEEEQQE    1156
Chicken        SDQ---------------NRVGSTLGINQKVNQSLCQVDDYDDDKPTNYSERYSEEEQHE    1157
Opossum        SEQ---------------NRVSSGHGINQKVNQSLCHEDDYDEDKPTNYSERYSEEEQHE    1154
Mouse          SET---------------NRMGSSHAINQNVNQSLCQEDDYEDDKPTNYSERYSEEEQHE    1152
Cow            SET---------------NRVGSNHGISQNVNQSLCQEDDYEDDKPTNYSERYSEEGQHE    1154
Monkey         SET---------------NRVGSNHGINQNVSQSLCQEDDYEDDKPTNYSERYSEEEQHE    1154
Human          SET---------------NRVGSNHGINQNVSQSLCQEDDYEDDKPTNYSERYSEEEQHE    1154
                .                                              :    *::  . :

Sea Squirt     -------------------AEETPTNFGAIYREEQ-------------------------    1003
Sea Urchin     ----------------PFAEEDERPTDFSQRYANDMSHGDDAESFGFVQTSLETNTGGTVY    1141
Zebrafish      EQ---TPSYSMK--YTEDHHVEQPIDYSLKYSEA---PSKKGMFSHSKTSSAQSSAKEHL    1182
Frog           DETERQNKYNIKAYASEEHHGEQPIDYSRKYSTDVPSSAQKPSFPYSNNSSKQKPKKEQV    1216
Chicken        E-EDRPTNYSIK-YNEEEHQVDQPIDYSLKYSTEVPPSSQKPSFTFSKTSSVQSTKTDHI    1215
Opossum        E-EDRPTNYSMK-YNEEEHHADQPIDYSLKYAADITPSSQKPSFSFSKSSSVQSNKTGHI    1212
Mouse          EEEERPTNYSIK-YNEEKHHVDQPIDYSLKYATDI-SSSQKPSFSFSKNSSAQSTKPEHL    1210
Cow            E-EERPTNYSIK-YSEEKHHVDQPIDYSLKYTTDI-PSSQKPAFSFSKNSSGQSTKTEHI    1211
Monkey         E-EERPTNYSIK-YNEEKHHVDQPIDYSLKYATDI-PSSQKQSFSFSKSSSGQSTKTEHI    1211
Human          E-EERPTNYSIK-YNEEKRHVDQPIDYSLKYATDI-PSSQKQSFSFSKSSSGQSSKTEHM    1211
                                    : * ::.  *

Sea Squirt     -------------------EDP---------------------LFRDAGDENAINQDQPK    1023
Sea Urchin     MSSEEPDVRDSSYNASSNQEIPSSVVSQFGSEHGGSRHSIASSHHEDEEEPPCSHDDNTK    1201
Zebrafish      SQDSSSSVASLKNQGRQKQLHPSSAQSRSGPTR-------AAVQKNPTCKAPTINQETLQ    1234
Frog           SSNS-NTPTPSPNSNRQNQLHPNSAQSRPGLNR----------PKQIPNKPPSINQETIQ    1265
Chicken        SSSSGNTSAPSAGSKRQNQLHPSSAQSRGGHA-----------QKTASCKTPSINQETIQ    1264
Opossum        SSGG-NTSTAPASTKRQNQLLPSSAQNRSGHT-----------QKTASCKAPSINQETIQ    1260
Mouse          SPSSENTAVPPSNAKRQNQLRPSSAQRN-GQT-----------QKGTTCKVPSINQETIQ    1258
Cow            SSSSENTSTTSSNAKRQNQLHPSSAQSRSGQTP---------KATSSSCKVPSINQETIQ    1262
Monkey         SSSSENTSTPSSNAKRQNQLHPSSAQSRSGQTQ---------KA--ATCKVSSINQETIQ    1260
Human          SSSSENTSTPSSNAKRQNQLHPSSAQSRSGQPQ---------KA--ATCKVSSINQETIQ    1260
                                    *                          .    :::  :
```

```
Sea Squirt          EYKVEDTPACFTPRSAISDLPCEEEDDQNIHGDQNAQ-----------KKNEQA------        1066
Sea Urchin          TYCVEGTPGPISRCSSLSSLDLNEELDEVEKTKDDSAHSPSNDLQSTTPPNEP-------        1254
Zebrafish           TYCVEDTPICFSRGSSLSSLSSEEDEMESCKRNVNSA-SNYPTLPISEKQSTNN-VAADQ        1292
Frog                TYCVEDTPICFSRGSSLSSLSSAEDEIEGRERNSRGQ-ESNNTLQITEPKEISA-VSKDG        1323
Chicken             TYCVEDTPICFSRCSSLSSLSSAEDEI-GRDQSTRVT-DTNATLQISELKENSGALSAEA        1322
Opossum             TYCVEDTPICFSRCSSLSSLSSAEDEI-GRDQTTRGS-ETTNTLQIAELKENSASVSTGD        1318
Mouse               TYCVEDTPICFSRCSSLSSLSSADDEI-GCDQTTQEA-DSANTLQTAEVKENDVTRSAED        1316
Cow                 TYCVEDTPICFSRCSSLSSLSSAEDEV-GCDQTTQEA-ESANTLQIAEIKDNSGPRSNED        1320
Monkey              TYCVEDTPICFSRCSSLSSLSSAEDEI-GCDQTTQEA-DSANTLQIAEIKDKIGTRSTED        1318
Human               TYCVEDTPICFSRCSSLSSLSSAEDEI-GCNQTTQEA-DSANTLQIAEIKEKIGTRSAED        1318
                     * **.**  ::  *::*.*   ::      .                 .

Sea Squirt          -------YHNNVE------------------DDKVDLQPGAMTPAFSTRGRKSGTMTPKG        1101
Sea Urchin          -------TPEKVTN------------------KQVL------------LQLPSDVHHHDE        1277
Zebrafish           RTSESQSSVHYVRAKPPRHHL--GHGDGSRHHKTVEFSSGAKSPSKSGAQTP-KSPPEHY        1349
Frog                AVNETRSSVHHTRTKNNRLQTSNISPSDSSRHKSVEFSSGAKSPSKSGAQTP-KSPPEHY        1382
Chicken             AVSEITSTSQHIRTKSSRLPTSSLSPSESSRHKAVEFSSGAKSPSKSGAQTP-KSPPEHY        1381
Opossum             TGSEVPSTSQHIRTKANRLQTTSLSPSDSTRHKAVEFSSGAKSPAKSGAQTP-KSPPEHY        1377
Mouse               PATEVPAVSQNARAKPSRLQASGLSSESTRHNKAVEFSSGAKSPSKSGAQTP-KSPPEHY        1375
Cow                 SVSKVPAGSQHIRTKSSRLQASGLSSESARH-KAVEFSSGAKSPSKSGAQTP-KSPPEHY        1378
Monkey              PVSEVPAVSQHTRTKSSRLQGSSLSSESTRH-KAVEFSSGAKSPSKSGAQTP-KSPPEHY        1376
Human               PVSEVPAVSQHPRTKSSRLQGSSLSSESARH-KAVEFSSGAKSPSKSGAQTP-KSPPEHY        1376
                             .                       . *                       .

Sea Squirt          YQETPMMFSRCSSMCSLSSFEAPSVQSQVESEP--SRFCSGVISPSELPDSPGQSMPVSR        1159
Sea Urchin          VQETPLVFSRCSSVCSLSSDDVPDICDDVSSIYTNSRAASGFVSPSELPDSPSDTMPPSP        1337
Zebrafish           VQETPLMFSRCTSVSSLDSFESHSIASSVQSEP-CSGMVSGIISPSDLPDSPGQTMPPSR        1408
Frog                VQETPLMFSRCTSGSSLDSFESHSIASSIASSVASEHMISGIISPSDLPDSPGQTMPPSR        1442
Chicken             VQETPLMFSRCTSVSSLDSFESRSIASSVQSEP-CSGMVSGIISPSDLPDSPGQTMPPSR        1440
Opossum             VQETPLMFSRCTSVSSLDSFESRSIASSVQSEP-CSGMVSGIISPSDLPDSPGQTMPPSR        1436
Mouse               VQETPLVFSRCTSVSSLDSFESRSIASSVQSEP-CSGMVSGIISPSDLPDSPGQTMPPSR        1434
Cow                 VQETPLMFSRCTSVSSLDSFESRSIASSVQSEP-CSGMVSGIISPSDLPDSPGQTMPPSR        1437
Monkey              VQETPLMFSRCTSVSSLDSFESRSIASSVQSEP-CSGIVSGIISPSDLPDSPGQTMPPSR        1435
Human               VQETPLMFSRCTSVSSLDSFESRSIASSVQSEP-CSGMVSGIISPSDLPDSPGQTMPPSR        1435
                     ****::****:* .**.* :  .: ..: *    .    **.:***:*****.::** *

Sea Squirt          SRSYSNLNDVLKDPSYISKPLPNAQPNVSNSEQKEEKKPQILQQLSRASEHVRAGYVTSL        1219
Sea Urchin          RRTSQS---------NVKVDL-------------------------------LNAPKL        1355
Zebrafish           SKTPPPPPPRSTS---VKQKV----TVPPHT-EKHDLAPRH------AVVSAAVQKVQVL        1454
Frog                SKTPP-----PPQTVQAKKDGSK--PIVPDE-ERG--KVAK------TAVHSAIQRVQVL        1486
Chicken             SKTPP-----PAQGVQVKRDVTK--GKVPTA-EKREPGPRQ------AAVNAAVQRVQVL        1486
Opossum             SKTPP-----PPQTGQTKREATK--SKLPSA-EKRESGPRQ------AAVNAAVQRVQVL        1482
Mouse               SKTPP----PPPQTVQAKREVPK--SKVPAA-EKRESGPKQ------TAVNAAVQRVQVL        1481
Cow                 SKTPPPPPPPPPQTVQTKQEVPK--NKAPSA-EKRESGPKQ------AAVNAAVQRVQVL        1488
Monkey              SKTPPP----PPQTAQTKREVPK--NKTPTA-EKRESGPKQ------AAVNAAVQRVQVL        1482
Human               SKTPPP----PPQTAQTKREVPK--NKAPTA-EKRESGPKQ------AAVNAAVQRVQVL        1482
                     ::               .                                    .  *

Sea Squirt          PQ-NDEIKCYNSEPAM---SDMTAFSGLSIGGDKVA---PLGILKTSTD-----SSHPEL        1267
Sea Urchin          PEMEEKPTMYATEGTPIDNSCATSLSALTIDGDVK-ATVAKRLLPPKGNEGSENVGSEDK        1414
Zebrafish           PD-NDTLLHFATESTPDGFSCASSLSALSLDEPFIQKDVELKIMPPVHEDDHSIEAEPEM        1513
Frog                QE-ADTLLHFATESTPDGFSCASSLSALSLDEPYIQKDVQLKIMPPVLENDQGNKAEPEK        1545
Chicken             PD-ADTLLHFATESTPDGFSCSSSLSALSLDEPFIQKDVELRIMPPVHENEHGNEAEPEQ        1545
Opossum             PD-ADTLLHFATESTPDGFSCSSSLSALSLDEPFIQKDVELRLMPPVQENEHGNETEPEQ        1541
Mouse               PD-VDTLLHFATESTPDGFSCSSSLSALSLDEPFIQKDVELRIMPPVQENDNGNETESEQ        1540
Cow                 PE-ADTLLHFATESTPDGFSCSSSLSALSLDEPFIQKDVELRIMPPVQENDNGNETESEQ        1547
Monkey              PD-ADTLLHFATESTPDGFSCSSSLSALSLDEPFIQKDVELRIMPPVQENDNGNETESEQ        1541
Human               PD-ADTLLHFATESTPDGFSCSSSLSALSLDEPFIQKDVELRIMPPVQENDNGNETESEQ        1541
                     :  :    : :* :    *  :::*.*::.          ::    :          :

Sea Squirt          EK--------------------AGNRFV------TQAGYITSLPTADEIKNYGC----EG        1297
Sea Urchin          E-----ITDEKNVEPS-------GNEFSEQEQSLLDECINAAMPVRRKGIPKPT------        1456
Zebrafish           EDMHEPKVQEKSPATSEAAKDILDDSDDDDDTEILNACINSAMPTKSSRKPKKQ---STS        1570
Frog                E--FIDNKAKKEDKRSEQEKDMLDDT--DDDIDILEECIISAMPRKPSRKNKKVPQPTPG        1601
Chicken             SDETKDNQENKAEKPSEAEKDILDDSD-DDDIEILEECIISAMPTKSSRKAKKPSQASAP        1604
Opossum             LEDVSEVKEKKAEKPTESEKDILDDSD-DDDIEILEECIISAMPTKSSRKAKKPSQA-AS        1599
Mouse               PEESNENQDKEVEKP-DSEKDLLDDSD-DDDIEILEECIISAMPTKSSRKAKKLAQT-AS        1597
Cow                 PEESNENQEKEAEKPTDSEKDLLDESD-DDDIEILEECIISAMPTKSSRKAKKPAQT-TS        1605
Monkey              PKESNENQEKEAEKTIDSEKDLLDDSD-DDDIEILEECIISAMPTKSSRKAKKPAQT-AS        1599
Human               PKESNENQEKEAEKTIDSEKDLLDDSD-DDDIEILEECIISAMPTKSSRKAKKPAQT-AS        1599
                                             .:          :    :::*   .
```

```
Sea Squirt       TLSPM-TSL-SEISVLRDHNEGFVKSTSGASPLKSRTGSHKSTSRS----SNIAHLASLP    1351
Sea Urchin       ------KSKSKREITS------------RKIPVSHKPSSSKHVSFEKSNAAPPTTMQDLP    1498
Zebrafish        RIPPPVACKPSQLPVY------------KLLPPQNRGQPQKHVALA--------HGEDMP    1610
Frog             KPPPPVARKPSQLPVY------------KLLSSQNRLQTQKHVNFT--------HSDDMP    1641
Chicken          KIPPPVARKPSQLPVY------------KLLPSQSRLQSQKHVSFT--------PGDDMP    1644
Opossum          KIPPPVARKPSQLPVY------------KLLPSQNRLQAQKHVSFT--------PGDDVP    1639
Mouse            KLPPPVARKPSQLPVY------------KLLPAQNRLQAQKHVSFT--------PGDDVP    1637
Cow              KLPPPVARKPSQLPVY------------KLLPSQNRLQAQKHVSFT--------PGDDMP    1645
Monkey           KLPPPVARKPSQLPVY------------KLLPSQNRLQPQKHVSFT--------PGDDMP    1639
Human            KLPPPVARKPSQLPVY------------KLLPSQNRLQPQKHVSFT--------PGDDMP    1639
                           ..  .                  . :    * .              .:*

Sea Squirt       ESSRSSGTHASTSSG--------------SKSDHALPVT-DENDHLDRIDD---------    1387
Sea Urchin       KPFCTEDTPVNFSAATSLSDLSIDDIEIESSSDGKLMIANPDRSALEKPSGIESDNQVRT    1558
Zebrafish        RVYCVEGTPINFSTATSLSDLTIDSPPNELAGMESSA-PHVEA-SGQRRDTL------PE    1662
Frog             RVYCVEGTPINFSTATSLSDLTIESPPSEPTND-QPN-TDSLSTDLEKRDTI------PT    1693
Chicken          RVYCVEGTPINFSTATSLSDLTIESPPNELANVDSVG-AGAESGEFEKRDTI------PT    1697
Opossum          RVYCVEGTPINFSTATSLSDLTIESPPNELAGVEGTS-TGALLGDFEKRDTI------PT    1692
Mouse            RVYCVEGTPINFSTATSLSDLTIESPPNELATGDGVR-AGIQSGEFEKRDTI------PT    1690
Cow              RVYCVEGTPINFSTATSLSDLTIESPPNELAAGEGVR-AGAQSSEFEKRDTI------PT    1698
Monkey           RVYCVEGTPINFSTATSLSDLTIESPPNELAAGEGVR-AGAQSGEFEKRDTI------PT    1692
Human            RVYCVEGTPINFSTATSLSDLTIESPPNELAAGEGVR-GGAQSGEFEKRDTI------PT    1692
                 .    ..*  . *:.                               :: .

Sea Squirt       LGDVDDLS---------DSGSLGSLSDGEDLLQACIQSAIPKKSSSRSSSTKPSSRGKDK    1438
Sea Urchin       SGDLLHCMDDAR-----SETNSVTKEAEDSMLQECINSAIPRSKSCKPRSSLLAKHRSPR    1613
Zebrafish        -GKSAEAKETGLSPPM-QSA--LAENEGDDILAECINSAMPKSKIHKPFRVQKMP----D    1714
Frog             EGRSTDDTDASKPLNP-TTVLDEDKAEEGDILAECIHSAMPKGKSHKPYRVKKIM----D    1748
Chicken          EGRSTDDTQRAKSITVTGPGLDDDKTEEGDILAECINSAMPKGKSHKPFRVKKIM----D    1753
Opossum          EGRSTDDIQIGKSSNVNTSAFDDNKTEEGEILAECINSAMPKGKSHKPFRVKKIM----D    1748
Mouse            EGRSTDDAQRGKISSIVTPDLDDNKAEEGDILAECINSAMPKGKSHKPFRVKKIM----D    1746
Cow              EGRSTDEAQRGKASSVTVPELDDSKTEEGDILAECINSAMPKGKSHKPFRVKKIM----D    1754
Monkey           EGRSTDEAQGGKTSSVTIPELDDNKAEEGDILAECINSAMPKGKSHKPFRVKKIM----D    1748
Human            EGRSTDEAQGGKTSSVTIPELDDNKAEEGDILAECINSAMPKGKSHKPFRVKKIM----D    1748
                  *   .                       .:*  **:**:*: .  :

Sea Squirt       SMGRK-ANTSKHPVKPAPKKEEPISKQSPLSPPAQVRPVRKFDIDSSLAAAENKLQGMPI    1497
Sea Urchin       QIGKFRSPKGVAPRKRLPSADVRHKSPSPIPEMSS---------DTRSTSSQER------    1658
Zebrafish        QAQHPSTATGS---LV-QQDLEKKKPTSPVKPMPQ---------SSEYRARMLK------    1755
Frog             QINHTSAATSSGNSRS-MQETDKNKPTSPVKPMPQ---------SIGFKERLKK------    1792
Chicken          QIQQASTS---LNNKN-QPEGEKKKPTSPVKPVPQ---------NSEYRARVRK------    1794
Opossum          QIQQASAS-SSGNSKN-QPEGEKKKPTSPVKPMPQ---------SAEYRTRIRK------    1791
Mouse            QVQQASST-SSGANKN-QVDTKKKKPTSPVKPMPQ---------NTEYRTRVRK------    1789
Cow              QVQQASMS-SSGTNKN-QLDGKTKKPTSPVKPIPQ---------NTEYRTRVRK------    1797
Monkey           QVQQASAS-SSATNKN-QLDGKKKKPTSPVKPIPQ---------NTEYRTRIRK------    1791
Human            QVQQASAS-SSAPNKN-QLDGKKKKPTSPVKPIPQ---------NTEYRTRVRK------    1791
                 .  :                    .  **:    .         .        :

Sea Squirt       LQYIEKKISEAPGLSDEDKETHAHPTTSNPSKSDTLDASDMTLTSQDITLNQSALDASDA    1557
Sea Urchin       --GQDWGNNPSPPPDTVTT----YCMEGTPGISNATSLSDLTS-----P-----IDSE--    1700
Zebrafish        --RPEANNS----LADPAT----YPDKNKETR-------KQEP-----------------    1781
Frog             --NTELKLN----PNSENQ----YCDP-R----------KPSS-----------------    1814
Chicken          --NTESKSQ----INNERS----YPEN-RDAK-------KQNL-----------------    1819
Opossum          --NAESK-N----VNVERS----YS-D-KDSK-------KLSL-----------------    1814
Mouse            --NTDSKVN----VNTEET----FSDN-KDSK-------KPSL-----------------    1814
Cow              --NTDSKNN----LNAERN----FSEN-KDSK-------KQHL-----------------    1822
Monkey           --NADSKNN----LNAERV----FSDN-KDSK-------KQNL-----------------    1816
Human            --NADSKNN----LNAERV----FSDN-KDSK-------KQNL-----------------    1816
                     :   .              .               .

Sea Squirt       ARRSKTCREVIKTVETLNESYSVKKQLKVFEKQRTDVTTPTTSQSPCNVASRLVPFPSMS    1617
Sea Urchin       -----------------------DVNGNITSQN-----------LPQNRS-------TLG    1719
Zebrafish        -----------------------KVVIRDFADK-----------PSNAE-----------    1796
Frog             -----------------------KKPSKVANEK-----------IPNNE-----------    1829
Chicken          -----------------------KNNSRDFNDK-----------LPNNE-----------    1834
Opossum          -----------------------KNNARDFLDK-----------MPNNE-----------    1829
Mouse            -----------------------QTNAKAFNEK-----------LPNNE-----------    1829
Cow              -----------------------KNNSKDFNDK-----------LPNNE-----------    1837
Monkey           -----------------------KNNSKDFNDK-----------LPNNE-----------    1831
Human            -----------------------KNNSKDFNDK-----------LPNNE-----------    1831
                                        .   .   .:
```

```
Sea Squirt     NNVKVQAFL-QSRPNTFMTEGTPVEMSCSTSLSNITIESGSDNYDFAQLQKQMVEQ--HR    1674
Sea Urchin     VGVSRGSTPSDTPR-VFAVEGTPINFSCNGSLSSLSCDEDA---ELTEAKAQMKEVSKRN    1775
Zebrafish      -ERTRPGFAFDSPHHYTPIEGTPYCFSRNDSLSSLDFEDED--LDFSKEKAVLRKDKEQR    1853
Frog           -ERTKG-FAFDSPHHYTPIEGTPYCFSRNDSLSSLDFEDDD--IDLSKEKAELRKEKGTK    1885
Chicken        -ERVRGSFTFDSPHHYTPIEGTPYCFSRNDSLSSLDFDDDD--VDLSREKAELRKGKEAK    1891
Opossum        -DRVRGSFTFDSPHHYTPIEGTPYCFSRNDSLSSLDFDDDD--VDLSREKAEL-KGKEAK    1885
Mouse          -DRVRGSFALDSPHHYTPIEGTPYCFSRNDSLSSLDFDDDD--VDLSREKAELRKGKESK    1886
Cow            -DRVRGSFTFDSPHHYTPIEGTPYCFSRNDSLSSLDFDDDD--VDLSREKAELRKGKENK    1894
Monkey         -DRVRGSFAFDSPHHYTPIEGTPYCFSRNDSLSSLDFDDDD--VDLSREKAELRKAKENK    1888
Human          -DRVRGSFAFDSPHHYTPIEGTPYCFSRNDSLSSLDFDDDD--VDLSREKAELRKAKENK    1888
                         ::       ****  :* . ***.:  :.     :::. :  : :    .

Sea Squirt     EPPQEMKYSTTTQSDKPTIQSNNKSEQ--------NNTDLFQHAEVPNNPTCSSSQEILN    1726
Sea Urchin     QAGGGMSRQQTVIPKQR-ASFSNPIEQVQSPENKYYSMDDYHNAEASKSAM---------    1825
Zebrafish      KVPLLKCS-VEQPANTNMVSTFQTAPTKPL----------QKTVFPQAPKEN---TVVVC    1863
Frog           DTDQKVKYKHENRAINPMGKQDQTGPKSLGGRDQPKALVQKPTSFSSAAKGTQDRGGATD    1913
Chicken        EVETKDCPNVEQPSGQQPSNRTQVCQKHPTSRSQSK-------TFCQPSKDIPDRGAATD    1944
Opossum        ETEAKVSNHLELTSNQQSANRAQICAKHPVERGQSKPLLQKQSTFPQSSKDMPDRVAATD    1945
Mouse          DSEAKVTCRPEPNSSQQAASKSQASIKHPANRAQSKPVLQKQPTFPQSSKDGPDRGAATD    1946
Cow            ESEAKVTNHTELTSNQQSASKTPAVTKQPINRGQSKPVLQKQPTFPQSSKDIPDRGAATD    1954
Monkey         ESEAKVTSHTELTSNQQSASKTQAIAKHPINRGQLKPILQKQSTFPQSSKDIPDRGAATD    1948
Human          ESEAKVTSHTELTSNQQSANKTQAIAKQPINRGQPKPILQKQSTFPQSSKDIPDRGAATD    1948
                .                       *

Sea Squirt     NLNKQIESLTIQNTETCTSGTGYVT------SLPHSD----EIKQFKTEGSVGAYTIFSA    1776
Sea Urchin     ------SPLDSPRVFAVEGTPGIISRADSLSSLSCDEEDASPAVEKSAKERLAEKTRTS-    1878
Zebrafish      DEKQKFSIEDTPVCFSRNSSLSSL------------------------------------    1925
Frog           EKMENFAIENTPVCFSRNSSLSSL------------------------------------    1969
Chicken        EKMQNFAIENTPVCFSRNSSLSSL------------------------------------    1968
Opossum        EKLQNFAIENTPVCFSRNSSLSSL------------------------------------    1969
Mouse          EKLQNFAIENTPVCFSRNSSLSSL------------------------------------    1970
Cow            EKLQNFAIENTPVCFSRNSSLSSL------------------------------------    1978
Monkey         EKLHNFAIENTPVCFSHNSSLSSL------------------------------------    1972
Human          EKLQNFAIENTPVCFSHNSSLSSL------------------------------------    1972

Sea Squirt     LTIDSETKIVPKGPEALPGRTN--DESPPKRSPTQSKIPVRTPPPVLKEEKSRKSSESSL    1834
Sea Urchin     --RISGSRMNHLGEGSMAMRRLSSEEAP-------LSYAVEDTPTCFS----HNSSLSAL    1925
Zebrafish      ------------------------------------------------------------    1923
Frog           ------------------------------------------------------------    1969
Chicken        ------------------------------------------------------------    1968
Opossum        ------------------------------------------------------------    1969
Mouse          ------------------------------------------------------------    1970
Cow            ------------------------------------------------------------    1978
Monkey         ------------------------------------------------------------    1972
Human          ------------------------------------------------------------    1972

Sea Squirt     E--------------AKEEAASASQHYGEDLINVVKDNPLNFGVEDTPFHLSGNNSSLSS    1880
Sea Urchin     SDNDEEERPDLTEQW-HDDESQPQRSSGGANGINKDSRRVFATEDTPVCFSR-NSSLSS    1983
Zebrafish      SDIDQENNNKDCSHK-----DDVTQ-MEAPRPQASGYAPKAFHVEDTPVCFSR-NSSLSS    1976
Frog           SDIDQENN-NKETEPLKQTGTSETQ-LGLRRPQTSGYAPKSFHVEDTPVCFSR-NSSLSS    2026
Chicken        SDIDQENNNNKEGEPVKRTEAPDSQ-IESSRPQTSGYAPKSFHVEDTPVCFSR-NSSLSS    2026
Opossum        SDIDQENNNNKESEPTKETEPPDSQ-GEPNRPQTSGYAPKSFHVEDTPVCFSR-NSSLSS    2027
Mouse          SDIDQENNNNKESEPIKEAEPANSQ-GEPSKPQASGYAPKSFHVEDTPVCFSR-NSSLSS    2028
Cow            SDIDQENNNNKENEPVKETEPPASQ-GEPGKPQASGYAPKSFHVEDTPVCFSR-NSSLSS    2036
Monkey         SDIDQENNNNKENEPIKETEPPDSQ-GEPSKPQASGYAPKSFHVEDTPVCFSR-NSSLSS    2030
Human          SDIDQENN-NKENEPIKETEPPDSQ-GEPSKPQASGYAPKSFHVEDTPVCFSR-NSSLSS    2029
                .                      *                * .****. :*  ******

Sea Squirt     IEIDSDGE---GNDLLNACISSAIPSATYNMNPPPEPMHSH-------------------    1918
Sea Urchin     LDAESDGDAASEQALLDECITSGMPQSKVKKPVKRI------------NGKIISGGPSSM    2031
Zebrafish      LSIDS------EDDLLQECISSAMPKKKKQTPRSKTEESGVKEEKSMMADGILSEEPDLI    2030
Frog           LSIDS------EDDLLQECISSAMPKKRKPSKIKNE----VGKSRSNSVGGILAEEPDLT    2076
Chicken        LSIDS------EDDLLQECISSAMPKKKKPSRIKSE----SEKSNSRNIGGMLAE--DLT    2074
Opossum        LSIDS------EDDLLQECISSAMPKKKRPSRFKGD----DEKPSPRNMDGILAE--DLT    2075
Mouse          LSIDS------EDDLLQECISSAMPKKKRPSRLKSE----SEKQSPRKVGGILAE--DLT    2076
Cow            LSIDS------EDDLLQECISSAMPKKKKPSRLKPD----NEKHSPRNMGGILAE--DLT    2084
Monkey         LSIDS------EDDLLQECISSAMPKKKKPSRLKGD----NEKHSPRNMGGMLAE--DLT    2078
Human          LSIDS------EDDLLQECISSAMPKKKKPSRLKGD----NEKHSPRNMGGILGE--DLT    2077
               :. :*       : **: **:*.:*.
```

```
Sea Squirt         -YNENHHRPSTLQADSTYQSNAIWVEMNKGIPQGQHLLNAISPLSLVSLTSPDPTSNSFS      1977
Sea Urchin         ------------------------------------------------------------      2031
Zebrafish          LDLTDTHSPISEQALSPDSESFDWKAIQEGANSIVSSLH---------------------      2069
Frog               LDLRDIQSPDSENAFSPDSENFDWKAIQEGANSIVSRLH---------------------      2115
Chicken            LDLREIQRPDSEHGFSPDSENFDWKAIQEGANSIVSSLH---------------------      2113
Opossum            LDLRDIQRPDSEHGFSPDSENFDWKAIQEGANSIVSSLH---------------------      2114
Mouse              LDLKDLQRPDSEHAFSPDSENFDWKAIQEGANSIVSSLH---------------------      2115
Cow                LDLKDIQRPDSEHGLSPDSENFDWKAIQEGANSIVSSLH---------------------      2123
Monkey             LDLKDIQRPDSEHGLSPDSENFDWKAIQEGANSIVSSLH---------------------      2117
Human              LDLKDIQRPDSEHGLSPDSENFDWKAIQEGANSIVSSLH---------------------      2116

Sea Squirt         ITSAGTGTQTNEDYYRLSRQPSVG-D---SIGSEISIGSLV-LGGDAKSLVKTQNAGRPK      2032
Sea Urchin         -------------------------------------EDDDSSPANQENEPKPVRKG-PR      2053
Zebrafish          --------QAA---ASLSRQGSSDSDSILSLKSGISIGSPFHLPLNQDDKPA-PNKG-PR      2116
Frog               --------QAA-AAGSLSRQGSSDSDSILSLKSGISLGSPFHLTLDKEEKTITSNKG-PK      2165
Chicken            --------QAA-AAASLSRQASSDSDSILSLKSGISLGSPFHLTPDQEEKPFTSNKG-PR      2163
Opossum            --------QAA-AAACLSRQASSDSDSILSLKSGISLGSPFHLTPDQEEKPFTSNKG-PR      2164
Mouse              --------QAAAAAACLSRQASSDSDSILSLKSGISLGSPFHLTPDQEEKPFTSNKG-PR      2166
 Cow               --------QAA-AAACLSRQASSDSDSILSLKSGISLGSPFHLTPDQEEKPFTSNKG-PR      2173
Monkey             --------QAA-AAACLSRQASSDSDSILSLKSGISLGSPFHLTPDQEEKPFTSNKG-PR      2167
Human              --------QAA-AAACLSRQASSDSDSILSLKSGISLGSPFHLTPDQEEKPFTSNKG-PR      2166
                                                         .      : ..     . * *:

Sea Squirt         ISKG-KD--VER-VSSDDKPPVVKGGKKIYRSPITGKPRENLRYPNKHKDNT--------      2080
Sea Urchin         ITKPSASVEEKSVNEEEEGPKGVKGGKKIYRSPITGKIRSITPPKSVLPPKSPSSTRGGL      2113
Zebrafish          ILKPGEKSSIEAKKKEEETAKSLKGGKKVYKSLITGKPRPSLES--MAS--------QHR      2166
Frog               ILKPAEKSALENKKTEE-EPKGIKGGKKVYKSLITGKSRSSSDFSSHCK--------QSV      2216
Chicken            ILKPGEKSTLESKKVES-ESKGIKGGKRVYKSIITGKARSNSEVSSQIK--------QPQ      2214
Opossum            ILKPGEKSTLETKKIES-ENKGIKGGKKVYKSLITGKVRSNSEVSGQLK--------QPL      2215
Mouse              ILKPGEKSTLEAKKIES-ENKGIKGGKKVYKSLITGKIRSNSEISSQMK--------QPL      2217
Cow                ILKPGEKSTLETKKIES-ENKGIKGGKKVYKSLITGKVRSNSEISSQMK--------QPL      2224
Monkey             ILKPGEKSTLETKKIES-ESKGIKGGKKVYKSLITGKVRSNSEISGQMK--------QPL      2218
Human              ILKPGEKSTLETKKIES-ESKGIKGGKKVYKSLITGKVRSNSEISGQMK--------QPL      2217
                   * *   .   :    ..     :****::*:* **** *

Sea Squirt         -------STGSSSARIREHPRNNRNSGRSKSPC-----NKCHPPHQSR------QLPISS      2122
Sea Urchin         AKGSPTTSRGRGAIRG--ARGGFARSASTTPPRSSTPTGRGTPPRTTTPPRTTSPMTAGR      2171
Zebrafish          QAQAPVISRGRTMVHVP----GVRSSSPSTSPV-----PKKPPPRG--------QMSKPP      2209
Frog               QTNMPSISRGRTMIHIP----GVRASSPSTSPV-----SKKGPVFKN-------VPSKGS      2260
Chicken            QTSVPSISRGRTMIHIP----GVRNSSSSTSPV-----SKKGPPFKN-------TNSKSP      2258
Opossum            PTNMPSISRGRTMIHIP----GIRNSSSSTSPV-----SKKGPSLKT-------PTSKSP      2259
Mouse              PTNMPSISRGRTMIHIP----GLRNSSSSTSPV-----SKKGPPLKT-------PASKSP      2261
Cow                QTNMPSISRGRTMIHIP----GVRNSSSSTSPV-----SKKGPPLKT-------PASKSP      2268
Monkey             QANMPSISRGRTMIHIP----GVRNSSSSTSPV-----SKKGPPLKT-------PASKSP      2262
Human              QANMPSISRGRTMIHIP----GVRNSSSSTSPV-----SKKGPPLKT-------PASKSP      2261
                          * *    :      .   *. :. *       :  *

Sea Squirt         SMGKLA------------------------------------------------------      2128
Sea Urchin         TTTPPRTTSPRTTSPRSGTPPKPKPKGSIA---NKSITPVRNATNGARSITPPRPVRKSSVD      2228
Zebrafish          SQAPGAGSSPRTMKVPPSSEPSPASG--PPSSQGGSSKASSRSGSRDSTPSRPVQQSLTR      2267
Frog               NENPSSSSSPKGTKPLKS-ELVY--GSRPSSTPGGSSKGNSRSGSRDSASSRPSPQPLSR      2317
Chicken            SEGQSSASSPRGVKSSVKPEPAPVTRQLSGLNQGGSSKGPSRSGSRDSTPSRPQQQPLSR      2318
Opossum            SEGPTSSTSPRGAKPSVKSELSPVTRQTS--QPGGSSKGPSRSGSRDSTPSRPSQQPLSR      2317
Mouse              SEGPGATTSPRGTKPAGKSELSPITRQTS--QISGSNKGSSRSGSRDSTPSRPTQQPLSR      2319
Cow                SEGQPATTSPRGTKPSVKSELSPVTRQAS--QTAGSNKGPSRSGSRDSTPSRPAQQPLSR      2326
Monkey             SEGQTATTSPRGAKPSVKSELSPVARQTS--QIGGSSKAPSRSGSRDSTPSRPAQQPLSR      2320
Human              SEGQTATTSPRGAKPSVKSELSPVARQTS--QIGGSSKAPSRSGSRDSTPSRPAQQPLSR      2319
                   .

Sea Squirt         --------ETSPGRSHRKTN--------------ASST--------YSKQMQPTCGGNPH      2158
Sea Urchin         SQKDGDAKSETSSR--RSSKESISSIP-KPRQIKPQSTQTKTSPNSSTSPKPPTGRGSPA      2285
Zebrafish          PMQSPGRASVSPGRNGLSPSNKLSQLPQLPRTASPSASSTKSS-----------GS----      2312
Frog               PLQSPGRNSISPGKNGISPPNKFSQ---LPRTTSPSTASTKSS-----------GS----      2359
Chicken            PLQSPGRNSISPGRNGISPPNKLSQ---LPRTSSPSTASTKSS-----------SS----      2360
Opossum            PMQSPGRNSISPGRNGISPPNKLSQ---LPRTSSPSTSSTKSS-----------GS----      2359
Mouse              PMQSPGRNSISPGRNGISPPNKLSQ---LPRTSSPSTASTKSS-----------GS----      2361
Cow                PMQSPGRNSISPGRNGISPPNKLSQ---LPRTSSPSTASTKSS-----------GS----      2368
Monkey             PIQSPGRNSISPGRNGISPPNKLSQ---LPRTSSPSTASTKSS-----------GS----      2362
Human              PIQSPGRNSISPGRNGISPPNKLSQ---LPRTSSPSTASTKSS-----------GS----      2361
                           . : .:   .                 .::
```

```
Sea Squirt         GKPSRSRS--AHAGYQSSVTQI--YPPLKHVNSIM-----LNGVA-SMAARGDLVVQQLE     2208
Sea Urchin         GKPPLSTSRSSSPKSKMPVPSRSNSPSTKSSGSVTTPRKPLNKSPSSTKARDES-PTDAE     2344
Zebrafish          GRMAY-----TSPGRQLVQPTP-----TKQSGLPR-STSGIPRSESASKILNQC----GP     2357
Frog               GRMSY-----TSPGRQLSQPNL-----SKQSGLPK-THSSIPRSESASKSLNQNVNT-GS     2407
Chicken            GRMSY-----TSPGRQMSQQNL-----TKQTALTK-NTSSIPRSESASKGLNQILGSGAS     2409
Opossum            GKISY-----TSPGRQMSQQNL-----TKQTGLSK-NTSNIPRSESASKGLNQISNSNGT     2408
Mouse              GKMSY-----TSPGRQLSQQNL-----TKQASLSK-NASSIPRSESASKGLNQMSNGNGS     2410
Cow                GKMSY-----TSPGRQMSQQNL-----TKQTGLSK-NGSGIPRSESASKGLNQMSNSNGS     2417
Monkey             GKMSY-----TSPGRQMSQQNL-----TKQTGLSK-NASSIPRSESASKGLNQVNNGNGA     2411
Human              GKMSY-----TSPGRQMSQQNL-----TKQTGLSK-NASSIPRSESASKGLNQMNNGNGA     2410
                   *:        :    :            *           :     :    .:

Sea Squirt         GKTAQV----VPASTLANQFQHVSRPVLIKQGTFIQDEPSQALLQSMSRKPEVPKKP---     2261
Sea Urchin         EVTSQMKSMHLESNGSSPDLDDGDRPVLLKQSTFTKDSASLPEQTP-----EMNPVSGD-     2398
Zebrafish          SKKAELSRM-SSTKSSGSESDRSEKPGLVRQSTFIKEAPSPTLKRKLEESASFESLSPSS     2416
Frog               NKKVELSRM-SSTKSSGSESDRSERPALVRQSTFIKEAPSPTLRRKLEESASFESLSSSS     2466
Chicken            NKKTDLSRM-SSAKSSGSESDRSERPVLVRQSTFIKEAPSPTLRRKLEESASFESLSP-S     2467
Opossum            NKKVELSRM-SSTKSSGSESDRSERPVLVRQSTFIKETPSPTLRRKLEESASLESLSSSS     2467
Mouse              NKKVELSRM-SSTKSSGSESDRSERPALVRQSTFIKEAPSPTLRRKLEESASFESLSPSS     2469
Cow                NKKVELSRM-SSTKSSGSESDRSERPVLVRQSTFIKEAPSPTLRRKLEESASFESLSPSS     2476
Monkey             NKKVELSRM-SSTKSSGSESDRSERPVLVRQSTFIKEAPSPTLRRKLEESASFESLSPSS     2470
Human              NKKVELSRM-SSTKSSGSESDRSERPVLVRQSTFIKEAPSPTLRRKLEESASFESLSPSS     2469
                     . ::      :.  . : :  .:* *::*.** ::  *           ..

Sea Squirt         -AKSPPKAVK-----------SKKQSEPQQKSKTLWKGLKKLLSPSDEAKNIVPSPTRKT     2309
Sea Urchin         -------EKVLSPVQSEEAPSVTESESSVSLSKGGWKKTGSGIQGSQESKKSSTGN-KPV     2450
Zebrafish          T------SQSQTPVSSPSLPDMSL-SLP--YQGSGWTKAPQSQN-SAENGDGKSLKRHDI     2466
Frog               RADSPPRSQTQTPALSPSLPDMAL-STHS-IQAGGWRKMPPNLNPAAEH--GDSRRRHDI     2522
Chicken            RPDSPTRSQLQTPVLSPSLPDMSL-STHSTAQTSGWRKLPPNLSPSVEY-DGRPAKRHDI     2525
Opossum            RPDSPTKSQVQTPILSPSLPDMSL-STHSSIQTGSWRKLPPNLNPSIEFNDGRSTKRHDI     2526
Mouse              RPDSPTRSQAQTPVLSPSLPDMSL-STHPSVQAGGWRKLPPNLSPTIEYNDGRPTKRHDI     2528
Cow                RPDSPTRSQAHTPVLSPSLPDMSL-STHSSLQSGGWRKLPPNLSPTIEYNDGRPVKRHDI     2535
Monkey             RPASPTRSQAQTPVLSPSLPDMSL-STHSSVQAGGWRKLPPNLSPTIEYNDGRPAKRHDI     2529
Human              RPASPTRSQAQTPVLSPSLPDMSL-STHSSVQAGGWRKLPPNLSPTIEYNDGRPAKRHDI     2528
                                            .     .   *       . : *         :

Sea Squirt         PKKPQVTTRGKVVQNQS---DRVIESEDSSTGFNSGTWTKSKTGSTSSSLQSKQVR--R     2363
Sea Urchin         SK--TTGVSKITPRRTGSPGLRTPAARSSSPGQRVNTPARRSESP--SRMSSTSQRSES     2505
Zebrafish          SRSHSESPSRLPINRTGTWK--REHSKHSSSLPRVGTWKRTGSSS--SILSASSESSEK     2521
Frog               SRSHSESPSRLPITRSGTWK--REHSKHSSSLPRVSTWRRTGSSS--SILSASSESSEK     2577
Chicken            ARSHSESPSRLPINRSGTWK--REHSKHSSSLPRVSTWRRTGSSS--SILSASSESSEK     2580
Opossum            ARSHSESPSRLPVNRSGTWK--REHSKHSSSLPRVSTWRRTGSSS--SILSASSESSEK     2581
Mouse              ARSHSESPSRLPINRAGTWK--REHSKHSSSLPRVSTWRRTGSSS--SILSASSESSEK     2583
Cow                ARSHSESPSRLPINRSGTWK--REHSKHSSSLPRVSTWRRTGSSS--SILSASSESSEK     2590
Monkey             ARSHSESPSRLPINRSGTWK--REHSKHSSSLPRVSTWRRTGSSS--SILSASSESSEK     2584
Human              ARSHSESPSRLPINRSGTWK--REHSKHSSSLPRVSTWRRTGSSS--SILSASSESSEK     2583
                     :      .::   ::         :. **   . .*  :   .   * :.:..

Sea Squirt         ----------TPERPHRTPSNAGSQSSSLSLSSSSASNGSSSSVDVYQQPST-RMNIYPT     2412
Sea Urchin         PSRASTTSQSSVSKQRTTPQTKGPASTQRNNSATSKINTGLQKSGGMSKRSASPSGVKTN     2565
Zebrafish          GR-------SEDERQP-TN--PPQKSGKEG---------GLERKGTWRKAK--GSETSYA     2560
Frog               AK-------SEDEKQQ-VCSFPGPRS--EC---------SSSAKGTWRKIK--ESEILET     2616
Chicken            AK-------SEDEKQH-GSSLSGQKQSKES---------QAPAKGTWRKIK--ENEIPQI     2621
Opossum            AK-------SEDEKHV--SSLLGTKQTKEN---------QGPAKGTWRKIK--ESEMTPI     2621
Mouse              AK-------SEDERHV--SSMPAPRQMKEN---------QVPTKGTWRKIK--ESDISPT     2623
Cow                AK-------SEDEKQV--NSISGSKQTKEN---------QVSTKGTWRKIK--ESEISPT     2630
Monkey             AK-------SEDEKHV--NSISGTKQSKEN---------QVSAKGTWRKIK--ENEISPT     2624
Human              AK-------SEDEKHV--NSISGTKQSKEN---------QVSAKGTWRKIK--ENEFSPT     2623
                               .:           .                  .   :  .

Sea Squirt         ARNMSPGAHV---PK--------SPS------A-KSRIIVNSKSPADKTNQGWRRT-GDS     2453
Sea Urchin         --PNVNGTHASTPPKSTASRPNTRPTKSGNAGVRTGPPTTRVASASPKPSGASARAVRNK     2623
Zebrafish          --PM--------------------------------SLDLQDQTGDAMSKSEDVWV-RIEDC     2587
Frog               --PSNGSSST---------------IAESNCSLESKTKLVYQMAPAVSKTEDVWV-RIEDC     2658
Chicken            --MNDPQ-HP---------------SSSATSSSDSKTLIYQMAPAVSKTEDVWV-RIEDC     2662
Opossum            --SNVSQ-VT---------------SSGTTNGADSKTLIYQMAPAVSKTEDVWV-RIEDC     2662
Mouse              --GMASQ-SA---------------SSGAASGAESKPLIYQMAPPVSKTEDVWV-RIEDC     2664
Cow                --NSTSQ-TT---------------SSGAANGAESKTLIYQMAPAVSKTEDVWV-RIEDC     2671
Monkey             --NSTSQ-TV---------------SSGATNGAESKTLIYQMAPAVSKTEDVWV-RIEDC     2665
Human              --NSTSQ-TV---------------SSGATNGAESKTLIYQMAPAVSKTEDVWV-RIEDC     2664
                                                  .     .      * .        :
```

```
Sea Squirt    RIHSRKISME--------SSSSATSAAGN----------------RASGRKH--------      2481
Sea Urchin    PAS-RPSSAEGSRSLSGNNSSSKETPSPQTPSKFGTFTKK--------------------      2662
Zebrafish     PIN---------------NPRSSKSPTASTPPVIDSLPIKGLACDRDSSESHSKLMSENA      2632
Frog          PIN---------------NPRSGRSPTGNSPPVIDNVLDQGQKEE-AAKDCHTRHNSGNG      2702
Chicken       PIN---------------NPRSGRSPTGNTPPVIDSVSEKGVVNGKDSKEIQEKQNPGNG      2707
Opossum       PIN---------------NPRSGRSPTGNTPPVIDNVAEKVSSGNKESKDNQGKPNGGNG      2707
Mouse         PIN---------------NPRSGRSPTGNTPPVIDSVSEKGSSSIKDSKDTHGKQSVGSG      2709
Cow           PIN---------------NPRSGRSPTGNTPPVIDTVSEKGNPNPKDSKDNQGKQNVSNG      2716
Monkey        PIN---------------NPRSGRSPTGNTPPVIDSVSEKGNPN-KDSKDNQAKQNVGNG      2709
Human         PIN---------------NPRSGRSPTGNTPPVIDSVSEKANPNIKDSKDNQAKQNVGNG      2709
                                .  *  : : .

Sea Squirt    --------GSQAS-----IATSSDKRSVSVASGQSPSARLFYEVVENPSR-RN--PGEGA      2525
Sea Urchin    --------------KAQSSVETYDKKETERNSGESDSVS------------KSDLSSD--      2694
Zebrafish     ---AMSHLGSETNLNLLRSSESLDKKVTDIKPAPS-NPNIGPELHEFPVSERTPFSSTNS      2688
Frog          N-----VPLLENRQKSFIKVDGLDTKGTDPKSLINN----QQETNENTVAERTAFSSSSS      2753
Chicken       S-VPVRTIGLENRLNSFFQMDSPDKKGNETKPLQT-NPVPAPENNESTVSERTPFSSSSS      2765
Opossum       SSAPARTVGLENRLNSFIQIDSPDKKAAETKSGQV-NLVPAPETSETSVAERTPFSSTSS      2766
Mouse         --SPVQTVGLETRLNSFVQVEAPEQKGTEAKPGQS-NPVSIAETAETCIAERTPFSSSSS      2766
Cow           S-APTRTMGLENRLNSFIQVDPPDQKGTETKPGHSNNPVPASETSESSIAERTPFSSSSS      2775
Monkey        S-VPMRTVGLENRLNSFIQVDAPDQKGTETKPGQ-NNPVPVSETNESSIVERTPFSSSSS      2767
Human         S-VPMRTVGLENRLNSFIQVDAPDQKGTEIKPGQ-NNPVPVSETNESSIVERTPFSSSSS      2767
                                     : :  .                      :.   .

Sea Squirt    LPFPSPVRRVVQEVAPFNYRPYRIGESEEEEPNHKLDLTRKQTTV---------------      2570
Sea Urchin    ---DQKSSQLKAK-------------------DLCKELNIPVKNCGGKIVSPTRETAEGD      2732
Zebrafish     SKHSSPSGAVAARVSPFNYTPSPRKSSADGSTPRPSQIPTPISSNAKK------------      2736
Frog          SKHSSPSGTVAARVTPFNYNPSPRKSNGENSTSRPSQIPTPVTNSTKK------------      2801
Chicken       SKHNSPIGAVAARVTPFNYNPSRRKSSVDNSSARPSQIPTPVNNSTKK------------      2813
Opossum       SKHSSPSGTVAARVTPFNYNPSPRKSSADSSAARPSQIPTPVNNSTKK------------      2814
Mouse         SKHSSPSGTVAARVTPFNYNPSPRKSSADSTSARPSQIPTPVSTNTKK------------      2814
Cow           SKHSSPSGTVAARVSPFNYNPSPRKSSTDGTSARPSQIPTPVSNNTKK------------      2823
Monkey        SKHSSPSGTVAARVTPFNYNPSPRKSSADSTSARPSQIPTPVNNNTKK------------      2815
Human         SKHSSPSGTVAARVTPFNYNPSPRKSSADSTSARPSQIPTPVNNNTKK------------      2815
                  .    :  .                       ::     ..

Sea Squirt    ------------------------------------------------------------      2570
Sea Urchin    GIWMKRPEDEVKDCASVSIHSSSRHSSSYSLSSISTNHQVGSLSRQKSAQPELPSSKKSP      2792
Zebrafish     ------RDTKGDTT----------ESGSYIVTSV--------------------------      2754
Frog          ------RDSKTETTDSSGSQSPKRHSGSYLVTSV--------------------------      2829
Chicken       ------RDSKSENTDSSGTQSPKRHSGSYLVTSV--------------------------      2841
Opossum       ------RDSKTENTESSGTQSPKRHSGSYLVTSV--------------------------      2842
Mouse         ------RDSKTDSTESSGAQSPKRHSGSYLVTSV--------------------------      2842
Cow           ------RDSKPDSTEPSGTQSPKRHSGSYLVTSV--------------------------      2851
Monkey        ------RDSKTDSTESSGTQSPKRHSGSYLVTSV--------------------------      2843
Human         ------RDSKTDSTESSGTQSPKRHSGSYLVTSV--------------------------      2843

Sea Squirt    ------------------------------------------------------------      2570
Sea Urchin    AVNTRRNIFSSLNKNSNSKSSSKSSLDSSKSGGKSTPKMTSKQKHESEAKPEEKKDKKSE      2852
Zebrafish     ------------------------------------------------------------      2754
Frog          ------------------------------------------------------------      2829
Chicken       ------------------------------------------------------------      2841
Opossum       ------------------------------------------------------------      2842
Mouse         ------------------------------------------------------------      2842
Cow           ------------------------------------------------------------      2851
Monkey        ------------------------------------------------------------      2843
Human         ------------------------------------------------------------      2843

Sea Squirt    ------------------------------------------------------------      2570
Sea Urchin    KKRFSFLKLKIGGKKSDSDSEGKGKSKGFFSKKEKKSPKNLKVQTKSRSESDSMAEHVLP      2912
Zebrafish     ------------------------------------------------------------      2754
Frog          ------------------------------------------------------------      2829
Chicken       ------------------------------------------------------------      2841
Opossum       ------------------------------------------------------------      2842
Mouse         ------------------------------------------------------------      2842
Cow           ------------------------------------------------------------      2851
Monkey        ------------------------------------------------------------      2843
Human         ------------------------------------------------------------      2843

Sea Squirt    ------------------------------------------------------------      2570
Sea Urchin    SPPLAELEPDLNMSGADLNFDRHLDELEAWNSDNDIDNEVMPDEFVEWDSGSDIFKEEMP      2972
Zebrafish     ------------------------------------------------------------      2754
```

```
Frog            ------------------------------------------------------------    2829
Chicken         ------------------------------------------------------------    2841
Opossum         ------------------------------------------------------------    2842
Mouse           ------------------------------------------------------------    2842
Cow             ------------------------------------------------------------    2851
Monkey          ------------------------------------------------------------    2843
Human           ------------------------------------------------------------    2843

Sea Squirt      ------------------------------------------------------------    2570
Sea Urchin      EEFIESCSEVDTDPATPLSPGVTTGPGGGNIQIDPNGQRGGFSPHSETGSLSPNGLSGDC    3032
Zebrafish       ------------------------------------------------------------    2754
Frog            ------------------------------------------------------------    2829
Chicken         ------------------------------------------------------------    2841
Opossum         ------------------------------------------------------------    2842
Mouse           ------------------------------------------------------------    2842
Cow             ------------------------------------------------------------    2851
Monkey          ------------------------------------------------------------    2843
Human           ------------------------------------------------------------    2843

Sea Squirt      ------------------------------------------------------------    2570
Sea Urchin      MSPVDEGDLSMEAGEQYFYSENRLNSFIRLDDTKGETSDTFGEGELSISRNVQNQRSRSL    3092
Zebrafish       ------------------------------------------------------------    2754
Frog            ------------------------------------------------------------    2829
Chicken         ------------------------------------------------------------    2841
Opossum         ------------------------------------------------------------    2842
Mouse           ------------------------------------------------------------    2842
Cow             ------------------------------------------------------------    2851
Monkey          ------------------------------------------------------------    2843
Human           ------------------------------------------------------------    2843

Sea Squirt      ------------------------------------------------------------    2570
Sea Urchin      SDTAVEQSPHRPQRLKLPQKPFGFHQNDASPSSNNVVPVLVSPYNYSPNPNRKGEIDTNY    3152
Zebrafish       ------------------------------------------------------------    2754
Frog            ------------------------------------------------------------    2829
Chicken         ------------------------------------------------------------    2841
Opossum         ------------------------------------------------------------    2842
Mouse           ------------------------------------------------------------    2842
Cow             ------------------------------------------------------------    2851
Monkey          ------------------------------------------------------------    2843
Human           ------------------------------------------------------------    2843

Sea Squirt      -----------------------------    2570
Sea Urchin      SGMGFHKDDSSRSSSEVVSPTGSTRVTTV    3181
Zebrafish       -----------------------------    2754
Frog            -----------------------------    2829
Chicken         -----------------------------    2841
Opossum         -----------------------------    2842
Mouse           -----------------------------    2842
Cow             -----------------------------    2851
Monkey          -----------------------------    2843
Human           -----------------------------    2843
```
