# Supplementary material for: Curated multiple sequence alignment for the Adenomatous Polyposis Coli (APC) gene and accuracy of in silico pathogenicity predictions
Source: PLoS One. 2020 Aug 4;15(8):e0233673. doi: 10.1371/journal.pone.0233673 (PMC7402488; doi:10.1371/journal.pone.0233673)
Supplement: S2 Fig — PMSA was generated from the program Clustal Omega. No annotation is added. (PDF) [file pone.0233673.s002.pdf]

```
Ciona.intestinalis                  ESNRNGETNKELSRYDQLLQQVECLKLENTTLKKELSNNSSQISKLESEALNLKEMVVML    60
Strongylocentrotus.purpuratus       -----------MSSYDQLLHQVESLKAENSHLKMELKDNSSQLNKLDSVVGHLQSGKP--    47
Danio.rerio                         ---------MAAASYDQLLKQVEALKMENSNLRQELEDNSNHLNKLETEASNMKEVLKQL    51
Xenopus.tropicalis                  ---------MAAASYDQLLKQVAALKMENTNLRQELEDNSNHLTKLESEASNMKEVLKQL    51
Xenopus.laevis                      ---------MAAASYDQLVKQVEALTMENTNLRQELEDNSNHLTKLETEATNMKEVLKQL    51
Ornithorhynchus.anatinus            ---------MAAASYDQLLKQVEALKMENSNLRQELEDNSNHLTKLETEASNMKEVLKQL    51
Monodelphis.domestica               ---------MAAASYDQLLKQVEALKMENSNLRQELEDNSNHLTKLETEASNMKEVLKQL    51
Mus.musculus                        ---------MAAASYDQLLKQVEALKMENSNLRQELEDNSNHLTKLETEASNMKEVLKQL    51
Rattus.norvegicus                   ---------MAAASYDQLLKQVEALKMENSNLRQELEDNSNHLTELETEASNMKEVLKQL    51
Sorex.araneus                       ---------MAAASYDQLLKQVEALKMENSNLRQELEDNSNHLTKLETEASNMKEVLKQL    51
Octodon.degus                       ---------MAAASYDQLLKQVEALKMENSNLRQELEDNSNHLTKLETEASNMKEVLKQL    51
Heterocephalus                      ---------MAAASYDQLLKQVEALKMENSNLRQELEDNSNHLTKLETEASNMKEVLKQL    51
Jaculus.jaculus                     ---------MAAASYDQLLKQVEALKMENSNLRQELEDNSNHLTKLETEASNMKEVLKQL    51
Loxodonta.africana                  ---------MAAASYDQLLKQVEALKMENSNLRQELEDNSNHLTKLETEASNMKEVLKQL    51
Trichechus.manatus.latirostris      ---------MAAASYDQLLKQVEALKMENSNLRQELEDNSNHLTKLETEASNMKEVLKQL    51
Otolemur.garnettii                  ---------MAAASYDQLLKQVEALKMENSNLRQELEDNSNHLTKLETEASNMKEVLKQL    51
Callithrix.jacchus                  ---------MAAASYDQLLKQVEALKMENSNLRQELEDNSNHLTKLETEASNMKEVLKQL    51
Macaca.mulatta                      ---------MAAASYDQLLKQVEALKMENSNLRQELEDNSNHLTKLETEASNMKEVLKQL    51
Nomascus.leucogenys                 ---------MAAASYDQLLKQVEALKMENSNLRQELEDNSNHLTKLETEASNMKEVLKQL    51
Pongo.abelii                        ---------MAAASYDQLLKQVEALKMENSNLRQELEDNSNHLTKLETEASNMKEVLKQL    51
Homo.sapiens                        ---------MAAASYDQLLKQVEALKMENSNLRQELEDNSNHLTKLETEASNMKEVLKQL    51
Gorilla.gorilla                     ---------MAAASYDQLLKQVEALKMENSNLRQELEDNSNHLTKLETEASNMKEVLKQL    51
Pan.troglodytes                     ---------MAAASYDQLLKQVEALKMENSNLRQELEDNSNHLTKLETEASNMKEVLKQL    51
Pan.paniscus                        ---------MAAASYDQLLKQVEALKMENSNLRQELEDNSNHLTKLETEASNMKEVLKQL    51
Canis.lupus.familiaris              ---------MAAASYDQLLKQVEALKMENSNLRQELEDNSNHLTKLETEASNMKEVLKQL    51
Bos.taurus                          ---------MAAASYDQLLKQVEALKMENSNLRQELEDNSNHLTKLETEASNMKEVLKQL    51
Ovis.aries                          ---------MAAASYDQLLKQVEALKMENSNLRQELEDNSNHLTKLETEASNMKEVLKQL    51
Orcinus.orca                        ---------MAAASYDQLLKQVEALKMENSNLRQELEDNSNHLTKLETEASNMKEVLKQL    51
Sus.scrofa                          ---------MAAASYDQLLKQVEALKMENSNLRQELEDNSNHLTKLETEASNMKEVLKQL    51
Ceratotherium.simum.simum           ---------MAAASYDQLLKQVEALKMENSNLRQELEDNSNHLTKLETEASNMKEVLKQL    51
Equus.caballus                      ---------MAAASYDQLLKQVEALKMENSNLRQELEDNSNHLTKLETEASNMKEVLKQL    51
Felis.catus                         ---------MAAASYDQLLKQVEALKMENSNLRQELEDNSNHLTKLETEASNMKEVLKQL    51
Ailuropoda.melanoleuca              ---------MAAASYDQLLKQVEALKMENSNLRQELEDNSNHLTKLETEASNMKEVLKQL    51
Mustela.putorius.furo               ---------MAAASYDQLLKQVEALKMENSNLRQELEDNSNHLTKLETEASNMKEVLKQL    51
Odobenus.rosmarus.divergens         ---------MAAASYDQLLKQVEALKMENSNLRQELEDNSNHLTKLETEASNMKEVLKQL    51
Taeniopygia.guttata                 ---------MAAASYDQLLKQVEALKMENSNLRQELEDNSNHLTKLETEASTMKEVLKQL    51
Anolis.carolinensis                 ---------MAAVSYDQLLKQVEALKMENTNLRQELEDNSNHLTKLETEASNMKEVLKQL    51
Gallus.gallus                       ---------MAAASYDQLLKQVEALKMENSNLRQELEDNSNHLTKLETEASNMKEVLKQL    51
                                                  ****::** .*. **: *: **.:**.::.:*:: .  ::.

Ciona.intestinalis                  GNSIKPPTEASEPNDPRATGFVNPPNRPTALFPPHVYSP------DMYYR-YPMPNHHML    113
Strongylocentrotus.purpuratus       -GMGEDDLRAQI-TIHGLAMTTAAASATSSSAATSG---------AGPASSFQVNG-GAM    95
Danio.rerio                         QGSIDEDSKDSQGQIEFLERI-KEMSLDPSGFSGVKLRS------KASLQGSA-------    97
Xenopus.tropicalis                  QGSIEDEAMASSGQIDLLERL-KELNLDSSNFPAGKTRP------KMSMRSYGSRE-GSL    103
Xenopus.laevis                      QGSIEDEAMASSGPIDLLERF-KDLNLDSSNIPAGKARP------KMSMRSYGSRE-GSL    103
Ornithorhynchus.anatinus            QGSIEDEAMASSGQIDLLERL-KELNLDSGNFPGVKLRP------KMSMRSYGSRE-GSV    103
Monodelphis.domestica               QGSIEDEAMASSGQIDLLERL-KELNLDSSNFPGVKLRP------KMSIRSYGSRE-GSV    103
Mus.musculus                        QGSIEDETMT-SGQIDLLERL-KEFNLD-SNFPGVKLRS------KMSLRSYGSRE-GSV    101
Rattus.norvegicus                   QGSIEDETMT-SGQIDLLERL-KEFNLD-SNFPGVKLRS------KMSLRSYGSRE-GSV    101
Sorex.araneus                       QGSIEDEAMASAGQIDLLERL-KELNLDSSNFPGVKLRS------KMSLRSFGSRE-GSV    103
Octodon.degus                       QGSIEDEAMASSGQIDLLERL-KELNLDSSSFPGVKLRS------KMSLRSYGSRE-GSV    103
Heterocephalus                      QGSIEDEAMASSGQIDLLERL-KELNLDSSSFPGVKLRS------KMSLRSYGSRE-GSI    103
Jaculus.jaculus                     QGSIEDEAMASSGQMDLLERL-KELNLDSSNFLGVKLRS------KMSLRSYGSRE-GSV    103
Loxodonta.africana                  QGSIEDEAMASSGQIDLLERL-KELNLDSSNFPGVKLRS------KMSLRSYGSRE-GSV    103
Trichechus.manatus.latirostris      QGSIEDEAMASSGQIDLLERL-KELNLDSSNFPGVKLRS------KMSLRSYGSRE-GSV    103
Otolemur.garnettii                  QGSIEDEAMASSGQIDLLERL-KELNLDSSNFPGVKLRS------KMSLRSYGSRE-GSV    103
Callithrix.jacchus                  QGSIEDEAMASSGQIDLLERL-KELNLDSSNFPGVKLRS------KMSLRSYGSRE-GSV    103
Macaca.mulatta                      QGSIEDEAMASSGQIDLLERL-KELNLDSSNFPGVKLRS------KMSLRSYGSRE-GSV    103
Nomascus.leucogenys                 QGSIEDEAMASSGQIDLLERL-KELNLDSSNFPGVKLRS------KMSLRSYGSRE-GSV    103
Pongo.abelii                        QGSIEDEAMASSGQIDLLERL-KELNLDSSNFPGVKLRS------KMSLRSYGSRE-GSV    103
Homo.sapiens                        QGSIEDEAMASSGQIDLLERL-KELNLDSSNFPGVKLRS------KMSLRSYGSRE-GSV    103
Gorilla.gorilla                     QGSIEDEAMASSGQIDLLERL-KELNLDSSNFPGVKLRS------KMSLRSYGSRE-GSV    103
Pan.troglodytes                     QGSIEDEAMASSGQIDLLERL-KELNLDSSNFPGVKLRS------KMSLRSYGSRE-GSV    103
Pan.paniscus                        QGSIEDEAMASSGQIDLLERL-KELNLDSSNFPGVKLRS------KMSLRSYGSRE-GSV    103
Canis.lupus.familiaris              QGSIEDEAMASSGQIDLLERL-KELNLDSSNFPGVKLRS------KMSLRSYGSRE-GSV    103
```

```
Bos.taurus                          QGSIEDEAMASSGQIDLLERL-KELNLDSSNFPGVKLRS------KMSLRSYGSRE-GSV    103
Ovis.aries                          QGSIEDEAMASSGQIDLLERL-KELNLDSSNFPGVKLRS------KMSLRSYGSRE-GSV    103
Orcinus.orca                        QGSIEDEAMASSGQIDLLERL-KELNLDSSNFPGVKLRS------KMSLRSYGSRE-GSV    103
Sus.scrofa                          QGSIEDEAMASSGQIDLLERL-KELNLDSSNFPGVKLRS------KMSLRSYGSRE-GSV    103
Ceratotherium.simum.simum           QGSIEDEAMASSGQIDLLERL-KELNLDSSNFPGVKLRS------KMSLRSFGSRE-GSV    103
Equus.caballus                      QGSIEDEAMASSGQIDLLERL-KELNLDSSNFPGVKLRS------KMSLRSYGSRE-GSV    103
Felis.catus                         QGSIEDEAMASSGQIDLLERL-KELNLDSSNFPGVKLRS------KMSLRSYGSRE-GSV    103
Ailuropoda.melanoleuca              QGSIDNEAMASSGQTDLLERL-KELNLDSSNFPGVKLRS------KMSLRSYGSRE-GSV    103
Mustela.putorius.furo               QGSIEDEAMASSGQIDLLERL-KELNLDSSNFPGVKLRS------KMSLRSYESRE-GSV    103
Odobenus.rosmarus.divergens         QGSIEDEAMASSGQIDLLERL-KELNLDSSNFPGVKLRS------KMSLRSYGSRE-GSV    103
Taeniopygia.guttata                 QGSIEDETMASSGQIDLLERL-KELNLESTSFAGVKLRP------KMSVRSYGSRE-GSV    103
Anolis.carolinensis                 QGSIEDEAMASSGQIDLLERL-KELNLDSNNFPGVKLRP------KISMRSYGSRE-GSV    103
Gallus.gallus                       QGSIEDEAIASSGQIDLLERL-KELNLESTSFPGVKLRQ------KVSVRSYGSRE-GSV    103
                                     .                  : .  .

Ciona.intestinalis                  DVGH-----TPLSPSPSSTIA---VEIRRHLSNLHLQRTALLESLKREELAKDGYMRQLN    165
Strongylocentrotus.purpuratus       LPGKPHSLPTSLGTSGTDGSLRQTKDHFIRHQELERDRLLILNEQAKEENLRSQCLAQIQ    155
Danio.rerio                         ---DSSPSPSPVSSCPRRGASSGGRDSAGYLEELEKERSLLVAELEKEEKEKDWYYAQLQ    154
Xenopus.tropicalis                  SGHSGECSPVPVGSFQRRGLLNGSRESTGYLEELEKERLLLIAEHEKEEKEKRWYYAQLQ    163
Xenopus.laevis                      SGHSGECSPVPVGSFQRRGLLNGSRESAGYMEELEKERLLLIAEHEKEEKEKRWYYAQLQ    163
Ornithorhynchus.anatinus            SSRSGECSPVPMGSFPRRGFVNGSRESTGYLEELEKERSLLLAELEKEEKEKDWYYAQLQ    163
Monodelphis.domestica               SSRSGECSPVPMGSFSRRGFMNGSRESTGYLEELEKERSLLLAELEKEEKEKDWYYAQLQ    163
Mus.musculus                        SSRSGECSPVPMGSFPRRTFVNGSRESTGYLEELEKERSLLLADLDKEEKEKDWYYAQLQ    161
Rattus.norvegicus                   SSRSGECSPVPMGSFPRRAFVNGSRESTGYLEELEKERSLLLADLDKEEKEKDWYYAQLQ    161
Sorex.araneus                       SSRSGECGPLPSGSFPRRAVVNGSRENASYLEELEKERSLLLADLDKEEKEKDWYYAQLQ    163
Octodon.degus                       SSRSGECSPVPMGSFPRRGFVNGSRESTGYLEELEKERSLLLADLDKEEKEKDWYYAQLQ    163
Heterocephalus                      SSRSGECSPVPMGSFPRRGFVNGSRESTGYLEELEKERSLLLADLDKEEKEKDWYYAQLQ    163
Jaculus.jaculus                     SSRSGEGSPVPMGSFPRRVFVNGSRESTGYLEELEKERSLLLADLDKEEKEKDWYYAQLQ    163
Loxodonta.africana                  SSRSGECSPVPMGSFPRRTFVNGSRESTGYLEELEKERSLLLADLDKEEKEKDWYYAQLQ    163
Trichechus.manatus.latirostris      SSRSGECSPVPMGSFPRRGFVNGSRESTGYLEELEKERSLLLADLDKEEKEKDWYYAQLQ    163
Otolemur.garnettii                  SSRSGECSPVPMGSFPRRGFVNGSRESTGYLEELEKERSLLLADLDKEEKEKDWYYAQLQ    163
Callithrix.jacchus                  SSRSGECSPVPMGSFPRRGFVNGSRESTGYLEELEKERSLLLADLDKEEKEKDWYYAQLQ    163
Macaca.mulatta                      SSRSGECSPVPMGSFPRRGFVNGSRESTGYLEELEKERSLLLADLDKEEKEKDWYYAQLQ    163
Nomascus.leucogenys                 SSRSGECSPVPMGSFPRRGFVNGSRESTGYLEELEKERSLLLADLDKEEKEKDWYYAQLQ    163
Pongo.abelii                        SSRSGECSPVPMGSFPRRGFVNGSRESTGYLEELEKERSLLLADLDKEEKEKDWYYAQLQ    163
Homo.sapiens                        SSRSGECSPVPMGSFPRRGFVNGSRESTGYLEELEKERSLLLADLDKEEKEKDWYYAQLQ    163
Gorilla.gorilla                     SSRSGECSPVPMGSFPRRGFVNGSRESTGYLEELEKERSLLLADLDKEEKEKDWYYAQLQ    163
Pan.troglodytes                     SSRSGECSPVPMGSFPRRGFVNGSRESTGYLEELEKERSLLLADLDKEEKEKDWYYAQLQ    163
Pan.paniscus                        SSRSGECSPVPMGSFPRRGFVNGSRESTGYLEELEKERSLLLADLDKEEKEKDWYYAQLQ    163
Canis.lupus.familiaris              SSRSGECSPVPMGSFPRRGFVNGSRENTGYLEELEKERSLLLADLDKEEKEKDWYYAQLQ    163
Bos.taurus                          SSRSGECSPVPMGSFPRRGFVNGSRENTGYLEELEKERSLLLADLDKEEKEKDWYYAQLQ    163
Ovis.aries                          SSRSGECSPVPMGSFPRRGFVNGSRENTGYLEELEKERSLLLADLDKEEKEKDWYYAQLQ    163
Orcinus.orca                        SSRSGECSPVPMGSFPRRGFVNGSRENTGYLEELEKERSLLLADLDKEEKEKDWYYAQLQ    163
Sus.scrofa                          SSRSGECSPVPMGSFPRRGFVNGSRENTSYLEELEKERSLLLADLDKEEKEKDWYYAQLQ    163
Ceratotherium.simum.simum           SSRSGECSPVPMGSFPRRGFVNGSRENTGYLEELEKERSLLLADLDKEEKEKDWYYAQLQ    163
Equus.caballus                      SSRSGECSPVPMGSFPRRGFVNGSRENPGYLEELEKERSLLLADLDKEEKEKDWYYAQLQ    163
Felis.catus                         SSRSGECSPVPMGSFPKRGFVNGSRENTGYLEELEKERSLLLADLDKEEKEKDWYYAQLQ    163
Ailuropoda.melanoleuca              SSRSGECSPVPMGSFPRRGFVNGSRENTGYLEELEKERSLLLADLDKEEKEKDWYYAQLQ    163
Mustela.putorius.furo               SSRSGECSPVPMGSFPRRGFVNGSRENTGYLEELEKERSLLLADLDKEEKEKDWYYAQLQ    163
Odobenus.rosmarus.divergens         SSRSGECSPVPMGSFPRRGFVNGSRENTGYLEELEKERSLLLADLDKEEKEKDWYYAQLQ    163
Taeniopygia.guttata                 SSRSGECSPVPMGSFPRRGFMNGSRESTGYLEELEKERSLLLAELEKEEKEKDWYYAQLQ    163
Anolis.carolinensis                 SSRSGDCSPVPMGSYPRRGFMNGSRESTGYLEELEKERSLLLAELEKEEKEKDWYYAQLQ    163
Gallus.gallus                       SSRSGECSPVPMGSFPRRGFMNGSRESTGYLEELEKERSLLLAELEKEEKEKDWYYAQLQ    163
                                                .            :     .:*. :*  :: .  :**  :     *::

Ciona.intestinalis                  DLSEQLKIMHDTDKPFSPTSEKTRLQLEAGIREIQDQMEKNLGSPEMMSHRVQ-------    218
Strongylocentrotus.purpuratus       SLTRRIEDLPIT-DNYSLQTDMSRRQLEYEARQLRELMQERLGSMEDIAARHRMRMQRLS    214
Danio.rerio                         NLTKRIDSLPLT-ENFSLQTDMTRRQLEYEARQIRAAMEDQLGTCQDMEKRAQGRVARIQ    213
Xenopus.tropicalis                  NLTKRIDSLPLT-ENFSLQTDMTRRQLEYEARQIRAAMEEQLGTCQDMEKRVQTRVGKIQ    222
Xenopus.laevis                      NLTKRIDSLPLT-ENFSMQTDMTRRQLEYEARQIRAAMEEQLGTCQDMEKRVQTRVGKIH    222
Ornithorhynchus.anatinus            NLTKRIDSLPLT-ENFSLQTDMTRRQLEYEARQIRAAMEEQLGTCQDMEKRAQLRVARIQ    222
Monodelphis.domestica               NLTKRIDSLPLT-ENFSLQTDMTRRQLEYEARQIRAAMEEQLGTCQDMEKRAQLRVARIQ    222
Mus.musculus                        NLTKRIDSLPLT-ENFSLQTDMTRRQLEYEARQIRAAMEEQLGTCQDMEKRAQRRIARIQ    220
Rattus.norvegicus                   NLTKRIDSLPLT-ENFSLQTDMTRRQLEYEARQIRAAMEEQLGTCQDMEKRAQRRIARIQ    220
Sorex.araneus                       NLTKRIDSLPLT-ENFSLQTDMTRRQLEYEARQIRVAMEKQLGTCQDMEKRAQQRVTRIQ    222
```

```
Octodon.degus                       NLTKRIDSLPLT-ENFSLQTDMTRRQLEYEARQIRVAMEEQLGTCQDMEKRAQRRIARIQ    222
Heterocephalus                      NLTKRIDSLPLT-ENFSLQTDMTRRQLEYEARQIRVAMEEQLGTCQDMEKRAQRRIARIQ    222
Jaculus.jaculus                     NLTKRIDSLPLT-ENFSLQTDMTRRQLEYEARQIRVAMEEQLGTCQDMEKRAQRRITRIQ    222
Loxodonta.africana                  NLTKRIDSLPLT-ENFSLQTDMTRRQLEYEARQIRVAMEEQLGTCQDMEKRAQRRVTRIQ    222
Trichechus.manatus.latirostris      NLTKRIDSLPLT-ENFSLQTDMTRRQLEYEARQIRVAMEEQLGTCQDMEKRAQRRVARIQ    222
Otolemur.garnettii                  NLTKRIDSLPLT-ENFSLQTDMTRRQLEYEARQIRVAMEEQLGTCQDMEKRAQRRIARIQ    222
Callithrix.jacchus                  NLTKRIDSLPLT-ENFSLQTDMTRRQLEYEARQIRVAMEEQLGTCQDMEKRAQRRIARIQ    222
Macaca.mulatta                      NLTKRIDSLPLT-ENFSLQTDMTRRQLEYEARQIRVAMEEQLGTCQDMEKRAQRRIARIQ    222
Nomascus.leucogenys                 NLTKRIDSLPLT-ENFSLQTDMTRRQLEYEARQIRVAMEEQLGTCQDMEKRAQRRIARIQ    222
Pongo.abelii                        NLTKRIDSLPLT-ENFSLQTDMTRRQLEYEARQIRVAMEEQLGTCQDMEKRAQRRIARIQ    222
Homo.sapiens                        NLTKRIDSLPLT-ENFSLQTDMTRRQLEYEARQIRVAMEEQLGTCQDMEKRAQRRIARIQ    222
Gorilla.gorilla                     NLTKRIDSLPLT-ENFSLQTDMTRRQLEYEARQIRVAMEEQLGTCQDMEKRAQRRIARIQ    222
Pan.troglodytes                     NLTKRIDSLPLT-ENFSLQTDMTRRQLEYEARQIRVAMEEQLGTCQDMEKRAQRRIARIQ    222
Pan.paniscus                        NLTKRIDSLPLT-ENFSLQTDMTRRQLEYEARQIRVAMEEQLGTCQDMEKRAQRRIARIQ    222
Canis.lupus.familiaris              NLTKRIDSLPLT-ENFSLQTDMTRRQLEYEARQIRVAMEEQLGTCQDMEKRAQRRVTRIQ    222
Bos.taurus                          NLTKRIDSLPLT-ENFSLQTDMTRRQLEYEARQIRVAMEEQLGTCQDMEKRAQRRITRIQ    222
Ovis.aries                          NLTKRIDSLPLT-ENFSLQTDMTRRQLEYEARQIRVAMEEQLGTCQDMEKRAQRRITRIQ    222
Orcinus.orca                        NLTKRIDSLPLT-ENFSLQTDMTRRQLEYEARQIRVAMEEQLGTCQDMEKRAQRRITRIQ    222
Sus.scrofa                          NLTKRIDSLPLT-ENFSLQTDMTRRQLEYEARQIRVAMEEQLGTCQDMEKRAQRRITRIQ    222
Ceratotherium.simum.simum           NLTKRIDSLPLT-ENFSLQTDMTRRQLEYEARQIRVAMEEQLGTCQDMEKRAQRRITRIQ    222
Equus.caballus                      NLTKRIDSLPLT-ENFSLQTDMTRRQLEYEARQIRVAMEEQLGTCQDMEKRAQRRVTRIQ    222
Felis.catus                         NLTKRIDSLPLT-ENFSLQTDMTRRQLEYEARQIRVAMEEQLGTCQDMEKRAQRRVTRIQ    222
Ailuropoda.melanoleuca              NLTKRIDSLPLT-ENFSLQTDMTRRQLEYEARQIRVAMEEQLGTCQDMEKRAQRRVTRIQ    222
Mustela.putorius.furo               NLTKRIDSLPLT-ENFSLQTDMTRRQLEYEARQIRVAMEEQLGTCQDMEKRAQRRVTRIQ    222
Odobenus.rosmarus.divergens         NLTKRIDSLPLT-ENFSLQTDMTRRQLEYEARQIRVAMEEQLGTCQDMEKRAQRRVTRIQ    222
Taeniopygia.guttata                 NLTKRIDSLPLT-ENFSLQTDMTRRQLEYEARQIRAAMEEQLGTCQDMEKRAQARVARIQ    222
Anolis.carolinensis                 NLTKRIDSLPLT-ENFSLQTDMTRRQLEYEARQIRAAMEEQLGTCQDMEKRAQVRVSRIQ    222
Gallus.gallus                       NLTKRIDSLPLT-ENFSLQTDMTRRQLEYEARQIRAAMEEQLGTCQDMEKRAQVRVARIQ    222
                                    .*:.::. :  * . :*  :: :* ***   *:::  *:..**: : :  * :

Ciona.intestinalis                  ------------------------------------------------------------    218
Strongylocentrotus.purpuratus       AIEYELRQVQEQQQQHQQQQQ-------------QQQQQQQQQQQQQ----M--------    249
Danio.rerio                         QIEKDMLRIRTRLQAQSAESESSGK-RYRERVKHEPLSQTEGSHAAGDAGAAAAASVCSQ    272
Xenopus.tropicalis                  QIEEDILRIRQLLQSQAAEAAERTPQSKHDAGSRDADKLPDGQGTSEI--VTSSNVGSGQ    280
Xenopus.laevis                      QIEEEILRIRQLLQSQVAEAAERTPQSKHDAGSRDAEKLPDGQGTSEI--TASGNVGSGQ    280
Ornithorhynchus.anatinus            QIEKDILRIRQLLQSQAAE-AERTPQSKHDAGSHEAERQNEGQGTAEI--SM-ATAGSGQ    278
Monodelphis.domestica               QIEKDILRIRQLLQSQPAE-AERASQSKPDHASHEAERPNEGPGAADV--SV-APPAGSQ    278
Mus.musculus                        QIEKDILRVRQLLQSQAAE-AERSSQSRHDAASHEAGRQHEGHGVAES--NT-AASSSGQ    276
Rattus.norvegicus                   QIEKDILRVRQLLQSQAAE-AERSSQSKHETASHEAERQLEGQGVAES--NL-ATSGSGQ    276
Sorex.araneus                       QIEKDILRIRQLLQAQASE-TERPAQNRHENGSQEMERQSEGQGVAEV--ST-AASGSGQ    278
Octodon.degus                       QIEKDILRIRQLLQSQATE-AERSSQNKHEAGLHEAERQNEGQGVAEV--NI-ATSGS-Q    277
Heterocephalus                      QIEKDILRIRQLLQSQAAE-AERSSQNKHEAGLHEAERQNEGQGVAEI--NM-APSGS-Q    277
Jaculus.jaculus                     QIEKDILRIRQLLQSQATE-AERSSQSKHEASSQEAERQNEGQGVAEL--NM-ATSGSGQ    278
Loxodonta.africana                  QIEKDILRIRQLLQSQATE-AERSSQSKHEAGSHEAERQNEGQGVAEI--SM-ATSGSGQ    278
Trichechus.manatus.latirostris      QIEKDILRIRQLLQSQATE-TERSSQSKHEAGSHEAERQNEGQGVAEI--SM-ATSGSGQ    278
Otolemur.garnettii                  QIEKDILRIRQLLQSQATE-AERSSQNKHEASSHEAERQNEGQGVPEI--NM-ATSGSGQ    278
Callithrix.jacchus                  QIEKDILRIRQLLQSQATE-AERSSQNKHETGSHEAERQNEGQGVTEI--NM-ATSGNGQ    278
Macaca.mulatta                      QIEKDILRIRQLLQSQATE-AERSSQNKHETGSHDAERQNEGQGVAEI--NM-ATSGNGQ    278
Nomascus.leucogenys                 QIEKDILRIRQLLQSQATE-AERSSQSKHETGSHDAERQNEGQGVGEI--NM-ATSGNGQ    278
Pongo.abelii                        QIEKDILRIRQLLQSQATE-AERSSQNKHETGSHDAERQNEGQGVGEI--NM-ATSGNGQ    278
Homo.sapiens                        QIEKDILRIRQLLQSQATE-AERSSQNKHETGSHDAERQNEGQGVGEI--NM-ATSGNGQ    278
Gorilla.gorilla                     QIEKDILRIRQLLQSQATE-AERSSQNKHETGSHDAERQNEGQGVGEI--NM-ATSGNGQ    278
Pan.troglodytes                     QIEKDILRIRQLLQSQATE-AERSSQNKHETGSHDAERQNEGQGVGEI--NM-ATSGNGQ    278
Pan.paniscus                        QIEKDILRIRQLLQSQATE-AERSSQNKHETGSHDAERQNEGQGVGEI--NM-ATSGNGQ    278
Canis.lupus.familiaris              QIEKDILRIRQLLQSQATE-AERSSQSKHEAGSHEAERQNEGQGVGEI--NM-ATSGSAQ    278
Bos.taurus                          QIEKDILRIRQLLQSQATE-AERSSQSKHEAGSHEAERQNEGQGVAEI--NM-ATSGSGQ    278
Ovis.aries                          QIEKDILRIRQLLQSQATE-AERSSQSKHEAGSREAERQNEGQGAAET--SM-AASGSGQ    278
Orcinus.orca                        QIEKDILRIRQLLQSQATE-AERSSQSKHEAGSHEAERQNESQGVAEI--NM-ATSGSGQ    278
Sus.scrofa                          QIEKDILRIRQLLQSQATE-AERSSQSKHEAGSYEAERQNEGQGVAEI--SM-ATSGNGQ    278
Ceratotherium.simum.simum           QIEKDILRIRQLLQSQATE-AERSSQSKHEAGSHEAERQNEGQGVAEI--NM-ATSGSGQ    278
Equus.caballus                      QIEKDILRIRQLLQSQATE-AERSSQSKHEAGSHEAERQNEGQGVAEI--NM-ATSGSGQ    278
Felis.catus                         QIEKDILRIRQLLQSQATE-AERSSQSKHEAGSHEAERQNEGQGVAEI--NM-ATPGSGQ    278
Ailuropoda.melanoleuca              QIEKDILRIRQLLQPQATE-AERSSQSKHEAGSHEAERQNEGQGMADI--NM-AASGSGQ    278
Mustela.putorius.furo               QIEKDILRIRQLLQSQATE-AERSSQNKHEAGSHEAERQNEGQGVAEI--NM-ATSGSGQ    278
Odobenus.rosmarus.divergens         QIEKDILRIRQLLQSQATE-AERSSQSKHEAGSHEAERQNEGQGVAEI--NM-ATSGSGQ    278
```

```
Taeniopygia.guttata               QIEKDILRIRQLLQSQAAE-AERAPQSKHDAGSHDTERQNEGQGAAEV--NV-ATSSTGQ    278
Anolis.carolinensis               QIEKDILRIRQLLQSQVAE-TERAPPSKHDAGLHESERQNEGQGAAEI--SV-ATAGSSQ    278
Gallus.gallus                     QIEKDILRIRQLLQSQAAE-AERAPQGKHDAASHDTERQSEGQGAPEI--SM-STSNTGQ    278


Ciona.intestinalis                -----------------------------------------T---GEVCSLLGSHDRHDM    234
Strongylocentrotus.purpuratus     ------------------------------LA-KVWEQGAQVGFVYSLLSMLGSHDRDDM    278
Danio.rerio                       GSASRVDHDSASEMSSAGSYSVPRRLTSHLGTK--------VEMVYSLLSMLGTHDKDDM    324
Xenopus.tropicalis                GSSSRADHDTASVMSSNSTYSVPRRLTSHLGTK--------VEMVYSLLSMLGTHDKDDM    332
Xenopus.laevis                    GSSSRADHDTTSVMSSNSTYSVPRRLTSHLGTK--------VEMVYSLLSMLGTHDKDDM    332
Ornithorhynchus.anatinus          GSTARMDHETASVMSSSSTYSVPRRLTSHLGTK--------VEMVYSLLSMLGTHDKDDM    330
Monodelphis.domestica             GSVAQVDQETASGGSANGAYSVPRRLTSHLGTK--------VEMVYSLLSMLGTHDKDDM    330
Mus.musculus                      SPATRVDHETASVLSSSGTHSAPRRLTSHLGTK--------VEMVYSLLSMLGTHDKDDM    328
Rattus.norvegicus                 SSAARVDHETAGVLSSSGTHSAPRRLTSHLGTK--------VEMVYSLLSMLGTHDKDDM    328
Sorex.araneus                     GPAARVDPETASVLSSSSTHSAPRRLTSHLGTK--------VEMVYSLLSMLGTHDKDDM    330
Octodon.degus                     GSATRMDHETASILSSNSTHSAPRRLTSHLGTK--------VEMVYSLLSMLGTHDKDDM    329
Heterocephalus                    GSATRMDHETASVLSS-STHSAPRRLTSHLGTK--------VEMVYSLLSMLGTHDKDDM    328
Jaculus.jaculus                   SSTTRVDHETASVLSSSSTHSAPRRLTSHLGTK--------VEMVYSLLSMLGTHDKDDM    330
Loxodonta.africana                GSAARMDHETASVLSSSSTHSAPRRLTSHLGTK--------VEMVYSLLSMLGTHDKDDM    330
Trichechus.manatus.latirostris    GSAARMDHETASVLSSSSTHSAPRRLTSHLGTK--------VEMVYSLLSMLGTHDKDDM    330
Otolemur.garnettii                GSTTRMDHETASVLSSGSTHSAPRRLTSHLGTK--------VEMVYSLLSMLGTHDKDDM    330
Callithrix.jacchus                GSTTRMDHETASVLSSSSTHSAPRRLTSHLGTK--------VEMVYSLLSMLGTHDKDDM    330
Macaca.mulatta                    GSTTRMDHETASVLSSSSTHSAPRRLTSHLGTK--------VEMVYSLLSMLGTHDKDDM    330
Nomascus.leucogenys               GSATRVDHETASVLSSSSTHSAPRRLTSHLGTK--------VEMVYSLLSMLGTHDKDDM    330
Pongo.abelii                      GSTTRMDHETASVLSSSSTHSAPRRLTSHLGTK--------VEMVYSLLSMLGTHDKDDM    330
Homo.sapiens                      GSTTRMDHETASVLSSSSTHSAPRRLTSHLGTK--------VEMVYSLLSMLGTHDKDDM    330
Gorilla.gorilla                   GSTTRMDHETASVLSSSSTHSAPRRLTSHLGTK--------VEMVYSLLSMLGTHDKDDM    330
Pan.troglodytes                   GSTTRMDHETASVLSSSSTHSAPRRLTSHLGTK--------VEMVYSLLSMLGTHDKDDM    330
Pan.paniscus                      GSTTRMDHETASVLSSSSTHSAPRRLTSHLGTK--------VEMVYSLLSMLGTHDKDDM    330
Canis.lupus.familiaris            GSTARMDHETASVLSSSSTHSAPRRLTSHLGTK--------VEMVYSLLSMLGTHDKDDM    330
Bos.taurus                        GSTTRIDHETASVLSSSSTHSAPRRLTSHLGTK--------VEMVYSLLSMLGTHDKDDM    330
Ovis.aries                        GSTTRIDHETASVLSSSSTHSAPRRLTSHLGTK--------VEMVYSLLSMLGTHDKDDM    330
Orcinus.orca                      GSTARIDHETASVLSSSSTHSAPRRLTSHLGTK--------VEMVYSLLSMLGTHDKDDM    330
Sus.scrofa                        GSSTRVDHETASVLSSSSTHSAPRRLTSHLGTK--------VEMVYSLLSMLGTHDKDDM    330
Ceratotherium.simum.simum         GSAARMDHETASVLSSSSTHSAPRRLTSHLGTK--------VEMVYSLLSMLGTHDKDDM    330
Equus.caballus                    GSTARMDHETASVLSSSSTHSAPRRLTSHLGTK--------VEMVYSLLSMLGTHDKDDM    330
Felis.catus                       GSAARMDHETASVLSSSSTHSAPRRLTSHLGTK--------VEMVYSLLSMLGTHDKDDM    330
Ailuropoda.melanoleuca            GSTTRMDHETASVLSSGSTHSAPRRLTSHLGTK--------VEMVYSLLSMLGTHDKDDM    330
Mustela.putorius.furo             GSTTRMDHETASVLSSSSTHSAPRRLTSHLGTK--------VEMVYSLLSMLGTHDKDDM    330
Odobenus.rosmarus.divergens       GSTTRMDHETASVLSSSSTHSAPRRLTSHLGTK--------VEMVYSLLSMLGTHDKDDM    330
Taeniopygia.guttata               GSAARVDHETASVMSSSSNYSVPRRLTSHLGTK--------VEMVYSLLSMLGTHDKDDM    330
Anolis.carolinensis               SSTARMDHETSSVMSSNSSYSVPRRLTSHLGTK--------VEMVYSLLSMLGTHDKDDM    330
Gallus.gallus                     GSAARMDHETASVMSSSNNYSVPRRLTSHLGTKVTEDYKPQVEMVYSLLSMLGTHDKDDM    338
                                                                           .    .: *:**:**:.**

Ciona.intestinalis                SCTFQRLSQSEDSCIVMRQDGYLPSMLRLLHDGS---ILPGGANPTPEEVKLRLEIRSKT    291
Strongylocentrotus.purpuratus     ASTLLMMSRSADSCLAMRQSGCIPLLIHILHGTDQESV-LGNF-------RGSQKARDCA    330
Danio.rerio                       SRTLLAMSSSQDSCIAMRQSGCLPLLIQLLHGNDKDSVLLGNS-------RGSKEARARA    377
Xenopus.tropicalis                SRTLLAMSSSQDSCIAMRQSGCLPLLIQLLHGNDKDSVLLGNS-------RGSKEARARA    385
Xenopus.laevis                    SRTLLAMSSSQDSCIAMRQSGCLPLLIQLLHGNDKDSVLLGNS-------RGSKEARASG    385
Ornithorhynchus.anatinus          SRTLLAMSSSQDSCISMRQSGCLPLLIQLLHGNDKDSVLLGNS-------RGSKEARARA    383
Monodelphis.domestica             SRTLLAMSSSQDSCISMRQSGCLPLLIQLLHGNDKDSVLLGNS-------RGSKEARARA    383
Mus.musculus                      SRTLLAMSSSQDSCISMRQSGCLPLLIQLLHGNDKDSVLLGNS-------RGSKEARARA    381
Rattus.norvegicus                 SRTLLAMSSSQDSCISMRQSGCLPLLIQLLHGNDKDSVLLGNS-------RGSKEARARA    381
Sorex.araneus                     SRTLLAMSSSQDSCISMRQSGCLPLLIQLLHGNDKDSVLLGSA-------RGSKEARARA    383
Octodon.degus                     SRTLLAMSSSQDSCISMRQSGCLPLLIQLLHGNDKDSVLLGNS-------RGSKEARARA    382
Heterocephalus                    SRTLLAMSSSQDSCISMRQSGCLPLLIQLLHGNDKDSVLLGNS-------RGSKEARARA    381
Jaculus.jaculus                   SRTLLAMSSSQDSCISMRQSGCLPLLIQLLHGNDKDSVLLGNS-------RGSKEARARA    383
Loxodonta.africana                SRTLLAMSSSQDSCISMRQSGCLPLLIQLLHGNDKDSVLLGNS-------RGSKEARARA    383
Trichechus.manatus.latirostris    SRTLLAMSSSQDSCISMRQSGCLPLLIQLLHGNDKDSVLLGNS-------RGSKEARARA    383
Otolemur.garnettii                SRTLLAMSSSQDSCISMRQSGCLPLLIQLLHGNDKDSVLLGNS-------RGSKEARARA    383
Callithrix.jacchus                SRTLLAMSSSQDSCISMRQSGCLPLLIQLLHGNDKDSVLLGNS-------RGSKEARARA    383
Macaca.mulatta                    SRTLLAMSSSQDSCISMRQSGCLPLLIQLLHGNDKDSVLLGNS-------RGSKEARARA    383
Nomascus.leucogenys               SRTLLAMSSSQDSCISMRQSGCLPLLIQLLHGNDKDSVLLGNS-------RGSKEARARA    383
Pongo.abelii                      SRTLLAMSSSQDSCISMRQSGCLPLLIQLLHGNDKDSVLLGNS-------RGSKEARARA    383
```

```
Homo.sapiens                        SRTLLAMSSSQDSCISMRQSGCLPLLIQLLHGNDKDSVLLGNS-------RGSKEARARA    383
Gorilla.gorilla                     SRTLLAMSSSQDSCISMRQSGCLPLLIQLLHGNDKDSVLLGNS-------RGSKEARARA    383
Pan.troglodytes                     SRTLLAMSSSQDSCISMRQSGCLPLLIQLLHGNDKDSVLLGNS-------RGSKEARARA    383
Pan.paniscus                        SRTLLAMSSSQDSCISMRQSGCLPLLIQLLHGNDKDSVLLGNS-------RGSKEARARA    383
Canis.lupus.familiaris              SRTLLAMSSSQDSCISMRQSGCLPLLIQLLHGNDKDSVLLGNS-------RGSKEARARA    383
Bos.taurus                          SRTLLAMSSSQDSCISMRQSGCLPLLIQLLHGNDKDSVLLGNS-------RGSKEARARA    383
Ovis.aries                          SRTLLAMSSSQDSCISMRQSGCLPLLIQLLHGNDKDSVLLGNS-------RGSKEARARA    383
Orcinus.orca                        SRTLLAMSSSQDSCISMRQSGCLPLLIQLLHGNDKDSVLLGNS-------RGSKEARARA    383
Sus.scrofa                          SRTLLAMSSSQDSCISMRQSGCLPLLIQLLHGNDKDSVLLGNS-------RGSKEARARA    383
Ceratotherium.simum.simum           SRTLLAMSSSQDSCISMRQSGCLPLLIQLLHGNDKDSVLLGNS-------RGSKEARARA    383
Equus.caballus                      SRTLLAMSSSQDSCISMRQSGCLPLLIQLLHGNDKDSVLLGNS-------RGSKEARARA    383
Felis.catus                         SRTLLAMSSSQDSCISMRQSGCLPLLIQLLHGNDKDSVLLGNS-------RGSKEARARA    383
Ailuropoda.melanoleuca              SRTLLAMSSSQDSCISMRQSGCLPLLIQLLHGNDKDSVLLGNS-------RGSKEARARA    383
Mustela.putorius.furo               SRTLLAMSSSQDSCISMRQSGCLPLLIQLLHGNDKDSVLLGNS-------RGSKEARARA    383
Odobenus.rosmarus.divergens         SRTLLAMSSSQDSCISMRQSGCLPLLIQLLHGNDKDSVLLGNS-------RGSKEARARA    383
Taeniopygia.guttata                 SRTLLAMSSSQDSCIAMRQSGCLPLLIQLLHGNDKDSVLLGNS-------RGSKEARARA    383
Anolis.carolinensis                 SRTLLAMSSSQDSCIAMRQSGCLPLLIQLLHGNDKDSVLLGNS-------RGSKEARARA    383
Gallus.gallus                       SRTLLAMSSSQDSCIAMRQSGCLPLLIQLLHGNDKDSVLLGNS-------RGSKEARARA    391
                                    : *:  :* * ***: ***.* :* ::::**. .   :  *.        :   : *

Ciona.intestinalis                  SQALTNIVSSNTDGDQKRIESHVLRHLEMIRRVSEDLFESLVEKNHTGASSESAESPTRH    351
Strongylocentrotus.purpuratus       STALHNIVHLNPDEKRRKQEGRVLRLLEQIRTYCDSLVEAETKES--------ASSQNAL    382
Danio.rerio                         SAALHNIIHSQPDDKRGRREIRVLHLLEQIRAYCETCWEWQESHE-RGV--DQDKNPMPS    434
Xenopus.tropicalis                  SAALHNIIHSQPDDKRGRREIRVLHLLEQIRAYCETCWEWQEAHE-QGM--DQDKNPMPA    442
Xenopus.laevis                      SAALDNIIHSQPDDKRGRREIRVLHLLEQIRAYCETCWEWQEAHE-QGM--DQDKNPMPA    442
Ornithorhynchus.anatinus            SAALHNIIHSQPDDKRGRREIRVLHLLEQIRAYCETCWEWQEAHE-QGM--DQDKNPMPA    440
Monodelphis.domestica               SAALHNIIHSQPDDKRGRREIRVLHLLEQIRAYCETCWQWQEAHE-QGM--DQDKNPMPA    440
Mus.musculus                        SAALHNIIHSQPDDKRGRREIRVLHLLEQIRAYCETCWEWQEAHE-QGM--DQDKNPMPA    438
Rattus.norvegicus                   SAALHNIIHSQPDDKRGRREIRVLHLLEQIRAYCETCWEWQEAHE-QGM--DQDKNPMPA    438
Sorex.araneus                       SAALHNIIHSQPDDKRGRREIRVLHLLEQIRAYCETCWEWQEAHE-QGM--DQDKNPMPV    440
Octodon.degus                       SAALHNIIHSQPDDKRGRREIRVLHLLEQIRAYCETCWEWQEAHE-QGM--DQDKNPMPA    439
Heterocephalus                      SAALHNIIHSQPDDKRGRREIRVLHLLEQIRAYCETCWEWQEAHE-QGM--DQDKNPMPA    438
Jaculus.jaculus                     SAALHNIIHSQPDDKRGRREIRVLHLLEQIRAYCETCWEWQEAHE-QGM--DQDKNPMPA    440
Loxodonta.africana                  SAALHNIIHSQPDDKRGRREIRVLHLLEQIRAYCETCWEWQEAHE-QGM--DQDKNPMPA    440
Trichechus.manatus.latirostris      SAALHNIIHSQPDDKRGRREIRVLHLLEQIRAYCETCWEWQEAHE-QGM--DQDKNPMPA    440
Otolemur.garnettii                  SAALHNIIHSQPDDKRGRREIRVLHLLEQIRAYCETCWEWQEAHE-QGM--DQDKNPMPA    440
Callithrix.jacchus                  SAALHNIIHSQPDDKRGRREIRVLHLLEQIRAYCETCWEWQEAHE-QGM--DQDKNPMPA    440
Macaca.mulatta                      SAALHNIIHSQPDDKRGRREIRVLHLLEQIRAYCETCWEWQEAHE-QGM--DQDKNPMPA    440
Nomascus.leucogenys                 SAALHNIIHSQPDDKRGRREIRVLHLLEQIRAYCETCWEWQEAHE-QGM--DQDKNPMPA    440
Pongo.abelii                        SAALHNIIHSQPDDKRGRREIRVLHLLEQIRAYCETCWEWQEAHE-QGM--DQDKNPMPA    440
Homo.sapiens                        SAALHNIIHSQPDDKRGRREIRVLHLLEQIRAYCETCWEWQEAHE-PGM--DQDKNPMPA    440
Gorilla.gorilla                     SAALHNIIHSQPDDKRGRREIRVLHLLEQIRAYCETCWEWQEAHE-QGM--DQDKNPMPA    440
Pan.troglodytes                     SAALHNIIHSQPDDKRGRREIRVLHLLEQIRAYCETCWEWQEAHE-QGM--DQDKNPMPA    440
Pan.paniscus                        SAALHNIIHSQPDDKRGRREIRVLHLLEQIRAYCETCWEWQEAHE-QGM--DQDKNPMPA    440
Canis.lupus.familiaris              SAALHNIIHSQPDDKRGRREIRVLHLLEQIRAYCETCWEWQEAHE-QGM--DQDKNPMPA    440
Bos.taurus                          SAALHNIIHSQPDDKRGRREIRVLHLLEQIRAYCETCWEWQEAHE-QGM--DQDKNPMPA    440
Ovis.aries                          SAALHSIVHSQPDDKRGRREIRVLHLLEQIRAYCEACWEWQEAHE-QGL--DQDRNPMPA    440
Orcinus.orca                        SAALHNIIHSQPDDKRGRREIRVLHLLEQIRAYCETCWEWQEAHE-QGM--DQDKNPMPA    440
Sus.scrofa                          SAALHNIIHSQPDDKRGRREIRVLHLLEQIRAYCETCWEWQEAHE-QGM--DQDKNPMPA    440
Ceratotherium.simum.simum           SAALHNIIHSQPDDKRGRREIRVLHLLEQIRAYCETCWEWQEAHE-QGM--DQDKNPMPA    440
Equus.caballus                      SAALHNIIHSQPDDKRGRREIRVLHLLEQIRAYCETCWEWQEAHE-QGM--DQDKNPMPA    440
Felis.catus                         SAALHNIIHSQPDDKRGRREIRVLHLLEQIRAYCETCWEWQEAHE-QGM--DQDKNPMPA    440
Ailuropoda.melanoleuca              SAALHNIIHSQPDDKRGRREIRVLHLLEQIRAYCETCWEWQEAHE-QGM--DQDKNPMPA    440
Mustela.putorius.furo               SAALHNIIHSQPDDKRGRREIRVLHLLEQIRAYCETCWEWQEAHE-QGM--DQDKNPMPA    440
Odobenus.rosmarus.divergens         SAALHNIIHSQPDDKRGRREIRVLHLLEQIRAYCETCWEWQEAHE-QGM--DQDKNPMPA    440
Taeniopygia.guttata                 SAALHNIIHSQPDDKRGRREIRVLHLLEQIRAYCETCWEWQEAHE-QGM--DQDKNPMPA    440
Anolis.carolinensis                 SAALHNIIHSQPDDKRGRREIRVLHLLEQIRAYCETCWEWQEAHD-QGM--DQDKNPMPA    440
Gallus.gallus                       SAALHNIIHSQPDDKRGRREIRVLHLLEQIRAYCETCWEWQEAHE-QGM--DQDKNPMPA    448
                                    * ** .*:  : * .: : * :**: ** **  .:   :    ..          .

Ciona.intestinalis                  PVAETLKNAESIIHSLREIMELVNYKVQRLAINELGGLFCVAEILILHCSSKHDEEAQEE    411
Strongylocentrotus.purpuratus       PIDH------NPGPAMAALMKLSFDEEHRSAICHLGGLHAIAELLQVDYEVHGS--SNDQ    434
Danio.rerio                         PVEH------QICPAVCVLMKLSFDEEHRHAMNELGGLQAIGELLQVDCEIYGL--TNDH    486
Xenopus.tropicalis                  PVDH------QICPAVCVLMKLSFDEEHRHAMNELGGLQAIAELLQVDCEMYGL--INDH    494
Xenopus.laevis                      PVDH------QICPAVCVLMKLSFDEEHRHAMNELGGLQAIAELLQVDCEMYGL--INDH    494
```

```
Ornithorhynchus.anatinus           PVEH------QICPAVCVLMKLSFDEEHRHAMNELGGLQAIAELLQVDCEMYGL--THDH    492
Monodelphis.domestica              PVEH------QICPAVCVLMKLSFDEEHRHAMNELGGLQAIAELLQVDCEMYGL--TSDH    492
Mus.musculus                       PVEH------QICPAVCVLMKLSFDEEHRHAMNELGGLQAIAELLQVDCEMYGL--TNDH    490
Rattus.norvegicus                  PVEH------QICPAVCVLMKLSFDEEHRHAMNELGGLQAIAELLQVDCEMHGL--TDDH    490
Sorex.araneus                      PVEH------QICPAVCVLMKLSFDEEHRHAMNELGGLQAIAELLQVDCEMYGL--TSDH    492
Octodon.degus                      PVEH------QICPAVCVLMKLSFDEEHRHAMNELGGLQAIAELLQVDCEMYGL--TNDH    491
Heterocephalus                     PVEH------QICPAVCVLMKLSFDEEHRHAMNELGGLQAIAELLQVDCEMYGL--TNDH    490
Jaculus.jaculus                    PVEH------QICPAVCVLMKLSFDEEHRHAMNELGGLQAIAELLQVDCEMYGL--TNDH    492
Loxodonta.africana                 PVEH------QICPAVCVLMKLSFDEEHRHAMNELGGLQAIAELLQVDCEMYGL--TNDH    492
Trichechus.manatus.latirostris     PVEH------QICPAVCVLMKLSFDEEHRHAMNELGGLQAIAELLQVDCEMYGL--TNDH    492
Otolemur.garnettii                 PVEH------QICPAVCVLMKLSFDEEHRHAMNELGGLQAIAELLQVDCEMYGL--TNDH    492
Callithrix.jacchus                 PVEH------QICPAVCVLMKLSFDEEHRHAMNELGGLQAIAELLQVDCEMYGL--TNDH    492
Macaca.mulatta                     PVEH------QICPAVCVLMKLSFDEEHRHAMNELGGLQAIAELLQVDCEMYGL--TNDH    492
Nomascus.leucogenys                PVEH------QICPAVCVLMKLSFDEEHRHAMNELGGLQAIAELLQVDCEMYGL--TNDH    492
Pongo.abelii                       PVEH------QICPAVCVLMKLSFDEEHRHAMNELGGLQAIAELLQVDCEMYGL--TNDH    492
Homo.sapiens                       PVEH------QICPAVCVLMKLSFDEEHRHAMNELGGLQAIAELLQVDCEMYGL--TNDH    492
Gorilla.gorilla                    PVEH------QICPAVCVLMKLSFDEEHRHAMNELGGLQAIAELLQVDCEMYGL--TNDH    492
Pan.troglodytes                    PVEH------QICPAVCVLMKLSFDEEHRHAMNELGGLQAIAELLQVDCEMYGL--TNDH    492
Pan.paniscus                       PVEH------QICPAVCVLMKLSFDEEHRHAMNELGGLQAIAELLQVDCEMYGL--TNDH    492
Canis.lupus.familiaris             PVEH------QICPAVCVLMKLSFDEEHRHAMNELGGLQAIAELLQVDCEMYGL--TNDH    492
Bos.taurus                         PVEH------QICPAVCVLMKLSFDEEHRHAMNELGGLQAIAELLQVDCEMYGL--TNDH    492
Ovis.aries                         PVEH------QICPAVCVLMKLSFDEEHRHAMNELGGLQAIAELLRVDCEMHGL--TKDH    492
Orcinus.orca                       PVEH------QICPAVCVLMKLSFDEEHRHAMNELGGLQAIAELLQVDCEMYGL--TNDH    492
Sus.scrofa                         PVEH------QICPAVCVLMKLSFDEEHRHAMNELGGLQAIAELLQVDCEMYGL--TNDH    492
Ceratotherium.simum.simum          PVEH------QICPAVCVLMKLSFDEEHRHAMNELGGLQAIAELLQVDCEMYGL--TNDH    492
Equus.caballus                     PVEH------QICPAVCVLMKLSFDEEHRHAMNELGGLQAIAELLQVDCEMYGL--TNDH    492
Felis.catus                        PVEH------QICPAVCVLMKLSFDEEHRHAMNELGGLQAIAELLQVDCEMYGL--TNDH    492
Ailuropoda.melanoleuca             PVEH------QICPAVCVLMKLSFDEEHRHAMNELGGLQAIAELLQVDCEMYGL--TNDH    492
Mustela.putorius.furo              PVEH------QICPAVCVLMKLSFDEEHRHAMNELGGLQAIAELLQVDCEMYGL--TNDH    492
Odobenus.rosmarus.divergens        PVEH------QICPAVCVLMKLSFDEEHRHAMNELGGLQAIAELLQVDCEMYGL--TNDH    492
Taeniopygia.guttata                PVDH------QICPAVCVLMKLSFDEEHRHAMNELGGLQAIAELLQVDCEMYGL--TNDH    492
Anolis.carolinensis                PVDH------QICPAVCVLMKLSFDEEHRHAMNELGGLQAIAELLQVDCEMYGL--TNDH    492
Gallus.gallus                      PVDH------QICPAVCVLMKLSFDEEHRHAMNELGGLQAIAELLQVDCEMYGL--TNDH    500
                                   *: .      .   ::  :*:*   : :* *: .**** .:.*:* :. .        :.

Ciona.intestinalis                 TGSRLRQYSGRILTNLTYADNLNKVLLMNMRGLLETVRDQLQHESEEIQQAMASILRNLS    471
Strongylocentrotus.purpuratus      YTVTLRRYAGMALTNLTFGDVTNKALLCSMKGCMKALVALLSAESEDLRQVAASVLRNLS    494
Danio.rerio                        YSVTLRRYAGMALTNLTFGDVANKATLCSMKGCMRAMVAQLKSESEDLQQVIASVLRNLS    546
Xenopus.tropicalis                 YSVTLRRYAGMALTNLTFGDVANKATLCSMKSCMRALVAQLKSESEDLQQVIASVLRNLS    554
Xenopus.laevis                     YSVTLRRYAGMALTNLTFGDVANKATLCSMKSCMRALVAQLKSESEDLQQVIASVLRNLS    554
Ornithorhynchus.anatinus           YSVTLRRYAGMALTNLTFGDVANKATLCSMKGCMRALVAQLKSESEDLQQVIASVLRNLS    552
Monodelphis.domestica              YSVTLRRYAGMALTNLTFGDVANKATLCSMKGCMRALVAQLKSESEDLEQVIASVLRNLS    552
Mus.musculus                       YSVTLRRYAGMALTNLTFGDVANKATLCSMKGCMRALVAQLKSESEDLQQVIASVLRNLS    550
Rattus.norvegicus                  YSVTLRRYAGMALTNLTFGDVANKATLCSMKGCMRALVAQLKSESEDLQQVIASVLRNLS    550
Sorex.araneus                      YSITLRRYAGMALTNLTFGDVANKATLCSMKGCMRALVAQLKSDSEDLQQVIASVLRNLS    552
Octodon.degus                      YSITLRRYAGMALTNLTFGDVANKATLCSMKGCMRALVAQLKSESEDLQQVIASVLRNLS    551
Heterocephalus                     YSITLRRYAGMALTNLTFGDVANKATLCSMKGCMRALVAQLKSESEDLQQVIASVLRNLS    550
Jaculus.jaculus                    YSITLRRYAGMALTNLTFGDVANKATLCSMKGCMRALVAQLKSESEDLQQVIASVLRNLS    552
Loxodonta.africana                 YSVTLRRYAGMALTNLTFGDVANKATLCSMKGCMRALVAQLKSESEDLQQVIASVLRNLS    552
Trichechus.manatus.latirostris     YSITLRRYAGMALTNLTFGDVANKATLCSMKGCMRALVAQLKSESEDLQQVIASVLRNLS    552
Otolemur.garnettii                 YSITLRRYAGMALTNLTFGDVANKATLCSMKGCMRALVAQLKSESEDLQQVIASVLRNLS    552
Callithrix.jacchus                 YSITLRRYAGMALTNLTFGDVANKATLCSMKGCMRALVAQLKSESEDLQQVIASVLRNLS    552
Macaca.mulatta                     YSITLRRYAGMALTNLTFGDVANKATLCSMKGCMRALVAQLKSESEDLQQVIASVLRNLS    552
Nomascus.leucogenys                YSITLRRYAGMALTNLTFGDVANKATLCSMKGCMRALVAQLKSESEDLQQVIASVLRNLS    552
Pongo.abelii                       YSITLRRYAGMALTNLTFGDVANKATLCSMKGCMRALVAQLKSESEDLQQVIASVLRNLS    552
Homo.sapiens                       YSITLRRYAGMALTNLTFGDVANKATLCSMKGCMRALVAQLKSESEDLQQVIASVLRNLS    552
Gorilla.gorilla                    YSITLRRYAGMALTNLTFGDVANKATLCSMKGCMRALVAQLKSESEDLQQVIASVLRNLS    552
Pan.troglodytes                    YSITLRRYAGMALTNLTFGDVANKATLCSMKGCMRALVAQLKSESEDLQQVIASVLRNLS    552
Pan.paniscus                       YSITLRRYAGMALTNLTFGDVANKATLCSMKGCMRALVAQLKSESEDLQQVIASVLRNLS    552
Canis.lupus.familiaris             YSITLRRYAGMALTNLTFGDVANKATLCSMKGCMRALVAQLKSESEDLQQVIASVLRNLS    552
Bos.taurus                         YSITLRRYAGMALTNLTFGDVANKATLCSMKGCMRALVAQLQSESEDLQQVIASVLRNLS    552
Ovis.aries                         YSITLRRYAGMALTNLTFGDVANKATLCSMKGCMRALVAQLQSESEDLQQVIASVLRNLS    552
Orcinus.orca                       YSITLRRYAGMALTNLTFGDVANKATLCSMKGCMRALVAQLKSESEDLQQVIASVLRNLS    552
Sus.scrofa                         YSITLRRYAGMALTNLTFGDVANKATLCSMKGCMRALVAQLKSESEDLQQVIASVLRNLS    552
Ceratotherium.simum.simum          YSITLRRYAGMALTNLTFGDVANKATLCSMKGCMRALVAQLKSESEDLQQVIASVLRNLS    552
```

```
Equus.caballus                      YSITLRRYAGMALTNLTFGDVANKATLCSMKGCMRALVAQLKSESEDLQQVIASVLRNLS    552
Felis.catus                         YSITLRRYAGMALTNLTFGDVANKATLCSMKGCMRALVAQLKSESEDLQQVIASVLRNLS    552
Ailuropoda.melanoleuca              YSITLRRYAGMALTNLTFGDVANKATLCSMKGCMRALVAQLKSESEDLQQVIASVLRNLS    552
Mustela.putorius.furo               YSITLRRYAGMALTNLTFGDVANKATLCSMKGCMRALVAQLKSESEDLQQVIASVLRNLS    552
Odobenus.rosmarus.divergens         YSITLRRYAGMALTNLTFGDVANKATLCSMKGCMRALVAQLKSESEDLQQVIASVLRNLS    552
Taeniopygia.guttata                 YSVTLRRYAGMALTNLTFGDVANKATLCSMKGCMRALVAQLKSESEDLQQVIASVLRNLS    552
Anolis.carolinensis                 YSVTLRRYAGMALTNLTFGDVANKATLCSMKGCMRALVAQLKSESEDLQQVIASVLRNLS    552
Gallus.gallus                       YSVTLRRYAGMALTNLTFGDVANKATLCSMKGCMRALVAQLKSESEDLQQVIASVLRNLS    560
                                        **:*:*  *****:.*  **. * .*:. :.::   *. :**::.*. **:*****

Ciona.intestinalis                  WQADKEGRDLLRQKGVVRTLTECVICAKGEGTLKAMLSALWNLSGHCPANREDICSVEGS    531
Strongylocentrotus.purpuratus       WRADMASKKALREAGAVVALMTCSLEVKKESTLKSVLSALWNLSAHCTENKADICAVNGA    554
Danio.rerio                         WRADVNSKKILREVGSVRALMECALEVQKESTLKSVLSALWNLSAHCTENKADICTVPGA    606
Xenopus.tropicalis                  WRADVNSKKTLREVGSVKALMECALDVKKESTLKSVLSALWNLSAHCTENKADICSVDGA    614
Xenopus.laevis                      WRADVNSKKTLREVGSVKALMECALDVKKESTLKSVLSALWNLSAHCTENKADICSVDGA    614
Ornithorhynchus.anatinus            WRADVNSKKTLREVGSVKALMECALEVKKESTLKSVLSALWNLSAHCTENKAEICAVDGA    612
Monodelphis.domestica               WRADVNSKKTLREVGSVKALMECALEVKKESTLKSVLSALWNLSAHCTENKADICAVDGA    612
Mus.musculus                        WRADVNSKKTLREVGSVKALMECALEVKKESTLKSVLSALWNLSAHCTENKADICAVDGA    610
Rattus.norvegicus                   WRADVNSKKTLREVGSVKALMECALEVKKESTLKSVLSALWNLSAHCTENKADICAVDGA    610
Sorex.araneus                       WRADVNSKKTLREVGSVRALMECALEVKKESTLKSVLSALWNLSAHCTENKADICAVDGA    612
Octodon.degus                       WRADVNSKKTLREVGSVKALMECALEVKKESTLKSVLSALWNLSAHCTENKADICAVDGA    611
Heterocephalus                      WRADVNSKKTLREVGSVKALMECALEVKKESTLKSVLSALWNLSAHCTENKADICAVDGA    610
Jaculus.jaculus                     WRADVNSKKTLREVGSVKALMECALEVKKESTLKSVLSALWNLSAHCTENKADICAVDGA    612
Loxodonta.africana                  WRADVNSKKTLREVGSVKALMECALEVKKESTLKSVLSALWNLSAHCTENKADICAVDGA    612
Trichechus.manatus.latirostris      WRADVNSKKTLREVGSVKALMECALEVKKESTLKSVLSALWNLSAHCTENKADICAVDGA    612
Otolemur.garnettii                  WRADVNSKKTLREVGSVRALMECALEVKKESTLKSVLSALWNLSAHCTENKADICAVDGA    612
Callithrix.jacchus                  WRADVNSKKTLREVGSVKALMECALEVKKESTLKSVLSALWNLSAHCTENKADICAVDGA    612
Macaca.mulatta                      WRADVNSKKTLREVGSVKALMECALEVKKESTLKSVLSALWNLSAHCTENKADICAVDGA    612
Nomascus.leucogenys                 WRADVNSKKTLREVGSVKALMECALEVKKESTLKSVLSALWNLSAHCTENKADICAVDGA    612
Pongo.abelii                        WRADVNSKKTLREVGSVKALMECALEVKKESTLKSVLSALWNLSAHCTENKADICAVDGA    612
Homo.sapiens                        WRADVNSKKTLREVGSVKALMECALEVKKESTLKSVLSALWNLSAHCTENKADICAVDGA    612
Gorilla.gorilla                     WRADVNSKKTLREVGSVKALMECALEVKKESTLKSVLSALWNLSAHCTENKADICAVDGA    612
Pan.troglodytes                     WRADVNSKKTLREVGSVKALMECALEVKKESTLKSVLSALWNLSAHCTENKADICAVDGA    612
Pan.paniscus                        WRADVNSKKTLREVGSVKALMECALEVKKESTLKSVLSALWNLSAHCTENKADICAVDGA    612
Canis.lupus.familiaris              WRADVNSKKTLREVGSVKAXXKCAXKLKPESTLKSVLSALWNLSAHCTENKADICAVDGA    612
Bos.taurus                          WRADVNSKKTLREVGSVKALMECALEVKKESTLKSVLSALWNLSAHCTENKADICAVDGA    612
Ovis.aries                          WRADVNSKKTLREVGSVKALMECALEVKKESTLKSVLSALWNLSAHCTENKADICAVDGA    612
Orcinus.orca                        WRADVNSKKTLREVGSVKALMECALEVKKESTLKSVLSALWNLSAHCTENKADICAVDGA    612
Sus.scrofa                          WRADVNSKKTLREVGSVKALMECALEVKKESTLKSVLSALWNLSAHCTENKADICAVDGA    612
Ceratotherium.simum.simum           WRADVNSKKTLREVGSVKALMECALEVKKESTLKSVLSALWNLSAHCTENKADICAVDGA    612
Equus.caballus                      WRADVNSKKTLREVGSVKALMECALEVKKESTLKSVLSALWNLSAHCTENKADICAVDGA    612
Felis.catus                         WRADVNSKKTLREVGSVKALMECALEVKKESTLKSVLSALWNLSAHCTENKADICAVDGA    612
Ailuropoda.melanoleuca              WRADVNSKKTLREVGSVKALMECALEVKKESTLKSVLSALWNLSAHCTENKADICAVDGA    612
Mustela.putorius.furo               WRADVNSKKTLREVGSVKALMECALEVKKESTLKSVLSALWNLSAHCTENKADICAVDGA    612
Odobenus.rosmarus.divergens         WRADVNSKKTLREVGSVKALMECALEVKKESTLKSVLSALWNLSAHCTENKADICAVDGA    612
Taeniopygia.guttata                 WRADVNSKKTLREVGSVKALMECALEVKKESTLKSVLSALWNLSAHCTGNKADICAVDGA    612
Anolis.carolinensis                 WRADVNSKKTLREVGSVKALMECALEVKKESTLKSVLSALWNLSAHCTENKADICAVDGA    612
Gallus.gallus                       WRADVNSKKTLREVGSVKALMECALEVKKESTLKSVLSALWNLSAHCTENKADICAVDGA    620
                                    *:**  .:. **: * * :   *    : *.***::********.**  *: :**:* *:

Ciona.intestinalis                  LAFLVSTLTYRSPSKSAMVIESGGGILRNISSIVATNETFRQILREHNCLQTLLQQLTSA    591
Strongylocentrotus.purpuratus       LEFLVSSLTYRSPTRNSAVVENGGGILRNVSSHIATSEKYRQLLRKHNCLQILLHHLKSS    614
Danio.rerio                         LAFLVSTLTYRSQTNTLAIIESGGGILRNVSSLIATNEEHRQILRENSCLQTLLQHLKSH    666
Xenopus.tropicalis                  LAFLVGTLTYRSQTNTLAIIESGGGILRNVSSLIATNEDHRQILRENNCLQTLLQHLKSH    674
Xenopus.laevis                      LAFLVSTLTYRSQTNTLAIIESGGGILRNVSSLIATNEDHRQILRENNCLQTLLQHLKSH    674
Ornithorhynchus.anatinus            LAFLVGTLTYRSQTNTLAIIESGGGILRNVSSLIATNEDHRQILRESSCLQTLLQHLKSH    672
Monodelphis.domestica               LAFLVGTLTYRSQTNTLAIIESGGGILRNVSSLIATNEDHRQILRENSCLQTLLQHLKSH    672
Mus.musculus                        LAFLVGTLTYRSQTNTLAIIESGGGILRNVSSLIATNEDHRQILRENNCLQTLLQHLKSH    670
Rattus.norvegicus                   LAFLVGTLTYRSQTNTLAIIESGGGILRNVSSLIATNEDHRQILRENNCLQTLLQHLKSH    670
Sorex.araneus                       LAFLVGTLTYRSQTNTLAIIESGGGILRNVSSLIATNEDHRQILRENNCLQTLLQHLKSH    672
Octodon.degus                       LAFLVGTLTYRSQTNTLAIIESGGGILRNVSSLIATNEDHRQILRENNCLQTLLQHLKSH    671
Heterocephalus                      LAFLVGTLTYRSQTNTLAIIESGGGILRNVSSLIATNEDHRQILRDNNCLQTLLQHLKSH    670
Jaculus.jaculus                     LAFLVGTLTYRSQTNTLAIIESGGGILRNVSSLIATNEDHRQILRENNCLQTLLQHLKSH    672
Loxodonta.africana                  LAFLVGTLTYRSQTNTLAIIESGGGILRNVSSLIATNEDHRQILRENNCLQTLLQHLKSH    672
Trichechus.manatus.latirostris      LAFLVGTLTYRSQTNTLAIIESGGGILRNVSSLIATNEDHRQILRDNNCLQTLLQHLKSH    672
```

```
Otolemur.garnettii                   LAFLVGTLTYRSQTNTLAIIESGGGILRNVSSLIATNEDHRQILRDNNCLQTLLQHLKSH    672
Callithrix.jacchus                   LAFLVGTLTYRSQTNTLAIIESGGGILRNVSSLIATNEDHRQILRENNCLQTLLQHLKSH    672
Macaca.mulatta                       LAFLVGTLTYRSQTNTLAIIESGGGILRNVSSLIATNEDHRQILRENNCLQTLLQHLKSH    672
Nomascus.leucogenys                  LAFLVGTLTYRSQTNTLAIIESGGGILRNVSSLIATNEDHRQILRENNCLQTLLQHLKSH    672
Pongo.abelii                         LAFLVGTLTYRSQTNTLAIIESGGGILRNVSSLIATNEDHRQILRENNCLQTLLQHLKSH    672
Homo.sapiens                         LAFLVGTLTYRSQTNTLAIIESGGGILRNVSSLIATNEDHRQILRENNCLQTLLQHLKSH    672
Gorilla.gorilla                      LAFLVGTLTYRSQTNTLAIIESGGGILRNVSSLIATNEDHRQILRENNCLQTLLQHLKSH    672
Pan.troglodytes                      LAFLVGTLTYRSQTNTLAIIESGGGILRNVSSLIATNEDHRQILRENNCLQTLLQHLKSH    672
Pan.paniscus                         LAFLVGTLTYRSQTNTLAIIESGGGILRNVSSLIATNEDHRQILRENNCLQTLLQHLKSH    672
Canis.lupus.familiaris               LAFLVGTLTYRSQTNTLAIIESGGGILRNVSSLIATNEDHRQILRENNCLQTLLQHLKSH    672
Bos.taurus                           LAFLVGTLTYRSQTNTLAIIESGGGILRNVSSLIATNEDHRQILRENNCLQTLLQHLKSH    672
Ovis.aries                           LAFLVGTLTYRSQTNTLAIIESGGGILRNVSSLIATNEDHRQILRENNCLQTLLQHLKSH    672
Orcinus.orca                         LAFLVGTLTYRSQTNTLAIIESGGGILRNVSSLIATNEDHRQILRENNCLQTLLQHLKSH    672
Sus.scrofa                           LAFLVGTLTYRSQTNTLAIIESGGGILRNVSSLIATNEEHRQILRENNCLQTLLQHLKSH    672
Ceratotherium.simum.simum            LAFLVGTLTYRSQTNTLAIIESGGGILRNVSSLIATNEDHRQILRDNNCLQTLLQHLKSH    672
Equus.caballus                       LAFLVGTLTYRSQTNTLAIIESGGGILRNVSSLIATNEDHRQILRENNCLQTLLQHLKSH    672
Felis.catus                          LAFLVGTLTYRSQTNTLAIIESGGGILRNVSSLIATNEDHRQILRENNCLQTLLQHLKSH    672
Ailuropoda.melanoleuca               LAFLVGTLTYRSQTNTLAIIESGGGILRNVSSLIATNEDHRQILRENNCLQTLLQHLKSH    672
Mustela.putorius.furo                LAFLVGTLTYRSQANTLAIIESGGGILRNVSSLIATNEDHRQILRENNCLQTLLQHLKSH    672
Odobenus.rosmarus.divergens          LAFLVGTLTYRSQTNTLAIIESGGGILRNVSSLIATNEDHRQILRENNCLQTLLQHLKSH    672
Taeniopygia.guttata                  LAFLVGTLTYRSQTNTLAIIESGGGILRNVSSLIATNEDHRQILRENSCLQTLLQHLKSH    672
Anolis.carolinensis                  LAFLVGTLTYRSQTNTLAIIESGGGILRNVSSLIATNEDHRQILRENSCLQTLLHHLKSH    672
Gallus.gallus                        LAFLVGTLTYRSQTNTLAIIESGGGILRNVSSLIATNEDHRQILRENSCLQTLLQHLKSH    680
                                     * ***.:***** :..  ::*.*******:** :**.* .**:**. .*** **::*.*

Ciona.intestinalis                   SLTIVSNACGTLWNLSARDETDQQTLRELGAVNKLQKLIHSKHTVIAQGSAAALRNLLAN    651
Strongylocentrotus.purpuratus        SLTIVSNACGTLWNLSARNKADQDLLWELGAVSMLKNLISSKHKMIAMGSSAALRNLMAS    674
Danio.rerio                          SLTIVSNACGTLWNLSARNAKDQEALWDMGAVSMLKNLIHSKHKMIAMGSAAALRNLMAN    726
Xenopus.tropicalis                   SLTIVSNACGTLWNLSARNAKDQEALWDMGAVSMLKNLIHSKHKMIAMGSAAALRNLMAN    734
Xenopus.laevis                       SLTIVSNACGTLWNLSARNAKDQEGLWDMGAVSMLKNLIHSKHKMIAMGSAAALRNLMAN    734
Ornithorhynchus.anatinus             SLTIVSNACGTLWNLSARNAKDQEALWDMGAVSMLKNLIHSKHKMIAMGSAAALRNLMAN    732
Monodelphis.domestica                SLTIVSNACGTLWNLSARNTKDQEALWDMGAVSMLKNLIHSKHKMIAMGSAAALRNLMAN    732
Mus.musculus                         SLTIVSNACGTLWNLSARNPKDQEALWDMGAVSMLKNLIHSKHKMIAMGSAAALRNLMAN    730
Rattus.norvegicus                    SLTIVSNACGTLWNLSARNPKDQEALWDMGAVSMLKNLIHSKHKMIAMGSAAALRNLMAN    730
Sorex.araneus                        SLTIVSNACGTLWNLSARNPKDQEALWDMGAVSMLKNLIHSKHKMIAMGSAAALRNLMAN    732
Octodon.degus                        SLTIVSNACGTLWNLSARNPKDQEALWDMGAVSMLKNLIHSKHKMIAMGSAAALRNLMAN    731
Heterocephalus                       SLTIVSNACGTLWNLSARNPKDQEALWDMGAVSMLKNLIHSKHKMIAMGSAAALRNLMAN    730
Jaculus.jaculus                      SLTIVSNACGTLWNLSARNPKDQEALWDMGAVSMLKNLIHSKHKMIAMGSAAALRNLMAN    732
Loxodonta.africana                   SLTIVSNACGTLWNLSARNPKDQEALWDMGAVSMLKNLIHSKHKMIAMGSAAALRNLMAN    732
Trichechus.manatus.latirostris       SLTIVSNACGTLWNLSARNPKDQEALWDMGAVSMLKNLIHSKHKMIAMGSAAALRNLMAN    732
Otolemur.garnettii                   SLTIVSNACGTLWNLSARNPKDQEALWDMGAVSMLKNLIHSKHKMIAMGSAAALRNLMAN    732
Callithrix.jacchus                   SLTIVSNACGTLWNLSARNPKDQEALWDMGAVSMLKNLIHSKHKMIAMGSAAALRNLMAN    732
Macaca.mulatta                       SLTIVSNACGTLWNLSARNPKDQEALWDMGAVSMLKNLIHSKHKMIAMGSAAALRNLMAN    732
Nomascus.leucogenys                  SLTIVSNACGTLWNLSARNPKDQEALWDMGAVSMLKNLIHSKHKMIAMGSAAALRNLMAN    732
Pongo.abelii                         SLTIVSNACGTLWNLSARNPKDQEALWDMGAVSMLKNLIHSKHKMIAMGSAAALRNLMAN    732
Homo.sapiens                         SLTIVSNACGTLWNLSARNPKDQEALWDMGAVSMLKNLIHSKHKMIAMGSAAALRNLMAN    732
Gorilla.gorilla                      SLTIVSNACGTLWNLSARNPKDQEALWDMGAVSMLKNLIHSKHKMIAMGSAAALRNLMAN    732
Pan.troglodytes                      SLTIVSNACGTLWNLSARNPKDQEALWDMGAVSMLKNLIHSKHKMIAMGSAAALRNLMAN    732
Pan.paniscus                         SLTIVSNACGTLWNLSARNPKDQEALWDMGAVSMLKNLIHSKHKMIAMGSAAALRNLMAN    732
Canis.lupus.familiaris               SLTIVSNACGTLWNLSARNPKDQEALWDMGAVSMLKNLIHSKHKMIAMGSAAALRNLMAN    732
Bos.taurus                           SLTIVSNACGTLWNLSARNPKDQEALWDMGAVSMLKNLIHSKHKMIAMGSAAALRNLMAN    732
Ovis.aries                           SLTIVSNACGTLWNLSARNPKDQEALWDMGAVSMLKNLIHSKHKMIAMGSAAALRNLMAN    732
Orcinus.orca                         SLTIVSNACGTLWNLSARNPKDQEALWDMGAVSMLKNLIHSKHKMIAMGSAAALRNLMAN    732
Sus.scrofa                           SLTIVSNACGTLWNLSARNPKDQEALWDMGAVSMLKNLIHSKHKMIAMGSAAALRNLMAN    732
Ceratotherium.simum.simum            SLTIVSNACGTLWNLSARNPKDQEALWDMGAVSMLKNLIHSKHKMIAMGSAAALRNLMAN    732
Equus.caballus                       SLTIVSNACGTLWNLSARNPKDQEALWDMGAVSMLKNLIHSKHKMIAMGSAAALRNLMAN    732
Felis.catus                          SLTIVSNACGTLWNLSARNPKDQEALWDMGAVSMLKNLIHSKHKMIAMGSAAALRNLMAN    732
Ailuropoda.melanoleuca               SLTIVSNACGTLWNLSARNPKDQEALWDMGAVSMLKNLIHSKHKMIAMGSAAALRNLMAN    732
Mustela.putorius.furo                SLTIVSNACGTLWNLSARNPKDQEALWDMGAVSMLKNLIHSKHKMIAMGSAAALRNLMAN    732
Odobenus.rosmarus.divergens          SLTIVSNACGTLWNLSARNPKDQEALWDMGAVSMLKNLIHSKHKMIAMGSAAALRNLMAN    732
Taeniopygia.guttata                  SLTIVSNACGTLWNLSARNAKDQEALWDMGAVSMLKNLIHSKHKMIAMGSAAALRNLMAN    732
Anolis.carolinensis                  SLTIVSNACGTLWNLSARNAKDQEALWDMGAVSMLKNLIHSKHKMIAMGSAAALRNLMAN    732
Gallus.gallus                        SLTIVSNACGTLWNLSARNAKDQEALWDMGAVSMLKNLIHSKHKMIAMGSAAALRNLMAN    740
                                     ******************:  **: * ::***. *::** ***.:** **:******:*.
```

```
Ciona.intestinalis                 RGDRLNTVFQNETKGDMPTLQTRKLRQLEADIQLASK-NAPEISENFSPRHHAHIT----    706
Strongylocentrotus.purpuratus      RPDVLATADGQK--EGTPGLHVRKQRALQAEIDKN-LKDTYAEMEGRTDQHGLLQSQQRA    731
Danio.rerio                        RPAKYKDANIMSPGSSLPSLHVRKQKALIEELDAQHLSETFDNIDNLSPKA---------    777
Xenopus.tropicalis                 RPAKYKDANIMSPGSSVPSLHVRKQKALEAELDAQHLSETFDNIDNLSPKT---------    785
Xenopus.laevis                     RPAKYKDANIMSPGSSVPSLHVRKQKALEAELDAQHLSETFDNIDNLSPKT---------    785
Ornithorhynchus.anatinus           RPAKYKDANIMSPGSSLPSLHVRKQKALEAELDAQHLSETFDNIDNLSPKA---------    783
Monodelphis.domestica              RPAKYKDANIMSPGSSLPSLHVRKQKALEAELDAQHLSETFDNIDNLSPKT---------    783
Mus.musculus                       RPAKYKDANIMSPGSSLPSLHVRKQKALEAELDAQHLSETFDNIDNLSPKA---------    781
Rattus.norvegicus                  RPAKYKDANIMSPGSSLPSLHVRKQKALEAELDAQHLSETFDNIDNLSPKA---------    781
Sorex.araneus                      RPAKYKDANIMSPGSSLPSLHVRKQKALEAELDAQHLSETFDNIDNLSPKA---------    783
Octodon.degus                      RPAKYKDANIMSPGSSLPSLHVRKQKALEAELDAQHLSETFDNIDNLSPKA---------    782
Heterocephalus                     RPAKYKDANIMSPGSSLPSLHVRKQKALEAELDAQHLSETFDNIDNLSPKA---------    781
Jaculus.jaculus                    RPAKYKDANIMSPGSSLPSLHVRKQKALEAELDAQHLSETFDNIDNLSPKA---------    783
Loxodonta.africana                 RPAKYKDANIMSPGSSLPSLHVRKQKALEAELDAQHLSETFDNIDNLSPKT---------    783
Trichechus.manatus.latirostris     RPAKYKDANIMSPGSSLPSLHVRKQKALEAELDAQHLSETFDNIDNLSPKA---------    783
Otolemur.garnettii                 RPAKYKDANIMSPGSSLPSLHVRKQKALEAELDAQHLSETFDNIDNLSPKA---------    783
Callithrix.jacchus                 RPAKYKDANIMSPGSSLPSLHVRKQKALEAELDAQHLSETFDNIDNLSPKA---------    783
Macaca.mulatta                     RPAKYKDANIMSPGSSLPSLHVRKQKALEAELDAQHLSETFDNIDNLSPKA---------    783
Nomascus.leucogenys                RPTKYKDANIMSPGSSVPSLHVRKQKALEAELDAQHLSETFDNIDNLSPKA---------    783
Pongo.abelii                       RPAKYKDANIMSPGSSLPSLHVRKQKALEAELDAQHLSETFDNIDNLSPKA---------    783
Homo.sapiens                       RPAKYKDANIMSPGSSLPSLHVRKQKALEAELDAQHLSETFDNIDNLSPKA---------    783
Gorilla.gorilla                    RPAKYKDANIMSPGSSLPSLHVRKQKALEAELDAQHLSETFDNIDNLSPKA---------    783
Pan.troglodytes                    RPAKYKDANIMSPGSSLPSLHVRKQKALEAELDAQHLSETFDNIDNLSPKA---------    783
Pan.paniscus                       RPAKYKDANIMSPGSSLPSLHVRKQKALEAELDAQHLSETFDNIDNLSPKA---------    783
Canis.lupus.familiaris             RPAKYKDANIMSPGSSLPSLHVRKQKALEAELDAQHLSETFDNIDNLSPKA---------    783
Bos.taurus                         RPAKYKDANIMSPGSSLPSLHVRKQKALEAELDAQHLSETFDNIDNLSPKA---------    783
Ovis.aries                         RPAKYKDANIMSPGSSLPSLHVRKQKALEAELDAQHLSETFDNIDNLSPKA---------    783
Orcinus.orca                       RPAKYKDANIMSPGSSLPSLHVRKQKALEAELDAQHLSETFDNIDNLSPKA---------    783
Sus.scrofa                         RPAKYKDANIMSPGSSLPSLHVRKQKALEAELDAQHLSETFDNIDNLSPKA---------    783
Ceratotherium.simum.simum          RPAKYKDANIMSPGSSLPSLHVRKQKALEAELDAQHLSETFDNIDNLSPKA---------    783
Equus.caballus                     RPAKYKDANIMSPGSSLPSLHVRKQKALEAELDAQHLSETFDNIDNLSPKA---------    783
Felis.catus                        RPAKYKDANIMSPGSSLPSLHVRKQKALEAELDAQHLSETFDNIDNLSPKA---------    783
Ailuropoda.melanoleuca             RPAKYKDANIMSPGSSLPSLHVRKQKALEAELDAQHLSETFDNIDNLSPKA---------    783
Mustela.putorius.furo              RPAKYKDANIMSPGSSLPSLHVRKQKALEAELDAQHLSETFDNIDNLSPKA---------    783
Odobenus.rosmarus.divergens        RPAKYKDANIMSPGSSLPSLHVRKQKALEAELDAQHLSETFDNIDNLSPKA---------    783
Taeniopygia.guttata                RPAKYKDANIMSPGSSLPSLHVRKQKALEAELDAQHLSETFDNIDNLSPKA---------    783
Anolis.carolinensis                RPAKYKDANIMSPGSSLPSLHVRKQKALEAELDAQHLSETFDNIDNLSPKT---------    783
Gallus.gallus                      RPAKYKDTNIMSPGSSLPSLHVRKQKALEAELDAQHLSETFDNIDNLSPKA---------    791
                                   *      .   .   . * *:.** : *  :::     ::    :. : :

Ciona.intestinalis                 ------------TNQNSMARLSLQE----PGFVQK------------SGVNSHLSPGI--    736
Strongylocentrotus.purpuratus      SLRNRGHRSRQQQHG---PDYS-------------------PVPQRIPIWNPNATPLPDS    769
Danio.rerio                        -----SHRVKPRHKHNVYGDY--------DAVCRSDGYNPNGVGVRSPYMNTPVLSSPS-    823
Xenopus.tropicalis                 -----THRNKQRHKQNLCSEYVLDSSRHDDSICRSDNFSLGNLTVLSPYINSTVLPG---    837
Xenopus.laevis                     -----THRNKQRHKQNLCSEYALDSSRHDDSICRSDNFSIGNLTVLSPYINTTVLPG---    837
Ornithorhynchus.anatinus           -----SHRNKQRHKQNLYSDYVFDPNRHDES--RSESFTTGNVTVLSPYLNATVLPVPGS    836
Monodelphis.domestica              -----SHRPKQRHKQSVYGEYALDASRHDDS--RPDAFSTGNLTVLSPYLNSTVLPG---    833
Mus.musculus                       -----SHRSKQRHKQNLYGDYAFDANRHDDS--RSDNFNTGNMTVLSPYLNTTVLPS---    831
Rattus.norvegicus                  -----SHRSKQRHKQNLYGDYVFDASRHDDN--RSDNFNTGNMTVLSPYLNTTVLPS---    831
Sorex.araneus                      -----SHRSKQRHKQSGYGDYVFDTSRHDDN--RSDGFNTGNMTVLSPYLNTTVLPN---    833
Octodon.degus                      -----SHRSKQRHKQSLYSDYVFDTNRHDDN--RSESFNTGNMTVLSPYLNTTVLPS---    832
Heterocephalus                     -----SHRSKQRHKQSLYGDYVFDTNRHDDN--RSDSFNTGNMTVLSPYLNTTVLPS---    831
Jaculus.jaculus                    -----SHRSKQRHKQNLYGDYVFDTNRHDDN--RSENFNTGNMTVLSPYLNTTVLPS---    833
Loxodonta.africana                 -----SHRSKQRHKQNLYGDYVFDANRHDDN--RSDNFNAGTMTVLSPYLNATVLPS---    833
Trichechus.manatus.latirostris     -----SHRSKQRHKQNLYGDYVFDASRHDDN--RSDNFNAGNMTVLSPYLNTTVLPS---    833
Otolemur.garnettii                 -----SHRSKQRHKQNLYGDYVFDTNRHDDN--RSDNFNAGNMTVLSPYLNTTVLPS---    833
Callithrix.jacchus                 -----SHRSKQRHKQSLYGDYVFDTNRHDDN--RSENFNTGNMTVLSPYLNTTVLPS---    833
Macaca.mulatta                     -----SHRSKQRHKQSLYGDYVFDTNRHEDN--RSDNFNAGNMTVLSPYLNTTVLPS---    833
Nomascus.leucogenys                -----SHRSKQRHKQSLYGDFVFDTNRHDDN--RSDNFNAGNMTVLSPYLNTTVLPS---    833
Pongo.abelii                       -----SHRSKQRHKQSLYGDYVFDTNRHDDN--RSDNFNTGNMTVLSPYLNTTVLPS---    833
Homo.sapiens                       -----SHRSKQRHKQSLYGDYVFDTNRHDDN--RSDNFNTGNMTVLSPYLNTTVLPS---    833
Gorilla.gorilla                    -----SHRSKQRHKQSLYGDYVFDTNRHDDN--RSDNFNTGNMTVLSPYLNTTVLPS---    833
Pan.troglodytes                    -----SHRSKQRHKQSLYGDYVFDTNRHDDN--RSDNFNTGNMTVLSPYLNTTVLPS---    833
Pan.paniscus                       -----SHRSKQRHKQSLYGDYVFDTNRHDDN--RSDNFNTGNMTVLSPYLNTTVLPS---    833
Canis.lupus.familiaris             -----SHRNKQRHKPNLYGDYVFDTNRHDDN--RSDNFNTGNMTVLSPYLNTTVLPS---    833
```

```
Bos.taurus                          -----SHRSKQRHKQNLYGDYVFDTNRHDDN--RSDNFNTGNMTVLSPYLNTTVLPS---    833
Ovis.aries                          -----SHRSKQRHKQNLYGDYVFDTNRHDDN--RSDNFNTGNMTVLSPYLNTTVLPS---    833
Orcinus.orca                        -----SHRSKQRHKQNLYGDYVFDTNRHDDN--RSDNFNTGNMTVLSPYLNTTVLPS---    833
Sus.scrofa                          -----SHRSKQRHKQNLYGDYAFDANRHDDN--RSDNFNTGNMTVLSPYLNTTVLPS---    833
Ceratotherium.simum.simum           -----SHRSKQRHKQSLYGDYVFDTNRHDDN--RPDNFNTGNMTVLSPYLNTTVLPS---    833
Equus.caballus                      -----SHRSKQRHKQSLYGDYVFDTNRHDDN--RPDNFNTGNMTVLSPYLNTTVLPS---    833
Felis.catus                         -----SHRSKQRHKPNLYGDYVFDTNRHDDN--RSDNFNTGNMTVLSPYLNTTVLPS---    833
Ailuropoda.melanoleuca              -----SHRNKQRHKQSLYSDYGFDTNRHDDN--RSDNFNAGNMTVLSPYLNTTVLPS---    833
Mustela.putorius.furo               -----SHRNKQRHKQSLYSDYVFDTNRHDDN--RSDNFNTGNMTVLSPYLNTTVLPS---    833
Odobenus.rosmarus.divergens         -----SHRNKQRHKQSLYSDYVFDTNRHDDN--RSDNFNTGNMTVLSPYLNTTVLPS---    833
Taeniopygia.guttata                 -----SHRNKQRHKQNIYSEYVLDASRHDDGVCRSESFNAGNMTVLSPYVNTTVLPG--S    836
Anolis.carolinensis                 -----SHRNKQRHKQNLYSEYVLDGSRHDDGVCRSENFNTNN--VLSPYLNTTVLPG--S    834
Gallus.gallus                       -----SHRNKQRHKQNIYGEYVLDSSRHDDGVCRTESFNTGNMTVLSPYLNSTVLPG--S    844
                                                 :                                    *

Ciona.intestinalis                  -------------HYSGSLGNLPQ-----QTYSPSPGLPFRA----WEDSGSK---NMLR    771
Strongylocentrotus.purpuratus       LMNSQQRAHSPSNRHHGNMINSPETCSDDQSFQEGKTKD-SEGQSTSSQPGSVE--NSPG    826
Danio.rerio                         SRDN-----------RGNAESVR--AERDRSLDRERRGFLPD---------GEAAKRMMQ    861
Xenopus.tropicalis                  SSSP-----------RPTMEGNR--PEK----DRERTAGLSNYHSATENSGNSSKRIGVQ    880
Xenopus.laevis                      SSSP-----------RPTMDGSR--PEK----DRERTAGLGNYHSTTESSGNSSKRIGIQ    880
Ornithorhynchus.anatinus            SSSA-----------RGGLESSR--SEKDRSLDRERGIGLGAYHPAPENPGNSSKRIGMQ    883
Monodelphis.domestica               SSSS-----------RTSLESSR--SEKDRSLDRERAVALSTFHPAADSPGNPSKRLGMQ    880
Mus.musculus                        SSSS-----------RGSLDSSR--SEKDRSLERERGIGLSAYHPTTENAGTSSK-RGLQ    877
Rattus.norvegicus                   SSSS-----------RGSLDSSR--SEKDRSLERERGIGLSTYHSATENPGTSSK-RGLQ    877
Sorex.araneus                       S-SS-----------RGSLDSSR--SEKDRSLERERGISLGNYHPATENPGTSSK-RGLQ    878
Octodon.degus                       SSSS-----------RGSLDSSR--SEKDRSLERERGIGLSGYHPTTENPGTSSK-RNLQ    878
Heterocephalus                      SSSS-----------RGSLDSSR--SEKDRSLEREREIGLSSYHPATENPGTSSK-RGLQ    877
Jaculus.jaculus                     SSSS-----------RGSLDSSR--SEKDRSLERERGLGLSTYHPATENPGTSSK-RGLQ    879
Loxodonta.africana                  SSSS-----------RGSLDSSR--SEKDRSLERERGLGLGTYHPATENPGTSSK-RSLQ    879
Trichechus.manatus.latirostris      SSSS-----------RGSLDSSR--SEKDRSLERERGLGLGSYHPATENPGTSSK-RGLQ    879
Otolemur.garnettii                  SSSS-----------RGSLDSSR--SEKDKSLERERGIGLGNYHPGTENPGTSSK-RGLQ    879
Callithrix.jacchus                  SSSS-----------RGSLDSSR--SEKDRSLERERGIGLGNYHPATENPGTSSK-RGLQ    879
Macaca.mulatta                      SSSS-----------RGSLDSSR--SEKDRSLERERGIGLGNYHPATENPGTSSK-RGLQ    879
Nomascus.leucogenys                 SSSS-----------RGSLDSSR--SEKDRSLERERGIGLGNYHPATENPGTSSK-RGLQ    879
Pongo.abelii                        SSSS-----------RGSLDSSR--SEKDRSLERERGIGLGNYHPATENPGTSSK-RGLQ    879
Homo.sapiens                        SSSS-----------RGSLDSSR--SEKDRSLERERGIGLGNYHPATENPGTSSK-RGLQ    879
Gorilla.gorilla                     SSSS-----------RGSLDSSR--SEKDRSLERERGIGLGNYHPATENPGTSSK-RGLQ    879
Pan.troglodytes                     SSSS-----------RGSLDSSR--SEKDRSLERERGIGLGNYHPATENPGTSSK-RGLQ    879
Pan.paniscus                        SSSS-----------RGSLDSSR--SEKDRSLERERGIGLGNYHPATENPGTSSK-RGLQ    879
Canis.lupus.familiaris              SSSS-----------RGSLDSSR--SEKDRSLERERGISLGNYHPATENPGTSSK-RGLQ    879
Bos.taurus                          SSSS-----------RGSLDSSR--SEKDRSLERERGISLGNYHPATENPGTSSK-RGLQ    879
Ovis.aries                          SSSS-----------RGSLDSSR--SEKDRSLERERGISLGNYHPATENPGTSSK-RGLQ    879
Orcinus.orca                        SSSS-----------RGSLDSSR--SEKDRSLERERGISLVNYHPATENPGTSSK-RGLQ    879
Sus.scrofa                          SSSS-----------RGSLDSSR--SEKDRSLERERGISIGNYHPATENPGTSSK-RGLQ    879
Ceratotherium.simum.simum           SSSS-----------RGSLDSSR--SEKDRSLERERGISLGNYHPATENSGTSSK-RGLQ    879
Equus.caballus                      SSSS-----------RGSLDSSR--SEKDRSLERERGISLGNYHPATENSGSSSK-RGLQ    879
Felis.catus                         SSSS-----------RGSLDSSR--SEKDRSLERERGISLGNYHPATENPGTSSK-RGLQ    879
Ailuropoda.melanoleuca              SSSS-----------RGSLDSSR--SEKDRSLERERGISLGNYHPATENPGTSSK-RGLQ    879
Mustela.putorius.furo               SSSS-----------RGSLESSR--SEKDRSLERERGISLGNFHPATENSGTSSK-RGLQ    879
Odobenus.rosmarus.divergens         SSSS-----------RGSVDSSR--SEKDRSLERERGISLGSYHPATENPGTSSK-RGLQ    879
Taeniopygia.guttata                 S--S-----------RGNTENSR--SEKDRSVERDRTVGLNTYHQAAESTGNSSKRIGMQ    881
Anolis.carolinensis                 SSSS-----------RSNAESSR--SEKDRSLDRERSIGLSSYHSATENSGNASKRIGMQ    881
Gallus.gallus                       ASSS-----------RGNIENCL--SEKDRSLDRDRAVGLNAYHPATENSGNSSKRIGMQ    891
                                                        .           .

Ciona.intestinalis                  APTSNSGVFQN--HPLSSHLQFNNN-----------------IS----------RATSF    801
Strongylocentrotus.purpuratus       RPHGASRIAQIMQEVAQDGLP--TDSSSGSESPRRESLESRLQSRNGHQQKPTCPRSSSF    884
Danio.rerio                         IPTSAAQIAVVMEEVQNMHLG-MDDRSAGSTPDPHSVQDD--MIRRQ-----------T-    906
Xenopus.tropicalis                  LSTT-AQISKVMDEVSNIHMV-QEDRSSGSASEMHCMSDERTSQRKP-----------S-    926
Xenopus.laevis                      LSTT-AQISKVMDEVSNIHLV-QENRSSGSASEMHCMSDERNSQRKP-----------S-    926
Ornithorhynchus.anatinus            LSTTAAQIAKVMEEVSSIHVA-QDDRSSGPTTEMHCMADERTALRRA-----------S-    930
Monodelphis.domestica               LSTTTAQIAKVMEEVSAIHA--QEDQSSASTTDLHCVAEERSTLRRA-----------S-    926
Mus.musculus                        ITTTAAQIAKVMEEVSAIHTS-QDDRSSASTTEFHCVADDRSAARRS-----------S-    924
Rattus.norvegicus                   LSATAAQIAKVMEEVSALHTS-QDDRSPASAAELHCVAEERTAARRS-----------S-    924
Sorex.araneus                       ISTTAAQIAKVMEEVSAIHSS-QEDRTSGSATEIHCGTDERNALRRS-----------S-    925
```

```
Octodon.degus                       ISTTAAQIAKVMEEVSAIHTSSQEDRNSGSTTELHCVTEERNAIRRS-----------S-    926
Heterocephalus                      ISTTAVQIAKVMEEVSVIHTS-QEDRNSGSTTELHCVTDERNAIRRS-----------S-    924
Jaculus.jaculus                     ISTTAAQIAKVMEEVSAIHTS-QEERSSGSTTDLHCVTDERNAVRRS-----------S-    926
Loxodonta.africana                  ISTTAAQIAKVMEEVSAIHTP-QEDRSSGSTSESHCVTDERNALRRS-----------S-    926
Trichechus.manatus.latirostris      ISTTVAQIAKVMEEVSAIHTP-QEDRSSESTTELHCVPDERNALRRS-----------S-    926
Otolemur.garnettii                  ISTTAAQIAKVMEEVSAIHTS-QEERSSGSTTELHCVTDERNPLRRS-----------S-    926
Callithrix.jacchus                  ITTTAAQIAKVMEEVSAIHTS-QEDRSSGSTTELHCVTDERNALRRS-----------S-    926
Macaca.mulatta                      ISTTAAQIAKVMEEVSAIHTS-QEDRSSGSTTELHCVTDERNALRRS-----------S-    926
Nomascus.leucogenys                 LSTTAAQIAKVMEEVSAIHTS-QEDRSSGSTTELHCVTDERNALRRS-----------S-    926
Pongo.abelii                        ISTTAAQIAKVMEEVSAIHTS-QEDRSSGSTTELHCVTDERNALRRS-----------S-    926
Homo.sapiens                        ISTTAAQIAKVMEEVSAIHTS-QEDRSSGSTTELHCVTDERNALRRS-----------S-    926
Gorilla.gorilla                     ISTTAAQIAKVMEEVSAIHTS-QEDRSSGSTTELHCVTEERNALRRS-----------S-    926
Pan.troglodytes                     ISTTAAQIAKVMEEVSAIHTS-QEDRSSGSTTELHCVTDERNALRRS-----------S-    926
Pan.paniscus                        ISTTAAQIAKVMEEVSAIHTS-QEDRSSGSTTELHCVTDERNALRRS-----------S-    926
Canis.lupus.familiaris              ISTTAAQIAKVMEEVSAIHTS-QEDRSSGSTTELHCGTDERNTLRRS-----------S-    926
Bos.taurus                          ISTTAAQIAKVMEEVSAIHTS-QEDRSSGSTTELHCGTDERNALRRS-----------S-    926
Ovis.aries                          IPTTAAQIAKVMEEVSAIHTS-QEDRSSGSTTELHCGTDERNALRRS-----------S-    926
Orcinus.orca                        ISTTAAQIAKVMEEVSAIHTS-QEDRSSGSTPELHCGTDERNALRRS-----------S-    926
Sus.scrofa                          ISTTAAQIAKVMEEVSAIHPS-QEDRNSGSTTELHCGTDERNALRRS-----------S-    926
Ceratotherium.simum.simum           ISTTAAQIAKVIEEVSAIHTS-QEDRSSGSTTELHCGTDERNALRRS-----------S-    926
Equus.caballus                      ISTTAAQIAKVMEEVSAIHTS-QEDRSSGSTTELHCGTDERNALRRS-----------S-    926
Felis.catus                         ISTTAAQIAKVMEEVSAIHTS-QEDRSSGSTGELHCGTDERNALRRS-----------S-    926
Ailuropoda.melanoleuca              ISTTAAQIAKVMEEVSAIHTS-QEDRSSGSTTELHCGTDERNALRRS-----------S-    926
Mustela.putorius.furo               ISTTAAQIAKVMEEVSAIHTS-QEDRSSGSTTELHCGTDERNALRRS-----------S-    926
Odobenus.rosmarus.divergens         ISTTAAQIAKVMEEVSAIHTS-QEDRSSGSTTEVHCGTEERNALRRS-----------S-    926
Taeniopygia.guttata                 ISTAAAQIAKVMEEVTNMHIP-QEDRSSGSTSEIHCLTEDRNSQRRS-----------A-    928
Anolis.carolinensis                 ISTAAAQISKVMEEVTNMHIP-PEERNNGSVTEMHCLSEDRSALRRT-----------S-    928
Gallus.gallus                       ISTAAAQIAKVMEEVTSMHIP-QEDRSSGSTSEMHCLTEDRNTTRRA-----------A-    938
                                           :     .          :                                :

Ciona.intestinalis                  ADQ-----NQNPFSNSLPSSRTKSGNLYGKLPQCDFKAAKTWGHYHSVNNNSNNT---EL    853
Strongylocentrotus.purpuratus       THMPPEGSNLSSRSNSYCF--GFDGG------------HGL--VARRSSTESINSISSDI    928
Danio.rerio                         --------AVHGHQNIYSY--SKTDP---SGRPCPMPKL----EY-RASNDSLNSVNSTD    948
Xenopus.tropicalis                  --------SNHPHSNPFSF--AKGDS---SNRGCPVAFMKM--EYKMASNDSLNSVSSTD    971
Xenopus.laevis                      --------SNHPQSNPFTF--TKAES---STRGCPVAFMKM--EYKMASNDSLNSVSSTE    971
Ornithorhynchus.anatinus            --------TAHPHSSTYSF--PKSEN---ANRTCPVPYPKM--EYERASNDSLNSVSSSD    975
Monodelphis.domestica               --------AAHAHSNTYNF--PKPDN---SNRTCAMPYAKV--EYKRSSNDSLNSVSSSD    971
Mus.musculus                        --------ASHTHSNTYNF--TKSEN---SNRTCSMPYAKV--EYKRSSNDSLNSVTSSD    969
Rattus.norvegicus                   --------ASHTHPNTHNF--AKSES---SNRTCSMPYAKV--EYKRSSNDSLNSVTSSD    969
Sorex.araneus                       --------TAHTHSNTYNF--TKSDN---SNRTCPMPYSKL--EYKRSSNDSLNSVSSSD    970
Octodon.degus                       --------AAHTHSNTYNF--TKTEN---SNRTCSMPYAKL--EYKRSSNDSLNSVSSSD    971
Heterocephalus                      --------AAHTHSNTYNF--TKSEN---SNRTCSMPYAKL--EYKRSSNDSLNSVSSSD    969
Jaculus.jaculus                     --------AAHTHSNTYNF--TKSEN---SNRTCSMPYAKA--EYKRSSNDSLNSVSSSD    971
Loxodonta.africana                  --------AAHTHSNTYNF--TKSEN---SNRTCPMPYAKL--EYKRSSNDSLNSVSSSD    971
Trichechus.manatus.latirostris      --------AAHTHSNTYNF--TKSEN---SNRTCPLPYAKL--EYKRSSNDSLNSVSSSD    971
Otolemur.garnettii                  --------AVHTHPNTYSF--TKSEN---SNRTCSMPYAKL--EYKRSSNDSLNSVSSSD    971
Callithrix.jacchus                  --------TAHTHSNTYNF--TKSEN---SNRTCSMPYAKL--EYKRSSNDSLNSVSSSD    971
Macaca.mulatta                      --------AAHTHSNTYNF--TKSEN---SNRTCSMPYAKL--EYKRSSNDSLNSVSSSD    971
Nomascus.leucogenys                 --------AAHTHSSTYNF--SKSEN---SNRTCSMPYAKL--EYKRSSNDSLNSVSSSD    971
Pongo.abelii                        --------AAHTHSNTYNF--TKSEN---SNRTCSMPYAKL--EYKRSSNDSLNSVSSSD    971
Homo.sapiens                        --------AAHTHSNTYNF--TKSEN---SNRTCSMPYAKL--EYKRSSNDSLNSVSSSD    971
Gorilla.gorilla                     --------AAHTHSNTYNF--TKSEN---SNRTCSMPYAKL--EYKRSSNDSLNSVSSSD    971
Pan.troglodytes                     --------AAHTHSNTYNF--TKSEN---SNRTCSMPYAKL--EYKRSSNDSLNSVSSSD    971
Pan.paniscus                        --------AAHTHSNTYNF--TKSEN---SNRTCSMPYAKL--EYKRSSNDSLNSVSSSD    971
Canis.lupus.familiaris              --------TAHTHANTYSF--TKSEN---SNRTCPVPYAKL--EYKRSSNDSLNSVSSSD    971
Bos.taurus                          --------TTHTHANTYNF--TKSEN---SNRTCPIPYAKV--EYKRSSNDSLNSVSSSD    971
Ovis.aries                          --------TTHTHANTYNF--TKSEN---SNRTCPIPYAKV--EYKRSSNDSLNSVSSSD    971
Orcinus.orca                        --------TAHTHANSYNF--TKSEN---SNRTCPVPYAKV--EYKRSSNDSLNSVSSSD    971
Sus.scrofa                          --------TAHSHANTYNF--TKSEN---SNRTCPMPYAKV--EYKRSSNDSLNSVSSSD    971
Ceratotherium.simum.simum           --------TAHTHANTYNF--TKSEN---SNRTCPMPYAKL--EYKRSSNDSLNSVSSSD    971
Equus.caballus                      --------TAHTHANTYNF--TKSEN---SNRTCPMPYAKL--EYKRSSNDSLNSVSSSD    971
Felis.catus                         --------TAHTHANTYNF--TKSEN---SNRTCPMPYAKL--EYKRSSNDSLNSVSSSD    971
Ailuropoda.melanoleuca              --------TAHTHASTYNF--SKSEN---SNRTCPLPYAKL--EYKRSSNDSLNSVSSSD    971
Mustela.putorius.furo               --------TAHTHANTYSF--SKSEG---SNRTCPMTYANL--EYKRSSNDSLNSVSSSD    971
Odobenus.rosmarus.divergens         --------TAHSHANTYNF--SKSEN---ATRTCPMPYAKL--EYKRSSNDSLNSVSSSD    971
```

```
Taeniopygia.guttata                 --------SAHTHSNTY-F--PKSEN---SNRTCPVPYTKM--EYKRASNDSLNSVSSSD    972
Anolis.carolinensis                 --------AAHSHTSAYNF--PKS-----ENRTCPVPYAKV--EYKRASNDSLNSVSSSD    971
Gallus.gallus                       --------TAHTHSNTY-F--PKSEN---SSRPCPVPYTKM--EYKRASNDSLNSVSSSD    982
                                                  .                                 ..:* *:

Ciona.intestinalis                  W--------------NP--NQSGNGNPEKMLSPSRMPDDLEEKLRHMN--DDYGMDQDD    894
Strongylocentrotus.purpuratus       FPAGIHERLAQNRSQMDHS---QSADSSLNMHGTGRSLQNTTALVHSAD--EAFG-TNMD    982
Danio.rerio                         ---GYG-----KRGQMKPSVDSYSEDDEGKCCVYRKYPADLAHKIHNANHMEDDN-GDLD    999
Xenopus.tropicalis                  ---GYG-----KRGQVKPSVESYSEDDEGKFGSYGHYPAGLAHKIHSANHMDDND-TELD    1022
Xenopus.laevis                      ---GYG-----KRGQVKPSVESYSEDDESKFFSYGQYPAGLAHKIQSANHMDDND-TELD    1022
Ornithorhynchus.anatinus            ---GYG-----KRGQMKPSVESYSEDDESKFCSYGQYPADLAHKIHSANHMDDND-GDLD    1026
Monodelphis.domestica               ---GYG-----KRGQMKPSIESYSEDDESKFCSYGQYPADLAHKIHSANHMDDND-EELD    1022
Mus.musculus                        ---GYG-----KRGQMKPSVESYSEDDESKFCSYGQYPADLAHKIHSANHMDDND-GELD    1020
Rattus.norvegicus                   ---GYG-----KRGQMKPSVESYSEDDEGKFCSYGQYPADLAHKIHSANHMDDNG-GELD    1020
Sorex.araneus                       ---GYG-----KRGQMKPSIESYSEDDESKFCSYGQYPADLAHKIHSANHMDDND-GELD    1021
Octodon.degus                       ---GYG-----KRGQMKPSIESYSEDDESKFCSYGQYPADLAHKIHSANHMDDND-GELD    1022
Heterocephalus                      ---GYG-----KRGQMKPSIESYSEDDESKFCSYGQYPADLAHKIHSANHMDDND-GELD    1020
Jaculus.jaculus                     ---GYG-----KRGQMKPSIESYSEDDESKFCSYGQYPADLAHKIHSANHMDDND-GELD    1022
Loxodonta.africana                  ---GYG-----KRGQMKPSIESYSEDDESKFCSYGQYPADLAHKIHSANHMDDND-GELD    1022
Trichechus.manatus.latirostris      ---GYG-----KRGQMKPSVESYSEDDESKFCSYGQYPADLAHKIHSANHMDDND-GELD    1022
Otolemur.garnettii                  ---GYG-----KRGQMKPSIESYSEDDESKFCSYGQYPADLAHKIHSANHMDDND-GELD    1022
Callithrix.jacchus                  ---GYG-----KRGQMKPSIESYSEDDESKFCSYGQYPADLAHKIHSANHMDDND-GELD    1022
Macaca.mulatta                      ---GYG-----KRGQMKPSIESYSEDDESKFCSYGQYPADLAHKIHSANHMDDND-GELD    1022
Nomascus.leucogenys                 ---GYG-----KRGQMKPSIESYSEDDESKFCSYGQYPADLAHKIHSANHMDDND-GELD    1022
Pongo.abelii                        ---GYG-----KRGQMKPSIESYSEDDESKFCSYGQYPADLAHKIHSANHMDDND-GELD    1022
Homo.sapiens                        ---GYG-----KRGQMKPSIESYSEDDESKFCSYGQYPADLAHKIHSANHMDDND-GELD    1022
Gorilla.gorilla                     ---GYG-----KRGQMKPSIESYSEDDESKFCSYGQYPADLAHKIHSANHMDDND-GELD    1022
Pan.troglodytes                     ---GYG-----KRGQMKPSIESYSEDDESKFCSYGQYPADLAHKIHSANHMDDND-GELD    1022
Pan.paniscus                        ---GYG-----KRGQMKPSIESYSEDDESKFCSYGQYPADLAHKIHSANHMDDND-GELD    1022
Canis.lupus.familiaris              ---GYG-----KRGQMKPSIESYSEDDESKFCSYGQYPADLAHKIHSANHMDDND-GELD    1022
Bos.taurus                          ---GYG-----KRGQMKPSIESYSEDDESKFCSYGQYPADLAHKIHSANHMDDND-GELD    1022
Ovis.aries                          ---GYG-----KRGQMKPSIESYSEDDESKFCSYGQYPADLAHKIHSANHMDDND-GELD    1022
Orcinus.orca                        ---GYG-----KRGQMKPSVESYSEDEESKFCSYGQYPADLAHKIHSANHMDDND-GELD    1022
Sus.scrofa                          ---GYG-----KRGQMKPSIESYSEDDESKFCSYGQYPADLAHKIHSANHMDDND-GELD    1022
Ceratotherium.simum.simum           ---GYG-----KRGQMKPSIESYSEDDESKFCSYGQYPADLAHKIHSANHMDDND-GEVD    1022
Equus.caballus                      ---GYG-----KRGQMKPSIESYSEDDESKFCSYGQYPADLAHKIHSANHMDDND-GELD    1022
Felis.catus                         ---GYG-----KRGQMKPSIESYSEDDESKFCSYGQYPADLAHKIHSANHMDDND-GELD    1022
Ailuropoda.melanoleuca              ---GYG-----KRGQMKPSIESYSEDDESKFCSYGQYPADLAHKIHSANHMDDND-GELD    1022
Mustela.putorius.furo               ---GYG-----KRGQMKPSIESYSEDDESKFCSYGQYPADLAHKIHSANHMDDND-GELD    1022
Odobenus.rosmarus.divergens         ---GYG-----KRGQMKPSIESYSEDDESKFCSYGQYPADLAHKIHSANHMDDND-GELD    1022
Taeniopygia.guttata                 ---GYG-----KRGQMKPSIESYSEDDESKFCSYGQYPADLAHKIHSANHMDDND-EELD    1023
Anolis.carolinensis                 ---GYG-----KRGQMKP-IESYSEDEESKFCSYGQYPADLAHKIHSANHMDDND-GELD    1021
Gallus.gallus                       ---GYG-----KRGQMKPSIESYSEDDESKFCSYGQYPADLAHKIHSANHMDDND-GELD    1033
                                                    .      . ..  :     :   .    ::  :  :  .  : *

Ciona.intestinalis                  LPVNYSLQFTDEQSQTPGIQSPRTEEIYEKSATRMPKKSSA--------------IATKP    940
Strongylocentrotus.purpuratus       STTNYSLKYSEEDL-PPGMHSPKRTAPVE---HRCKDGNS-----------QHSSVDGEQ    1027
Danio.rerio                         TPINYSLKYSDEQL-NSGRQSPSQNERWARPK-LLDDEMKRPDQKPPRSQSPGYPMYTEG    1057
Xenopus.tropicalis                  TPINYSLKYSDEQL-NSGRQSPSQNERWARPKHIIDSEMKQSEQRQPRTTKTTYSSYTEN    1081
Xenopus.laevis                      TPINYSLKYSDEQL-NSGRQSPTQNERWSRPKHIIDSEMKQSEQRQPRTTKTTYSSYTEN    1081
Ornithorhynchus.anatinus            TPINYSLKYSDEQL-NSGRQSPSQNERWARPKHMIEDELKQNEQRQSRGPSTTYPGYGES    1085
Monodelphis.domestica               TPINYSLKYSDEQL-NSGRQSPSQNDRWARPKHVIEDEIKQNEQRQARGQNTPFSAYSES    1081
Mus.musculus                        TPINYSLKYSDEQL-NSGRQSPSQNERWARPKHVIEDEIKQNEQRQARSQNTSYPVYSEN    1079
Rattus.norvegicus                   TPINYSLKYSDEQL-NSGRQSPSQNERWARPKHVIEDEIKQNEQRQSRSQNTNFPVYSEN    1079
Sorex.araneus                       TPINYSLKYSDEQL-NSGRQSPSQNERWSRPKHILEDEIKQSEQRQPRTQSTTYPLYTET    1080
Octodon.degus                       TPINYSLKYSDEQL-NSGRQSPSQNERWARPKHIIEDEIKQNEQRQSRNQSTTYSVYSES    1081
Heterocephalus                      TPINYSLKYSDEQL-NSGRQSPSQNERWARPKHIIEDEIKQSEQRQSRSQNTTYPVYTES    1079
Jaculus.jaculus                     TPINYSLKYSDEQL-NSGRQSPSQNERWARPKHIIEDEIKQSEQRQSRTQNTTYPVYTES    1081
Loxodonta.africana                  TPINYSLKYSDEQL-NSGRQSPSQNERWARPKHIIEDEIKQSEQRQSRSQSTTFPVYSES    1081
Trichechus.manatus.latirostris      TPINYSLKYSDEQL-NSGRQSPSQNERWARPKHIIEDEIKQSEQRQSRSQSTTFPVYNES    1081
Otolemur.garnettii                  TPINYSLKYSDEQL-NSGRQSPSQNERWARPKHIIEDEMKQSEQRQSRSQSTTYPGYTEN    1081
Callithrix.jacchus                  TPINYSLKYSDEQL-NSGRQSPSQSERWARPKHIIEDEIKQSEQRQSRSQSTTYPVYTET    1081
Macaca.mulatta                      TPINYSLKYSDEQL-NSGRQSPSQNERWARPKHIIEDEIKQSEQRQSRSQSTTYPVYTES    1081
Nomascus.leucogenys                 TPINYSLKYSDEQL-NSGRQSPSQNERWARPKHIIEDELKQSEQRQSRNQSTTYPVYTES    1081
Pongo.abelii                        TPINYSLKYSDEQL-NSGRQSPSQNERWARPKHIIEDEIKQSEQRQSRNQSTTYPVYTES    1081
```

```
Homo.sapiens                      TPINYSLKYSDEQL-NSGRQSPSQNERWARPKHIIEDEIKQSEQRQSRNQSTTYPVYTES    1081
Gorilla.gorilla                   TPINYSLKYSDEQL-NSGRQSPSQNERWARPKHIIEDEIKQSEQRQSRNQSTAYPVYTES    1081
Pan.troglodytes                   TPINYSLKYSDEQL-NSGRQSPSQNERWARPKHIIEDEIKQSEQRQSRNQSTTYPVYTES    1081
Pan.paniscus                      TPINYSLKYSDEQL-NSGRQSPSQNERWARPKHIIEDEIKQSEQRQSRNQSTTYPVYTES    1081
Canis.lupus.familiaris            TPINYSLKYSDEQL-NSGRQSPSQNERWARPKHIIEDEIKQSEQRQSRSQSTTYPVYTES    1081
Bos.taurus                        TPINYSLKYSDEQL-NSGRQSPSQNERWARPKHILEDEIKPNEQRQSRSQSTAYPVYPES    1081
Ovis.aries                        TPINYSLKYSDEQL-NSGRQSPSQNERWARPKHILEDEIKPNEQRQSRSQSTAYPVYPES    1081
Orcinus.orca                      TPINYSLKYSDEQL-NSGRQSPSQNERWARPKHIIEDEIKQNEERQSRSQSTTYPVYPES    1081
Sus.scrofa                        TPINYSLKYSDEQL-NSGRQSPSQNERWARPKHIIEDEIKQNEQRQSRSQSTTYPVYPES    1081
Ceratotherium.simum.simum         TPINYSLKYSDEQL-NSGRQSPSQNERWARPKHIIEDEIKQSEQRQSRSQSTTYPVYTES    1081
Equus.caballus                    TPINYSLKYSDEQL-NSGRQSPSQNERWARPKHIIEDEIKQSEQRQSRSQSTTYPVYTES    1081
Felis.catus                       TPINYSLKYSDEQL-NSGRQSPSQNERWARPKHIIEDEIKQSEQRQSRSQSTTYPVYTES    1081
Ailuropoda.melanoleuca            TPINYSLKYSDEQL-NSGRQSPSQNERWARPKHIIEDEIKQSEQRQSRSQGTTYPVYTES    1081
Mustela.putorius.furo             TPINYSLKYSDEQL-NSGRQSPSQNERWARPKHIIEDEIKQSEQRQSRSQSTTYPVYTES    1081
Odobenus.rosmarus.divergens       TPINYSLKYSDEQL-NSGRQSPSQNERWARPKHIIEDEIKQSEQRQSRSQTTTYPVYTES    1081
Taeniopygia.guttata               TPINYSLKYSDEQL-NSGRQSPSQNERWARPKHIIDDEMKQNEQRQSRNQNATYPVYTES    1082
Anolis.carolinensis               TPINYSLKYSDEQL-NSGRQSPSQNERWARPKHIIEEEIKQSEQRQSRSQSGAYPVYTEG    1080
Gallus.gallus                     TPINYSLKYSDEQL-NSGRQSPSQNERWARPKHIIDDEMKQNDQRQSRSQSATYPVYTES    1092
                                     ****::::*:    * :**          .  .                :

Ciona.intestinalis                E--------------------VPGKSMGNAVVGNKALKIAPRRHKPPAKVDFSVEQRI--    978
Strongylocentrotus.purpuratus     SEEGL---------SQPCQKCNSPHRPREGLAIGNSYPASQQQRPFPQQYSDQSVSSHHNV   1079
Danio.rerio                       SSEGEDKPKKYQPRFVQQDL-PAFRSRG----SNEQISSGSSHGLNKK-ISQTICSVDDY    1111
Xenopus.tropicalis                K---EEKHKKFPPHFNQPENAPAYARSRGTNNQADQSRVGSNLSNNSK-ASKPHCQVDDY    1137
Xenopus.laevis                    K---EEKHKKFPPHFNQSENVPAYTRSRGANNQVDQSRVSSNLSNNSK-ASKPHCQVDDY    1137
Ornithorhynchus.anatinus          S---DDKHIKFQSHFGQQECVSPYRSR-G-ANNAEPNRVGSGHGMNQK-VNPSLCQEDDY    1139
Monodelphis.domestica             T---DDKHMKFQSRFGQQECVSPYRSR-G-ASGSEQNRVSSGHGINQK-VNQSLCHEDDY    1135
Mus.musculus                      T---DDKHLKFQPHFGQQECVSPYRSR-G-TSGSETNRMGSSHAINQN-VNQSLCQEDDY    1133
Rattus.norvegicus                 T---DDKHLKFQQHFGQQECVSPYRSR-G-TNGSETNRMGSSHAVNQN-VNQSLCQEDDY    1133
Sorex.araneus                     T---DDKHLKFQPHFGQQECVSPYRSR-G-ANGSEANRGGSNHGINQN-VSQSLCQEDDY    1134
Octodon.degus                     T---DDKHLKFQPHFGQQECVSPYRSR-G-TSGSEANRVGSSHGINQN-VKQSLCQEDDY    1135
Heterocephalus                    N---DDKHLKFQPHFGQQECVSPYRSR-G-TNGSEANRVGSSHGINQN-VNQSLCQEDDY    1133
Jaculus.jaculus                   T---DDKHLKFQPHFGQQECVSPYRSR-G-TSGSETNRVGSSHGINQN-VSQSLCQEDDY    1135
Loxodonta.africana                T---DDKHLKFQPHFGQQECVSPYRSR-G-ANGSETNRVGSSHGINQN-VNPSLCQEDDY    1135
Trichechus.manatus.latirostris    T---DDKHLKFQPHFGQQECVSPYRSR-G-PNGSETNRVGSNHGINQN-VNQSLCQEDDY    1135
Otolemur.garnettii                T---DDKHLKFQPHFGQQDCVSPYRSR-G-ASGSETNRVGSNHGINQN-VSQSLCQEDDY    1135
Callithrix.jacchus                T---DDKHLKFQPHFGQQECVSPYRSR-G-ANGSETNRVGSNHGINQN-VSQSLCQEDDY    1135
Macaca.mulatta                    T---DDKHLKFQPHFGQQECVSPYRSR-G-ANGSETNRVGSNHGINQN-VSQSLCQEDDY    1135
Nomascus.leucogenys               T---DDKHLKFQPHFGQQECVSPYRSR-G-ANGSETNRVGSNHGINQN-VSQSLCQEDDY    1135
Pongo.abelii                      T---DDKHLKFQPHFGQQECVSPYRSR-G-ANGSETNRVGSNHGINQN-VSQSLCQEDDY    1135
Homo.sapiens                      T---DDKHLKFQPHFGQQECVSPYRSR-G-ANGSETNRVGSNHGINQN-VSQSLCQEDDY    1135
Gorilla.gorilla                   T---DDKHLKFQPHFGQQECVSPYRSR-G-ANGSETNRVGSNHGINQN-VSQSLCQEDDY    1135
Pan.troglodytes                   T---DDKHLKFQPHFGQQECVSPYRSR-G-ANGSETNRVGSNHGINQN-VSQSLCQEDDY    1135
Pan.paniscus                      T---DDKHLKFQPHFGQQECVSPYRSR-G-ANGSETNRVGSNHGINQN-VSQSLCQEDDY    1135
Canis.lupus.familiaris            T---DDKHLKFQPHFGQQECVSPYRSR-G-ASGSETNRVGSNHGINQN-VNQSLCQEDDY    1135
Bos.taurus                        T---DDKHLKFQPHFGQQECVSPYRSR-A-ANGSETNRVGSNHGISQN-VNQSLCQEDDY    1135
Ovis.aries                        S---DDKHLKFQPHFGQQECVSPYRSR-A-ANGSETNRVGSNHGISQN-VTQSLCQEDDY    1135
Orcinus.orca                      T---DDKHLKFQPHFGQQECVSPYRSR-A-ANGSETNRVGSNHGINQN-VNQSLCQEDDY    1135
Sus.scrofa                        T---DDKHLKFQPHFGQQECVSPYRSR-A-ANGSEANRVGSNHGINQN-VNQSLCQEDDY    1135
Ceratotherium.simum.simum         T---DDKHLKFQPHFGQQECVSPYRSR-G-ANGSETNRVGSNHGINQN-VNQSLCQEDDY    1135
Equus.caballus                    T---DDKHLKFQPHFGQQECVSPYRSR-G-ANGSETNRVGSNHGINQN-VSQSLCQEDDY    1135
Felis.catus                       T---DDKHLKFQPHFGQQECVSPYRSR-G-ANGSETNRVSSNHGINQN-VNQSLCQEDDY    1135
Ailuropoda.melanoleuca            T---DDKHLKFQPHFGQQECVSPYRSG-G-ASGSETNRVGSNHGINQN-VNQSLCQEDDY    1135
Mustela.putorius.furo             T---DDKHLKFQPHFGQQECVSPYRSR-G-ASGSETNRVGSNHGINQN-VNQSLCQEDDY    1135
Odobenus.rosmarus.divergens       T---DDKHLKFQPHFGQQECVSPYRSR-G-ANGSETNRVGSNHGINQN-VNQSLCQEDDY    1135
Taeniopygia.guttata               G---EDKHMKYQTPFGQQECVSSFRSR-G-SSGSDQNRVGPTLGMNQK-VNQSLCQVDDY    1136
Anolis.carolinensis               G---DNKHLSYPSAFGQPECVSPFRSR-D-SSTSEQNRTGCSHGINQK-VNQSLRQVDDY    1134
Gallus.gallus                     G---DDKHMKYQSPFGQQDCVPSFRSR-G-SNGSDQNRVGSTLGINQK-VNQSLCQVDDY    1146
                                                                 .

Ciona.intestinalis                -----------LYADEESFD-------------------AEETPTNFGAIYREE------    1002
Strongylocentrotus.purpuratus     ---IDPMQGSSMYSNMNDHE------------PFAE---EDERPTDFSQRYANDMSHGDD    1121
Danio.rerio                       AD-DKPTNYSERYSEEEQLEE---QTPSYSMK-Y-TEDHHVEQPIDYSLKYSEA---PSK    1162
Xenopus.tropicalis                DD-DKTTNFSERYSEGEQQEDENERQNKYNIKPYASDEHHGEQPIDYSRKYPTDVPSSAQ    1196
Xenopus.laevis                    DD-DKTTNFSERYSEEEQQEDETERQNKYNIKAYASEEHHGEQPIDYSRKYSTDVPSSAQ    1196
```

```
Ornithorhynchus.anatinus            DDDDKPTNYSERYSEEEQHE--EDRPTNYSIK-YSEETHHTDQPIDYSLKYASDVPPSSQ    1196
Monodelphis.domestica               DE-DKPTNYSERYSEEEQHEE-EDRPTNYSMK-YNEEEHHADQPIDYSLKYAADITPSSQ    1192
Mus.musculus                        ED-DKPTNYSERYSEEEQHEEEEERPTNYSIK-YNEEKHHVDQPIDYSLKYATDI-SSSQ    1190
Rattus.norvegicus                   ED-DKPTNYSERYSEEEQHEE-EERPTNYSIK-YNEEKHHVDQPIDYSLKYATDI-SSSQ    1189
Sorex.araneus                       ED-DKPTNYSERYSEEEQREE-EERATNYSIK-YNEEKHHVDQPIDYSLKYTTDI-PSSQ    1190
Octodon.degus                       ED-DKPTNYSERYSEEEQHE--EERPTNYSIK-YNEEKHHVDQPIDYSLKYATDI-TSSQ    1190
Heterocephalus                      ED-DKPTNYSERYSEEEQHE--EERPTNYSIK-YNEEKHHVDQPIDYSLKYATDI-SSSQ    1188
Jaculus.jaculus                     ED-DKPTNYSERYSEEEQPEE-EERPTNFSIK-YSEEK-HVDQPIDYSLKYTSDI-SSSQ    1190
Loxodonta.africana                  ED-DKPTNYSERYSEEEQHE--EERPTNYSIK-YNEEEHHVDQPIDYSLKYASDI-PAPQ    1190
Trichechus.manatus.latirostris      ED-DKPTNYSERYSEEEQHEE-EERPTNYSIK-YNEEKHHVDQPIDYSLKYATDI-PPSQ    1191
Otolemur.garnettii                  ED-DKPTNYSERYSEEEQHEE-EERPTNYSIK-YNEEKHLVDQPIDYSLKYATDI-SSSQ    1191
Callithrix.jacchus                  ED-DKPTNYSERYSEEEQHEE-EERPTNYSIK-YNEEKHHVDQPIDYSLKYSTDI-PSSQ    1191
Macaca.mulatta                      ED-DKPTNYSERYSEEEQHEE-EERPTNYSIK-YNEEKHHVDQPIDYSLKYATDI-PSSQ    1191
Nomascus.leucogenys                 ED-DKPTNYSERYSEEEQHEE-EERPTNYSIK-YNEEKHHVDQPIDYSLKYATDI-PSSQ    1191
Pongo.abelii                        ED-DKPTNYSERYSEEEQHEE-EERPTNYSIK-YNEEKHHVDQPIDYSLKYTTDI-PSSQ    1191
Homo.sapiens                        ED-DKPTNYSERYSEEEQHEE-EERPTNYSIK-YNEEKRHVDQPIDYSLKYATDI-PSSQ    1191
Gorilla.gorilla                     ED-DKPTNYSERYSEEEQHEE-EERPTNYSIK-YNEEKHHVDQPIDYSLKYATDI-PSSQ    1191
Pan.troglodytes                     ED-DKPTNYSERYSEEEQHEE-EERPTNYSIK-YNEEKHHVDQPIDYSLKYATDI-PSSQ    1191
Pan.paniscus                        ED-DKPTNYSERYSEEEQHEE-EERPTNYSIK-YNEEKHHVDQPIDYSLKYATDI-PSSQ    1191
Canis.lupus.familiaris              ED-DKPTNYSERYSEEEQHEE-EERPTNYSIK-YNEEKHHVDQPIDYSLKYATDI-PSSQ    1191
Bos.taurus                          ED-DKPTNYSERYSEEGQHEE-EERPTNYSIK-YSEEKHHVDQPIDYSLKYTTDI-PSSQ    1191
Ovis.aries                          ED-DKPTNYSERYSEEGQHEE-EERPTNYSIK-YSEEKHHVDQPIDYSLKYTTDI-PSSQ    1191
Orcinus.orca                        ED-DKPTNYSERYSEEEQHEE-EERPTNYSIK-YNEEKHHVDQPIDYSLKYTTDI-PSSQ    1191
Sus.scrofa                          ED-DKPTNYSERYSEEEQHEE-EERPTNYSIK-YNEEKHHVDQPIDYSLKYATDI-PSSQ    1191
Ceratotherium.simum.simum           ED-DKPTNYSERYSEEEQHEE-EERPTNYSIK-YNEEKHHVDQPIDYSLKYTTDI-PSSQ    1191
Equus.caballus                      ED-DKPTNYSERYSEEEQHEE-EERPTNYSIK-YNEEKHHVDQPIDYSLKYTTDI-PSSQ    1191
Felis.catus                         ED-DKPTNYSERYSEEEQHEE-EERPTNYSIK-YNEEKHHVDQPIDYSLKYATDI-PASQ    1191
Ailuropoda.melanoleuca              ED-DKPTNYSERYSEEEQHEE-EERPTNYSIK-YNEEKHHVDQPIDYSLKYATDI-TSSQ    1191
Mustela.putorius.furo               ED-DKPTNYSERYSEEEQHEE-EDRPTNYSIK-YNEEKHHVDQPIDYSLKYATDI-PSSQ    1191
Odobenus.rosmarus.divergens         ED-DKPTNYSERYSEEEQHEE-EERPTNYSIK-YNEEKHHVDQPIDYSLKYATDI-PSSQ    1191
Taeniopygia.guttata                 DD-DKPTNYSERYSEEEQHEE-EDRPTNYSIK-YNEEEHHVDQPIDYSLKYSTEVPAPSQ    1193
Anolis.carolinensis                 ED-DKPTNYSERYSEEEEHEE-EDHPTNYSIK-YNEEEDHVDQPIDYSLKYSTDVPSSTQ    1191
Gallus.gallus                       DD-DKPTNYSERYSEEEQHEE-EDRPTNYSIK-YNEEEHQVDQPIDYSLKYSTEVPPSSQ    1203
                                                *::  . :                     : * ::.  *

Ciona.intestinalis                  ---------------------------------------QED------------------    1005
Strongylocentrotus.purpuratus       AESFGFVQTSLETNTGGTVYMSSEEPDVRDSSYNASSNQEIPSSVVSQFGSEHGGSRHSI    1181
Danio.rerio                         KGMFSHSKTSSAQSSAKEHLSQDSSSSVASLKNQGRQKQLHPSSAQSRSGPTR-------    1215
Xenopus.tropicalis                  KPSFPYSNNPSKQKPKKEQVSSSS-NTPTPSPNSGRQNQLHPNSAQTRPGLNR-------    1248
Xenopus.laevis                      KPSFPYSNNSSKQKPKKEQVSSNS-NTPTPSPNSNRQNQLHPNSAQSRPGLNR-------    1248
Ornithorhynchus.anatinus            KPSFSFSKSSSVQSAKAEHISTSNGNVSTSSAGAKRQGQLHPSSVPGRGGQTQ-------    1249
Monodelphis.domestica               KPSFSFSKSSSVQSNKTGHISSGG-NTSTAPASTKRQNQLLPSSAQNRSGHTQ-------    1244
Mus.musculus                        KPSFSFSKNSSAQSTKPEHLSPSSENTAVPPSNAKRQNQLRPSSAQR-NGQTQ-------    1242
Rattus.norvegicus                   KPSFSFSKTPSVQGTKTEHNSPSSEAASAPSSNAKRQSQLHPSSAQR-NGQTP-------    1241
Sorex.araneus                       KPSFSFSKSPSAPNPNPEHVSSSTESAATPSSRTQRQNPLHPGSAQSRSGHTQ-------    1243
Octodon.degus                       KPSFSFPKSSSAQSSKTEHVSSSSENAATPSPNAKRQAQLHPSSAHSRSGQTQ-------    1243
Heterocephalus                      KPSFTFSKSSSVQSSKTEHISSSSETTSTPSSNTKRQNQVHPNSAQSISGQTQ-------    1241
Jaculus.jaculus                     KPSFSFSKSSSTQSTKPEHISSSSENVSTPLSNAKRQNQLHPSSAQGRSGQAQ-------    1243
Loxodonta.africana                  KPSFSFSKSSSGQSTKTEHISSSSENASPPSSNAKRQNQLHPSSAQSRSGQAQ-------    1243
Trichechus.manatus.latirostris      KPSFSFSKSSSGQSTKTDHISSGSENASTPSSNAKRQNHLHPSSAQTRSGQTQ-------    1244
Otolemur.garnettii                  KQSFSFSKSSSGPSAKTEHIPSSSENSSTPSSNSKRQNQLHPSSTQSRSAQTQ-------    1244
Callithrix.jacchus                  KQSFSFSKSSSGQSTKTEHISSSSENTSTPSSNAKRQNQLHPSSAQNRSGQTQ-------    1244
Macaca.mulatta                      KQSFSFSKSSSGQSTKTEHISSSSENTSTPSSNAKRQNQLHPSSAQSRSGQTQ-------    1244
Nomascus.leucogenys                 KQSFSFSKSSSGQSTKTEHMSSSSENTSTPSSNAKRQNQLHPSSAQSRSGQTQ-------    1244
Pongo.abelii                        KQSFSFSKSSSGQSTKTEHMSSSSENTSTPSSNAKRQNQLHPSSAQSRSGQTQ-------    1244
Homo.sapiens                        KQSFSFSKSSSGQSSKTEHMSSSSENTSTPSSNAKRQNQLHPSSAQSRSGQPQ-------    1244
Gorilla.gorilla                     KQSFSFSKSSSGQSSKTEHMSSSSENTSTPSSNAKRQNQLHPSSAQSRSGQTQ-------    1244
Pan.troglodytes                     KQSFSFSKSSSGQSSKTEHMSSSSENTSTPSSNAKRQNQLHPSSAQSRSGQTQ-------    1244
Pan.paniscus                        KQSFSFSKSSSGQSSKTEHMSSSSENTSTPSSNAKRQNQLHPSSAQSRSGQTQ-------    1244
Canis.lupus.familiaris              KPPFSFSKNSSGQSTKTEHLSASSENTSTPSSSAKRQTQHHPSSAQSRNGQTP-------    1244
Bos.taurus                          KPAFSFSKNSSGQSTKTEHISSSSENTSTTSSNAKRQNQLHPSSAQSRSGQTP-------    1244
Ovis.aries                          KPAFSFSKNSSGQSTKTEHISSSSENTSTTSSNAKRQNQLHPSSAQSRSGQTP-------    1244
Orcinus.orca                        KPAFSFSKNSSGQSTKTEHISSSSENTATPSSNAKRQNQLHHSSAQSRSGQTQ-------    1244
Sus.scrofa                          KPAFSFSKNSSGQSTKTERISPSGENTSTPSSNAKRQSQLHPSSAQSRSGQTP-------    1244
Ceratotherium.simum.simum           KPSFSFSKSSSGQSTKTEHISSSSENTSTPSSNAKRQNQLHPSSAQSRSGQTQ-------    1244
```

```
Equus.caballus                    KPSFSFSKSSSGQSTKTEHISSSSENTSTPSSNAQRQSQLHPSSAQGRSGQTQ-------    1244
Felis.catus                       KPPFSFSKSSSGQSTKTEHISSSSENTSTPSSNAKRQNQHHPSSTQSRNGQTP-------    1244
Ailuropoda.melanoleuca            KPPFSFSKNPSGQSTKTEHISSSSENTSTPSSNAKRQNQHHPSSAQSRNGQTP-------    1244
Mustela.putorius.furo             KPPFSFSKNPSGQSTKTEHISSSSENTSTPSSNAKRQNQHHPSSAQNRNGQTS-------    1244
Odobenus.rosmarus.divergens       KPPFSFSKNSSGQSTKTEHISSSSENTSTPSSNAKRQNQHHPSSAQSRNGQTP-------    1244
Taeniopygia.guttata               KPSFTFPKTSSVPLNKTDHIPPSSGSTSAPSSGSKRQNQLHPSSAQSRSGHAQ-------    1246
Anolis.carolinensis               KPSFSFSKSPSVQSSKTDHISSSSGNTS-TSAGSKRHHQLHPNSAQGRTGHAQ-------    1243
Gallus.gallus                     KPSFTFSKTSSVQSTKTDHISSSSGNTSAPSAGSKRQNQLHPSSAQSRGGHAQ-------    1256

Ciona.intestinalis                --PLFRDAGDENAINQDQPKEYKVEDTPACFTPRSAISDLPCEEEDDQNIHGDQNAQ---    1060
Strongylocentrotus.purpuratus     ASSHHEDEEEPPCSHDDNTKTYCVEGTPGPISRCSSLSSLDLNEELDEVEKTKDDSAHSP    1241
Danio.rerio                       -AVQKNPTCKAPTINQETLQTYCVEDTPICFSRGSSLSSLSSEEDEMESCKRNVNSA-SN    1273
Xenopus.tropicalis                --P-KQTSSKPPSITQETIQTYCVEDTPICFSRGSSLSSLSSAEDEIEGRERSSRGQ-ES    1304
Xenopus.laevis                    --P-KQIPNKPPSINQETIQTYCVEDTPICFSRGSSLSSLSSAEDEIEGRERNSRGQ-ES    1304
Ornithorhynchus.anatinus          -KA---ASCKAPSINQETIQTYCVEDTPICFSRCSSLSSLSSAEDEI-GRDQVTRST-DP    1303
Monodelphis.domestica             -KT---ASCKAPSINQETIQTYCVEDTPICFSRCSSLSSLSSAEDEI-GRDQTTRGS-ET    1298
Mus.musculus                      -K---GTTCKVPSINQETIQTYCVEDTPICFSRCSSLSSLSSADDEI-GCDQTTQEA-DS    1296
Rattus.norvegicus                 -K---GTACKVPSINQETMQTYCVEDTPICFSRCSSLSSLSSAEDEI-GCDQTTQEA-DS    1295
Sorex.araneus                     -KA---ASCKVPSINQETIQTYCVEDTPICFSRCSSLSSLSSAEDEI-GCNQTTQPT-DS    1297
Octodon.degus                     -KT---TTCKVPSINQETIQTYCVEDTPICFSRCSSLSSLSSAEDEI-GCAQTTQEV-DS    1297
Heterocephalus                    -KA---TTCKVPSINQETIQTYCVEDTPICFSRCSSLSSLSSAEDET-GCDQTTQEA-DS    1295
Jaculus.jaculus                   -KS---ATCKVPSINQETIQTYCVEDTPICFSRCSSLSSLSSAEDEM-GCDQATQEA-DS    1297
Loxodonta.africana                -KA---TSCKVPSINQETIQTYCVEDTPICFSRCSSLSSLSSAEDEI-GCDQTTQET-DS    1297
Trichechus.manatus.latirostris    -KA---ASCKVPSINQETIQTYCVEDTPICFSRCSSLSSLSSAEDEI-GCDQTTQET-DS    1298
Otolemur.garnettii                -KA---TSCKVSSINQETIQTYCVEDTPICFSRCSSLSSLSSADDEI-GCDQTTQEA-DS    1298
Callithrix.jacchus                -KA---ATCKVSSINQETIQTYCVEDTPICFSRCSSLSSLSSAEDEI-GCDQTTQEA-DS    1298
Macaca.mulatta                    -KA---ATCKVSSINQETIQTYCVEDTPICFSRCSSLSSLSSAEDEI-GCDQTTQEA-DS    1298
Nomascus.leucogenys               -KA---ATCKVSSINQETIQTYCVEDTPICFSRCSSLSSLSSAEDEI-GCDQRTQEA-DS    1298
Pongo.abelii                      -KA---ATCKVSSINQETIQTYCVEDTPICFSRCSSLSSLSSAEDEI-GCGQTTQEA-DS    1298
Homo.sapiens                      -KA---ATCKVSSINQETIQTYCVEDTPICFSRCSSLSSLSSAEDEI-GCNQTTQEA-DS    1298
Gorilla.gorilla                   -KA---ATCKVSSINQETIQTYCVEDTPICFSRCSSLSSLSSAEDEI-GCNQTTQEA-DS    1298
Pan.troglodytes                   -KA---ATCKVSSINQETIQTYCVEDTPICFSRCSSLSSLSSAEDEI-GCNQTTQEA-DS    1298
Pan.paniscus                      -KA---ATCKVSSINQETIQTYCVEDTPICFSRCSSLSSLSSAEDEI-GCNQTTQEA-DS    1298
Canis.lupus.familiaris            -KA---NSCKVPSINQETIQTYCVEDTPICFSRCSSLSSLSSAEDEI-GCDPAPQEA-ES    1298
Bos.taurus                        -KA-TSSSCKVPSINQETIQTYCVEDTPICFSRCSSLSSLSSAEDEV-GCDQTTQEA-ES    1300
Ovis.aries                        -KA-TSSSCKVPSINQETIQTYCVEDTPICFSRCSSLSSLSSAEDEV-GCDQTTQEA-ES    1300
Orcinus.orca                      -KA-TSSSCKVPSINQETIQTYCVEDTPICFSRCSSLSSLSSAEDEI-GCDQTTQEA-DS    1300
Sus.scrofa                        -KA-TSSSCKVPSINQETIQTYCVEDTPICFSRCSSLSSLSSAEDEI-GCDQQTQET-DS    1300
Ceratotherium.simum.simum         -KA---TSCKVPSINQETIQTYCVEDTPICFSRCSSLSSLSSAEDEI-GCDQTTQET-DS    1298
Equus.caballus                    -KA---ASCKVPSINQETIQTYCVEDTPICFSRCSSLSSLSSAEDEI-GCEQTTQET-ES    1298
Felis.catus                       -KA---TSCKVPSINQETIQTYCVEDTPICFSRCSSLSSLSSAEDEI-GCDQTTQET-DS    1298
Ailuropoda.melanoleuca            -KA---TSCKVPSINQETIQTYCVEDTPICFSRCSSLSSLSSAEDEI-GCDQATQET-DS    1298
Mustela.putorius.furo             -KA---TSCKVPSINQETIQTYCVEDTPICFSRCSSLSSLSSAEDEI-GCDQATQET-DS    1298
Odobenus.rosmarus.divergens       -KA---TSCKVPSINQETIQTYCVEDTPICFSRCSSLSSLSSAEDEI-GCDQATQET-DS    1298
Taeniopygia.guttata               -KN---TSCKAPSINQETIQTYCVEDTPICFSRCSSLSSLSSAEDEI-GRDQSTRGT-DA    1300
Anolis.carolinensis               -KP---TSSKAPSINQETIQTYCVEDTPICFSRCSSLSSLSSAEDET-GRDQATRVA-DA    1297
Gallus.gallus                     -KT---ASCKTPSINQETIQTYCVEDTPICFSRCSSLSSLSSAEDEI-GRDQSTRVT-DT    1310
                                           .     ::  : * **.**   :: *::*.*   ::

Ciona.intestinalis                --------KKNEQ------------------------------AYHNNVEDDKVDLQPG    1081
Strongylocentrotus.purpuratus     SNDLQSTTPPNEP------------------------------TP-EKVTNKQVL----    1265
Danio.rerio                       YPTLPISEKQSTN-NVAADQRTSESQSSVHYVRAKPPRHHLG--HGDGSRHHKTVEFSSG    1330
Xenopus.tropicalis                NNTLQITEPKENKSAVSKDGAINETRSSVHHTRTKNNRLQTSNISPSDSSRHKSVEFSSG    1364
Xenopus.laevis                    NNTLQITEPKEI-SAVSKDGAVNETRSSVHHTRTKNNRLQTSNISPSDSSRHKSVEFSSG    1363
Ornithorhynchus.anatinus          ASTLQITELKESAGAGSTEDAASEVPSASQHMRTKSSRLQTSNISPSDSSRHKAVEFSSG    1363
Monodelphis.domestica             TNTLQIAELKENSASVSTGDTGSEVPSTSQHIRTKANRLQTTSLSPSDSTRHKAVEFSSG    1358
Mus.musculus                      ANTLQTAEVKENDVTRSAEDPATEVPAVSQNARAKPSRLQASGLSSESTRHNKAVEFSSG    1356
Rattus.norvegicus                 ANTLQIAEIKENDVTRSAQDPASDVPAVSQSTRTKPSRLQASGLASESARH-KAVEFSSG    1354
Sorex.araneus                     ANTLQIAEIKENSGTRSTEDSVTEVSTVSQHIRAKSSRLQTSGLSA-ESTRHKAVEFSSG    1356
Octodon.degus                     ADTLKIAEIKESNVNRAAEDPANEVPAASQHVRAKSSRLQPSSLSS-ESTRHKAVEFSSG    1356
Heterocephalus                    AGTLKIAEIKESSVTRATEDPANEVSTASQHVRTKSSRLQASGLSS-ESTRHKAVEFSSG    1354
Jaculus.jaculus                   ATTLQIAEIKESSVAGSVEDPVSEVPAASQHARAKSSRLQASGLSS-ESARHKAVEFSSG    1356
Loxodonta.africana                ANTLQIAELKENSGTRSTEETVSEVPAVSQHIRTKSNRLQASGLSSSESTRHKAVEFSSG    1357
Trichechus.manatus.latirostris    ANTLQIAELKENGGTRSNEETVSEVPAVSQHIRTKSNRLQASGLSSSESARHKAVEFSSG    1358
```

```
Otolemur.garnettii                  ANTLQIAEIKENSGPRSAEDPVSEVPAVSQHIRSKSNRLQASGLSS-ESTRQKAVEFSSG    1357
Callithrix.jacchus                  ANTLQIAEIKENNGTRSTEDPVSEVPAVSQHTRTKSSRLQGSSLSS-ESTRHKAVEFSSG    1357
Macaca.mulatta                      ANTLQIAEIKDKIGTRSTEDPVSEVPAVSQHTRTKSSRLQGSSLSS-ESTRHKAVEFSSG    1357
Nomascus.leucogenys                 ANTLQVVEIKEKIGTRSTEDPVSEVPAVSQHTRTKSSRLQCSSLSS-ESARHKAVEFSSG    1357
Pongo.abelii                        ANTLQIAEIKEKIVTRSTEDPVSEVPAVSQHTRTKSSRLQGSNLSS-ESARHKAVEFSSG    1357
Homo.sapiens                        ANTLQIAEIKEKIGTRSAEDPVSEVPAVSQHPRTKSSRLQGSSLSS-ESARHKAVEFSSG    1357
Gorilla.gorilla                     ANTLQIAEIKEKIGTRSAEDPVSEVPAVSQHTRTKSSRLQGSSLSS-ESARHKAVEFSSG    1357
Pan.troglodytes                     ANTLQIAEIKEKIGTRSAEDPVSEVPAVSQHTRTKSSRLQGSSLSS-ESARHKAVEFSSG    1357
Pan.paniscus                        ANTLQIAEIKEKIGTRSAEDPVSEVPAVSQHTRTKSSRLQGSSLSS-ESARHKAVEFSSG    1357
Canis.lupus.familiaris              ANTLQRAEIKENSGTRTTEDAVSEVPAASPHIRTKSSRLQASGLSA-ESTRHKAVEFSSG    1357
Bos.taurus                          ANTLQIAEIKDNSGPRSNEDSVSKVPAGSQHIRTKSSRLQASGLSS-ESARHKAVEFSSG    1359
Ovis.aries                          ANTLQIAEIKDNSGPRSNEDSVSKVPTVSQHIRTKSSRLQASGLSS-ESARHKAVEFSSG    1359
Orcinus.orca                        ANTLQIAEIKESSGTRSTEDSVSEVPTVSQHIRTKSSRLQASGLSS-ESTRHKAVEFSSG    1359
Sus.scrofa                          ANTLQIAEIKENSGTRSTEESVSEVPTVPQHIRTKSSRLQASGLSS-ESTRHKAVEFSSG    1359
Ceratotherium.simum.simum           ANTLQIAEIKENNGTRSTEDSVSEVPTVSQHVRTKSSRLQASGLSS-ESTRHKAVEFSSG    1357
Equus.caballus                      ASTLQIAEIKESSGTRSTEDSVSEVPAVSQHVRTKSSRLQASGLSS-ESTRHKAVEFSSG    1357
Felis.catus                         ANTLQIAEIKENSGTRSTEESVSEVPTVSQHIRTKSSRLQASGLSA-ESTRHKAVEFSSG    1357
Ailuropoda.melanoleuca              ANTLQIAEIKENSGTRTTEDSVSEVPTVSQHIRTKSSRLQASGLSA-ESTRHKAVEFSSG    1357
Mustela.putorius.furo               ANTLQIAEIKENSGTRTTEDSVSEVPTVSQHIRTKSSRLQASGLPA-ESARHKAVEFSSG    1357
Odobenus.rosmarus.divergens         ANTLQIAEIKENSGTRTTEDSVSEVPTVSQHIRTKSSRLQASGLSS-ESTRQKAVEFSSG    1357
Taeniopygia.guttata                 NNTLQIAELKKENSGALPTEGAASEITSTAQHIRTKSTRLQTSSLSPSDSSRHKAVEFSSG   1360
Anolis.carolinensis                 DSSLKIGRIKENSGNLSAAGTVIETSSATQHIRTKSNRLQASSLSPSDSARHKTVEFSSG    1357
Gallus.gallus                       NATLQISELKENSGALSAEAAVSEITSTSQHIRTKSSRLPTSSLSPSESSRHKAVEFSSG    1370
                                              .                                         . *

Ciona.intestinalis                  AMTPAFSTRGRKSGTMTPKGYQETPMMFSRCSSMCSLSSFEAPSVQSQVESEP--SRFCS    1139
Strongylocentrotus.purpuratus       --------LQLPSDVHHHDEVQETPLVFSRCSSVCSLSSDDVPDICDDVSSIYTNSRAAS    1317
Danio.rerio                         AKSPSKSGAQTP-KSPPEHYVQETPLMFSRCTSVSSLDSFESHSIASSVQSEP-CSGMVS    1388
Xenopus.tropicalis                  AKSPSKSGAQTP-KSPPEHYVQETPLMFSRCTSVSSLDSFESHSIASSIASSVASEHMIS    1423
Xenopus.laevis                      AKSPSKSGAQTP-KSPPEHYVQETPLMFSRCTSGSSLDSFESHSIASSIASSVASEHMIS    1422
Ornithorhynchus.anatinus            AKSPSKSGAQTP-KSPPEHYVQETPLMFSRCTSVSSLDSFESRSIASSVQSEP-CSGMVS    1421
Monodelphis.domestica               AKSPAKSGAQTP-KSPPEHYVQETPLMFSRCTSVSSLDSFESRSIASSVQSEP-CSGMVS    1416
Mus.musculus                        AKSPSKSGAQTP-KSPPEHYVQETPLVFSRCTSVSSLDSFESRSIASSVQSEP-CSGMVS    1414
Rattus.norvegicus                   AKSPSKSGAQTP-KSPPEHYVQETPLVFSRCTSVSSLDSFESRSIASSVQSEP-CSGMVS    1412
Sorex.araneus                       AKSPSKSGAQTP-KSPPEHYVQETPLMFSRCTSVSSLDSFESRSIASSVQSEP-CSGMVS    1414
Octodon.degus                       AKSPSKSGAQTP-KSPPEHYVQETPLMFSRCTSVSSLDSFESRSIASSVQSEP-CSGMVS    1414
Heterocephalus                      AKSPSKSGAQTP-KSPPEHYVQETPLMFSRCTSVSSLDSFESRSIASSVQSEP-CSGMVS    1412
Jaculus.jaculus                     AKSPSKSGAQTP-KSPPEHYVQETPLMFSRCTSVSSLDSFESRSIASSVQSEP-CSGMVS    1414
Loxodonta.africana                  AKSPSKSGAQTP-KSPPEHYVQETPLMFSRCTSVSSLDSFESRSIASSVQSEP-CSGMIS    1415
Trichechus.manatus.latirostris      AKSPSKSGAQTP-KSPPEHYVQETPLMFSRCTSVSSLDSFESRSIASSVQSEP-CSGMIS    1416
Otolemur.garnettii                  AKSPSKSGAQTP-KSPPEHYVQETPLMFSRCTSVSSLDSFESRSIASSVQSEP-CSGMVS    1415
Callithrix.jacchus                  AKSPSKSGAQTP-KSPPEHYVQETPLMFSRCTSVSSLDSFESRSIASSVQSEP-CSGMVS    1415
Macaca.mulatta                      AKSPSKSGAQTP-KSPPEHYVQETPLMFSRCTSVSSLDSFESRSIASSVQSEP-CSGIVS    1415
Nomascus.leucogenys                 AKSPSKSGAQTP-KSPPEHYVQETPLMFSRCTSVSSLDSFESRSIASSVQSEP-CSGMVS    1415
Pongo.abelii                        AKSPSKSGAQTP-KSPPEHYVQETPLMFSRCTSVSSLDSFESRSIASSVQSEP-CSGMVS    1415
Homo.sapiens                        AKSPSKSGAQTP-KSPPEHYVQETPLMFSRCTSVSSLDSFESRSIASSVQSEP-CSGMVS    1415
Gorilla.gorilla                     AKSPSKSGAQTP-KSPPEHYVQETPLMFSRCTSVSSLDSFESRSIASSVQSEP-CSGMVS    1415
Pan.troglodytes                     AKSPSKSGAQTP-KSPPEHYVQETPLMFSRCTSVSSLDSFESRSIASSVQSEP-CSGMVS    1415
Pan.paniscus                        AKSPSKSGAQTP-KSPPEHYVQETPLMFSRCTSVSSLDSFESRSIASSVQSEP-CSGMVS    1415
Canis.lupus.familiaris              AKSPSKSGAQTP-KSPPEHYVQETPLMFSRCTSVSSLDSFESRSIASSVQSEP-CSGMVS    1415
Bos.taurus                          AKSPSKSGAQTP-KSPPEHYVQETPLMFSRCTSVSSLDSFESRSIASSVQSEP-CSGMVS    1417
Ovis.aries                          AKSPSKSGAQTP-KSPPEHYVQETPLMFSRCTSVSSLDSFESRSIASSVQSEP-CSGMVS    1417
Orcinus.orca                        AKSPSKSGAQTP-KSPPEHYVQETPLMFSRCTSVSSLDSFESRSIASSVQSEP-CSGMVS    1417
Sus.scrofa                          AKSPSKSGAQTP-KSPPEHYVQETPLMFSRCTSVSSLDSFESRSIASSVQSEP-CSGMVS    1417
Ceratotherium.simum.simum           AKSPSKSGAQTP-KSPPEHYVQETPLMFSRCTSVSSLDSFESRSIASSVQSEP-CSGMVS    1415
Equus.caballus                      AKSPSKSGAQTP-KSPPEHYVQETPLMFSRCTSVSSLDSFESRSIASSVQSEP-CSGMVS    1415
Felis.catus                         AKSPSKSGAQTP-KSPPEHYVQETPLMFSRCTSVSSLDSFESRSIASSVQSEP-CSGMVS    1415
Ailuropoda.melanoleuca              AKSPSKSGAQTP-KSPPEQYVQETPLMFSRCTSVSSLDSFESRSIASSVQSEP-CSGMVS    1415
Mustela.putorius.furo               AKSPSKSGAQTP-KSPPEHYVQETPLMFSRCTSVSSLDSFESRSIASSVQSEP-CSGMVS    1415
Odobenus.rosmarus.divergens         AKSPSKSGAQTP-KSPPEHYVQETPLMFSRCTSVSSLDSFESRSIASSVQSEP-CSGMVS    1415
Taeniopygia.guttata                 AKSPSKSGAQTP-KSPPEHYVQETPLMFSRCTSVSSLDSFESRSIASSVQSEP-CSGIVS    1418
Anolis.carolinensis                 AKSPSKSGAQTP-KSPPEHYVQETPLMFSRCTSVSSLDSFESRSIASSVQSEP-CSGMVS    1415
Gallus.gallus                       AKSPSKSGAQTP-KSPPEHYVQETPLMFSRCTSVSSLDSFESRSIASSVQSEP-CSGMVS    1428
                                                      .  ****::****:* .**.* :  .: ..: *    .   *
```

```
Ciona.intestinalis                 GVISPSELPDSPGQSMPVSRSRSYSNLNDVLKDPSYISKPLPNAQPNVSNSEQKEEKKPQ    1199
Strongylocentrotus.purpuratus      GFVSPSELPDSPSDTMPPSPRRTSQS-----------NVKVDL-----------------    1349
Danio.rerio                        GIISPSDLPDSPGQTMPPSRSKTPPPPPPRS-----TSVKQKV--TVPPHTEKHD-LAPR    1440
Xenopus.tropicalis                 GIISPSDLPDSPGQTMPPSRSKTPP------P-PQTVHAKKDSSKPKVSG-EERD-KVAK    1474
Xenopus.laevis                     GIISPSDLPDSPGQTMPPSRSKTPP------P-PQTVQAKKDGSKPIVPD-EERG-KVAK    1473
Ornithorhynchus.anatinus           GIISPSDLPDSPGQTMPPSRSKTPP------P-PQVVQAKRDAAKSKGLHPDKRE-PGPR    1473
Monodelphis.domestica              GIISPSDLPDSPGQTMPPSRSKTPP------P-PQTGQTKREATKSKLPSAEKRE-SGPR    1468
Mus.musculus                       GIISPSDLPDSPGQTMPPSRSKTPPP-----P-PQTVQAKREVPKSKVPAAEKRE-SGPK    1467
Rattus.norvegicus                  GIVSPSDLPDSPGQTMPPSRSKTPPPP---PP-PQPVQTKREVPKTKVPAAEQRE-GGPK    1467
Sorex.araneus                      GIISPSDLPDSPGQTMPPSRSKTPP------P-PQIITNKQEVPQNKIPAAEK---PGPK    1464
Octodon.degus                      GIISPSDLPDSPGQTMPPSRSKTPP-----PP-PQTVQSKREVPKNKVTPVEKRE-SGPK    1467
Heterocephalus                     GIISPSDLPDSPGQTMPPSRSKTPP-----PP-PQTVQSKREVPKNKVPPVEKRE-NGPK    1465
Jaculus.jaculus                    GIISPSDLPDSPGQTMPPSRSKTPP-----PP-PQAAQTKREVPKNKVPSAEKRE-SVPK    1467
Loxodonta.africana                 GIISPSDLPDSPGQTMPPSRSKTPP-----PP-PQTVQTKREASKNKVPNAEKRE-SGPK    1468
Trichechus.manatus.latirostris     GIISPSDLPDSPGQTMPPSRSKTPP-----PP-PQTVQTKREASKNKVSNAEKRE-SGPK    1469
Otolemur.garnettii                 GIISPSDLPDSPGQTMPPSRSKTPP-----PP-PQTVQTKREVPKNKVPTAEKRE-SGPK    1468
Callithrix.jacchus                 GIISPSDLPDSPGQTMPPSRSKTPP-----PP-PQTAQTKREVPKSKAPSAEKRE-SGPK    1468
Macaca.mulatta                     GIISPSDLPDSPGQTMPPSRSKTPP-----PP-PQTAQTKREVPKNKTPTAEKRE-SGPK    1468
Nomascus.leucogenys                GIISPSDLPDSPGQTMPPSRSKTPP-----PP-PQTAQTKREVPKNKAPTAEKRE-SGPK    1468
Pongo.abelii                       GIISPSDLPDSPGQTMPPSRSKTPP-----PP-PQTAQTKREVPKNKAPTAEKRE-SGPK    1468
Homo.sapiens                       GIISPSDLPDSPGQTMPPSRSKTPP-----PP-PQTAQTKREVPKNKAPTAEKRE-SGPK    1468
Gorilla.gorilla                    GIISPSDLPDSPGQTMPPSRSKTPP-----PP-PQTAQTKREVPKNKAPTAEKRE-SGPK    1468
Pan.troglodytes                    GIISPSDLPDSPGQTMPPSRSKTPP-----PP-PQTAQTKREVPKNKAPTAEKRE-SGPK    1468
Pan.paniscus                       GIISPSDLPDSPGQTMPPSRSKTPP-----PP-PQTAQTKREVPKNKAPTAEKRE-SGPK    1468
Canis.lupus.familiaris             GIISPSDLPDSPGQTMPPSRSKTPP-----PP-PQTVQTKREVPKNKASAAEKRE-SGPK    1468
Bos.taurus                         GIISPSDLPDSPGQTMPPSRSKTPPPP-PPPP-PQTVQTKQEVPKNKAPSAEKRE-SGPK    1474
Ovis.aries                         GIISPSDLPDSPGQTMPPSRSKTPPPPPPPPP-PQTVQTKQEVPKNKAPSAEKRE-SGPK    1475
Orcinus.orca                       GIISPSDLPDSPGQTMPPSRSKTPPP----PP-PQSAQTKQEVPKNKAPSAEKRE-SGPK    1471
Sus.scrofa                         GIISPSDLPDSPGQTMPPSRSKTPPP----PP-PQTSQTKQEVPKSKAPSAEKRE-SGPK    1471
Ceratotherium.simum.simum          GIISPSDLPDSPGQTMPPSRSKTPPP----PP-PQTVQTKREVPKNKVPNAEKRE-SGPK    1469
Equus.caballus                     GIISPSDLPDSPGQTMPPSRSKTPPP----PP-PQTVQTKREVPKNKVPNAEKRE-SGPK    1469
Felis.catus                        GIISPSDLPDSPGQTMPPSRSKTPPP----PP-PQTVQSKREVPKNKAPTAEKRE-SGPK    1469
Ailuropoda.melanoleuca             GIISPSDLPDSPGQTMPPSRSKTPPP-----P-PQSVQTKREVPKNKAPTAEKRE-SGPK    1468
Mustela.putorius.furo              GIISPSDLPDSPGQTMPPSRSKTPPP-----P-PQTVQTKREVPKNKAPAVEKRE-SGPK    1468
Odobenus.rosmarus.divergens        GIISPSDLPDSPGQTMPPSRSKTPPP-----P-PQTVQTKREIPKNKAPTAEKRE-SGPK    1468
Taeniopygia.guttata                GIISPSDLPDSPGQTMPPSRSKTPP------P-AQGVQVKREGPKGKATTTEKRE-PGPR    1470
Anolis.carolinensis                GIISPSDLPDSPGQTMPPSRSKTPP------P-PPVTQVKRDAAKPKVSSTEKRE-SGPR    1467
Gallus.gallus                      GIISPSDLPDSPGQTMPPSRSKTPP------P-AQGVQVKRDVTKGKVPTAEKRE-PGPR    1480
                                   *.:***:*****.::** *  ::

Ciona.intestinalis                 ILQQLSRASEHVRAGYVTSLP-QNDEIKCYNSEPAM---SDMTAFSGLSIGGDKVAP---    1252
Strongylocentrotus.purpuratus      --------------LNAPKLPEMEEKPTMYATEGTPIDNSCATSLSALTIDGDV-KATVA    1394
Danio.rerio                        H------AVVSAAVQKVQVLP-DNDTLLHFATESTPDGFSCASSLSALSLDEPFIQKDVE    1493
Xenopus.tropicalis                 Q------TGVHSAIQRVQVLQ-EADTLLHFATESTPDGFSCASSLSALSLDEPYIQKDVE    1527
Xenopus.laevis                     -------TAVHSAIQRVQVLQ-EADTLLHFATESTPDGFSCASSLSALSLDEPYIQKDVQ    1525
Ornithorhynchus.anatinus           L------AAVSAAVQRVQVLP-DADTLMHFATESTPDGFSCSSSLSALSLDEPYIQKDVE    1526
Monodelphis.domestica              Q------AAVNAAVQRVQVLP-DADTLLHFATESTPDGFSCSSSLSALSLDEPFIQKDVE    1521
Mus.musculus                       Q------TAVNAAVQRVQVLP-DVDTLLHFATESTPDGFSCSSSLSALSLDEPFIQKDVE    1520
Rattus.norvegicus                  Q------TAVSAAVQRVQVLP-DADTLLHFATESTPDGFSCSSSLSALSLDEPFIQKDVE    1520
Sorex.araneus                      Q------AAVHAAVQRVQVLP-DADTLMHFATESTPDGFSCSSSLSALSLDEPFIQKDVE    1517
Octodon.degus                      Q------AAVNAAVQRVQVLP-DADTLLHFATESTPDGFSCSSSLSALSLDEPFIQKDVE    1520
Heterocephalus                     Q------AAVNEAVQRVHVLP-DTDTLLHFATESTPDGFSCSSSLSALSLDEPFIQKDVE    1518
Jaculus.jaculus                    Q------AAVSAAVQRVQVLP-DTDTLLHFATESTPDGFSCSSSLSALSLDEPFIQKDVE    1520
Loxodonta.africana                 Q------AAINAAVQRVQVLP-DADTLLHFATESTPDGFSCSSSLSALSVDEPFIQKDVE    1521
Trichechus.manatus.latirostris     Q------AAVNAAVQRVQVLP-DADTLLHFATESTPDGFSCSSSLSALSLDEPFIQKDVE    1522
Otolemur.garnettii                 Q------AAINAAVQRVQVLP-DADTLLHFATESTPDGFSCSSSLSALSLDEPFIQKDVE    1521
Callithrix.jacchus                 Q------AAVNAAVQRVQVLP-DADTLLHFAMESTPDGFSCSSSLSALSLDEPFIQKDVE    1521
Macaca.mulatta                     Q------AAVNAAVQRVQVLP-DADTLLHFATESTPDGFSCSSSLSALSLDEPFIQKDVE    1521
Nomascus.leucogenys                Q------AAVNAAVQRVQVLP-DADTLLHFATESTPDGFSCSSSLSALSLDEPFIQKDVE    1521
Pongo.abelii                       Q------AAVNAAVQRVQVLP-DADTLLHFATESTPDGFSCSSSLSALSLDEPFIQKDVE    1521
Homo.sapiens                       Q------AAVNAAVQRVQVLP-DADTLLHFATESTPDGFSCSSSLSALSLDEPFIQKDVE    1521
Gorilla.gorilla                    Q------AAVNAAVQRVQVLP-DADTLLHFATESTPDGFSCSSSLSALSLDEPFIQKDVE    1521
Pan.troglodytes                    Q------AAVNAAVQRVQVLP-DADTLLHFATESTPDGFSCSSSLSALSLDEPFIQKDVE    1521
Pan.paniscus                       Q------AAVNAAVQRVQVLP-DADTLLHFATESTPDGFSCSSSLSALSLDEPFIQKDVE    1521
Canis.lupus.familiaris             Q------AAVNAAVQRVQVLP-DADTLLHFATESTPDGFSCSSSLSALSLDEPFIQKDVE    1521
```

```
Bos.taurus                            Q------AAVNAAVQRVQVLP-EADTLLHFATESTPDGFSCSSSLSALSLDEPFIQKDVE    1527
Ovis.aries                            Q------AAVNAAVQRVQVLP-DADTLLHFATESTPDGFSCSSSLSALSLDEPFIQKDVE    1528
Orcinus.orca                          Q------AAVNAAVQRVQVLP-DADALLHFATESTPDGFSCSSSLSALSLDEPFIQKDVE    1524
Sus.scrofa                            Q------AAVNAAVQRVQVLP-DADTLLHFATESTPDGFSCSSSLSALSLDEPFIQKDVE    1524
Ceratotherium.simum.simum             Q------AAVNAAVQRVQVLP-DADTLLHFATESTPDGFSCSSSLSALSLDEPFIQKDVE    1522
Equus.caballus                        Q------AAVNAAVQRVQVLP-DADTLLHFATESTPDGFSCSSSLSALSLDEPFIQKDVE    1522
Felis.catus                           Q------AAVNAAVQRVQVLP-DADTLLHFATESTPDGFSCSSSLSALSLDEPFIQKDVE    1522
Ailuropoda.melanoleuca                Q------AAVNAAVQRVQVLP-DADTLLHFATESTPDGFSCSSSLSALSLDEPFIQKDVE    1521
Mustela.putorius.furo                 Q------AAVNAAVQRVQVLP-DADTLLHFATESTPDGFSCSSSLSALSLDEPFIQKDVE    1521
Odobenus.rosmarus.divergens           Q------AAVNAAVQRVHVLP-DADTLLHFATESTPDGFSCSSSLSALSLDEPFIQKDVE    1521
Taeniopygia.guttata                   Q------AAVNAAVQRVQVLP-DADTLLHFATESTPDGFSCSSSLSALSLDEPFIQKDAE    1523
Anolis.carolinensis                   Q------AAVNTAVQRVQVLQ-DADTLLHFATESTPDGFSCSSSLSALSLDEPFIQKDVE    1520
Gallus.gallus                         Q------AAVNAAVQRVQVLP-DADTLLHFATESTPDGFSCSSSLSALSLDEPFIQKDVE    1533
                                                      .  *    :    :  * :    *  :::*.*::.

Ciona.intestinalis                    LGILKTSTDS-----SHPELEK--------------------AGNRF------VTQAGYI    1281
Strongylocentrotus.purpuratus         KRLLPPKGNEGSENVGSED-----KEITDEKNVEPS-------GNEFSEQEQSLLDECIN    1442
Danio.rerio                           LKIMPPVHEDDHSIEAEPEMEDMHEPKVQEKSPATSEAAKDILDDSDDDDDTEILNACIN    1553
Xenopus.tropicalis                    LKIMPPVLENDQGNEAEPEKES--TDNLNKKENKRSEQEKDMLDDT--DDDIDILEECII    1583
Xenopus.laevis                        LKIMPPVLENDQGNKAEPEKEF--IDNKAKKEDKRSEQEKDMLDDT--DDDIDILEECII    1581
Ornithorhynchus.anatinus              LRIMPPVQENDHGNETDPEQPGEPTEKQEKAAEKPTESEKDILDDSD-DDDIDILEECII    1585
Monodelphis.domestica                 LRLMPPVQENEHGNETEPEQLEDVSEVKEKKAEKPTESEKDILDDSD-DDDIEILEECII    1580
Mus.musculus                          LRIMPPVQENDNGNETESEQPEESNENQDKEVEKP-DSEKDLLDDSD-DDDIEILEECII    1578
Rattus.norvegicus                     LRIMPPVQENDNGNETEPEQPEESNENQDKEVEKP-DSEKDLLDDSD-DDDIEILEECII    1578
Sorex.araneus                         LRIMPPVQENDNGNETESEQPEESNENQEKKMEKPTDSEKDLLDDSD-DDDIEILEECII    1576
Octodon.degus                         LRIMPPVQENDNGNETEPEQPEESNENQEEEAEKRIDSEKDLLDDSD-DDDIEILEECII    1579
Heterocephalus                        LRIMPPVQENDNGNETEPEQPEESNENQEKEAEKHIDSEKDLLDDSD-DDDIEILEECII    1577
Jaculus.jaculus                       LRIMPPVQENDNGNETEPEQPEEPSEKQEKEMEKPIDSEKDLLDDSD-DDDIEILEECII    1579
Loxodonta.africana                    LRIMPPVQENDNGNETESEQPEESNENQEKEVEKPTDSEKDLLDDSD-DDDIEILEECII    1580
Trichechus.manatus.latirostris        LRIMPPVQENDNGNETESEQPEESNENQEKEAEKPVDSEKDILDDSD-DDDIEILEECII    1581
Otolemur.garnettii                    LRIMPPVQENDNGNETESEQHEESNENQEKEAEKPVDSEKDLLDDSD-DDDIEILEECII    1580
Callithrix.jacchus                    LRIMPPVQENDNGNETESEQPKESNENQEKEVEKTVDSEKDLLDDSD-DDDIEILEECII    1580
Macaca.mulatta                        LRIMPPVQENDNGNETESEQPKESNENQEKEAEKTIDSEKDLLDDSD-DDDIEILEECII    1580
Nomascus.leucogenys                   LRIMPPVQENDNGNETESEQPKESNENQEKEAEKTIDSEKDLLDDSD-DDDIEILEECII    1580
Pongo.abelii                          LRIMPPVQENDNGNETESEQPKESNENQEKEAEKTIDSEKDLLDDSD-DDDIEILEECII    1580
Homo.sapiens                          LRIMPPVQENDNGNETESEQPKESNENQEKEAEKTIDSEKDLLDDSD-DDDIEILEECII    1580
Gorilla.gorilla                       LRIMPPVQENDNGNETESEQPKESNENQEKEAEKTIDSEKDLLDDSD-DDDIEILEECII    1580
Pan.troglodytes                       LRIMPPVQENDNGNETESEQPKESNENQEKEAEKTIDSEKDLLDDSD-GDDIEILEECII    1580
Pan.paniscus                          LRIMPPVQENDNGNETESEQPKESNENQEKEAEKTIDSEKDLLDDSD-GDDIEILEECII    1580
Canis.lupus.familiaris                LRIMPPVQENDNGNETESEQPEEANENQEKEPEKPTDSEKDLLDDSD-DDDIEILEECII    1580
Bos.taurus                            LRIMPPVQENDNGNETESEQPEESNENQEKEAEKPTDSEKDLLDESD-DDDIEILEECII    1586
Ovis.aries                            LRIMPPVQENDNGNETESEQPEESNENQEKEAEKPTDSEKDLLDESD-DDDIEILEECII    1587
Orcinus.orca                          LRIMPPVQENDNGNETENEQPEESNESQGKEAEKPTDSEKDLLDDSD-DDDIEILEECII    1583
Sus.scrofa                            LRIMPPVQENDNGNETENEQPEKSNENQEKEAEKPTDSEKDLLDDSD-DDDIEILEECII    1583
Ceratotherium.simum.simum             LRIMPAVQENDNGNETESEQPEESNENQEKEAEKPTDSEKDLLDDSD-DDDIEILEECII    1581
Equus.caballus                        LRIMPAVQENDNGNETESEQPEESNENQEKETEKPTDSEKDLLDDSD-DDDIEILEECII    1581
Felis.catus                           LRIMPPVQENDNGNETESEQPEEANESQVKEAEKPTDSEKDLLDDSD-DDDIEILEECII    1581
Ailuropoda.melanoleuca                LRIMPPVQENDNGNETESEQPEEANENQEKETEKPTDSEKDLLDDSD-DDDTEILEECII    1580
Mustela.putorius.furo                 LRIMPPVQENDNGNETESEQPEEANENQEKETEKPTDSEKDLLDDSD-DDDIEILEECII    1580
Odobenus.rosmarus.divergens           LRIMPPVQENDNGNETESEQPEEANENQEKETEKPTDSEKDLLDDSD-DDDIEILEECII    1580
Taeniopygia.guttata                   LRIMPPVHENEHGNEAEPEQSDDTKDNQEKKPEKPPEAEKDILDDSD--DDIEILEACII    1581
Anolis.carolinensis                   LRIMPPVHENDHGNEAELEQSNDTKDKQEKNIEKPTETEKDILDDSD-EDDIEILEECII    1579
Gallus.gallus                         LRIMPPVHENEHGNEAEPEQSDETKDNQENKAEKPSEAEKDILDDSD-DDDIEILEECII    1592
                                        ::    :.        :                        .:        : :

Ciona.intestinalis                    TSLPTADEIKNYGC----EGTLSP-MTSL-SEISVLRDHNEGFVKSTSGASPLKSRTGSH    1335
Strongylocentrotus.purpuratus         AAMPVRRKGIPKPT------------KSKSKREITS------------RKIPVSHKPSSS    1478
Danio.rerio                           SAMPTKSSRKPKKQ---STSRIPPPVACKPSQLPVY------------KLLPPQNRGQPQ    1598
Xenopus.tropicalis                    SAMPRKPSRKNKKVPQPTAGKPPPPVARKPSQLPVY------------KLLSSQNRLQTQ    1631
Xenopus.laevis                        SAMPRKPSRKNKKVPQPTPGKPPPPVARKPSQLPVY------------KLLSSQNRLQTQ    1629
Ornithorhynchus.anatinus              SAMPTKSSRKAKKPSQA-ASKIPPPVARKPSQLPVY------------KLLPSQSRLQPQ    1632
Monodelphis.domestica                 SAMPTKSSRKAKKPSQA-ASKIPPPVARKPSQLPVY------------KLLPSQNRLQAQ    1627
Mus.musculus                          SAMPTKSSRKAKKLAQT-ASKLPPPVARKPSQLPVY------------KLLPAQNRLQAQ    1625
Rattus.norvegicus                     SAMPTKSSRKAKKLAQT-ASKLPPPVARKPSQLPVY------------KLLPSQSRLQAQ    1625
Sorex.araneus                         SAMPTKSSHKVKKPSQT-ASKLPPPVARKPSQLPVY------------KLLPSQNRLQAQ    1623
```

```
Octodon.degus                       SAMPTKSSRKAKKVAQT-ASKLPPPVARKPSQLPVY------------KLLPSQNRLQTQ    1626
Heterocephalus                      SAMPTKSSRKAKKLAQT-ASKLPPPVARKPSQLPVY------------KLLPSQNRLQTQ    1624
Jaculus.jaculus                     SAMPTKSSRKAKKLAQT-TSKLPPPVARKPSQLPVY------------KLLPSQNRLQAQ    1626
Loxodonta.africana                  SAMPTNSSRKDKKPAQT-ASKVPPPVARKPSQLPVY------------KLLPSQNRLQTQ    1627
Trichechus.manatus.latirostris      SAMPTNSSRKNKKPAQT-ASKVPPPVARKPSQLPVY------------KLLPSQNRLQAQ    1628
Otolemur.garnettii                  SAMPTKSSHKAKKTGQT-ASKLPPPVARKPSQLPVY------------KLLPSQNRLQAQ    1627
Callithrix.jacchus                  SAMPTKSSHKAKKPAQT-ASKLPPPVARKPSQLPVY------------KLLPSQNRLQPQ    1627
Macaca.mulatta                      SAMPTKSSRKAKKPAQT-ASKLPPPVARKPSQLPVY------------KLLPSQNRLQPQ    1627
Nomascus.leucogenys                 SAMPTKSSRKAKKPAQT-ASKLPPPVARKPSQLPVY------------KLLPSQNRLQPQ    1627
Pongo.abelii                        SAMPTKSSRKAKKPAQT-ASKLPPPVARKPSQLPVY------------KLLPSQNRLQPQ    1627
Homo.sapiens                        SAMPTKSSRKAKKPAQT-ASKLPPPVARKPSQLPVY------------KLLPSQNRLQPQ    1627
Gorilla.gorilla                     SAMPTKSSRKAKKPAQT-ASKLPPPVARKPSQLPVY------------KLLPSQNRLQPQ    1627
Pan.troglodytes                     SAMPTKSSCKAKKPAQT-ASKLPPPVARKPSQLPVY------------KLLPSQNRLQPQ    1627
Pan.paniscus                        SAMPTKSSRKAKKPAQT-ASKLPPPVARKPSQLPVY------------KLLPSQNRLQPQ    1627
Canis.lupus.familiaris              SAMPTKSSRKAKKPAQT-APKLPPPVARKPSQLPVY------------KLLPSQNRLQAQ    1627
Bos.taurus                          SAMPTKSSRKAKKPAQT-TSKLPPPVARKPSQLPVY------------KLLPSQNRLQAQ    1633
Ovis.aries                          SAMPTKSSRKAKKPAQT-TSKLPPPVARKPSQLPVY------------KLLPSQNRLQAQ    1634
Orcinus.orca                        SAMPTKSSRKAKKPAQT-SSKVPPPVARKPSQLPVY------------KLLPSQNRLQTQ    1630
Sus.scrofa                          SAMPTKSSRKAKKPAQT-ASKLPPPVARKPSQLPVY------------KLLPSQNRLQAQ    1630
Ceratotherium.simum.simum           SAMPTKSSRKAKKPAQT-ASKLPPPVARKPSQLPVY------------KLLPSQNRIQTP    1628
Equus.caballus                      SAMPTKSSRKAKKPAQT-ASKLPPPVARKPSQLPVY------------KLLPSQNRIQAQ    1628
Felis.catus                         SAMPTKSSRKAKKPAQT-APKLPPPVARKPSQLPVY------------KLLPSQNRLQTQ    1628
Ailuropoda.melanoleuca              SAMPTKSSRKAKKPAQT-ASKLPPPVARKPSQLPVY------------KLLPSQNRLQAQ    1627
Mustela.putorius.furo               SAMPTKSSRKAKKPAQA-ASKLPPPVARKPSQLPVY------------KLLPSQNRLQAQ    1627
Odobenus.rosmarus.divergens         SAMPTKSSRKAKKPAQT-ASKLPPPVARKPSQLPVY------------KLLPSQNRLQAQ    1627
Taeniopygia.guttata                 SAMPTKSSRKAKKPSQASAPKIPPPVARKPSQLPVY------------KLLPSQSRLQSQ    1629
Anolis.carolinensis                 SAMPTKSSRKAKKPTQTTASKIPPPVARKPSQLPVY------------KLLPSQNRLHSQ    1627
Gallus.gallus                       SAMPTKSSRKAKKPSQASAPKIPPPVARKPSQLPVY------------KLLPSQSRLQSQ    1640
                                    :::*   .                      ..  .                  . :

Ciona.intestinalis                  KSTSRS----SNIAHLASLPESSRSSGTHAST----------------------------    1363
Strongylocentrotus.purpuratus       KHVSFEKSNAAPPTTMQDLPKPFCTEDTPVNFSAATSLSDLSIDDIEIESSSDGKLMIAN    1538
Danio.rerio                         KHVALA--------HGEDMPRVYCVEGTPINFSTATSLSDLTIDSPPNELAGMESSA-PH    1649
Xenopus.tropicalis                  KHVNFT--------HSDDMPRVYCVEGTPINFSTATSLSDLTIESPPNELTNNDQPN-TG    1682
Xenopus.laevis                      KHVNFT--------HSDDMPRVYCVEGTPINFSTATSLSDLTIESPPSEPT-NDQPN-TD    1679
Ornithorhynchus.anatinus            KHVSFT--------PGDDMPRVYCVEGTPINFSTATSLSDLTIESPPNELAAIENIG-TG    1683
Monodelphis.domestica               KHVSFT--------PGDDVPRVYCVEGTPINFSTATSLSDLTIESPPNELAGVEGTS-TG    1678
Mus.musculus                        KHVSFT--------PGDDVPRVYCVEGTPINFSTATSLSDLTIESPPNELATGDGVR-AG    1676
Rattus.norvegicus                   KHVSFT--------PGDDVPRVYCVEGTPINFSTATSLSDLTIESPPNELAAGDGVR-AS    1676
Sorex.araneus                       KHVSFT--------PGDDVPRVYCVEGTPINFSTATSLSDLTIESPPNELAAGEGVR-AG    1674
Octodon.degus                       KHVSFT--------PGDDMPRVYCVEGTPINFSTATSLSDLTIESPPNELAAGEGAK-GG    1677
Heterocephalus                      KHVSFT--------PGDDMPRVYCVEGTPINFSTATSLSDLTIESPPNELAVGEGAR-AG    1675
Jaculus.jaculus                     KHVSFT--------PGDDMPRVYCVEGTPINFSTATSLSDLTIESPPNELAAGEGVK-AG    1677
Loxodonta.africana                  KHV---------------MPRVYCVEGTPVNFSTATALSDLTMESPPNELAAGEGVR-AG    1671
Trichechus.manatus.latirostris      KHV---------------MPRVYCVEGTPINFSTAASLNDLTIESPPDELAAGEGVR-AG    1672
Otolemur.garnettii                  KHVSFT--------PGDDMPRVYCVEGTPINFSTATSLSDLTIESPPNELAAGEGVR-AG    1678
Callithrix.jacchus                  KHVSFT--------PGDDVPRVYCVEGTPINFSTATSLSDLTIESPPNELAAGEGVR-AG    1678
Macaca.mulatta                      KHVSFT--------PGDDMPRVYCVEGTPINFSTATSLSDLTIESPPNELAAGEGVR-AG    1678
Nomascus.leucogenys                 KHVSFT--------PGDDMPRVYCVEGTPINFSTATSLSDLTIESPPNELAAGEGVR-GG    1678
Pongo.abelii                        KHVSFT--------PGDDMPRVYCVEGTPINFSTATSLSDLTIESPPNELAVGEGVR-GG    1678
Homo.sapiens                        KHVSFT--------PGDDMPRVYCVEGTPINFSTATSLSDLTIESPPNELAAGEGVR-GG    1678
Gorilla.gorilla                     KHVSFT--------PGDDMPRVYCVEGTPINFSTATSLSDLTIESPPNELAAGEGVR-GG    1678
Pan.troglodytes                     KHVSFT--------PGDDMPRVYCVEGTPINFSTATSLSDLTIESPPNELAAGEGVR-GG    1678
Pan.paniscus                        KHVSFT--------PGDDMPRVYCVEGTPINFSTATSLSDLTIESPPNELAAGEGVR-GG    1678
Canis.lupus.familiaris              KHVSFT--------PGEDMPRVYCVEGTPINFSTATSLSDLTIESPPNELAAGEGVR-AG    1678
Bos.taurus                          KHVSFT--------PGDDMPRVYCVEGTPINFSTATSLSDLTIESPPNELAAGEGVR-AG    1684
Ovis.aries                          KHVSFT--------PGDDMPRVYCVEGTPINFSTATSLSDLTIESPPNELAAGEGVR-AG    1685
Orcinus.orca                        KHVSFT--------PGDDMPRVYCVEGTPINFSTATSLSDLTIESPPNELAAGEGVR-AG    1681
Sus.scrofa                          KHVSFT--------PGDDVPRVYCVEGTPINFSTATSLSDLTIESPPNELAAGEGAR-AG    1681
Ceratotherium.simum.simum           KHVSFT--------PGDDMPRVYCVEGTPINFSTATSLSDLTIESPPNELAAGEGVR-AR    1679
Equus.caballus                      KHVSFT--------PGDDMPRVYCVEGTPINFSTATSLSDLTIESPPNELAAGEGVR-AG    1679
Felis.catus                         KHVSFT--------PGDDMPRVYCVEGTPINFSTATSLSDLTIESPPNELAAGEGVR-AG    1679
Ailuropoda.melanoleuca              KHVSFT--------PGDDMPRVYCVEGTPINFSTATSLSDLTIESPPNELAAGEGVR-AA    1678
Mustela.putorius.furo               KHVSFT--------PGDDMPRVYCVEGTPINFSTATSLSDLTIESPPNELAAGEGVR-PG    1678
Odobenus.rosmarus.divergens         KHVSFT--------PGDDMPRVYCVEGTPINFSTATSLSDLTIESPPNELAAGEGVR-TG    1678
```

```
Taeniopygia.guttata                 KHVTFT--------PGDDMPRVYCVEGTPINFSTATSLSDLTIESPPSELANADNVG-VG    1680
Anolis.carolinensis                 KHVSFT--------PGDDMPRVYCVEGTPINFSTATSLSDLTIESPPNELTGAETSG-TG    1678
Gallus.gallus                       KHVSFT--------PGDDMPRVYCVEGTPINFSTATSLSDLTIESPPNELANVDSVG-AG    1691
                                    * .               :*.    ..*  .

Ciona.intestinalis                  --------SSGSKSDHALPVTDENDHLDRIDDLGDVDDLSDSGSLGSLSDGEDLLQACIQ    1415
Strongylocentrotus.purpuratus       PDRSALEKPSGIESDNQVRT--SGDLLHCM------DDARSETNSVTKEAEDSMLQECIN    1590
Danio.rerio                         VEAS-GQRRDTL------PE---GKSAEAKET----GLSPPMQSALAENEGDDILAECIN    1695
Xenopus.tropicalis                  SLSTDLEKRDTI------PT--EGRSTDDTDASKKLNPSTSVLDEDKAEEG-DILAECIH    1733
Xenopus.laevis                      SLSTDLEKRDTI------PT--EGRSTDDTDASKPLNPT-TVLDEDKAEEG-DILAECIH    1729
Ornithorhynchus.anatinus            AQPAEFEKRDTI------PT--EGRSIEDIQRGKNSASTTPVLDDGKAEEG-DILAECIN    1734
Monodelphis.domestica               ALLGDFEKRDTI------PT--EGRSTDDIQIGKSSNVNTSAFDDNKTEEG-EILAECIN    1729
Mus.musculus                        IQSGEFEKRDTI------PT--EGRSTDDAQRGKISSIVTPDLDDNKAEEG-DILAECIN    1727
Rattus.norvegicus                   VQSGEFEKRDTI------PT--EGRSTDEAQRGKVSSIAIPDLDGSKAEEG-DILAECIN    1727
Sorex.araneus                       PQVGETEKRDTI------PT--EGRSTDEAHRGKVSSLTTPESEDSKAEES-DILAECIN    1725
Octodon.degus                       TQSSEFEKRDTI------PT--EGRSTDEAQRGKPSSIAMPELNDSKTEEG-DILAECIN    1728
Heterocephalus                      TQSSEFENRDTI------PT--EGRSTDEVQRGKPSSIPMPELDDNKTEEG-DILAECIN    1726
Jaculus.jaculus                     ALSGEFEKRDTI------PT--EGRSTDEAQRGKASSVATPDTDDSKAEEG-DILAECIN    1728
Loxodonta.africana                  AQSGEFEKRDTI------PT--EGRSTDEAQRGKP-SITIPELDDNKTEEG-DILAECIN    1721
Trichechus.manatus.latirostris      AQSGEFEKRDTI------PT--EGRSTDEAQRGKTSSITIPELDDSKTEEG-DILAECIN    1723
Otolemur.garnettii                  TQSGEFEKRDTI------PT--EGRSTDEAHRGKTSSLTIPELDDSKTEEG-DILAECIN    1729
Callithrix.jacchus                  AQSGEFEKRDTI------PT--EGRSTDEAQGGKNSSVTIPELDDNKAEEG-DILAECIN    1729
Macaca.mulatta                      AQSGEFEKRDTI------PT--EGRSTDEAQGGKTSSVTIPELDDNKAEEG-DILAECIN    1729
Nomascus.leucogenys                 AQSGEFEKRDTI------PT--EGRSTDEAQGGKTSSVTIPELDDNKTEEG-DILAECIN    1729
Pongo.abelii                        AQSGEFEKRDTI------PT--EGRSTDEAQGGKTSSVTIPELDDNKAEEG-DILAECIN    1729
Homo.sapiens                        AQSGEFEKRDTI------PT--EGRSTDEAQGGKTSSVTIPELDDNKAEEG-DILAECIN    1729
Gorilla.gorilla                     AQSGEFEKRDTI------PT--EGRSTDEAQGGKTSSVTIPELDDNKAEEG-DILAECIN    1729
Pan.troglodytes                     AQSGEFEKRDTI------PT--EGRSTDEAQGGKTSSVTIPELDDNKAEEG-DILAECIN    1729
Pan.paniscus                        AQSGEFEKRDTI------PT--EGRSTDEAQGGKTSSVTIPELDDNKAEEG-DILAECIN    1729
Canis.lupus.familiaris              AQPSEFEKRDTI------PT--EGRSTDEAQRGKAPAVTIPELDDSKTEEG-DILAECIN    1729
Bos.taurus                          AQSSEFEKRDTI------PT--EGRSTDEAQRGKASSVTVPELDDSKTEEG-DILAECIN    1735
Ovis.aries                          AQSSEFEKRDTI------PT--EGRSTDEAQRGNASSVTVPELDDNKTEEG-DILAECIN    1736
Orcinus.orca                        TQSGEFEKRDTI------PT--EGRSTDEAQTGKASSVTIPELDDNKTEEG-DILAECIN    1732
Sus.scrofa                          AQSGEFEKRDTI------PT--EGRSTDEAQRGKSTSVAIPELDDNKTEEG-DILAECIN    1732
Ceratotherium.simum.simum           AQSGEFEKRDTI------PT--EGRSTDEAQRGKPSSINIPELDDNKTEEG-DILAECIN    1730
Equus.caballus                      AQSGEFEKRDTI------PT--EGRSTDEAQRGKVSSITIPELDDNKTEEG-DILAECIN    1730
Felis.catus                         AQSGEFEKRDTI------PT--EGRSTDEAQRVKTPSATISELDDNKTEEG-DILAECIN    1730
Ailuropoda.melanoleuca              AQAREFEKRDTI------PT--EGRSTDEAQRGKTPSVTIPELDDSKTEEG-DILAECIN    1729
Mustela.putorius.furo               AQSSEFEKRDTI------PT--EGRSTDEAQRGKTPSVTIPELDDSKTEEG-DILAECIN    1729
Odobenus.rosmarus.divergens         AQSSEFEKRDTI------PT--EGRCTDEAQRGKTPSVTIPELDDNKTEEG-DILAECIN    1729
Taeniopygia.guttata                 AESGEFEKRDTI------PT--EGRSTDDSQRGKTSTVTPLGLDDDKTEEG-DILAECIN    1731
Anolis.carolinensis                 VESAEFEKRDTI------PT--EGRDTDDAYRGKNSTGTTVGLDDDKAEEGDDILAECIN    1730
Gallus.gallus                       AESGEFEKRDTI------PT--EGRSTDDTQRAKSITVTGPGLDDDKTEEG-DILAECIN    1742
                                             .             .   .               .    .    .:* **:

Ciona.intestinalis                  SAIPKKSSSRSSSTKPSSRGKDKSMGRK-ANTSKHPVKPAPKKEEPISKQSPLSPPAQV-    1473
Strongylocentrotus.purpuratus       SAIPRSKSCKPRSSLLAKHRSPRQIGKFRSPKGVAPRKRLPSADVRHKSPSPIPEMSSDT    1650
Danio.rerio                         SAMPKSKIHKPFRVQKMP----DQAQHPSTATG---SLVQQ-DLEKKKPTSPVKPMPQSS    1747
Xenopus.tropicalis                  SAMPKGKSHKPYRVKKIM----DQINHTSAGTSSGNNRSMQ-EIDKNKPTSPVKPMPQSI    1788
Xenopus.laevis                      SAMPKGKSHKPYRVKKIM----DQINHTSAATSSGNSRSMQ-ETDKNKPTSPVKPMPQSI    1784
Ornithorhynchus.anatinus            SAMPKGKSHKPFRVKKIM----DQVQQASAA-SSGSSISQP-DGERKKPTSPVKPMPQSI    1788
Monodelphis.domestica               SAMPKGKSHKPFRVKKIM----DQIQQASAS-SSGNSKNPL-DSEKKKPTSPVKPMPQSA    1783
Mus.musculus                        SAMPKGKSHKPFRVKKIM----DQVQQASST-SSGANKNQV-DTKKKKPTSPVKPMPQNT    1781
Rattus.norvegicus                   SALPKGRSHKPFRVKKIM----DQVQQASMT-SSGTNKNQI-DTKKKKPTSPVKPMPQNT    1781
Sorex.araneus                       SAMPKGKSHKPFRVKKIM----DQVQQASIS-SSGTNKNLA-DSKKKKPTSPVKPMPQNS    1779
Octodon.degus                       SAMPKGKSHKPFRVKKIM----DQVQQASVS-SPGTNKTQI-DNKKKKPTSPVKPMPQNT    1782
Heterocephalus                      SAMPKGKSHKPFRVKKIM----DQVQQASVS-SPGTNKNQI-DSKKKKPTSPVKPMPQNT    1780
Jaculus.jaculus                     SAMPKGKSHKPFRVKKIM----DQVQQAVS-SSGTNKNQV-DGKKKKPTSPVKPMPQNI    1782
Loxodonta.africana                  SAMPKGKSHKPFRVKKIM----DQVQQASAS-SSGTNKNQL-DGKKKKPTSPVKPIPQNT    1775
Trichechus.manatus.latirostris      SAMPKGKSHKPFRVKKIM----DQVQQASAS-SSGTNENQS-DGKKKKPTSPVKPIPQNT    1777
Otolemur.garnettii                  SAMPKGKSHKPFRVKKIM----DQVQQASVS-SPGTNKNQL-DAKKKKPTSPVKPIPQNT    1783
Callithrix.jacchus                  SAMPKGKSHKPFRVKKIM----DQVQQASAS-SSATNKNQL-DGKKKKPTSPVKPIPQNN    1783
Macaca.mulatta                      SAMPKGKSHKPFRVKKIM----DQVQQASAS-SSATNKNQL-DGKKKKPTSPVKPIPQNT    1783
Nomascus.leucogenys                 SAMPKGKSHKPFRVKKIM----DQVQQASAS-SSAPNKNQL-DGKKKKPTSPVKPIPQNT    1783
Pongo.abelii                        SAMPKGKSHKPFRVKKIM----DQVQQASAS-SSAPNKNQL-DGKKKKPTSPVKPIPQNT    1783
```

```
Homo.sapiens                          SAMPKGKSHKPFRVKKIM----DQVQQASAS-SSAPNKNQL-DGKKKKPTSPVKPIPQNT    1783
Gorilla.gorilla                       SAMPKGKSHKPFRVKKIM----DQVQQASAS-SSAPNKNQL-DGKKKKPTSPVKPIPQNT    1783
Pan.troglodytes                       SAMPKGKSHKPFRVKKIM----DQVQQASAS-SSAPNKNQL-DGKKKKPTSPVKPIPQNT    1783
Pan.paniscus                          SAMPKGKSHKPFRVKKIM----DQVQQASAS-SSAPNKNQL-DGKKKKPTSPVKPIPQNT    1783
Canis.lupus.familiaris                SAMPKGKSHKPFRVKKIM----DQVQQASVS-SSGTNKTQL-DGKKKKPTSPVKPIPQSA    1783
Bos.taurus                            SAMPKGKSHKPFRVKKIM----DQVQQASMS-SSGTNKNQL-DGKTKKPTSPVKPIPQNT    1789
Ovis.aries                            SAMPKGKSHKPFRVKKIM----DQVQQASMS-SSGTNKNQL-DGKTKKPTSPVKPIPQST    1790
Orcinus.orca                          SAMPKGKSHKPFRVKKIM----DQVQQASMS-SSGTNKNQL-DGKKKKPTSPVKPIPQNT    1786
Sus.scrofa                            SAMPKGKSHKPFRVKKIM----DQVQQASMS-SSGANKNQL-DGKKKKPTSPVKPIPQNA    1786
Ceratotherium.simum.simum             SAMPKGKSHKPFRVKKIM----DQVQQASVS-SSGTNKNQL-DGKKTKPTSPVKPMPQNT    1784
Equus.caballus                        SAMPKGKSHKPFRVKKIM----DQVQQASTS-SSGTNKTQL-DGKKTKPTSPVKPMPQNT    1784
Felis.catus                           SAMPKGKSHKPFRVKKIM----DQVQQASMS-SSGANKNQL-DGKKKKPTSPVKPIPQNT    1784
Ailuropoda.melanoleuca                SAMPKGKSHKPFRVKKIM----DQVQQASMS-SSGANKNQL-DGKKKKPTSPVKPMPQNT    1783
Mustela.putorius.furo                 SAMPKGKSHKPFRVKKIM----DQVQQASMS-SSGANKNQL-DGKKKKPTSPVKPIPQNT    1783
Odobenus.rosmarus.divergens           SAMPKGKSHKPFRVKKIM----DQVQQASMS-SSGANKNQL-DGKKKKPTSPVKPIPQNT    1783
Taeniopygia.guttata                   SAMPKGKSHKPFRVKKIM----DQIQQASS---SPSSKNQP-EGEKKKPTSPVKPVSQNS    1783
Anolis.carolinensis                   SAMPKGKSHKPFRVKKIM----DQIQQASPP-SSVSNKNQL-EVEKKKPTSPVKPMPQSN    1784
Gallus.gallus                         SAMPKGKSHKPFRVKKIM----DQIQQAST---SLNNKNQP-EGEKKKPTSPVKPVPQNS    1794
                                      **:*:    :             .  :                    .  **:    .

Ciona.intestinalis                    --RPV--RKFDIDSSLAAAENKLQGMPILQYIEKKISEAPGLSDEDKETHAHPTTSNPSK    1529
Strongylocentrotus.purpuratus         RSTSSQERGQDWGNNPSPPPD-----TVTTYCME---GTPGISNAT--------------    1688
Danio.rerio                           EYRARMLKRPEANNSLAD---------PATYPDK---NK-----ET--------------    1776
Xenopus.tropicalis                    EFKGRLKKNTESKLNPSS---------ENQYSD------------P--------------    1813
Xenopus.laevis                        GFKERLKKNTELKLNPNS---------ENQYCD------------P--------------    1809
Ornithorhynchus.anatinus              EYRARVKKNADSKNNFNM---------ERSYTD----HK-----EA--------------    1816
Monodelphis.domestica                 EYRTRIRKNAESKN-VNV---------ERSYS-----DK-----DS--------------    1809
Mus.musculus                          EYRTRVRKNTDSKVNVNT---------EETFSD----NK-----DS--------------    1809
Rattus.norvegicus                     EYRTRVRKNTDSKVNVNT---------EETFSD----NK-----DS--------------    1809
Sorex.araneus                         EYRTRVRKNVDLKSNLSA---------ERTFLD----NK-----DP--------------    1807
Octodon.degus                         ECRTRVRKNTDSKNNLST---------EQTFSD----NK-----DS--------------    1810
Heterocephalus                        ECRTRARKNTDSKNSLNA---------DRTFSD----NK-----DS--------------    1808
Jaculus.jaculus                       EYRTRVRKNADSKNNIS----------EQSFPD----NK-----DS--------------    1809
Loxodonta.africana                    EYRTRVRKNTETKNNLNA---------ERTFSD----NK-----DS--------------    1803
Trichechus.manatus.latirostris        EYRTRVRKNTDTKNNLNA---------ERTFSD----NK-----DS--------------    1805
Otolemur.garnettii                    EYRAHVRKNADSKNTLSA---------ERAFSD----NK-----DS--------------    1811
Callithrix.jacchus                    EYRTRVRKNVDSKNNLNA---------ERALSD----NK-----DS--------------    1811
Macaca.mulatta                        EYRTRIRKNADSKNNLNA---------ERVFSD----NK-----DS--------------    1811
Nomascus.leucogenys                   EYRTRVRKNADSKNNLNA---------ERVFSD----NK-----DS--------------    1811
Pongo.abelii                          EYRTRVRKNADSKNNLNA---------ERVFPD----NK-----DS--------------    1811
Homo.sapiens                          EYRTRVRKNADSKNNLNA---------ERVFSD----NK-----DS--------------    1811
Gorilla.gorilla                       EYRTRVRKNADSKNNLNA---------ERVFSD----NK-----DS--------------    1811
Pan.troglodytes                       EYRTRVRKNADSKNNLNA---------ERVFSD----NK-----DS--------------    1811
Pan.paniscus                          EYRTRVRKNADSKNNLNA---------ERVFSD----NK-----DS--------------    1811
Canis.lupus.familiaris                EYRTRVRKHTDSKNNVNA---------ERTFSD----NK-----DS--------------    1811
Bos.taurus                            EYRTRVRKNTDSKNNLNA---------ERNFSE----NK-----DS--------------    1817
Ovis.aries                            EYRTRVRKNTDSKNNLNA---------ERNFSE----NK-----DS--------------    1818
Orcinus.orca                          EYRTRVRKTTDSKNNLNA---------ERNFSD----NK-----DS--------------    1814
Sus.scrofa                            EYRTRVRKNTDTKNNLNA---------ERAFSD----NK-----DS--------------    1814
Ceratotherium.simum.simum             EYRTRVRKNADSKNNLNA---------ERTFSD----NK-----DS--------------    1812
Equus.caballus                        EYRTRVRKNADSKNNLNA---------ERTFSD----NK-----DS--------------    1812
Felis.catus                           EYRTRVRKNTDSKNNLNA---------ERTFSD----NK-----DS--------------    1812
Ailuropoda.melanoleuca                EYRTRVRKNTDSKNNVNA---------ERTFSD----NK-----DS--------------    1811
Mustela.putorius.furo                 EYRTRVRKNADSKNNLNA---------ERTFSD----NK-----ES--------------    1811
Odobenus.rosmarus.divergens           EYRTRVRKNTDSKNNLNG---------ERTFSD----NK-----DS--------------    1811
Taeniopygia.guttata                   EYRARVRKSIESKSNASN---------ERGYPE----NR-----DA--------------    1811
Anolis.carolinensis                   EYRARLRKNTEPKNNFNS---------ERTYTD----NK-----DT--------------    1812
Gallus.gallus                         EYRARVRKNTESKSQINN---------ERSYPE----NR-----DA--------------    1822
                                             :  :

Ciona.intestinalis                    SDTLDASDMTLTSQDITLNQSALDASDAARRSKTCREVIKTVETLNESYSVKKQLKVFEK    1589
Strongylocentrotus.purpuratus         ---------SLSDLTSPID-----------------------------------S-----    1699
Danio.rerio                           -----------RKQEPKVV-----------------------------------I-----    1785
Xenopus.tropicalis                    -----------RKQNSKNT-----------------------------------S-----    1822
Xenopus.laevis                        -----------RKPSSKKP-----------------------------------S-----    1818
```

```
Ornithorhynchus.anatinus            -----------KKQNLKNH-----------------------------------S-----    1825
Monodelphis.domestica               -----------KKLSLKNN-----------------------------------A-----    1818
Mus.musculus                        -----------KKPSLQTN-----------------------------------A-----    1818
Rattus.norvegicus                   -----------KKQSLKNN-----------------------------------P-----    1818
Sorex.araneus                       -----------KKQNLKNN-----------------------------------S-----    1816
Octodon.degus                       -----------KKQNLKNN-----------------------------------S-----    1819
Heterocephalus                      -----------KKQNLKNN-----------------------------------S-----    1817
Jaculus.jaculus                     -----------KKQSMKNN-----------------------------------S-----    1818
Loxodonta.africana                  -----------KKQSLKNN-----------------------------------S-----    1812
Trichechus.manatus.latirostris      -----------KKQNLKNN-----------------------------------S-----    1814
Otolemur.garnettii                  -----------KKQNLKNN-----------------------------------S-----    1820
Callithrix.jacchus                  -----------KKQNLKNN-----------------------------------S-----    1820
Macaca.mulatta                      -----------KKQNLKNN-----------------------------------S-----    1820
Nomascus.leucogenys                 -----------KKQNLKNN-----------------------------------S-----    1820
Pongo.abelii                        -----------KKQNLKNN-----------------------------------S-----    1820
Homo.sapiens                        -----------KKQNLKNN-----------------------------------S-----    1820
Gorilla.gorilla                     -----------KKQNLKNN-----------------------------------S-----    1820
Pan.troglodytes                     -----------KKQNLKNN-----------------------------------S-----    1820
Pan.paniscus                        -----------KKQNLKNN-----------------------------------S-----    1820
Canis.lupus.familiaris              -----------KKQNLKNN-----------------------------------S-----    1820
Bos.taurus                          -----------KKQHLKNN-----------------------------------S-----    1826
Ovis.aries                          -----------KKQHLKNN-----------------------------------S-----    1827
Orcinus.orca                        -----------KKQNLKNN-----------------------------------S-----    1823
Sus.scrofa                          -----------KKQSLKNN-----------------------------------S-----    1823
Ceratotherium.simum.simum           -----------RKQNLKNN-----------------------------------S-----    1821
Equus.caballus                      -----------KKQNLKNN-----------------------------------S-----    1821
Felis.catus                         -----------KKQNLKNN-----------------------------------S-----    1821
Ailuropoda.melanoleuca              -----------KKQNLKNN-----------------------------------A-----    1820
Mustela.putorius.furo               -----------KKQNLKNN-----------------------------------S-----    1820
Odobenus.rosmarus.divergens         -----------KKQNLKNN-----------------------------------S-----    1820
Taeniopygia.guttata                 -----------KKQNLKNN-----------------------------------S-----    1820
Anolis.carolinensis                 -----------KKQNLKNN-----------------------------------S-----    1821
Gallus.gallus                       -----------KKQNLKNN-----------------------------------S-----    1831
                                                .

Ciona.intestinalis                  QRTDVTTPTTSQSPCNVASRLVPFPSMSNNVKVQAFL-QSRPNTFMTEGTPVEMSCSTSL    1648
Strongylocentrotus.purpuratus       --EDVNGNITSQNLPQNRS------TLGVGVSRGSTPSDTPR-VFAVEGTPINFSCNGSL    1750
Danio.rerio                         --RDFA-----DKPSNAE-----------ERTRPGFAFDSPHHYTPIEGTPYCFSRNDSL    1827
Xenopus.tropicalis                  --KDAN-----EKIPNNE-----------ERTKG-FAFDSPHHYTPIEGTPYCFSRNDSL    1863
Xenopus.laevis                      --KVAN-----EKIPNNE-----------ERTKG-FAFDSPHHYTPIEGTPYCFSRNDSL    1859
Ornithorhynchus.anatinus            --RDFN-----DKLPNNE-----------DRVRGSYAFDSPHHYTPIEGTPYCFSRNDSL    1867
Monodelphis.domestica               --RDFL-----DKMPNNE-----------DRVRGSFTFDSPHHYTPIEGTPYCFSRNDSL    1860
Mus.musculus                        --KAFN-----EKLPNNE-----------DRVRGSFALDSPHHYTPIEGTPYCFSRNDSL    1860
Rattus.norvegicus                   --KDLN-----DKLPDNE-----------DRVRGGFTFDSPHHYAPIEGTPYCFSRNDSL    1860
Sorex.araneus                       --KDYN-----DKQPNNE-----------DRIRGSFAFDSPHHYTPIEGTPYCFSRNDSL    1858
Octodon.degus                       --KDLS-----DKLPNNE-----------DRVKGSFTFDSPHHYTPIEGTPYCFSRNDSL    1861
Heterocephalus                      --KDLN-----DKLPNNE-----------DRVKGSFAFDSPHHYTPIEGTPYCFSRNDSL    1859
Jaculus.jaculus                     --KDFN-----DKLPNNE-----------DRGRGSFTFDSPHHYTPIEGTPYCFSRNDSL    1860
Loxodonta.africana                  --KDLN-----DKLPNNE-----------DRVRGSFAFDSPHHYTPIEGTPYCFSRNDSL    1854
Trichechus.manatus.latirostris      --KDFN-----DKLPNNE-----------DRVRGSFTFDSPHHYTPIEGTPYCFSRNDSL    1856
Otolemur.garnettii                  --KDFN-----NKLPNNE-----------DRVRGSFAFDSPHHYTPIEGTPYCFSRNDSL    1862
Callithrix.jacchus                  --KDLN-----DKLPNNE-----------DRVRGSFAFDSPHHYTPIEGTPYCFSRNDSL    1862
Macaca.mulatta                      --KDFN-----DKLPNNE-----------DRVRGSFAFDSPHHYTPIEGTPYCFSRNDSL    1862
Nomascus.leucogenys                 --KDFN-----DKLPNNE-----------DRVRGSFAFDSPHHYTPIEGTPYCFSRNDSL    1862
Pongo.abelii                        --KDFN-----DKVPNNE-----------DRVRGSFAFDSPHHYTPIEGTPYCFSRNDSL    1862
Homo.sapiens                        --KDFN-----DKLPNNE-----------DRVRGSFAFDSPHHYTPIEGTPYCFSRNDSL    1862
Gorilla.gorilla                     --KDFN-----DKLPNNE-----------DRVRGSFAFDSPHHYTPIEGTPYCFSRNDSL    1862
Pan.troglodytes                     --KDFN-----DKLPNNE-----------DRVRGSFAFDSPHHYTPIEGTPYCFSRNDSL    1862
Pan.paniscus                        --KDFN-----DKLPNNE-----------DRVRGSFAFDSPHHYTPIEGTPYCFSRNDSL    1862
Canis.lupus.familiaris              --KDFN-----DKLPSNE-----------DRVRGSFTFDSPHHYTPIEGTPYCFSRNDSL    1862
Bos.taurus                          --KDFN-----DKLPNNE-----------DRVRGSFTFDSPHHYTPIEGTPYCFSRNDSL    1868
Ovis.aries                          --KDLN-----DKLPNNE-----------DGVRGSFTFDSPHHYTPIEGTPYCFSRNDSL    1869
Orcinus.orca                        --KDFN-----DKVPNNE-----------DRVRGSFTFDSPHHYTPIEGTPYCFSRNDSL    1865
Sus.scrofa                          --KDFN-----DKLPNNE-----------DRVRGSFTFDSPHHYTPIEGTPYCFSRNDSL    1865
Ceratotherium.simum.simum           --KDFN-----DKLPNNE-----------DRVRGSFTFDSPHHYTPIEGTPYCFSRNDSL    1863
```

```
Equus.caballus                    --KDFN-----DKLPNNE-----------DRVRGSFSFDSPHHYTPIEGTPYCFSRNDSL    1863
Felis.catus                       --KDFN-----DKLPNNE-----------DRVRGSFTFDSPHHYTPIEGTPYCFSRNDSL    1863
Ailuropoda.melanoleuca            --KDFN-----DKLPNND-----------DRVRGSFTFDSPHHYTPIEGTPYCFSRNDSL    1862
Mustela.putorius.furo             --KDFN-----DKLPNNE-----------DRVRGSFTFDSPHHYTPIEGTPYCFSRNDSL    1862
Odobenus.rosmarus.divergens       --KDFS-----DKLPNNE-----------DRVRGGFTFDSPHHYTPIEGTPYCFSRNDSL    1862
Taeniopygia.guttata               --REFH-----DKLPNNE-----------ERVRGSFAFDSPHHYTPIEGTPYCFSRNDSL    1862
Anolis.carolinensis               --RDFN-----DKPPNNE-----------ERTRGSFAFDSPHHYTPIEGTPYCFSRNDSL    1863
Gallus.gallus                     --RDFN-----DKLPNNE-----------ERVRGSFTFDSPHHYTPIEGTPYCFSRNDSL    1873
                                             :.  .                      ::       ****  :* . **

Ciona.intestinalis                SNITIESGSDNYDFAQLQKQMVEQHREPPQEMKYS--TTTQ-SDKPTIQSNNKSEQ----    1701
Strongylocentrotus.purpuratus     SSLSCDE--D-AELTEAKAQMKEVSKRNQAGGMSRQQTVIPKQRASF--SNPIEQVQSP    1805
Danio.rerio                       SSLDFED-ED-LDFSKEKAVLRKDKEQRKVPLLKC----------------SV-EQ---P    1865
Xenopus.tropicalis                SSLDFDD-DD-IDLSKEKAELRKEKGTKDTDRKGK----------------YKNEN---P    1902
Xenopus.laevis                    SSLDFED-DD-IDLSKEKAELRKEKGTKDTDQKVK----------------YKHEN---R    1898
Ornithorhynchus.anatinus          SSLDFDD-DD-VDLSREKAELRKGKETKEADANAS----------------SHPEP----    1905
Monodelphis.domestica             SSLDFDD-DD-VDLSREKAEL-KGKEAKETEAKVS----------------NHLEL---T    1898
Mus.musculus                      SSLDFDD-DD-VDLSREKAELRKGKESKDSEAKVT----------------CRPEP---N    1899
Rattus.norvegicus                 SSLDFDD-DD-VDLSREKAELRKGKESKDSEAKVT----------------CHTEP---S    1899
Sorex.araneus                     SSLDFDDDDD-VDLSERAELKKGKENKESEAKVT----------------VHTDL---T    1898
Octodon.degus                     SSLDFDD-DD-VDLSREKDELKKGKENKDTEGKVT----------------SHTEL---T    1900
Heterocephalus                    SSLDFDD-DD-VDLSREKAELRKGKENKDSEAKVT----------------SHTEL---T    1898
Jaculus.jaculus                   SSLDFDD-DD-VDLSREKAELRKGKENKDSEAKAA----------------CHTEL---T    1899
Loxodonta.africana                SSLDFDD-DD-VDLSREKAELRKGKENKESEAKVT----------------SHTEL---T    1893
Trichechus.manatus.latirostris    SSLDFDD-DD-VDLSREKAELRKGKENKESEAKVT----------------SHTEL---T    1895
Otolemur.garnettii                SSLDFDD-DD-VDLSREKAELRKGKENKDSEAKVT----------------SHTDL---S    1901
Callithrix.jacchus                SSLDFDD-DD-VDLSREKAELRKGKENKESEAKVT----------------SHTEL---T    1901
Macaca.mulatta                    SSLDFDD-DD-VDLSREKAELRKAKENKESEAKVT----------------SHTEL---T    1901
Nomascus.leucogenys               SSLDFDD-DD-VDLSREKAELRKAKENKESEAKVT----------------SHTEL---T    1901
Pongo.abelii                      SSLDFDD-DD-VDLSREKAELRKAKENKESEAKVT----------------SHTEL---T    1901
Homo.sapiens                      SSLDFDD-DD-VDLSREKAELRKAKENKESEAKVT----------------SHTEL---T    1901
Gorilla.gorilla                   SSLDFDD-DD-VDLSREKAELRKAKENKESEAKVT----------------SHTEL---T    1901
Pan.troglodytes                   SSLDFDD-DD-VDLSREKAELRKAKENKESEAKVT----------------SHTEL---T    1901
Pan.paniscus                      SSLDFDD-DD-VDLSREKAELRKAKENKESEAKVT----------------SHTEL---T    1901
Canis.lupus.familiaris            SSLDFED-DD-VDLSREKAELRKGKENKDSEAKVP----------------SHTEP---I    1901
Bos.taurus                        SSLDFDD-DD-VDLSREKAELRKGKENKESEAKVT----------------NHTEL---T    1907
Ovis.aries                        SSLDFDD-DD-VDLSREKAELRKGKENKESEAKVT----------------NHTEL---T    1908
Orcinus.orca                      SSLDFDD-DD-VDLSREKAELRKGKESKESEAKVT----------------NHTEL---T    1904
Sus.scrofa                        SSLDFDD-DD-VDLSREKAELRKGKENKESEAKVS----------------NHTEL---A    1904
Ceratotherium.simum.simum         SSLDFDD-DD-VDLSREKAELRKGKENKESEAKVT----------------SHTEL---T    1902
Equus.caballus                    SSLDFDD-DD-VDLSREKAELRKGKENKESEAKVT----------------SHSEL---T    1902
Felis.catus                       SSLDFDD-DD-VDLSREKAELRKGKENKESEAKVT----------------SHTEL---T    1902
Ailuropoda.melanoleuca            SSLDFDD-DD-VDLSREKAELRKGKENKESEAKVT----------------SHTEL---T    1901
Mustela.putorius.furo             SSLDFDD-DD-VDLSREKAELRKGKENKESEAKVT----------------SHTEL---T    1901
Odobenus.rosmarus.divergens       SSLDFDD-DD-VDLSREKAELRKGKENKESEAKVT----------------SHTEL---T    1901
Taeniopygia.guttata               SSLDFDD-DD-VDLSREKAELRKGKEGKEIESKEC----------------SNAEQ---S    1901
Anolis.carolinensis               SSLDFDD--D-VDLSREKAELRKGKESEI---KSN----------------SNTEQ---I    1898
Gallus.gallus                     SSLDFDD-DD-VDLSREKAELRKGKEAKEVETKDC----------------PNVEQ---P    1912
                                  *.:  :.  *  :::. :  : :                               :

Ciona.intestinalis                ----NNTDLFQHAEVPNN---PTCSSSQEILNNLNKQIESLTIQNTETCTSGTGYVTSLP    1754
Strongylocentrotus.purpuratus     ENKYYSMDDYHNAEASKSAMSPLDSPR---------------------------------    1832
Danio.rerio                       ANTN---------MVSTF---QTAPTK---------------------------------    1880
Xenopus.tropicalis                AINQ---------IGKQE----QVGPK---------------------------------    1916
Xenopus.laevis                    AINP---------MGKQD----QTGPK---------------------------------    1912
Ornithorhynchus.anatinus          -------------STNRT---QICPKP---------------------------------    1916
Monodelphis.domestica             SNQQ---------SANRA---QICAKH---------------------------------    1913
Mus.musculus                      SSQQ---------AASKS---QASIKH---------------------------------    1914
Rattus.norvegicus                 SSQQ---------SARKA---QASTKH---------------------------------    1914
Sorex.araneus                     SNQQ---------STTKT---QAVTKH---------------------------------    1913
Octodon.degus                     SSQQ---------SANKT---QVVTKH---------------------------------    1915
Heterocephalus                    SNQQ---------SANKT---QAVTKH---------------------------------    1913
Jaculus.jaculus                   SNQQ---------PANKS---QAVTKQ---------------------------------    1914
Loxodonta.africana                SNQQ---------SANKT---QAVTKQ---------------------------------    1908
Trichechus.manatus.latirostris    SNQQ---------SANKT---QAVTKQ---------------------------------    1910
```

```
Otolemur.garnettii                  SNQQ---------SANKT---QAVTKH---------------------------------    1916
Callithrix.jacchus                  SNQQ---------SANKT---QAIAKY---------------------------------    1916
Macaca.mulatta                      SNQQ---------SASKT---QAIAKH---------------------------------    1916
Nomascus.leucogenys                 SNQQ---------SANKT---QAIAKQ---------------------------------    1916
Pongo.abelii                        SNQQ---------SANKT---QAIAKQ---------------------------------    1916
Homo.sapiens                        SNQQ---------SANKT---QAIAKQ---------------------------------    1916
Gorilla.gorilla                     SNQQ---------SANKT---QAIAKQ---------------------------------    1916
Pan.troglodytes                     SNQQ---------SANKT---QAIAKQ---------------------------------    1916
Pan.paniscus                        SNQQ---------SANKT---QAIAKQ---------------------------------    1916
Canis.lupus.familiaris              SNQQ---------SANKT---QAVTKH---------------------------------    1916
Bos.taurus                          SNQQ---------SASKT---PAVTKQ---------------------------------    1922
Ovis.aries                          SNQQ---------SASKT---PAVTKQ---------------------------------    1923
Orcinus.orca                        SNQQ---------SANKT---QAVPKH---------------------------------    1919
Sus.scrofa                          SNQQ---------SAKTT---QAVTKH---------------------------------    1919
Ceratotherium.simum.simum           SNQQ---------SADKT---QAVPKH---------------------------------    1917
Equus.caballus                      SNQQ---------SADKT---QAVTKH---------------------------------    1917
Felis.catus                         SSQQ---------SANKT---QAVTKH---------------------------------    1917
Ailuropoda.melanoleuca              SNQQ---------SANKT---QAVTKH---------------------------------    1916
Mustela.putorius.furo               TNQQ---------SANKT---QAVTKH---------------------------------    1916
Odobenus.rosmarus.divergens         SNQQ---------STNKT---QAVTKH---------------------------------    1916
Taeniopygia.guttata                 SNQQ---------PSNRT---QVCQKH---------------------------------    1916
Anolis.carolinensis                 SNHQ---------STSRI---QTCQKH---------------------------------    1913
Gallus.gallus                       SGQQ---------PSNRT---QVCQKH---------------------------------    1927


Ciona.intestinalis                  HSDEIKQFKTEGSVG----AYTIFSALTIDSE-------------------------TKI    1785
Strongylocentrotus.purpuratus       ------VFAVEGTPGII-SRADSLSSLSCDEEDASPAVEKSAKERLAEKTRTSRISGSRM    1885
Danio.rerio                         ------P-----------LQKTVF------------------------------------    1887
Xenopus.tropicalis                  ------SLEGRGQPKALVHKPTSF------------------------------------    1934
Xenopus.laevis                      ------SLGGRDQPKALVQKPTSF------------------------------------    1930
Ornithorhynchus.anatinus            ------PPS-RGQPKTSLQKPAAF------------------------------------    1933
Monodelphis.domestica               ------PVE-RGQSKPLLQKQSTF------------------------------------    1930
Mus.musculus                        ------PAN-RAQSKPVLQKQPTF------------------------------------    1931
Rattus.norvegicus                   ------PVN-RGPSKPLLQEQPTF------------------------------------    1931
Sorex.araneus                       ------PVN-RGQSKPILQKQSTF------------------------------------    1930
Octodon.degus                       ------PIN-RGHTKPLPQKQPTF------------------------------------    1932
Heterocephalus                      ------SVN-RGQSKPLPQKPPSF------------------------------------    1930
Jaculus.jaculus                     ------PVS-RSQSKPQLQKQPMF------------------------------------    1931
Loxodonta.africana                  ------PVN-RGQSKPTLQKQSTF------------------------------------    1925
Trichechus.manatus.latirostris      ------PIN-RGQSKPVLQKQSTF------------------------------------    1927
Otolemur.garnettii                  ------PLN-RAQSKPALQKQSTF------------------------------------    1933
Callithrix.jacchus                  ------PVN-RGQPKPILQKQSTF------------------------------------    1933
Macaca.mulatta                      ------PIN-RGQLKPILQKQSTF------------------------------------    1933
Nomascus.leucogenys                 ------PIN-RGQPKPILQKQSTF------------------------------------    1933
Pongo.abelii                        ------PIN-RGQPKPILQKQSTF------------------------------------    1933
Homo.sapiens                        ------PIN-RGQPKPILQKQSTF------------------------------------    1933
Gorilla.gorilla                     ------PIN-RGQPKPILQKQSTF------------------------------------    1933
Pan.troglodytes                     ------PIN-RGQPKPILQKQSTF------------------------------------    1933
Pan.paniscus                        ------PIN-RGQPKPILQKQSTF------------------------------------    1933
Canis.lupus.familiaris              ------PVN-RGQSKPVLQKQPTF------------------------------------    1933
Bos.taurus                          ------PIN-RGQSKPVLQKQSTF------------------------------------    1939
Ovis.aries                          ------PIN-RGQSKPVLQKQSTF------------------------------------    1940
Orcinus.orca                        ------PIN-RGQPKPVLQKQSTF------------------------------------    1936
Sus.scrofa                          ------PIN-RGPSKPMLQKQSTF------------------------------------    1936
Ceratotherium.simum.simum           ------PIN-RGQSKPMLQKQSTF------------------------------------    1934
Equus.caballus                      ------PIN-RGQSKPMLQKQSTF------------------------------------    1934
Felis.catus                         ------PIN-RGQSKPMLQKQSTF------------------------------------    1934
Ailuropoda.melanoleuca              ------PIN-RGQSKPMLQKQSTF------------------------------------    1933
Mustela.putorius.furo               ------PIN-RGQSKPLLQKQSTF------------------------------------    1933
Odobenus.rosmarus.divergens         ------PIN-RGQSKSMLQKQSTF------------------------------------    1933
Taeniopygia.guttata                 ------PVGR--------SQTKTF------------------------------------    1926
Anolis.carolinensis                 ------SPAVRNQPKSLIQKQTSF------------------------------------    1931
Gallus.gallus                       ------PTSR--------SQSKTF------------------------------------    1937
                                                           :
```

```
Ciona.intestinalis                  VPKGPEALPGRTN--DESPPKRSPTQSKIPVRTPPPVLKEEKSRKSSESSLEAK------    1837
Strongylocentrotus.purpuratus       NHLGEGSMAMRRLSSEEAP-------LSYAVEDTPTCF----SHNSSLSALSDNDEEERP    1934
Danio.rerio                         -PQAPKEN---TVVVCDEK-------QKFSIEDTPVCF----SRNSSLSSLSDIDQENNN    1932
Xenopus.tropicalis                  -CPAAKGTQDRGGAKDEKM-------QNFAIENTPVCF----SRNSSLSSLSDIDQENN-    1981
Xenopus.laevis                      -SSAAKGTQDRGGATDEKM-------ENFAIENTPVCF----SRNSSLSSLSDIDQENN-    1977
Ornithorhynchus.anatinus            -PQPSKDIPDRGAASDEKM-------QNFAIENTPVCF----SRNSSLSSLSDIDQENNN    1981
Monodelphis.domestica               -PQSSKDMPDRVAATDEKL-------QNFAIENTPVCF----SRNSSLSSLSDIDQENNN    1978
Mus.musculus                        -PQSSKDGPDRGAATDEKL-------QNFAIENTPVCF----SRNSSLSSLSDIDQENNN    1979
Rattus.norvegicus                   -PQSSKDVPDRGAATDEKL-------QNFAIENTPVCF----SRNSSLSSLSDVDQENNN    1979
Sorex.araneus                       -PQSSK---DRGAATDEKL-------QNFAIENTPVCF----SRNSSLSSLSDIDQENNN    1975
Octodon.degus                       -PQPSKDIPDRGAATDEKL-------QNFAIENTPVCF----SRNSSLSSLSDIDQENN-    1979
Heterocephalus                      -PQSSKDIPDRGAATDEKL-------QNFAIENTPVCF----SRNSSLSSLSDIDQENN-    1977
Jaculus.jaculus                     -SQSPKDIPDRGAATDEKL-------QNFAIENTPVCF----SRNSSLSSLSDIDQENNN    1979
Loxodonta.africana                  -PQSSKDIPDRGAATDEKL-------QNFAIENTPVCF----SRNSSLSSLSDIDQENNN    1973
Trichechus.manatus.latirostris      -PQSSKDLPDRGAATDEKL-------QNFAIENTPVCF----SRNSSLSSLSDIDQENNN    1975
Otolemur.garnettii                  -PQSSKDIPDRGAATDEKL-------QNFAIENTPVCF----SRNSSLSSLSDIDQENNN    1981
Callithrix.jacchus                  -PQSSKDIPERGAATDEKL-------QNFAIENTPVCF----SHNSSLSSLSDIDQENN-    1980
Macaca.mulatta                      -PQSSKDIPDRGAATDEKL-------HNFAIENTPVCF----SHNSSLSSLSDIDQENNN    1981
Nomascus.leucogenys                 -PQSSKDIPDRGAATDEKL-------QNFAIENTPVCF----SHNSSLSSLSDIDQENNN    1981
Pongo.abelii                        -PQSSKDIPDRGAATDEKL-------QNFAIENTPVCF----SHNSSLSSLSDIDQENNN    1981
Homo.sapiens                        -PQSSKDIPDRGAATDEKL-------QNFAIENTPVCF----SHNSSLSSLSDIDQENN-    1980
Gorilla.gorilla                     -PQSSKDIPDRGAATDEKL-------QNFAIENTPVCF----SHNSSLSSLSDIDQENNN    1981
Pan.troglodytes                     -PQSSKDIPDRGAATDEKL-------QNFAIENTPVCF----SHNSSLSSLSDIDQENNN    1981
Pan.paniscus                        -PQSSKDIPDRGAATDEKL-------QNFAIENTPVCF----SHNSSLSSLSDIDQENNN    1981
Canis.lupus.familiaris              -PQSSKDLPDRGAATDEKL-------QNFAIENTPVCF----SRNSSLSSLSDIDQENNN    1981
Bos.taurus                          -PQSSKDIPDRGAATDEKL-------QNFAIENTPVCF----SRNSSLSSLSDIDQENNN    1987
Ovis.aries                          -PQSSKDMPDRGAATDEKL-------QNFAIENTPVCF----SRNSSLSSLSDIDQENNN    1988
Orcinus.orca                        -PQPSKDIPDRGAATDEKL-------QNFAIENTPVCF----SRNSSLSSLSDIDQENNN    1984
Sus.scrofa                          -PQSSKDIPDRGAATDEKL-------QNFAIENTPVCF----SRNSSLSSLSDIDQENNN    1984
Ceratotherium.simum.simum           -PQSSKDIPDRGAATDEKL-------QNFAIENTPVCF----SQNSSLSSLSDIDQENNN    1982
Equus.caballus                      -PQSSKDIPDRGAATDEKL-------QNFAIENTPVCF----SRNSSLSSLSDIDQENNN    1982
Felis.catus                         -PQSSKDIPDRGAATDEKL-------QNFAIENTPVCF----SRNSSLSSLSDIDQENNN    1982
Ailuropoda.melanoleuca              -PQSSKDIPDRGAATDEKL-------QNFALENTPVCF----SRNSSLSSLSDIDQENNN    1981
Mustela.putorius.furo               -PQSSKDIPDRGAATDEKL-------QNFAIENTPVCF----SRNSSLSSLSDIDQENNN    1981
Odobenus.rosmarus.divergens         -PQSSKDIPDRGAATDEKL-------QNFAIENTPVCF----SRNSSLSSLSDIDQENNN    1981
Taeniopygia.guttata                 -SQSTKDIPDRGAATDEKM-------QNFAIENTPVCF----SRNSSLSSLSDIDQENNN    1974
Anolis.carolinensis                 -SQSSKDIVDRIAATDEKM-------QNFAIENTPVCF----SRNSSLSSLSDIDQENNN    1979
Gallus.gallus                       -CQPSKDIPDRGAATDEKM-------QNFAIENTPVCF----SRNSSLSSLSDIDQENNN    1985
                                                    :          .  :.  *  :    *::** *:*.

Ciona.intestinalis                  -------EEAASASQHYGEDLINVVKDNPLNFGVEDTPFHLSGNNSSLSSIEIDSDGE--    1888
Strongylocentrotus.purpuratus       DLTEQWHDDEESQPQRSSGGANGINKDSRRVFATEDTPVCFSR-NSSLSSLDAESDGDAA    1993
Danio.rerio                         KDCSHK-----DDVTQMEAPRPQASGYAPKAFHVEDTPVCFSR-NSSLSSLSIDS-----    1981
Xenopus.tropicalis                  NKETEPIKQTEMTETQIGLRRPQTSGYAPKSFHVEDTPVCFSR-NSSLSSLSIDS-----    2035
Xenopus.laevis                      NKETEPLKQTGTSETQLGLRRPQTSGYAPKSFHVEDTPVCFSR-NSSLSSLSIDS-----    2031
Ornithorhynchus.anatinus            NKESEPIKPPEPPETQIESSRPQASGYAPKSFHVEDTPVCFSR-NSSLSSLSIDS-----    2035
Monodelphis.domestica               NKESEPTKETEPPDSQGEPNRPQTSGYAPKSFHVEDTPVCFSR-NSSLSSLSIDS-----    2032
Mus.musculus                        NKESEPIKEAEPANSQGEPSKPQASGYAPKSFHVEDTPVCFSR-NSSLSSLSIDS-----    2033
Rattus.norvegicus                   NEETGPVRDAEPANAQGQPGKPQASGYAPKSFHVEDTPVCFSR-NSSLSSLSIDS-----    2033
Sorex.araneus                       SKENEPIKERETPDSQGEPSKPQASGYAPKSFHVEDTPVCFSR-NSSLSSLSIDS-----    2029
Octodon.degus                       NKENEPIKETEPPDSQGEPSKPQASGYAPKSFHVEDTPVCFSR-NSSLSSLSIDS-----    2033
Heterocephalus                      NKENEPIKETEPPDSQGGPSKPQASGYAPKSFHVEDTPVCFSR-NSSLSSLSIDS-----    2031
Jaculus.jaculus                     SKENEPVKETEPPDSQGETSKPQVSGYAPKSFHVEDTPVCFSR-NSSLSSLSIDS-----    2033
Loxodonta.africana                  NKENEPVKETEPPDSQGEPSKPQASGYAPKSFHVEDTPVCFSR-NSSLSSLSIDS-----    2027
Trichechus.manatus.latirostris      NKENEPIKETEPPDSQGEPSKPQASGYAPKSFHVEDTPVCFSR-NSSLSSLSIDS-----    2029
Otolemur.garnettii                  NKENEPMKETEPPDSQGEPNKPQASGYAPKSFHVEDTPVCFSR-NSSLSSLSIDS-----    2035
Callithrix.jacchus                  NKENEPIKETEPPDSQGEPSKPQASGYAPKSFHVEDTPVCFSR-NSSLSSLSIDS-----    2034
Macaca.mulatta                      NKENEPIKETEPPDSQGEPSKPQASGYAPKSFHVEDTPVCFSR-NSSLSSLSIDS-----    2035
Nomascus.leucogenys                 NKENEPIKETEPTDSQGEPSKPQASGYAPKSFHVEDTPVCFSR-NSSLSSLSIDS-----    2035
Pongo.abelii                        NKENEPVKETEPPDSQGEPSKPQAAGYAPKSFHVEDTPVCFSR-NSSLSSLSIDS-----    2035
Homo.sapiens                        NKENEPIKETEPPDSQGEPSKPQASGYAPKSFHVEDTPVCFSR-NSSLSSLSIDS-----    2034
Gorilla.gorilla                     NKENEPIKETEPPDSQGEPSKPQASGYAPKSFHVEDTPVCFSR-NSSLSSLSIDS-----    2035
Pan.troglodytes                     NKENEPIKETEPPDSQGEPSKPQASGYAPKSFHVEDTPVCFSR-NSSLSSLSIDS-----    2035
Pan.paniscus                        NKENEPIKETEPPDSQGEPSKPQASGYAPKSFHVEDTPVCFSR-NSSLSSLSIDS-----    2035
Canis.lupus.familiaris              NKENEPLKEAEPPDSQGEPSKPQASGYAPKSFHVEDTPVCFSR-NSSLSSLSIDS-----    2035
```

```
Bos.taurus                          NKENEPVKETEPPASQGEPGKPQASGYAPKSFHVEDTPVCFSR-NSSLSSLSIDS-----    2041
Ovis.aries                          NKENEPIKETEPPASQGEPGKPQASGYAPKSFHVEDTPVCFSR-NSSLSSLSIDS-----    2042
Orcinus.orca                        NKENEPIKETEPPDSQGEPSKPQASGYAPKSFHVEDTPVCFSR-NSSLSSLSIDS-----    2038
Sus.scrofa                          NKESEPIKETEPPHSQGEPSKPQASGYAPKSFHVEDTPVCFSR-NSSLSSLSIDS-----    2038
Ceratotherium.simum.simum           NKENEPSKETEPPDSQGEPSKPQASGYAPKSFHVEDTPVCFSR-NSSLSSLSIDS-----    2036
Equus.caballus                      NKENEPVKETEPPDSQGEPSKPQASGYAPKSFHVEDTPVCFSR-NSSLSSLSIDS-----    2036
Felis.catus                         NKEGEPVKETEPPDSQGEPSKPQASGYAPKSFHVEDTPVCFSR-NSSLSSLSIDS-----    2036
Ailuropoda.melanoleuca              NKANEPIKETEPPDSQGEPSKPQASGYAPKSFHVEDTPVCFSR-NSSLSSLSIDS-----    2035
Mustela.putorius.furo               NKENEPIKETEPPNSQGEPSKPQASGYAPKSFHVEDTPVCFSR-NSSLSSLSIDS-----    2035
Odobenus.rosmarus.divergens         NKENEPMKETEPPDSQGEPSKPQAPGYAPKSFHVEDTPVCFSR-NSSLSSLSIDS-----    2035
Taeniopygia.guttata                 NKEGEPAKCPEAPEPQVESNRPQTSGYAPKSFHVEDTPVCFSR-NSSLSSLSIDS-----    2028
Anolis.carolinensis                 NKESEPVEQMEPSDAQIESNRPQVSGYAPKSFHVEDTPVCFSR-NSSLSSLSIDS-----    2033
Gallus.gallus                       NKEGEPVKRTEAPDSQIESSRPQTSGYAPKSFHVEDTPVCFSR-NSSLSSLSIDS-----    2039
                                                   :               * .****. :*  ******:. :*

Ciona.intestinalis                  -GNDLLNACISSAIPSATYNMNPPPEPMH-----------SH---------YNENHHRPS    1927
Strongylocentrotus.purpuratus       SEQALLDECITSGMPQSKVKKPVKRIN------------GKIISGGPSS-----------    2030
Danio.rerio                         -EDDLLQECISSAMPKKKKQTPRSKTEESGVKEEKSMMADGILSEEPDLILDLTDTHSPI    2040
Xenopus.tropicalis                  -EDDLLQECISSAMPKKKKPSKAKNEGE----KSSSNSVGGILAEEPDLTLDLRDLQSPD    2090
Xenopus.laevis                      -EDDLLQECISSAMPKKRKPSKIKNEVG----KSRSNSVGGILAEEPDLTLDLRDIQSPD    2086
Ornithorhynchus.anatinus            -EDDLLQECISSAMPKKKKPSRLKGDNE----KNSSRNLGGILAE--DLTLDLRDIQRPD    2088
Monodelphis.domestica               -EDDLLQECISSAMPKKKRPSRFKGDDE----KPSPRNMDGILAE--DLTLDLRDIQRPD    2085
Mus.musculus                        -EDDLLQECISSAMPKKKRPSRLKSESE----KQSPRKVGGILAE--DLTLDLKDLQRPD    2086
Rattus.norvegicus                   -EDDLLRECISSAMPKKRRPSRLKGEGE----WQSPRKVGSVLAE--DLTLDLKDIQRPE    2086
Sorex.araneus                       -EDDLLQECISSAMPKKKKPSRPKGDNE----KHSPRNMGGILAE--DLTLDLKDIERPD    2082
Octodon.degus                       -EDDLLQECISSAMPKKKRPSRLKGDNE----KHSPRNMGGILAE--DLTLDLKDIQRPD    2086
Heterocephalus                      -EDDLLQECISSAMPKKKRPSRLKGDNE----KHSPRNMGSLLAE--DLTLDLKDIQRPD    2084
Jaculus.jaculus                     -EDDLLQECISSAMPKKKRPPRLRADNE----KPSPRSMGGILAE--DLTLDLKDIQRPD    2086
Loxodonta.africana                  -EDDLLQECISSAMPKKKKPSRLKGDNE----KHSPRNMGGILAE--DLTLDLKDTQRPD    2080
Trichechus.manatus.latirostris      -EDDLLQECISSAMPKKKKPSRLKGDNE----KHSPRNMGGILAE--DLTLDLKDTQRPD    2082
Otolemur.garnettii                  -EDDLLQECISSAMPKKKKPSRLKGDNE----KHSPRNMGGILAE--DLTLDLKDIQRPD    2088
Callithrix.jacchus                  -EDDLLQECISSAMPKKKKPSRLKGDSE----KHSPRNMGGILAE--DLTLDLKDIQRPD    2087
Macaca.mulatta                      -EDDLLQECISSAMPKKKKPSRLKGDNE----KHSPRNMGGMLAE--DLTLDLKDIQRPD    2088
Nomascus.leucogenys                 -EDDLLQECISSAMPKKKKPSRLKGDNE----KHSPRNMGGILAE--DLTLDLKDIQRPD    2088
Pongo.abelii                        -EDDLLQECISSAMPKKKKPSRLKGDNE----KHSPRNMGGILAE--DLTLDLKDIQRPD    2088
Homo.sapiens                        -EDDLLQECISSAMPKKKKPSRLKGDNE----KHSPRNMGGILGE--DLTLDLKDIQRPD    2087
Gorilla.gorilla                     -EDDLLQECISSAMPKKKKPSRLKGDHE----KHSPRNMGGILAE--DLTLDLKDIQRPD    2088
Pan.troglodytes                     -EDDLLQECISSAMPKKKKPSRLKGDHE----KYSPRNMGGILAE--DLTLDLKDIQRPD    2088
Pan.paniscus                        -EDDLLQECISSAMPKKKKPSRLKGDHE----KHSPRNMGGILAE--DLTLDLKDIQRPD    2088
Canis.lupus.familiaris              -EDDLLQECISSAMPKKKKPSRLKGDTE----KHSPRNMSGILAE--DLTLDLKDIQRPD    2088
Bos.taurus                          -EDDLLQECISSAMPKKKKPSRLKPDNE----KHSPRNMGGILAE--DLTLDLKDIQRPD    2094
Ovis.aries                          -EDDLLQECISSAMPKKKKPSRLKPDNE----KHSPRNMSGILAE--DLTLDLKDIQRPD    2095
Orcinus.orca                        -EDDLLQECISSAMPKKKKPSRLKADNE----KHSPRNMGGILAE--DLTLDLKDIQRPD    2091
Sus.scrofa                          -EDDLLQECISSAMPKKKKPSRLKGDNE----KHSPRNMSGILAE--DLTLDLKDIQRPD    2091
Ceratotherium.simum.simum           -EDDLLQECISSAMPKKKKPSRLKGDNE----KHSPRNMGGILAE--DLTLDFKDVQRPD    2089
Equus.caballus                      -EDDLLQECISSAMPKKKKPSRFKGDNE----KHSPRNMGGILAE--DLTLDLKDIQRPN    2089
Felis.catus                         -EDDLLQECISSAMPKKKKPSRLKGDNE----KHSPRNMSGILAE--DLTLDLKDIQRPD    2089
Ailuropoda.melanoleuca              -EDDLLQECISSAMPKKKKPSRLKGDNE----KHSPRNMSGILAE--DLTLDLKDIQRPD    2088
Mustela.putorius.furo               -EDDLLQECISSAMPKKKKPSRLKGDNE----KHSPRNMSGILAE--DLTLDLKDIQRPD    2088
Odobenus.rosmarus.divergens         -EDDLLQECISSAMPKKKKPSRLKGDNE----KHSPRNMSGILAE--DLTLDLKDIQKPD    2088
Taeniopygia.guttata                 -EDDLLQECISSAMPKKKKPSRMKSDGE----KNNSRNSGGILAE--DLTLDLREIQRPD    2081
Anolis.carolinensis                 -EDDLLQECISSAMPKKKKPSRAKGDSE----KNSGRNMGGILAE--DLTLDLKDIQRPD    2086
Gallus.gallus                       -EDDLLQECISSAMPKKKKPSRIKSESE----KSNSRNIGGMLAE--DLTLDLREIQRPD    2092
                                      : **  **:*.:*.

Ciona.intestinalis                  TLQADSTYQSNAIWVEMNKGIPQGQHLLNAISPLSLVSLTSPDPTSNSFSITSAGTGTQT    1987
Strongylocentrotus.purpuratus       -----------------------M------------------------------------    2031
Danio.rerio                         SEQALSPDSESFDWKAIQEGANSIVSSLH-----------------------------QA    2071
Xenopus.tropicalis                  SENAFSPDSENFDWKAIQEGANSIVSRLH-----------------------------QA    2121
Xenopus.laevis                      SENAFSPDSENFDWKAIQEGANSIVSRLH-----------------------------QA    2117
Ornithorhynchus.anatinus            SEHGFSPDSENFDWKAIQEGANSIVSSLH-----------------------------QA    2119
Monodelphis.domestica               SEHGFSPDSENFDWKAIQEGANSIVSSLH-----------------------------QA    2116
Mus.musculus                        SEHAFSPDSENFDWKAIQEGANSIVSSLH-----------------------------QA    2117
Rattus.norvegicus                   SEHGLSPDSENFDWKAIQEGANSIVSSLH-----------------------------QA    2117
Sorex.araneus                       SEHGLSPDSETFDWKAIQEGANSIVSSLH-----------------------------QA    2113
```

```
Octodon.degus                       SEHGLSPDSENFDWKAIQEGANSIVSSLH-----------------------------QA    2117
Heterocephalus                      SEHGLSPDSENFDWKAIQEGANSIVSSLH-----------------------------QA    2115
Jaculus.jaculus                     SEHGLSPDSENFDWKAIQEGANSIVSSLH-----------------------------QA    2117
Loxodonta.africana                  SEHGLSPDSENFDWKAIQEGANSIVSSLH-----------------------------QA    2111
Trichechus.manatus.latirostris      SEHGLSPDSENFDWKAIQEGANSIVSSLH-----------------------------QA    2113
Otolemur.garnettii                  SEHGLSPDSENFDWKAIQEGANSIVSSLH-----------------------------QA    2119
Callithrix.jacchus                  SEHGLSPDSESFDWKAIQEGANSIVSSLH-----------------------------QA    2118
Macaca.mulatta                      SEHGLSPDSENFDWKAIQEGANSIVSSLH-----------------------------QA    2119
Nomascus.leucogenys                 SEHGLSPDSENFDWKAIQEGANSIVSSLH-----------------------------QA    2119
Pongo.abelii                        SEHGLSPDSENFDWKAIQEGANSIVSSLH-----------------------------QA    2119
Homo.sapiens                        SEHGLSPDSENFDWKAIQEGANSIVSSLH-----------------------------QA    2118
Gorilla.gorilla                     SEHGLSPDSENFDWKAIQEGANSIVSSLH-----------------------------QA    2119
Pan.troglodytes                     SEHGLSPDSENFDWKAIQEGANSIVSSLH-----------------------------QA    2119
Pan.paniscus                        SEHGLSPDSENFDWKAIQEGANSIVSSLH-----------------------------QA    2119
Canis.lupus.familiaris              SEHGLSPDSENFDWKAIQEGANSIVSSLH-----------------------------QA    2119
Bos.taurus                          SEHGLSPDSENFDWKAIQEGANSIVSSLH-----------------------------QA    2125
Ovis.aries                          SEHGLSPDSENFDWKAIQEGANSIVSSLH-----------------------------QA    2126
Orcinus.orca                        SEHGLSPDSENFDWKAIQEGANSIVSSLH-----------------------------QA    2122
Sus.scrofa                          SEHGLSPDSENFDWKAIQEGANSIVSSLH-----------------------------QA    2122
Ceratotherium.simum.simum           SEHGLSPDSENFDWKAIQEGANSIVSSLH-----------------------------QA    2120
Equus.caballus                      SEHGLSPDSENFDWKAIQEGANSIVSSLH-----------------------------QA    2120
Felis.catus                         SEHGLSPDSENFDWKAIQEGANSIVSSLH-----------------------------QA    2120
Ailuropoda.melanoleuca              SEHGLSPDSENFDWKAIQEGANSIVSSLH-----------------------------QA    2119
Mustela.putorius.furo               SEHGLSPDSENFDWKAIQEGANSIVSSLH-----------------------------QA    2119
Odobenus.rosmarus.divergens         SEHGLSPDSENFDWKAIQEGANSIVSSLH-----------------------------QA    2119
Taeniopygia.guttata                 SEHGFSPDSENFDWKAIQEGANSIVSSLH-----------------------------QA    2112
Anolis.carolinensis                 SEHGFSPDSENFDWKAIQEGANSIVSSLH-----------------------------QA    2117
Gallus.gallus                       SEHGFSPDSENFDWKAIQEGANSIVSSLH-----------------------------QA    2123


Ciona.intestinalis                  NEDYYRLSRQPSVG-D---SIGSEISIGSLV-LGGDAKSLVKTQNAGRPKISKG-KD--V    2039
Strongylocentrotus.purpuratus       ---------------------------EDDDSSPANQENEPKPVRKG-PRITKPSASVEE    2063
Danio.rerio                         A---ASLSRQGSSDSDSILSLKSGISIGSPFHLPLNQDDKPA-PNKG-PRILKPGEKSSI    2126
Xenopus.tropicalis                  A-AAGSLSRQGSSDSDSILSLKSGISLGSPFHLTSDKEEKTITCNKG-PKIVKPSDKSAL    2179
Xenopus.laevis                      A-AAGSLSRQGSSDSDSILSLKSGISLGSPFHLTLDKEEKTITSNKG-PKILKPAEKSAL    2175
Ornithorhynchus.anatinus            A-AAACLSRQASSDSDSILSLKSGISLGSPFHLTPDQEEKPFTSNKG-PRILKPGEKSTL    2177
Monodelphis.domestica               A-AAACLSRQASSDSDSILSLKSGISLGSPFHLTPDQEEKPFTSNKG-PRILKPGEKSTL    2174
Mus.musculus                        AAAAACLSRQASSDSDSILSLKSGISLGSPFHLTPDQEEKPFTSNKG-PRILKPGEKSTL    2176
Rattus.norvegicus                   AAAAACLSRQASSDSDSILSLKSGVSLGSPFHLTPDQEEKPFTSHKG-PRILKPGEKSTL    2176
Sorex.araneus                       A-AAACLSRQASSDSDSILSLKSGISLGSPFHLTPDQEEKPFTSNKG-PRILKPGEKSTL    2171
Octodon.degus                       A-AAACLSRQASSDSDSILSLKSGISLGSPFHLTPDQEEKPFTSNKG-PRILKPGEKSTL    2175
Heterocephalus                      A--AACLSRQASSDSDSILSLKSGISLGSPFHLTPDQEEKPFTSNKG-PRILKPGEKSTL    2172
Jaculus.jaculus                     A-AAACLSRQASSDSDSILSLKSGISLGSPFHLTPDQEEKPFTSNKG-PRILKPGEKSTS    2175
Loxodonta.africana                  A-AAACLSRQASSDSDSILSLKSGISLGSPFHLTPDQEEKPFTSNKG-PRILKPGEKSTL    2169
Trichechus.manatus.latirostris      A-AAACLSRQASSDSDSILSLKSGISLGSPFHLTPDQEEKPFTSNKG-PRILKPGEKSTL    2171
Otolemur.garnettii                  A-AAACLSRQASSDSDSILSLKSGISLGSPFHLTPDQEEKPFTSNKG-PRILKPGEKSTL    2177
Callithrix.jacchus                  A-AAACLSRQASSDSDSILSLKSGISLGSPFHLTPDQEEKPFTSNKG-PRILKPGEKSTL    2176
Macaca.mulatta                      A-AAACLSRQASSDSDSILSLKSGISLGSPFHLTPDQEEKPFTSNKG-PRILKPGEKSTL    2177
Nomascus.leucogenys                 A-AAACLSRQASSDSDSILSLKSGISLGSPFHLTPDQEEKPFTSNKG-PRILKPGEKSTL    2177
Pongo.abelii                        A-AAACLSRQASSDSDSILSLKSGISLGSPFHLTPDQEEKPFTSNKG-PRILKPGEKSTL    2177
Homo.sapiens                        A-AAACLSRQASSDSDSILSLKSGISLGSPFHLTPDQEEKPFTSNKG-PRILKPGEKSTL    2176
Gorilla.gorilla                     A-AAACLSRQASSDSDSILSLKSGISLGSPFHLTPDQEEKPFTSNKG-PRILKPGEKSTL    2177
Pan.troglodytes                     A-AAACLSRQASSDSDSILSLKSGISLGSPFHLTPDQEEKPFTSNKG-PRILKPGEKSTL    2177
Pan.paniscus                        A-AAACLSRQASSDSDSILSLKSGISLGSPFHLTPDQEEKPFTSNKG-PRILKPGEKSTL    2177
Canis.lupus.familiaris              A-AAACLSRQASSDSDSILSLKSGISLGSPFHLTPDQEEKPFTSNKG-PRILKPGEKSTL    2177
Bos.taurus                          A-AAACLSRQASSDSDSILSLKSGISLGSPFHLTPDQEEKPFTSNKG-PRILKPGEKSTL    2183
Ovis.aries                          A-AAACLSRQASSDSDSILSLKSGISLGSPFHLTPDQEEKPFMNNKG-PRILKPGEKSTL    2184
Orcinus.orca                        A-AAACLSRQASSDSDSILSLKSGISLGSPFHLTPDQEEKPFASNKG-PRILKPGEKSTL    2180
Sus.scrofa                          A-AAACLSRQASSDSDSILSLKSGISLGSPFHLTPDQEEKPFTSNKG-PRILKPGEKSTL    2180
Ceratotherium.simum.simum           A-AAACLSRQASSDSDSILSLKSGISLGSPFHLTPDQEEKPFTSNKG-PRILKPGEKSTL    2178
Equus.caballus                      A-AAACLSRQASSDSDSILSLKSGISLGSPFHLTPDQEEKPFTSNKG-PRILKPGEKSTL    2178
Felis.catus                         A-AAACLSRQASSDSDSILSLKSGISLGSPFHLTPDQEEKPFTSNKG-PRILKPGEKSTL    2178
Ailuropoda.melanoleuca              A-AAACLSRQASSDSDSILSLKSGISLGSPFHLTPDQEEKPFTSNKG-PRILKPGEKSTL    2177
Mustela.putorius.furo               A-AAACLSRQASSDSDSILSLKSGISLGSPFHLTPDQEEKPFTSNKG-PRILKPGEKSTL    2177
Odobenus.rosmarus.divergens         A-AAACLSRQASSDSDSILSLKSGISLGSPFHLTPDQEEKPFTSNKG-PRILKPGEKSTL    2177
```

```
Taeniopygia.guttata                A-AAASLSRQASSDSDSILSLKSGISLGSPFHLTPDQEEKPFTSNKG-PRIIKPGEKSTL    2170
Anolis.carolinensis                A-AAASLSRQASSDSDSILSLKSGISLGSPFHLTPDQEDKPFTSNKG-PRIIKPGEKSTL    2175
Gallus.gallus                      A-AAASLSRQASSDSDSILSLKSGISLGSPFHLTPDQEEKPFTSNKG-PRILKPGEKSTL    2181
                                                               .      : ..     . * *:* *   .

Ciona.intestinalis                 ERV-SSDDKPPVVKGGKKIYRSPITGKPRENLRYPNKHKDNT---------------STG    2083
Strongylocentrotus.purpuratus      KSVNEEEEGPKGVKGGKKIYRSPITGKIRSITPPKSVLPPKSPSSTRGGLAKGSPTTSRG    2123
Danio.rerio                        EAKKKEEETAKSLKGGKKVYKSLITGKPRPSLE--SMAS--------QHRQAQAPVISRG    2176
Xenopus.tropicalis                 ENKKTED-EPKGIKGGKKVYKSLITGKSRSSSDFSSHCK--------QSVQTNMPSISRG    2230
Xenopus.laevis                     ENKKTEE-EPKGIKGGKKVYKSLITGKSRSSSDFSSHCK--------QSVQTNMPSISRG    2226
Ornithorhynchus.anatinus           ESKKVES-ENKGIKGGKKVYKSLITGKVRSNSELSSQLK--------QPLPTNMPSISRG    2228
Monodelphis.domestica              ETKKIES-ENKGIKGGKKVYKSLITGKVRSNSEVSGQLK--------QPLPTNMPSISRG    2225
Mus.musculus                       EAKKIES-ENKGIKGGKKVYKSLITGKIRSNSEISSQMK--------QPLPTNMPSISRG    2227
Rattus.norvegicus                  EAKKIES-ENKGIKGGKKVYKSLITGKIRSNSEISSQMK--------QPLQTNMPSISRG    2227
Sorex.araneus                      ETKKIES-ENKGIKGGKKVYKSLITGKVRSHSEVSSQIK--------QPLQTNMPSISRG    2222
Octodon.degus                      ETKKMES-ENKGIKGGKKVYKSLITGKVRSNSEISSQMK--------QPIQTNMPSISRG    2226
Heterocephalus                     ETKKMES-ENKGIKGGKKVYKSLITGKVRSNSEISSQMK--------QPLQTNMPSISRG    2223
Jaculus.jaculus                    EAKKIES-ENKGIKGGKKVYKSLITGKVRSNSEVSSQMK--------QPLQTGMPSISRG    2226
Loxodonta.africana                 ETKKIES-ENKGIKGGKKVFKSLITGKVRSNSEVSSQMK--------QPLQTNMPSISRG    2220
Trichechus.manatus.latirostris     ETKKIES-ENKGIKGGKKVYKSLITGKVRSNSEVSSQMK--------QPLQTNMPSISRG    2222
Otolemur.garnettii                 ETKKIES-ENKGIKGGKKVYKSLITGKVRSNSEVASQMK--------QPLQTNMPSISRG    2228
Callithrix.jacchus                 ETKKIES-ESKGIKGGKKVYKSLITGKVRSNSEISGQMK--------QPLQANMPSISRG    2227
Macaca.mulatta                     ETKKIES-ESKGIKGGKKVYKSLITGKVRSNSEISGQMK--------QPLQANMPSISRG    2228
Nomascus.leucogenys                ETKKIES-ESKGIKGGKKVYKSLITGKVRSNSEISGQMK--------QPLQANMPSISRG    2228
Pongo.abelii                       ETKKIES-ESKGIKGGKKVYKSLITGKVRSNSEISGQMK--------QPLQANMPSISRG    2228
Homo.sapiens                       ETKKIES-ESKGIKGGKKVYKSLITGKVRSNSEISGQMK--------QPLQANMPSISRG    2227
Gorilla.gorilla                    ETKKIES-ESKGIKGGKKVYKSLITGKVRSNSEISGQMK--------QPLQANMPSISRG    2228
Pan.troglodytes                    ETKKIES-ESKGIKGGKKVYKSLITGKVRSNSEISGQMK--------QPLQANMPSISRG    2228
Pan.paniscus                       ETKKIES-ESKGIKGGKKVYKSLITGKVRSNSEISGQMK--------QPLQANMPSISRG    2228
Canis.lupus.familiaris             ETKKIES-ENKGIKGGKKVYKSLITGKIRSNSEVLSQMK--------QPLQANMPSISRG    2228
Bos.taurus                         ETKKIES-ENKGIKGGKKVYKSLITGKVRSNSEISSQMK--------QPLQTNMPSISRG    2234
Ovis.aries                         ETKKIES-ENKGIKGGKKVYKSLITGKVRSNSEISSQMK--------QPLPTNMPSISRG    2235
Orcinus.orca                       EAKKLES-ENKGIKGGKKVYRSLITGKIRSNSEVSSQMK--------QPLQTNMPSISRG    2231
Sus.scrofa                         ETKKMES-ENKGIKGGKKVYKSLITGKVRSNSEISSQMK--------QPLQTNMPSISRG    2231
Ceratotherium.simum.simum          ETKKIES-ENKGIKGGKKVYKSLITGKVRSNSEISGQMK--------QPLPTNMPSISRG    2229
Equus.caballus                     ETKKIES-ENKGIKGGKKVYKSLITGKVRSNSEISGQMK--------QPLQTNMPSISRG    2229
Felis.catus                        ETKKIES-ENKGIKGGKKVYRSLITGKVRSNSEISSQMK--------QPLQTNMPSISRG    2229
Ailuropoda.melanoleuca             ETKKIES-ENKGIKGGKKVYKSLITGKVRSHSEVSSQMK--------QPLQANMPSISRG    2228
Mustela.putorius.furo              ETKKIES-ENKGIKGGKKVYKSLLTGKVRSNSEISNQMK--------QSLQANMPSISRG    2228
Odobenus.rosmarus.divergens        ETKKIES-ENKGIKGGKKVYKSLITGKVRSNSEVSGQMK--------QPLQANMPSISRG    2228
Taeniopygia.guttata                ESKKVES-ESRGIKGGKKVYKSMITGKARSNSEVS-SLK--------QPQQTSVPSISRG    2220
Anolis.carolinensis                ETKKVEP-ENKGIKGGKKIYKSLITGKARSNSELTSHLK--------QPLQTNMPSISRG    2226
Gallus.gallus                      ESKKVES-ESKGIKGGKRVYKSIITGKARSNSEVSSQIK--------QPQQTSVPSISRG    2232
                                   :    .      :****::::* :*** *                            * *

Ciona.intestinalis                 SSSARIREHPRNNRNSGRSKSPC-----NKCHPPHQSR------QLPISSSMGKLAE---    2129
Strongylocentrotus.purpuratus      RGAIRG--ARGGFARSASTTPPRSSTPTGRGTPPRTTTPPRTTSPMTAGRTTTPPRTTSP    2181
Danio.rerio                        RTMVHV--P--GVRSSSPSTSPV-----PKKPPPRG--------QMSKPPSQAPGAGSSP    2219
Xenopus.tropicalis                 RTMIHI--P--GVRASSPSTSPV-----SKKGPVFKN-------APSKSPNENQSLSNSP    2274
Xenopus.laevis                     RTMIHI--P--GVRASSPSTSPV-----SKKGPVFKN-------VPSKGSNENPSSSSSP    2270
Ornithorhynchus.anatinus           RTMIHI--P--GVRNSSSSTSPV-----AKKAPPLKA-------PASKSPNEGQPATTSP    2272
Monodelphis.domestica              RTMIHI--P--GIRNSSSSTSPV-----SKKGPSLKT-------PTSKSPSEGPTSSTSP    2269
Mus.musculus                       RTMIHI--P--GLRNSSSSTSPV-----SKKGPPLKT-------PASKSPSEGPGATTSP    2271
Rattus.norvegicus                  RTMIHI--P--GVRNSSSSTSPV-----SKKGPPLKT-------PASKSPSEGPVATTSP    2271
Sorex.araneus                      RTMIHI--P--GVRNSSSSTSPV-----SKKGPPVKT-------TSSKSPSEGQTVATSP    2266
Octodon.degus                      RTMIHI--P--GVRNSSSSTSPV-----SKKGPPLKT-------PASKSPSEGQTATTSP    2270
Heterocephalus                     RTMIHI--P--GVRNSSSSTSPV-----SKKGPPLKT-------PASKSPSEAQTATTSP    2267
Jaculus.jaculus                    RTMIHI--P--GVRNSSSSTSPV-----SKKGPPLKT-------PASKSPSEGQTATTSP    2270
Loxodonta.africana                 RTMIHI--P--GVRNSSSSTSPV-----SKKGPPLKT-------PASKSPSESQTATTSP    2264
Trichechus.manatus.latirostris     RTMIHI--P--GVRNSSSSTSPV-----SKKGPPLKT-------PASKSPSESQTATTSP    2266
Otolemur.garnettii                 RTMIHI--P--GVRNSSSSTSPV-----SKKGPPLKT-------PPSKSPSEGQTATTSP    2272
Callithrix.jacchus                 RTMIHI--P--GVRNSSSSTSPV-----SKKGPPHKT-------PASKSPSEGQAATTSP    2271
Macaca.mulatta                     RTMIHI--P--GVRNSSSSTSPV-----SKKGPPLKT-------PASKSPSEGQTATTSP    2272
Nomascus.leucogenys                RTMIHI--P--GVRNSSSSTSPV-----SKKGPPLKT-------PASKSPSEGQTATTSP    2272
Pongo.abelii                       RTMIHI--P--GVRNSSSSTSPV-----SKKGPPLKT-------PASKSPSEGQTATTSP    2272
```

```
Homo.sapiens                    RTMIHI--P--GVRNSSSSTSPV-----SKKGPPLKT-------PASKSPSEGQTATTSP    2271
Gorilla.gorilla                 RTMIHI--P--GVRNSSSSTSPV-----SKKGPPLKT-------PASKSPSEGQTATTSP    2272
Pan.troglodytes                 RTMIHI--P--GVRNSSSSTSPV-----SKKGPPLKT-------PASKSPSEGQTATTSP    2272
Pan.paniscus                    RTMIHI--P--GVRNSSSSTSPV-----SKKGPPLKT-------PASKSPSEGQTATTSP    2272
Canis.lupus.familiaris          RTMIHI--P--GVRNSSSSTSPV-----SKKGPPLKT-------PASKSPSEGQTATTSP    2272
Bos.taurus                      RTMIHI--P--GVRNSSSSTSPV-----SKKGPPLKT-------PASKSPSEGQPATTSP    2278
Ovis.aries                      RTMIHI--P--GVRNSSSSTSPV-----SKKGPPLKT-------PASKSPSEGQPATTSP    2279
Orcinus.orca                    RTMIHI--P--GVRNSSSSTSPV-----SKKGPPLKT-------PASKSPSEGQAATTSP    2275
Sus.scrofa                      RTMIHI--P--GVRNSSSSTSPV-----SKKGPPLKT-------PASKSPSESQAATTSP    2275
Ceratotherium.simum.simum       RTMIHI--P--GVRNSSSSTSPV-----SKKGPPLKT-------PASKSPSEGQTATTSP    2273
Equus.caballus                  RTMIHI--P--GVRNSSSSTSPV-----SKKGPPLKT-------PASKSPSEGQTATTSP    2273
Felis.catus                     RTMIHI--P--GVRNSSSSTSPV-----SKKGPPLKT-------PASKSPSEGQPATTSP    2273
Ailuropoda.melanoleuca          RTMIHI--P--GVRNSSSSTSPV-----SKKGPPLKT-------PASKSPSEGQTATTSP    2272
Mustela.putorius.furo           RTMIHI--P--GVRNSSSSTSPV-----SKKGPPLKT-------PASKSPSEGQTTTTSP    2272
Odobenus.rosmarus.divergens     RTMIHI--P--GVRNSSSSTSPV-----SKKGPPLKT-------PASKSPSEGQTATTSP    2272
Taeniopygia.guttata             RTMIHI--P--GLRNSSSSTSPV-----SKKGPPLKN-------MNSKSPSEGQSLTSSP    2264
Anolis.carolinensis             RTMIHI--P--GVRNSSSSTSPV-----SKKGPQLKS-------MASKCPNETQSSTSSP    2270
Gallus.gallus                   RTMIHI--P--GVRNSSSSTSPV-----SKKGPPFKN-------TNSKSPSEGQSSASSP    2276
                                    :      .   *. :. *       :  *                 .

Ciona.intestinalis              -----------------------------------------------------------T    2130
Strongylocentrotus.purpuratus   RTTSPRSGTPPKPKGSIAN---KSITPVRNATNGARSITPPRPVRKSSVDSQKDGDAKSE    2238
Danio.rerio                     RTMKVPPSSEPSPASGPPS--SQGGSSKASSRSGSRDSTPSRPVQQSLTRPMQSPGRASV    2277
Xenopus.tropicalis              KGSKPLKSESF-YGSRQS--STSGGSSKGNSRSGSRDSVSSRPSSQQLSRPLQSPGRNSI    2331
Xenopus.laevis                  KGTKPLKSELV-YGSRPS--STPGGSSKGNSRSGSRDSASSRPSPQPLSRPLQSPGRNSI    2327
Ornithorhynchus.anatinus        RGAKPTIKSEASPVNRQPS--QAGGSSKGPSRSGSRDSTPSRPPQQPLSRPMQSPGRSSI    2330
Monodelphis.domestica           RGAKPSVKSELSPVTRQTS--QPGGSSKGPSRSGSRDSTPSRPSQQPLSRPMQSPGRNSI    2327
Mus.musculus                    RGTKPAGKSELSPITRQTS--QISGSNKGSSRSGSRDSTPSRPTQQPLSRPMQSPGRNSI    2329
Rattus.norvegicus               RGTKPAVKSELSPITRQTS--HISGSNKGPSRSGSRDSTPSRPTQQPLSRPMQSPGRNSI    2329
Sorex.araneus                   RGAKPPGKSDSSPVPKLTA--QAGGPNAAPSRSGSREPTPAGPAQPPLSRPVQSPGRNSI    2324
Octodon.degus                   RGAKPSVKSELSPITRQTS--QISGSNKGSSRSGSRDSTPSRPAQQPLSRPMQSPGRNSI    2328
Heterocephalus                  RGAKPSVKSELSPVTRQTS--QIGGSNKGSSRSGSRDSTPSRPAQQPLSRPMQSPGRNSI    2325
Jaculus.jaculus                 RGTKPSVKSELSPITRQTS--TAGCSNKGPSRSGSRDSTPSRPAQQPLTRPMQSPGRNSI    2328
Loxodonta.africana              RGAKPSVKSELSPVTRQTS--QPGGSNKGPSRSGSRDSTPSRPAQQPLSRPVQSPGRNSI    2322
Trichechus.manatus.latirostris  RGAKPSVKSELSPVTRQTS--QPSGSNKGPSRSGSRDSTPSRPAQQPLSRPMQSPGRNSI    2324
Otolemur.garnettii              RGTKPSMKSELSPVARQTS--QISGSNKGPSRSGSRDSTPSRPAQQPLSRPMQSPGRNSI    2330
Callithrix.jacchus              RGAKPSVKSELSPVARQTS--QIGGSSKAPSRSGSRDSTPSRPAQQPLSRPIQSPGRNSI    2329
Macaca.mulatta                  RGAKPSVKSELSPVARQTS--QIGGSSKAPSRSGSRDSTPSRPAQQPLSRPIQSPGRNSI    2330
Nomascus.leucogenys             RGAKPSVKSELSPVARQTS--QIGGSSKAPSRSGSRDSTPSRPAQQPLSRPIQSPGRNSI    2330
Pongo.abelii                    RGAKPSVKSELSPVARQTS--QIGGSSKAPSRSGSRDSTPSRPAQQPLSRPMQSPGRNSI    2330
Homo.sapiens                    RGAKPSVKSELSPVARQTS--QIGGSSKAPSRSGSRDSTPSRPAQQPLSRPIQSPGRNSI    2329
Gorilla.gorilla                 RGAKPSVKSELSPVARQTS--QIGGSSKAPSRSGSRDSTPSRPAQQPLSRPIQSPGRNSI    2330
Pan.troglodytes                 RGAKPSVKSELSPVARQTS--QIGGTSKAPSRSGSRDSTPSRPAQQPLSRPIQSPGRNSI    2330
Pan.paniscus                    RGAKPSVKSELSPVARQTS--QIGGTSKAPSRSGSRDSTPSRPAQQPLSRPIQSPGRNSI    2330
Canis.lupus.familiaris          RGTKPSVKSELSPVTRPAP--QPAGANKGPSRSGSRDSTPSRPAQQPLSRPMQSPGRNSI    2330
Bos.taurus                      RGTKPSVKSELSPVTRQAS--QTAGSNKGPSRSGSRDSTPSRPAQQPLSRPMQSPGRNSI    2336
Ovis.aries                      RGTKPSVKSELSPVTRQAS--QTAASNKGPSRSGSRDSTPSRPAQQPLSRPMQSPGRNSI    2337
Orcinus.orca                    RGAKPSVKSELSPVTRQAS--QTPGSNKGPSRSGSRDSTPSRPAQQPLSRPMQSPGRNSI    2333
Sus.scrofa                      RGAKPSVKSELSPVTRQTS--QTAGSNKGPSRSGSRDSTPSRPAQQPLSRPMQSPGRNSI    2333
Ceratotherium.simum.simum       RGAKPSVKSELSPVTKQTS--QPAGSNKGPSRSGSRDSTPSRPAQQPLSRPMQSPGRNSI    2331
Equus.caballus                  RGAKPSVKSELSPVTRQTS--QPAGSNKGPSRSGSRDSTPSRPAQQPLSRPMQSPGRNSI    2331
Felis.catus                     RGTKPSVKSELSPVTRQTS--QPAGANKGSSRSGSRDSTPSRPAQQPLSRPMQSPGRNSI    2331
Ailuropoda.melanoleuca          RGAKPSVKSELSPVTRQTS--QPAGANKGPSRSGSRDSTPSRPAQQPLSRPMQSPGRNSI    2330
Mustela.putorius.furo           RGAKPSVKSELSPVTRQTS--QPAVANKGPSRSGSRDSTPSRPAQQPLSRPMQSPGRNSI    2330
Odobenus.rosmarus.divergens     RGAKPSVKSELSPVTRQTS--QPAGANKGPSRSGSRDSTPSRPAQQPLSRPMQSPGRNSI    2330
Taeniopygia.guttata             RGAKSSVKPEPAPVTRQPSGLNQSGSSKGPSRSGSRDSTPSRPQQQPLSRPLQSPGRNSI    2324
Anolis.carolinensis             RVSKPSMKSEPSPISRQPS--QPGGSSKGTSRSGSRDSTPSRPQQQPLTRPLQSPGRNSI    2328
Gallus.gallus                   RGVKSSVKPEPAPVTRQLSGLNQGGSSKGPSRSGSRDSTPSRPQQQPLSRPLQSPGRNSI    2336


Ciona.intestinalis              SPGRSHRKTNAS-------------STYS---------KQMQPTCGGNPHGKPSRSRS--    2166
Strongylocentrotus.purpuratus   TSSR--RSSKESISSIP-KPRQIKPQSTQTKTSPNSSTSPKPPTGRGSPAGKPPLSTSRS    2295
Danio.rerio                     SPGRNGLSPSNKLSQLPQLPRTASPSASSTKS-----------SGSG----RMAY-----    2317
Xenopus.tropicalis              SPGRNGISPPNKFSQ---LPRTSSPSTASTKS-----------SGSG----RMSY-----    2368
Xenopus.laevis                  SPGKNGISPPNKFSQ---LPRTTSPSTASTKS-----------SGSG----RMSY-----    2364
```

```
Ornithorhynchus.anatinus            SPGRNGISPPNKLSQ---LPRTSSPSTASTKS-----------SGSG----RMAY-----    2367
Monodelphis.domestica               SPGRNGISPPNKLSQ---LPRTSSPSTSSTKS-----------SGSG----KISY-----    2364
Mus.musculus                        SPGRNGISPPNKLSQ---LPRTSSPSTASTKS-----------SGSG----KMSY-----    2366
Rattus.norvegicus                   SPGRNGISTPNKLSQ---LPRTSSPSTASTKS-----------SGSG----KMSY-----    2366
Sorex.araneus                       SPGRNGISP-NKFSQ---LPRTSSPSTASTKS-----------SGSG----KMLY-----    2360
Octodon.degus                       SPGRNGISPPNKLSQ---LPRTSSPSTISTKS-----------SGSG----KMSY-----    2365
Heterocephalus                      SPGRNGISPPNKLSQ---LPRTSSPSTVSTKS-----------SGSG----KMSY-----    2362
Jaculus.jaculus                     SPGRNGISPPNKLSQ---LPRTSSPSTASTKS-----------SGSG----KMSY-----    2365
Loxodonta.africana                  SPGRNGISPPNKLSQ---LPRTSSPSTASTKS-----------SGSG----KMSY-----    2359
Trichechus.manatus.latirostris      SPGRNGVSPPNKLSQ---LPRTSSPSTASTKS-----------SGSG----KMSY-----    2361
Otolemur.garnettii                  SPGRNGISPPNKLSQ---LPRTSSPSTASTKS-----------SGSG----KMSY-----    2367
Callithrix.jacchus                  SPGRNGISPPNKLSQ---LPRTSSPSTASTKS-----------SGSG----KMSY-----    2366
Macaca.mulatta                      SPGRNGISPPNKLSQ---LPRTSSPSTASTKS-----------SGSG----KMSY-----    2367
Nomascus.leucogenys                 SPGRNGISPPNKLSQ---LPRTSSPSTASTKS-----------SGSG----KMSY-----    2367
Pongo.abelii                        SPGRNGISPPNKLSQ---LPRTSSPSTASTKS-----------SGSG----KMSY-----    2367
Homo.sapiens                        SPGRNGISPPNKLSQ---LPRTSSPSTASTKS-----------SGSG----KMSY-----    2366
Gorilla.gorilla                     SPGRNGISPPNKLSQ---LPRTSSPSTASTKS-----------SGSG----KMSY-----    2367
Pan.troglodytes                     SPGRNGISPPNKLSQ---LPRTSSPSTASTKS-----------SGSG----KMSY-----    2367
Pan.paniscus                        SPGRNGISPPNKLSQ---LPRTSSPSTASTKS-----------SGSG----KMSY-----    2367
Canis.lupus.familiaris              SPGRNGISPPNKLSQ---LPRTSSPSTASTKS-----------SGSG----KMSY-----    2367
Bos.taurus                          SPGRNGISPPNKLSQ---LPRTSSPSTASTKS-----------SGSG----KMSY-----    2373
Ovis.aries                          SPGRNGISPPNKLSQ---LPRTSSPSTASTKS-----------SGSG----KMSY-----    2374
Orcinus.orca                        SPGRNGISPPNKLSQ---LPRTSSPSTASTKS-----------SGSG----KMSY-----    2370
Sus.scrofa                          SPGRNGISPPNKLSQ---LPRTSSPSTASTKS-----------SGSG----KMSY-----    2370
Ceratotherium.simum.simum           SPGRNGISPPNKLSQ---LPRTSSPSTASTKS-----------SGSG----KMSY-----    2368
Equus.caballus                      SPGRNGISPPNKLSQ---LPRTSSPSTASTKS-----------SGSG----KMSY-----    2368
Felis.catus                         SPGRNGISPPNKLSQ---LPRTSSPSTASTKS-----------SGSG----KMSY-----    2368
Ailuropoda.melanoleuca              SPGRNGISPPNKLSQ---LPRTSSPSTASTKS-----------SGSG----KMSY-----    2367
Mustela.putorius.furo               SPGRNGISPPNKLSQ---LPRTSSPSTASTKS-----------SGSG----KMSY-----    2367
Odobenus.rosmarus.divergens         SPGRNGISPPNKLSQ---LPRTSSPSTASTKS-----------SGSG----KMSY-----    2367
Taeniopygia.guttata                 SPGRNGISPPNKLSQ---LPRTSSPSTASTKS-----------SSSG----RMSY-----    2361
Anolis.carolinensis                 SPGRNGISPPNKLSQ---LPRTSSPSTASTKS-----------SGSG----RMAY-----    2365
Gallus.gallus                       SPGRNGISPPNKLSQ---LPRTSSPSTASTKS-----------SSSG----RMSY-----    2373
                                    : .:   .   .             .: .              :  *    :

Ciona.intestinalis                  AHAGYQSSVTQ-IYPPLKHVNSIMLNGVASMAARGD---LVVQQLE-----GKTAQV---    2214
Strongylocentrotus.purpuratus       SSPKSKMPVPSRSNSPSTKSSGSVTTPRKPL-NKSPSSTKARDESP-TDAEEVTSQMKSM    2353
Danio.rerio                         TSPGRQLVQPT-----PTKQSGLPRST-SGI-PRSESASKILNQ----CGPSKKAELSRM    2366
Xenopus.tropicalis                  TSPGRQLSQPN-----LSKQSGLPKTP-SSI-PRSESASKGLNQNVN-TGPNKKVELSRM    2420
Xenopus.laevis                      TSPGRQLSQPN-----LSKQSGLPKTH-SSI-PRSESASKSLNQNVN-TGSNKKVELSRM    2416
Ornithorhynchus.anatinus            TSPGRQMSQQN-----LAKPTGLSKNP-SSI-PRSESASKGLNQAAGGSGPNKKVELSRM    2420
Monodelphis.domestica               TSPGRQMSQQN-----LTKQTGLSKNT-SNI-PRSESASKGLNQISNSNGTNKKVELSRM    2417
Mus.musculus                        TSPGRQLSQQN-----LTKQASLSKNA-SSI-PRSESASKGLNQMSNGNGSNKKVELSRM    2419
Rattus.norvegicus                   TSPGRQLSQQN-----LSKQTGLSKNA-SSI-PRSESASKGLNQMNNSNGSNKKVELSRM    2419
Sorex.araneus                       TPPGRQMSQQS-----LTKQAGVSKNG-SAI-PRSESASKGLNQMSTSNGSNKKVELSRM    2413
Octodon.degus                       TSPGRQMGQQN-----F-KQTGLSKNT-GSI-TRSESASKGLNQMSNSNGSNKKVELSRM    2417
Heterocephalus                      TSPGRQMSQQN-----LTKQTGLSKNT-SSI-TRSESASKGLNQMSNSNGSNKKVELSRM    2415
Jaculus.jaculus                     TSPGRQLSQQN-----LTKQTGLSKNA-SSI-PRSESASKGLNQMNNGNGSNKKVELSRM    2418
Loxodonta.africana                  TSPGRQMSQQN-----LAKQTGFPKNG-SSI-PRSESASKGLNQMSNSNGSNKKVELSRM    2412
Trichechus.manatus.latirostris      TSPGRQMSQQN-----LTKQTGFPKNG-SSI-PRSESASKGLNQMNNSNGANKKVELSRM    2414
Otolemur.garnettii                  TSPGRQMSQQN-----LTKQTGLSKNG-SSI-PRSESASKGLNQMNNSNGSNKKVELSRM    2420
Callithrix.jacchus                  TSPGRQMSQQN-----LTKQTGLSKNA-SSI-PRSESASKGLNQMNNGNGANKKVELSRM    2419
Macaca.mulatta                      TSPGRQMSQQN-----LTKQTGLSKNA-SSI-PRSESASKGLNQVNNGNGANKKVELSRM    2420
Nomascus.leucogenys                 TSPGRQMSQQN-----LTKQTGLSKNA-SSI-PRSESASKGLNQMNNGNGANKKVELSRM    2420
Pongo.abelii                        TSPGRQMSQQN-----LTKQTGLSKNA-SSI-PRSESASKGLNQMNNGNGANKKVELSRM    2420
Homo.sapiens                        TSPGRQMSQQN-----LTKQTGLSKNA-SSI-PRSESASKGLNQMNNGNGANKKVELSRM    2419
Gorilla.gorilla                     TSPGRQMSQQN-----LTKQTGLSKNA-SSI-PRSESASKGLNQMNNGNGANKKVELSRM    2420
Pan.troglodytes                     TSPGRQMSQQN-----LTKQTGLSKNA-SSI-PRSESASKGLNQMNNGNGANKKVELSRM    2420
Pan.paniscus                        TSPGRQMSQQN-----LTKQTGLSKNA-SSI-PRSESASKGLNQMNNGNGANKKVELSRM    2420
Canis.lupus.familiaris              TSPGRQMSQQN-----LSKQTGLSKNG-SSI-PRSESASKGLNQMSNSNGSNKKVELSRM    2420
Bos.taurus                          TSPGRQMSQQN-----LTKQTGLSKNG-SGI-PRSESASKGLNQMSNSNGSNKKVELSRM    2426
Ovis.aries                          TSPGRQMSQQN-----LTKQTGLSKNG-SGI-PRSESASKGLNQMSSNNGANKKVELSRM    2427
Orcinus.orca                        TSPGRQMSQQN-----LTKQTGLSKNV-SSI-PRSESASKGLSQMSTSNGANKKVELSRM    2423
Sus.scrofa                          TSPGRQMSQTN-----LTKQTGLSKNG-SSI-PRSESASKGLNQMSCSNGSNKKVELSRM    2423
Ceratotherium.simum.simum           TSPGRQMSQQN-----LTKQTGLSKNG-SSI-PRSESASKGLNQMNNSNGSNKKVELSRM    2421
```

```
Equus.caballus                    TSPGRQMSQQN-----LTKQTGLSKNG-SSI-PRSESASKGLNQMNNSNGANKKVELSRM    2421
Felis.catus                       TSPGRQMSQQN-----LTKQTGLSKNG-SSI-PRSESASKGLNQMNNGNGSNKKVELSRM    2421
Ailuropoda.melanoleuca            TSPGRQMSQQN-----LTKQPGLSKNG-SSI-PRSESASKGLNQVNNSNGPNKKVELSRM    2420
Mustela.putorius.furo             TSPGRQMSQQN-----LTKQTGLSKNG-SSI-PRSESASKGLNQMNNSNGSNKKVELSRM    2420
Odobenus.rosmarus.divergens       TSPGRQMSQQN-----LTKQTGLSKNG-SSI-PRSESASKGLNQMNNSNGSNKKVELSRM    2420
Taeniopygia.guttata               TPPGRQMSQQN-----LTKQTALPKST-SSI-PRSESASKGLNQTLSTSGSNKKTDLSRM    2414
Anolis.carolinensis               TSPGRQLSQQS-----IAKQTGLPKST-SGI-PRSESASKGLNQMSGISGSSKKSDVSRM    2418
Gallus.gallus                     TSPGRQMSQQN-----LTKQTALTKNT-SSI-PRSESASKGLNQILGSGASNKKTDLSRM    2426
                                  :    :            :  .   .    :  :.       .:         . ::

Ciona.intestinalis                -VPASTLANQFQHVSRPVLIKQGTFIQDEPSQALLQSMSRKPEVPKKPAKSPP-KAVKSK    2272
Strongylocentrotus.purpuratus     HLESNGSSPDLDDGDRPVLLKQSTFTKDSASLPEQTP-----EMNPVSGDE--------K    2400
Danio.rerio                       -SSTKSSGSESDRSEKPGLVRQSTFIKEAPSPTLKRKLEESASFESLSPSST------SQ    2419
Xenopus.tropicalis                -SSTKSSGSESDRSERPALVRQSTFIKEAPSPTLRRKLEESASFESLSSSSRADSPTRSQ    2479
Xenopus.laevis                    -SSTKSSGSESDRSERPALVRQSTFIKEAPSPTLRRKLEESASFESLSSSSRADSPPRSQ    2475
Ornithorhynchus.anatinus          -SSTKSSGSESDRSERPVLVRQSTFIKEAPSPTLRRKLEESASFESLSPSSRPDSPTRSQ    2479
Monodelphis.domestica             -SSTKSSGSESDRSERPVLVRQSTFIKETPSPTLRRKLEESASLESLSSSSRPDSPTKSQ    2476
Mus.musculus                      -SSTKSSGSESDRSERPALVRQSTFIKEAPSPTLRRKLEESASFESLSPSSRPDSPTRSQ    2478
Rattus.norvegicus                 -SSTKSSGSESDRSERPALVRQSTFIKEAPSPTLRRKLEESASFESLSPSSRPDSPTRSQ    2478
Sorex.araneus                     -SSTKSSGSESDRSERPVLVRQSTFIKEAPSPTLRRKLEESASFESLSPSSRPGSPTRSQ    2472
Octodon.degus                     -SSTKSSGSESDRSERPVLVRQSTFIKEAPSPTLRRKLEESASFESLSPSSRPDSPTRSQ    2476
Heterocephalus                    -SSAKSSGSESDRSERPVLVRQSTFIKEAPSPTLRRKLEESASFESLSPSSRPDSPTRSQ    2474
Jaculus.jaculus                   -SSTKSSGSESDRSERPVLVRQSTFIKEAPSPTLRRKLEESASFESLSPSSRPDSPTRSQ    2477
Loxodonta.africana                -SSTKSSGSESDRSERPILVRQSTFIKEAPSPTLRRKLEESASFESLSPSSRPNSPTRSQ    2471
Trichechus.manatus.latirostris    -SSTKSSGSESDRSERPVLVRQSTFIKEAPSPTLRRKLEESASFESLSPSSRPNSPTRSQ    2473
Otolemur.garnettii                -SSTKSSGSESDRSERPILVRQSTFIKEAPSPTLRRKLEESASFESLSPSSRPDSPTRSQ    2479
Callithrix.jacchus                -SSTKSSGSESDRSERPVLVRQSTFIKEAPSPTLRRKLEESASFESLSPSSRPASPTRSQ    2478
Macaca.mulatta                    -SSTKSSGSESDRSERPVLVRQSTFIKEAPSPTLRRKLEESASFESLSPSSRPASPTRSQ    2479
Nomascus.leucogenys               -SSTKSSGSESDRSERPVLVRQSTFIKEAPSPTLRRKLEESASFESLSPSSRPASPSRSQ    2479
Pongo.abelii                      -SSTKSSGSESDRSERPVLVRQSTFIKEAPSPTLRRKLEESASFESLSPSSRPASPTRSQ    2479
Homo.sapiens                      -SSTKSSGSESDRSERPVLVRQSTFIKEAPSPTLRRKLEESASFESLSPSSRPASPTRSQ    2478
Gorilla.gorilla                   -SSTKSSGSESDRSERPVLVRQSTFIKEAPSPTLRRKLEESASFESLSPSSRPASPTRSQ    2479
Pan.troglodytes                   -SSTKSSGSESDRSERPVLVRQSTFIKEAPSPTLRRKLEESASFESLSPSSRPASPTRSQ    2479
Pan.paniscus                      -SSTKSSGSESDRSERPVLVRQSTFIKEAPSPTLRRKLEESASFESLSPSSRPASPTRSQ    2479
Canis.lupus.familiaris            -SSTKSSGSESDRSERPVLVRQSTFIKEAPSPTLRRKLEESASFESLSPSSRPDSPTRSQ    2479
Bos.taurus                        -SSTKSSGSESDRSERPVLVRQSTFIKEAPSPTLRRKLEESASFESLSPSSRPDSPTRSQ    2485
Ovis.aries                        -SSTKSSGSESDRSERPVLVRQSTFIKEAPSPTLRRKLEESASFESLSPSSRPDSPTRSQ    2486
Orcinus.orca                      -SSTKSSGSESDRSERPVLVRQSTFIKEAPSPTLRRKLEESASFESLSPSSRPDSPSRSQ    2482
Sus.scrofa                        -SSTKSSGSESDRSERPVLVRQSTFIKEAPSPTLRRKLEESASFESLSPSSRPDSPTRSQ    2482
Ceratotherium.simum.simum         -SSTKSSGSESDRSERPVLVRQSTFIKEAPSPTLRRKLEESASFESLSPSSRPDSPTRSQ    2480
Equus.caballus                    -SSTKSSGSESDRSERPVLVRQSTFIKEAPSPTLRRKLEESASFESLSPSSRPDSPTRSQ    2480
Felis.catus                       -SSTKSSGSESDRSERPVLVRQSTFIKEAPSPTLRRKLEESASFESLSPSSRPDSPTRSQ    2480
Ailuropoda.melanoleuca            -SSTKSSGSESDRSERPVLVRQSTFIKEAPSPTLRRKLEESASFESLSPSSRPDSPTRSQ    2479
Mustela.putorius.furo             -SSTKSSGSESDRSEKPVLVRQSTFIKEAPSPTLRRKLEESASFESLSPSSRPDSPTRSQ    2479
Odobenus.rosmarus.divergens       -SSTKSSGSESDRSERPVLVRQSTFIKEAPSPTLRRKLEESASFESLSPSSRPDSPTRSQ    2479
Taeniopygia.guttata               -PSTKSSGSESDRSERPVLVRQSTFIKEAPSPTLRRKLEESASFESLSP-SRPDSPTRSQ    2472
Anolis.carolinensis               -SSAKSSGSESDRSERPALVRQSTFIKETPSPTLRRKLEESASFESLSP-SRPDSPTQSQ    2476
Gallus.gallus                     -SSAKSSGSESDRSERPVLVRQSTFIKEAPSPTLRRKLEESASFESLSP-SRPDSPTRSQ    2484
                                     :.  . : :  .:* *::*.** ::  *           ..      .        :

Ciona.intestinalis                --------------KQSEPQQKSKTLWKGLKKLLSPSDEAKNIVPSPTRKTPKKPQVTTR    2318
Strongylocentrotus.purpuratus     VLSPVQSEEAPSVTESESSVSLSKGGWKKTGSGIQGSQESKKSSTGN-KPVSK---TTGV    2456
Danio.rerio                       SQTPVSSPSLPDMSL-SLP--YQGSGWTKAPQSQN-SAENGDGKSLKRHDISR-SHSESP    2474
Xenopus.tropicalis                TQTPALSPSLPDMSL-STHS-IQAGGWRKMPPNLNPAAEH--GDSRRRHDISR-SHSESP    2534
Xenopus.laevis                    TQTPALSPSLPDMAL-STHS-IQAGGWRKMPPNLNPAAEH--GDSRRRHDISR-SHSESP    2530
Ornithorhynchus.anatinus          AQTPVLSPSLPDTPL-STHPSVQAGGWRRLPPSLNPGVDYSEGRPAKRHDIAR-SHSESP    2537
Monodelphis.domestica             VQTPILSPSLPDMSL-STHSSIQTGSWRKLPPNLNPSIEFNDGRSTKRHDIAR-SHSESP    2534
Mus.musculus                      AQTPVLSPSLPDMSL-STHPSVQAGGWRKLPPNLSPTIEYNDGRPTKRHDIAR-SHSESP    2536
Rattus.norvegicus                 AQTPVLSPSLPDMSL-STHPSVQAGGWRKLPPNLSPTIEYSDGRPSKRHDIAR-SHSESP    2536
Sorex.araneus                     AQTPVLSPSLPDMPL-SAHSSVQAGGWRKLPPNLSPTIEYSDGRPAKRHDIAR-SHSESP    2530
Octodon.degus                     AQTPVLSPSLPDMCL-STHTSVQAGGWRKLPPNLSPTIEYNDGRPAKRHDIAR-SHSESP    2534
Heterocephalus                    AQTPVLSPSLPDMSL-PTHSSLQAGGWRKLPPNLSPTIEYNDGRPTKRHDIAR-SHSESP    2532
Jaculus.jaculus                   AQTPVLSPSLPDMSL-STHPSVQPGGWRKLPPNLSPTIEYNDGRAAKRHDIAR-SHSESP    2535
Loxodonta.africana                AQTPVLSPSLPDMSL-STHSSVQPGGWRKLPPNLSLTVEYNDGRPAKRHDIAR-SHSESP    2529
Trichechus.manatus.latirostris    AQTPVLSPSLPDMSL-STHSSVQPGGWRKLPPNLSLTVEYNDGRPAKRHDIAR-SHSESP    2531
```

```
Otolemur.garnettii                  AQTPVLSPSLPDMSL-STHSSVQAGGWRKLPPNLSPTIEYNDGRPAKRHDIAR-SHSESP    2537
Callithrix.jacchus                  AQTPVLSPSLPDMSL-STHSSVQPGGWRKLPPNLSPTIEYNDGRPAKRHDIAR-SHSESP    2536
Macaca.mulatta                      AQTPVLSPSLPDMSL-STHSSVQAGGWRKLPPNLSPTIEYNDGRPAKRHDIAR-SHSESP    2537
Nomascus.leucogenys                 AQTPVLSPSLPDMSL-STHSSVQAGGWRKLPPNLSPTIEYNDGRPAKRHDIAR-SHSESP    2537
Pongo.abelii                        AQTPVLSPSLPDMSL-STHSSVQAGGWRKLPPNLSPTIEYNDGRPAKRHDIAR-SHSESP    2537
Homo.sapiens                        AQTPVLSPSLPDMSL-STHSSVQAGGWRKLPPNLSPTIEYNDGRPAKRHDIAR-SHSESP    2536
Gorilla.gorilla                     AQTPILSPSLPDMSL-STHSSVQAGGWRKLPPNLSPTIEYSDGRPAKRHDIAR-SHSESP    2537
Pan.troglodytes                     AQTPVLSPSLPDMSL-STHSSVQAGGWRKLPPNLSPTIEYNDGRPAKRHDIAR-SHSESP    2537
Pan.paniscus                        AQTPVLSPSLPDMSL-STHSSVQAGGWRKLPPNLSPTIEYNDGRPAKRHDIAR-SHSESP    2537
Canis.lupus.familiaris              AQTPVLSPSLPDMSL-SAHPSIQSGGWRKLPPNLSPTIEYNDGRPAKRHDIAR-SHSESP    2537
Bos.taurus                          AHTPVLSPSLPDMSL-STHSSLQSGGWRKLPPNLSPTIEYNDGRPVKRHDIAR-SHSESP    2543
Ovis.aries                          AHTPVLSPSLPDMSL-PTHSSVQSGGWRKLPPNLSPTIEYNDGRPVKRHDIAR-SHSESP    2544
Orcinus.orca                        AQTPILSPSLPDMSL-STHSSVQAGGWRKLPPNLSPTIEYNDGRPVKRHDIAR-SHSESP    2540
Sus.scrofa                          AQTPVLSPSLPDMSL-STHSSVQAGGWRKLPPNLSPTIEYNDGRPIKRHDIAR-SHSESP    2540
Ceratotherium.simum.simum           AQTPILSPSLPDMSL-STHSSVQAGGWRKLPPNLSPTVEYNDGRPAKRHDIAR-SHSESP    2538
Equus.caballus                      AQTPILSPSLPDMSL-SMHSSVQAGGWRKLPPNLSPTVEYNDGRPAKRHDIAR-SHSESP    2538
Felis.catus                         AQTPVLSPSLPDMSL-STHSSVQSGGWRKLPPNLSPTIEYNDGRPAKRHDIAR-SHSESP    2538
Ailuropoda.melanoleuca              AQTPVLSPSLPDMSL-STHSSVQSGGWRKLPPNLSPTIEYNDGRPAKRHDIAR-SHSESP    2537
Mustela.putorius.furo               AQTPVLSPSLPDMSL-STHSSVQSGGWRKLPPNLSPTIEYNDGRPAKRHDIAR-SHSESP    2537
Odobenus.rosmarus.divergens         AQTPVLSPSLPDMSL-STHSSVQSGGWRKLPPNLSPTIEYNDGRPAKRHDIAR-SHSESP    2537
Taeniopygia.guttata                 VQTPVLSPSLPDMSL-STHSPAQSSGWRKLAPTQSPTIEY-DGRPAKRHDIAR-SHSESP    2529
Anolis.carolinensis                 TQTPILSPSLPDMSL-STHSAIQTSGWRKLPPSLSPSLEYSDGRPTKRHDITR-SHSESP    2534
Gallus.gallus                       LQTPVLSPSLPDMSL-STHSTAQTSGWRKLPPNLSPSVEY-DGRPAKRHDIAR-SHSESP    2541
                                                          .   *       .   :         :   :

Ciona.intestinalis                  GKVVQNQS----DRVIESEDSSTGFNSGTWTKSKTGSTSSSLQSKQVRRTPERPHRT---    2371
Strongylocentrotus.purpuratus       SKITPRRTGSPGLRTPAARSSSPGQRVNTPARRSES--PSRMSSTSQ--RSESPSRASTT    2512
Danio.rerio                         SRLPINRTGTWK--REHSKHSSSLPRVGTWKRTGSS--SSILSASSE--SSEK---GRS-    2524
Xenopus.tropicalis                  SRLPITRSGTWK--REHSKHSSSLPRVSTWRRTGSS--SSILSASSE--SSEK---AKS-    2584
Xenopus.laevis                      SRLPITRSGTWK--REHSKHSSSLPRVSTWRRTGSS--SSILSASSE--SSEK---AKS-    2580
Ornithorhynchus.anatinus            SRLPINRSGTWK--REHSKHSSSLPRVSTWRRTGSS--SSILSASSE--SSEK---AKS-    2587
Monodelphis.domestica               SRLPVNRSGTWK--REHSKHSSSLPRVSTWRRTGSS--SSILSASSE--SSEK---AKS-    2584
Mus.musculus                        SRLPINRAGTWK--REHSKHSSSLPRVSTWRRTGSS--SSILSASSE--SSEK---AKS-    2586
Rattus.norvegicus                   SRLPVNRAGTWK--REHSKHSSSLPRVSTWRRTGSS--SSILSASSE--SSEK---AKS-    2586
Sorex.araneus                       SRLPINRSGTWK--REHSKHSSSLPRVSTWRRTGSS--SSILSASSE--SSEK---AKS-    2580
Octodon.degus                       SRLPINRSGTWK--REHSKHSSSLPRVSTWRRTGSS--SSILSASSE--SSEK---AKS-    2584
Heterocephalus                      SRLPINRSGTWK--REHSKHSSSLPRVSTWRRTGSS--SSILSASSE--SSEK---AKS-    2582
Jaculus.jaculus                     SRLPINRSGTWK--REHSKHSSSLPRVSTWRRTGSS--SSILSASSE--SSEK---AKS-    2585
Loxodonta.africana                  SRLPINRSGTWK--REHSKHSSSLPRVSTWRRTGSS--SSILSASSE--SSEK---AKS-    2579
Trichechus.manatus.latirostris      SRLPINRSGTWK--REHSKHSSSLPRVSTWRRTGSS--SSILSASSE--SSEK---AKS-    2581
Otolemur.garnettii                  SRLPINRAGTWK--REHSKHSSSLPRVSTWRRTGSS--SSILSASSE--SSEK---AKS-    2587
Callithrix.jacchus                  SRLPINRSGTWK--REHSKHSSSLPRVSTWRRTGSS--SSILSASSE--SSEK---AKS-    2586
Macaca.mulatta                      SRLPINRSGTWK--REHSKHSSSLPRVSTWRRTGSS--SSILSASSE--SSEK---AKS-    2587
Nomascus.leucogenys                 SRLPINRSGTWK--REHSKHSSSLPRVSTWRRTGSS--SSILSASSE--SSEK---AKS-    2587
Pongo.abelii                        SRLPINRSGTWK--REHSKHSSSLPRVSTWRRTGSS--SSILSASSE--SSEK---AKS-    2587
Homo.sapiens                        SRLPINRSGTWK--REHSKHSSSLPRVSTWRRTGSS--SSILSASSE--SSEK---AKS-    2586
Gorilla.gorilla                     SRLPINRSGTWK--REHSKHSSSLPRVSTWRRTGSS--SSILSASSE--SSEK---AKS-    2587
Pan.troglodytes                     SRLPINRSGTWK--REHSKHSSSLPRVSTWRRTGSS--SSILSASSE--SSEK---AKS-    2587
Pan.paniscus                        SRLPINRSGTWK--REHSKHSSSLPRVSTWRRTGSS--SSILSASSE--SSEK---AKS-    2587
Canis.lupus.familiaris              SRLPINRSGTWK--REHSKHSSSLPRVSTWRRTGSS--SSILSASSE--SSEK---AKS-    2587
Bos.taurus                          SRLPINRSGTWK--REHSKHSSSLPRVSTWRRTGSS--SSILSASSE--SSEK---AKS-    2593
Ovis.aries                          SRLPINRSGTWK--REHSKHSSSLPRVSTWRRTGSS--SSILSASSE--SSEK---AKS-    2594
Orcinus.orca                        SRLPINRSGTWK--REHSKHSSSLPRVSTWRRTGSS--SSILSASSE--SSEK---AKS-    2590
Sus.scrofa                          SRLPINRSGTWK--REHSKHSSSLPRVSTWRRTGSS--SSILSASSE--SSEK---AKS-    2590
Ceratotherium.simum.simum           SRLPINRSGTWK--REHSKHSSSLPRVSTWRRTGSS--SSILSASSE--SSEK---AKS-    2588
Equus.caballus                      SRLPINRSGTWK--REHSKHSSSLPRVSTWRRTGSS--SSILSASSE--SSEK---AKS-    2588
Felis.catus                         SRLPINRSGTWK--REHSKHSSSLPRVSTWRRTGSS--SSILSASSE--SSEK---AKS-    2588
Ailuropoda.melanoleuca              SRLPINRSGTWK--REHSKHSSSLPRVSTWRRTGSS--SSILSASSE--SSEK---AKS-    2587
Mustela.putorius.furo               SRLPINRSGTWK--REHSKHSSSLPRVSTWRRTGSS--SSILSASSE--SSEK---AKS-    2587
Odobenus.rosmarus.divergens         SRLPINRSGTWK--REHSKHSSSLPRVSTWRRTGSS--SSILSASSE--SSEK---AKS-    2587
Taeniopygia.guttata                 SRLLINRSGTWK--REHSKHSSSLPRVSTWRRTGSS--SSILSASSE--SSEK---AKS-    2579
Anolis.carolinensis                 SRLPINRSGTWK--REHSKHSSSLPRVSTWRRTGSS--SSILSASSE--SSEK---AKS-    2584
Gallus.gallus                       SRLPINRSGTWK--REHSKHSSSLPRVSTWRRTGSS--SSILSASSE--SSEK---AKS-    2591
                                    .::   ::         :. **   . .*  :   .   * :.:..     *
```

```
Ciona.intestinalis                  -----------PSNAGSQSSSLSL------SSSSASNGSSSSV-----DVYQQPS-----    2404
Strongylocentrotus.purpuratus       SQSSVSKQRTTPQTKGPASTQRNNSATSKINTGLQKSGGMSKRSASPSGVKTNPNVNGTH    2572
Danio.rerio                         ---EDERQPT-NPP--QKSGKEGG---------LERKGTWRKAKG--SETSYAPM-----    2562
Xenopus.tropicalis                  ---EDEKQQV-CSFLGPKS--ECS---------SSAKGTWRKIKE--SEILETPS-----    2622
Xenopus.laevis                      ---EDEKQQV-CSFPGPRS--ECS---------SSAKGTWRKIKE--SEILETPS-----    2618
Ornithorhynchus.anatinus            ---EDEKQV--SAPAGPKLAKENQ---------APAKGTWRKIKE--SEMAPAGN-----    2626
Monodelphis.domestica               ---EDEKHV--SSLLGTKQTKENQ---------GPAKGTWRKIKE--SEMTPISN-----    2623
Mus.musculus                        ---EDERHV--SSMPAPRQMKENQ---------VPTKGTWRKIKE--SDISPTGM-----    2625
Rattus.norvegicus                   ---EDEKHV--NSVPGPRQMKENQ---------VPTKGTWRKIKE--SEISPTNT-----    2625
Sorex.araneus                       ---EDEKQM--NFSSSTKPAKESQ---------VSTKGTWRKIKE--SEISPTNS-----    2619
Octodon.degus                       ---EDEKHV--YSISGVKQAKENQ---------VSTKGTWRKIKE--SEISPTNL-----    2623
Heterocephalus                      ---EDEKHV--YSISGTKQTKENQ---------VSTRGTWRKIKE--SELSPTNM-----    2621
Jaculus.jaculus                     ---EDEKHV--NSMPGPKQTKESQ---------LSTKGTWRKIKE--SEISPTNT-----    2624
Loxodonta.africana                  ---EDEKHV--NSISATKQTKENH---------VPTKGTWRKIKE--SEISPTNS-----    2618
Trichechus.manatus.latirostris      ---EDEKLV--NSISATKQTKENQ---------VPTKGTWRKIKE--SEISPTNS-----    2620
Otolemur.garnettii                  ---EDEKHV--NSISGTKQSKENQ---------VSTKGTWRKIKE--SEISPTNS-----    2626
Callithrix.jacchus                  ---EDEKHV--NSISGTKQSKENQ---------VSAKGTWRKIKE--NEISPTNS-----    2625
Macaca.mulatta                      ---EDEKHV--NSISGTKQSKENQ---------VSAKGTWRKIKE--NEISPTNS-----    2626
Nomascus.leucogenys                 ---EDEKHV--NSISGTKQSKENQ---------VSAKGTWRKIKE--NEISPTNS-----    2626
Pongo.abelii                        ---EDEKHV--NSISGTKQSKENQ---------VSAKGTWRKIKE--NEISPTNS-----    2626
Homo.sapiens                        ---EDEKHV--NSISGTKQSKENQ---------VSAKGTWRKIKE--NEFSPTNS-----    2625
Gorilla.gorilla                     ---EDEKHV--NSISGTKQSKENQ---------VSAKGTWRKIKE--NEISPTNS-----    2626
Pan.troglodytes                     ---EDEKHV--NSISGTKQSKANQ---------VSAKGTWRKIKE--NEISPTNS-----    2626
Pan.paniscus                        ---EDEKHV--NSISGTKQSKENQ---------VSAKGTWRKIKE--NEISPTNS-----    2626
Canis.lupus.familiaris              ---EDEKHV--NSISGTKQTKENQ---------VSTKGTWRKIKE--NEISPTNS-----    2626
Bos.taurus                          ---EDEKQV--NSISGSKQTKENQ---------VSTKGTWRKIKE--SEISPTNS-----    2632
Ovis.aries                          ---EDEKQV--NSISGSKQTKENQ---------VSTKGTWRKIKE--SEISPTNS-----    2633
Orcinus.orca                        ---EDEKHV--NSTSGTKQTKENQ---------VSTKGTWRKMKE--SEISPTNS-----    2629
Sus.scrofa                          ---EDEKHV--NSISGTKQTKESQ---------VSTKGTWRKIKE--SEISPTNT-----    2629
Ceratotherium.simum.simum           ---EDEKHV--NSISGTKQTKENQ---------VSTKGTWRKIKE--SEISPTNS-----    2627
Equus.caballus                      ---EDERHV--NSISGTKQTKENQ---------VSTKGTWRKIKE--SEISPTNS-----    2627
Felis.catus                         ---EDEKHV--NSISGTKQTKENQ---------VSTKGTWRKIKE--NEISPTNS-----    2627
Ailuropoda.melanoleuca              ---EDEKHV--NSISGTKQTKENQ---------VSTKGTWRKIKE--SEISPTNS-----    2626
Mustela.putorius.furo               ---EDEKHV--NSISGTKQTKENQ---------VSTKGTWRKIKE--SEISPTNS-----    2626
Odobenus.rosmarus.divergens         ---EDEKHV--NFISGTKQTKENQ---------VSTKGTWRKIKE--SEISPTNS-----    2626
Taeniopygia.guttata                 ---EDEKQHG-GSLPGHKQSKESQ---------APAKGTWRKIKE--NEIPQIMN-----    2619
Anolis.carolinensis                 ---EDEKQYV-SSFSGLKQAKETQ---------APTKGTWRKIKE--NEIPQIMT-----    2624
Gallus.gallus                       ---EDEKQHG-SSLSGQKQSKESQ---------APAKGTWRKIKE--NEIPQIMN-----    2631
                                                                         *   .

Ciona.intestinalis                  -----TRMNIYPTARNMSPG-AHVPKSPSAKSRIIVNSKSPADKTNQGWRRTGDSRIHSR    2458
Strongylocentrotus.purpuratus       ASTPPKSTASRPNTRPTKSGNAGVRTGPPT--TRVASASPKPSGASARA--VRNKPASRP    2628
Danio.rerio                         -----------------------------SL-DLQDQTGDAMSKSEDVWVRIEDCPIN--    2590
Xenopus.tropicalis                  --NGSS---------NTI---AESSCSLESK-TLVYQMAPAVSKTEDVWVRIEDCPIN--    2665
Xenopus.laevis                      --NGSS---------STI---AESNCSLESK-TLVYQMAPAVSKTEDVWVRIEDCPIN--    2661
Ornithorhynchus.anatinus            --TPQ----------AVS---SGAPNGADSK-TLVYQMAPAVSKTEDVWVRIEDCPIN--    2668
Monodelphis.domestica               --VSQ----------VTS---SGTTNGADSK-TLIYQMAPAVSKTEDVWVRIEDCPIN--    2665
Mus.musculus                        --ASQ----------SAS---SGAASGAESK-PLIYQMAPPVSKTEDVWVRIEDCPIN--    2667
Rattus.norvegicus                   --VSQ----------TTS---SGAASGAESK-TLIYQMAPAVSRTEDVWVRIEDCPIN--    2667
Sorex.araneus                       --TSQ----------TTS---SGAANGAESK-TLIYQMAPAVSKTEDVWVRIEDCPIN--    2661
Octodon.degus                       --TSP----------TTS---SGATNGAESK-TLIYQMAPAVSKTEDVWVRIEDCPIN--    2665
Heterocephalus                      --TSP----------TTS---SGAINGAESK-TLIYQMAPAVSKTEDIWVRIEDCPIN--    2663
Jaculus.jaculus                     ---PQ----------TTS---SGATNGAESK-TLIYQMAPAVSKTEDVWVRIEDCPIN--    2665
Loxodonta.africana                  --TSP----------TTS---SGATNGAESK-TLIYQMAPAVSKTEDVWVRIEDCPIN--    2660
Trichechus.manatus.latirostris      --TSQ----------TTS---SGATNGAESK-TLIYQMAPAVSKTEDVWVRIEDCPIN--    2662
Otolemur.garnettii                  --TSQ----------TNS---SGATTGAESK-TLIYQMAPAVSKTEDVWVRIEDCPIN--    2668
Callithrix.jacchus                  --TSQ----------TIS---SGATNGAESK-TLIYQMAPAVSKTEDIWVRIEDCPIN--    2667
Macaca.mulatta                      --TSQ----------TVS---SGATNGAESK-TLIYQMAPAVSKTEDVWVRIEDCPIN--    2668
Nomascus.leucogenys                 --TSQ----------TVS---SGATNGAESK-TLIYQMAPAVSKTEDVWVRIEDCPIN--    2668
Pongo.abelii                        --TSQ----------TVS---SGATNGAESK-TLIYQMAPAVSKTEDVWVRIEDCPIN--    2668
Homo.sapiens                        --TSQ----------TVS---SGATNGAESK-TLIYQMAPAVSKTEDVWVRIEDCPIN--    2667
Gorilla.gorilla                     --TSQ----------TVS---SGATNGAESK-TLIYQMAPAVSKTEDVWVRIEDCPIN--    2668
Pan.troglodytes                     --TSQ----------TVS---SGATNGAESK-TLIYQMAPAVSKTEDVWVRIEDCPIN--    2668
Pan.paniscus                        --TSQ----------TVS---SGATNGAESK-TLIYQMAPAVSKTEDVWVRIEDCPIN--    2668
Canis.lupus.familiaris              --TSQ----------TTS---SGAANGAESK-TLIYQMAPAVSKTEDVWVRIEDCPIN--    2668
```

```
Bos.taurus                          --TSQ----------TTS---SGAANGAESK-TLIYQMAPAVSKTEDVWVRIEDCPIN--    2674
Ovis.aries                          --TSQ----------TTS---SGAANGAESK-TLIYQMAPAVSKTEDVWVRIEDCPIN--    2675
Orcinus.orca                        --TSQ----------TTS---SGAANGAESK-TLIYQMAPAVSKTEDVWVRIEDCPIN--    2671
Sus.scrofa                          --TSQ----------TTS---SGAANGAESK-TLIYQMAPAVSKTEDVWVRIEDCPIN--    2671
Ceratotherium.simum.simum           --TSQ----------TTS---SGAANGAESK-TLIYQMAPAVSKTEDVWVRIEDCPIN--    2669
Equus.caballus                      --TSQ----------TTS---SGAVNGAESK-TLIYQMAPAVSKTEDVWVRIEDCPIN--    2669
Felis.catus                         --TSQ----------TTS---SGAANGAESK-TLIYQMAPAVSKTEDVWVRIEDCPIN--    2669
Ailuropoda.melanoleuca              --TSQ----------TTS---LGAANGAESK-TLIYQMAPAVSKTEDVWVRIEDCPIN--    2668
Mustela.putorius.furo               --TSQ----------TTS---SGAANGAESK-TLIYQMAPAVSKTEDVWVRIEDCPIN--    2668
Odobenus.rosmarus.divergens         --TSQ----------TTF---SGAANGAESK-TLIYQMAPAVSKTEDVWVRIEDCPIN--    2668
Taeniopygia.guttata                 --DPQH---------S-S---SGATNGSDSK-TLIYQMAPAVSKTEDVWVRIEDCPIN--    2661
Anolis.carolinensis                 --SPPQ---------NSS---SGAANGADSK-TLIYQMAPAVSKTEDVWVRIEDCPIN--    2667
Gallus.gallus                       --DPQH---------P-S---SSATSSSDSK-TLIYQMAPAVSKTEDVWVRIEDCPIN--    2673
                                                                 :      .     . :.       :

Ciona.intestinalis                  KISM-------ESSSSATSAAGNR----------------ASGRKHGSQASIATSSDKRS    2495
Strongylocentrotus.purpuratus       SSAEGSRSLSGNNSSSKETPSPQTPSKFGTFTKKKAQ-----------------------    2665
Danio.rerio                         ------------NPRSSKSPTASTPPVIDSLPIKGLACDRDSSESHSKLMSE-------N    2631
Xenopus.tropicalis                  ------------NPRSGRSPTGNSPPVIDNVLDQGQKEEA-VKDCHTRHNSGNG-----N    2707
Xenopus.laevis                      ------------NPRSGRSPTGNSPPVIDNVLDQGQKEEA-AKDCHTRHNSGNG-----N    2703
Ornithorhynchus.anatinus            ------------NPRSGRSPTGNTPPVIDSVSEKGSSTGKEAKESQGKQNAGNG----N-    2711
Monodelphis.domestica               ------------NPRSGRSPTGNTPPVIDNVAEKVSSGNKESKDNQGKPNGGNG----SS    2709
Mus.musculus                        ------------NPRSGRSPTGNTPPVIDSVSEKGSSSIKDSKDTHGKQSVGSG----S-    2710
Rattus.norvegicus                   ------------NPRSGRSPTGNTPPVIDSISEKGNPSIKDSKDTQGKQSVGSG----S-    2710
Sorex.araneus                       ------------NPRSGRSPTGNTPPVIDSFSEKGNPNTKDSKENQGKQSMGNG----S-    2704
Octodon.degus                       ------------NPRSGRSPTGNTPPVIDRVSEKGNPSIKDSKDNQGKPNMSNG----S-    2708
Heterocephalus                      ------------NPRSGRSPTGNTPPVIDGVSEKGNPSIKDSKDNQGKQNVGNG----S-    2706
Jaculus.jaculus                     ------------NPRSGRSPTGNMPPVIDGVSEKGNPNVKDSKDNQGKQNVGNG----S-    2708
Loxodonta.africana                  ------------NPRSGRSPTGNTPPVIDTVSEKGDPNVKDSKDNQGKQDVGNG----S-    2703
Trichechus.manatus.latirostris      ------------NPRSGRSPTGNTPPVIDSVTEKGNSDVKDSKDYQGHQNVGNG----S-    2705
Otolemur.garnettii                  ------------NPRSGRSPTGNTPPVIDSVSEKGNPNVKDSKDNQGKQNVGNG----T-    2711
Callithrix.jacchus                  ------------NPRSGRSPTGNTPPVIDSVSEKGNPNIKDSKDNQAKQNVGNG----S-    2710
Macaca.mulatta                      ------------NPRSGRSPTGNTPPVIDSVSEKGNPN-KDSKDNQAKQNVGNG----S-    2710
Nomascus.leucogenys                 ------------NPRSGRSPTGNTPPVIDSVSEKGNPNIKDSKDNQAKQNVGNG----S-    2711
Pongo.abelii                        ------------NPRSGRSPTGNTPPVIDSVSEKGNPNIKDSKDNQAKQNVGNG----S-    2711
Homo.sapiens                        ------------NPRSGRSPTGNTPPVIDSVSEKANPNIKDSKDNQAKQNVGNG----S-    2710
Gorilla.gorilla                     ------------NPRSGRSPTGNTPPVIDSVSEKGNPNIKDSKDNQAKQNVGNG----S-    2711
Pan.troglodytes                     ------------NPRSGRSPTGNTPPVIDSVSEKGNPNIKDSKDNQAKQNVGNG----S-    2711
Pan.paniscus                        ------------NPRSGRSPTGNTPPVIDSVSEKGNPNIKDSKDNQAKQNMGNG----S-    2711
Canis.lupus.familiaris              ------------NPRSGRSPTGNTPPVIDTVLEKGNPNAKDAKDNQGKPSVGNG----S-    2711
Bos.taurus                          ------------NPRSGRSPTGNTPPVIDTVSEKGNPNPKDSKDNQGKQNVSNG----S-    2717
Ovis.aries                          ------------NPRSGRSPTGNSPPVIDTVSEKGSPNPKDSKDNQGKQNVSNG----S-    2718
Orcinus.orca                        ------------NPRSGRSPTGNTPPVIDTVSEKGNPNTKDSKDHQGKQNVSNG----S-    2714
Sus.scrofa                          ------------NPRSGRSPTGNTPPVIDTISEKGNPNAKDSKDNQGKHNVSNG----G-    2714
Ceratotherium.simum.simum           ------------NPRSGRSPTGNTPPVIDSLSEKGNSNAKDSKDNQGKQNVGNG----S-    2712
Equus.caballus                      ------------NPRSGRSPTGNTPPVIDSVSEKGNSHAKDSKDNQGKQNVGNA----S-    2712
Felis.catus                         ------------NPRSGRSPTGNTPPVIDTVSEKGNPNAKDSKDNQGKQNVGNG----S-    2712
Ailuropoda.melanoleuca              ------------NPRSGRSPTGNTPPVIDTVSEKGNPNVKDAKDNQGKQNVGNG----S-    2711
Mustela.putorius.furo               ------------NPRSGRSPTGNTPPVIDTVSEKGNPNAKDAKDNQGKQNVGNG----S-    2711
Odobenus.rosmarus.divergens         ------------NPRSGRSPTGNTPPVIDTVSEKGNPNAKDAKDNQGKQNVGNG----S-    2711
Taeniopygia.guttata                 ------------NPRSGRSPTGNTPPVIDSISEKGGVNGKDPKEIQEKQT-PGN----GG    2704
Anolis.carolinensis                 ------------NPRSGKSPTGKTPPVIDSVLEKGNVNSKDSKDLHGKQNAAGN----GN    2711
Gallus.gallus                       ------------NPRSGRSPTGNTPPVIDSVSEKGVVNGKDSKEIQEKQN-PGN----GS    2716
                                                .  *  : : .

Ciona.intestinalis                  VSV-ASGQSPSARLFYEVVENP----SRRNPGEGA------------------------L    2526
Strongylocentrotus.purpuratus       ---------------SS-VETYDKKETERNSGESDSV------------SKSDLSSD---    2694
Danio.rerio                         AAMSHLGSETNLNLLRS-SESLDKKVTDIKPAPSN-PNIGPELHEFPVSERTPFSSTNSS    2689
Xenopus.tropicalis                  V----PLLENRQKSFIK-LDGSDTKGTDPKTLINN----QQETNENTVAERTPFSSSSSS    2758
Xenopus.laevis                      V----PLLENRQKSFIK-VDGLDTKGTDPKSLINN----QQETNENTVAERTAFSSSSSS    2754
Ornithorhynchus.anatinus            APVRSAGLENRLNSFIQ-LDSPDKKGTEGKPAQSN-PTPAPEPGESSVPERTPFSSSSSS    2769
Monodelphis.domestica               APARTVGLENRLNSFIQ-IDSPDKKAAETKSGQVN-LVPAPETSETSVAERTPFSSTSSS    2767
Mus.musculus                        -PVQTVGLETRLNSFVQ-VEAPEQKGTEAKPGQSN-PVSIAETAETCIAERTPFSSSSSS    2767
Rattus.norvegicus                   -PVQTVGLENRLNSFIQ-VEAPEQKGTETKAGQGS-PAPVAETGETCMAERTPFSSSSSS    2767
Sorex.araneus                       VPVRTVGLENRLNSFLQ-VDAPDQKGTEVKPGQSN-SVLASETSESAIAERTPFSSSSSS    2762
```

```
Octodon.degus                        APVLSAGLENRLNSFIQ-VDAPDQKGTETKSGQS-NPVPTAETGENSIAERTPFSSSSSS    2766
Heterocephalus                       APMHTTGLENRLNSFIQ-VDAPDQKGTETKSGQS-NPVPTAETSENSIAERTPFSSSSSS    2764
Jaculus.jaculus                      VPVHTIGLENRLNSFIQ-VDAPDQKGTETKSGQS-NSVPAAETSETSIAERTPFSSSSSS    2766
Loxodonta.africana                   APVRTMGLENRRNSFIQ-VDSPDQKGTEAKAGQS-NAVPAPENSESSIAERTPFSSSSSS    2761
Trichechus.manatus.latirostris       AAVRTVGLENRRNSFIQ-VDSPDQKGTEAKLGQS-NAVPAPENSESSIAERTPFSSSSSS    2763
Otolemur.garnettii                   ASVRTMGLENRLNSFIQ-VDAPDQKGTETKAGQS-NPVPASETNESSVAERTPFSSSSSS    2769
Callithrix.jacchus                   VPMRTVGLENRLNSFIQ-VDAPDQKGTETKPGQN-NPVPASETNESSIVERTPFSSSSSS    2768
Macaca.mulatta                       VPMRTVGLENRLNSFIQ-VDAPDQKGTETKPGQN-NPVPVSETNESSIVERTPFSSSSSS    2768
Nomascus.leucogenys                  VPMRTVGLENRLNSFIQ-VDAPDQKGTETKPGQN-NPVPVSEANESSIVERTPFSSSSSS    2769
Pongo.abelii                         VPMRTVGLENRLNSFIQ-VDAPDQKGTETKPGQN-NPVPVSETNESSIVERTPFSSSSSS    2769
Homo.sapiens                         VPMRTVGLENRLNSFIQ-VDAPDQKGTEIKPGQN-NPVPVSETNESSIVERTPFSSSSSS    2768
Gorilla.gorilla                      VPMRTVGLENRLNSFIQ-VDAPDQKGTEIKPGQN-NPVPVSETNESSIVERTPFSSSSSS    2769
Pan.troglodytes                      VPMRTVGLENRLNSFIQ-VDAPDQKGTEIKPGQN-NPVPVSETNESSIVERTPFSSSSSS    2769
Pan.paniscus                         VPMRTVGLENRLNSFIQ-VDAPDQKGTEIKPGQN-NPVPVSETNESSIVERTPFSSSSSS    2769
Canis.lupus.familiaris               GPVRAVGLENRLNSFIQ-VEAPDQKGTEAKPGPS-NPVPAPEANESCAAERTPFSSSSSS    2769
Bos.taurus                           APTRTMGLENRLNSFIQ-VDPPDQKGTETKPGHSNNPVPASETSESSIAERTPFSSSSSS    2776
Ovis.aries                           APTRTMGLENRLNSFIQ-VDPPDQKGTETKPGQSNNPVPTSETNESSIAERTPFSSSSSS    2777
Orcinus.orca                         APVRTMGLENRLNSFIQ-VDAPDQKGTEGKPGQS-HPVPASETNESSIAERTPFSSSSSS    2772
Sus.scrofa                           APTRTMGLENRLNSFIQ-VDAPDQKGTETKPGQS-NSVPASETNESSIAERTPFSSSSSS    2772
Ceratotherium.simum.simum            APVRIMGLENRLNSFIQ-VDAPDQKGTEGKPGQS-NPVPASETSESSVAERTPFSSSSSS    2770
Equus.caballus                       APGRIMGLENRLNSFIQ-VDAPDQKGTEAKPGQS-NPVPASETNESSIAERTPFSSSSSS    2770
Felis.catus                          APIRTMGLENRLNSFIQ-VDAPDQKGTEAKPGQS-NPVPVSETNESSIAERTPFSSSSSS    2770
Ailuropoda.melanoleuca               APVRTMGLENRLNSFIQ-VDGPDQKGTEAKPGQS-NPVPASEANGSSVAERTPFSSSGSS    2769
Mustela.putorius.furo                APVRTMGLENRLNSFIQ-VDAPDQKGTEAKPGQS-NPVPASETNESSVAERTPFSSSSSS    2769
Odobenus.rosmarus.divergens          APGRTMGLENRLNSFIQ-VDAPDQKGTEAKPGQS-NPVPASETNESSVAERTPFSSSSSS    2769
Taeniopygia.guttata                  VPVRTIGLENRLNSFFQ-IDSPDKKGTETKPLQNN-PVPAPEINESTVNERTPFSSSSSS    2762
Anolis.carolinensis                  VPVRTMGLE-RLNSFIQ-VDSPDKQGTETKPVLTN-PVPPPETTESTVTERTPFSSSNSS    2768
Gallus.gallus                        VPVRTIGLENRLNSFFQ-MDSPDKKGNETKPLQTN-PVPAPENNESTVSERTPFSSSSSS    2774
                                                     .  :         :

Ciona.intestinalis                   PFPSPVRRVVQEVAPFNYRPYRIGESEEEEPNHKLDLTRKQTTV----------------    2570
Strongylocentrotus.purpuratus        ---------------------DQKSSQLKAKDLCKELNIPVKNCGGKIVSPTRETAEGDG    2733
Danio.rerio                          KHSSPSGAVAARVSPFNYTPSPRKSSADGSTPRPSQIPTPISSNAKK-------------    2736
Xenopus.tropicalis                   KHSSPSGTVAARVTPFNYNPSPRKSTAESSTSRPSQIPTPVTNSTKK-------------    2805
Xenopus.laevis                       KHSSPSGTVAARVTPFNYNPSPRKSNGENSTSRPSQIPTPVTNSTKK-------------    2801
Ornithorhynchus.anatinus             KHSSPSGAVAARVTPFNYNPSPRKSSADSSSARPSQIPTPVNNSTKK-------------    2816
Monodelphis.domestica                KHSSPSGTVAARVTPFNYNPSPRKSSADSSAARPSQIPTPVNNSTKK-------------    2814
Mus.musculus                         KHSSPSGTVAARVTPFNYNPSPRKSSADSTSARPSQIPTPVSTNTKK-------------    2814
Rattus.norvegicus                    KHSSPSGTVAARVTPFNYNPSPRKSSADSTSARPSQIPTPVGSSTKK-------------    2814
Sorex.araneus                        KHSSPSGTVAARVTPFNYNPSPRKSSADSTSARPSQIPTPVNNSTKK-------------    2809
Octodon.degus                        KHSSPSGTVAARVTPFNYNPSPRKSSADSTSARPSQIPTPVNNNTKK-------------    2813
Heterocephalus                       KHSSPSGTVAARVTPFNYNPSPRKSSADSTSARPSQIPTPVNTNTKK-------------    2811
Jaculus.jaculus                      KHSSPSGTVAARVTPFNYNPSPRKSSADSTSARPSQIPTPVSNNTKK-------------    2813
Loxodonta.africana                   KHSSPSGTVAARVTPFNYNPSPRKSSADSTSARPSQIPTPVNNNTKK-------------    2808
Trichechus.manatus.latirostris       KHSSPSGTVAARVTPFNYNPSPRKSSADGTSARPSQIPTPVNNNTKK-------------    2810
Otolemur.garnettii                   KHNSPSGTVAARVTPFNYNPSPRKSSADSTSARPSQIPTPVNN-TKK-------------    2815
Callithrix.jacchus                   KHSSPSGTVAARVTPFNYNPSPRKSSADSTSARPSQIPTPVNN-TKK-------------    2814
Macaca.mulatta                       KHSSPSGTVAARVTPFNYNPSPRKSSADSTSARPSQIPTPVNNNTKK-------------    2815
Nomascus.leucogenys                  KHSSPSGTVAARVTPFNYNPSPRKSSADSTSARPSQIPTPVNNNTKK-------------    2816
Pongo.abelii                         KHSSPSGTVAARVTPFNYNPSPRKSSADSTSARPSQIPTPVNNNTKK-------------    2816
Homo.sapiens                         KHSSPSGTVAARVTPFNYNPSPRKSSADSTSARPSQIPTPVNNNTKK-------------    2815
Gorilla.gorilla                      KHSSPSGTVAARVTPFNYNPSPRKSSADSTSARPSQIPTPVNNNTKK-------------    2816
Pan.troglodytes                      KHSSPSGTVAARVTPFNYNPSPRKSSADSTSARPSQIPTPVNNNTKK-------------    2816
Pan.paniscus                         KHSSPSGTVAARVTPFNYNPSPRKSSADSTSARPSQIPTPVNNNTKK-------------    2816
Canis.lupus.familiaris               KHSSPSGTVAARVTPFNYNPSPRKSSADGTSARPSQIPTPVATATKK-------------    2816
Bos.taurus                           KHSSPSGTVAARVSPFNYNPSPRKSSTDGTSARPSQIPTPVSNNTKK-------------    2823
Ovis.aries                           KHSSPSGTVAARVSPFNYNPSPRKSSTDGTSARPSQIPTPVSNNTKK-------------    2824
Orcinus.orca                         KHSSPSGTVAARVSPFNYNPSPRKSSADSTSARPSQIPTPVNNNTKK-------------    2819
Sus.scrofa                           KHSSPSGTVAARVTPFNYNPSPRKSSADSTSARPSQIPTPVNNNTKK-------------    2819
Ceratotherium.simum.simum            KHSSPSGTVAARVTPFNYNPSPRKSSADSTSARPSQIPTPVNNNTKK-------------    2817
Equus.caballus                       KHSSPSGTVAARVTPFNYNPSPRKSSADSTSARPSQIPTPVNNNTKK-------------    2817
Felis.catus                          KHSSPSGTVAARVTPFNYNPSPRKSSADSTSARPSQIPTPVNNNTKK-------------    2817
Ailuropoda.melanoleuca               KHSSPSGTVAARVTPFNYNPSPRKSSADSTSARPSQIPTPVSNNAKK-------------    2816
Mustela.putorius.furo                KHSSPSGTVAARVTPFNYNPSPRKSSADSTSARPSQIPTPVNNNTKK-------------    2816
Odobenus.rosmarus.divergens          KHSSPSGTVAARVTPFNYNPSPRKSSADSTSARPSQIPTPVNNNTKK-------------    2816
```

```
Taeniopygia.guttata                 KHSSPIGAVAARVTPFNYNPSRRKSSVDNSSARPSQIPTPVNNSTKK-------------    2809
Anolis.carolinensis                 KHSSPSGAVAARVTPFNYNPSPRKSSVDNSSVRPSQIPTPVNTSTKK-------------    2815
Gallus.gallus                       KHNSPIGAVAARVTPFNYNPSRRKSSVDNSSARPSQIPTPVNNSTKK-------------    2821
                                                            ..         ::     .                 

Ciona.intestinalis                  ------------------------------------------------------------    2570
Strongylocentrotus.purpuratus       IWMKRPEDEVKDCASVSIHSSSRHSSSYSLSSISTNHQVGSLSRQKSAQPELPSSKKSPA    2793
Danio.rerio                         -----RDTKGDTT----------ESGSYIVTSV---------------------------    2754
Xenopus.tropicalis                  -----RDSKTETTDSSGSQSPKRHSGSYLVTSV---------------------------    2833
Xenopus.laevis                      -----RDSKTETTDSSGSQSPKRHSGSYLVTSV---------------------------    2829
Ornithorhynchus.anatinus            -----RDSKTENPEPGGTQSPKRHSGSYLVTSV---------------------------    2844
Monodelphis.domestica               -----RDSKTENTESSGTQSPKRHSGSYLVTSV---------------------------    2842
Mus.musculus                        -----RDSKTDSTESSGAQSPKRHSGSYLVTSV---------------------------    2842
Rattus.norvegicus                   -----RDSKTDSTESSGAQSPKRHSGSYLVTSV---------------------------    2842
Sorex.araneus                       -----RDSKTDSTESSGTQSPKRHSGSYLVTSV---------------------------    2837
Octodon.degus                       -----RDSKADSTESSGTQSPKRHSGSYLVTSV---------------------------    2841
Heterocephalus                      -----RDSKTDSTESNGTQSPKRHSGSYLVTSV---------------------------    2839
Jaculus.jaculus                     -----RDSKSDGTDSSGAQSPKRHSGSYLVTSV---------------------------    2841
Loxodonta.africana                  -----RDSKTDNTESSGTQSPKRHSGSYLVTSV---------------------------    2836
Trichechus.manatus.latirostris      -----RDSKTDNTESSGTQSPKRHSGSYLVTSV---------------------------    2838
Otolemur.garnettii                  -----RDSKTDSTESSGTQSPKRHSGSYLVTSV---------------------------    2843
Callithrix.jacchus                  -----RDSKTDSTDSSGTQSPKRHSGSYLVTSV---------------------------    2842
Macaca.mulatta                      -----RDSKTDSTESSGTQSPKRHSGSYLVTSV---------------------------    2843
Nomascus.leucogenys                 -----RDSKTDSTESNGTQSPKRHSGSYLVTSV---------------------------    2844
Pongo.abelii                        -----RDSKTDSTESSGTQSPKRHSGSYLVTSV---------------------------    2844
Homo.sapiens                        -----RDSKTDSTESSGTQSPKRHSGSYLVTSV---------------------------    2843
Gorilla.gorilla                     -----RDSKTDSTESSGTQSPKRHSGSYLVTSV---------------------------    2844
Pan.troglodytes                     -----RDSKTDSTESSGTQSPKRHSGSYLVTSV---------------------------    2844
Pan.paniscus                        -----RDSKTDSTESSGTQSPKRHSGSYLVTSV---------------------------    2844
Canis.lupus.familiaris              -----RDSKTEGAESGGTQSPKRHSGSYLVTSV---------------------------    2844
Bos.taurus                          -----RDSKPDSTEPSGTQSPKRHSGSYLVTSV---------------------------    2851
Ovis.aries                          -----RDSKPDNTEPSGTQSPKRHSGSYLVTSV---------------------------    2852
Orcinus.orca                        -----RDSKSDNTESSGTQSPKRHSGSYLVTSV---------------------------    2847
Sus.scrofa                          -----RDSKTDNTDSSGTQSPKRHSGSYLVTSV---------------------------    2847
Ceratotherium.simum.simum           -----RDSKTDNTESSGTQSPKRHSGSYLVTSV---------------------------    2845
Equus.caballus                      -----RDSKTDSTESSGAQSPKRHSGSYLVTSV---------------------------    2845
Felis.catus                         -----RDSKTDSTESSGTQSPKRHSGSYLVTSV---------------------------    2845
Ailuropoda.melanoleuca              -----RDSKTDSTESSGTQSPKRHSGSYLVTSV---------------------------    2844
Mustela.putorius.furo               -----RDSKTDSTESSGTQSPKRHSGSYLVTSV---------------------------    2844
Odobenus.rosmarus.divergens         -----RDSKTDSTESSGTQSPKRHSGSYLVTSV---------------------------    2844
Taeniopygia.guttata                 -----RDTKSENTDSSGTQSPKRHSGSYLVTSV---------------------------    2837
Anolis.carolinensis                 -----RDSKTENADSNGAQSPKRHSGSYLVTSV---------------------------    2843
Gallus.gallus                       -----RDSKSENTDSSGTQSPKRHSGSYLVTSV---------------------------    2849


Ciona.intestinalis                  ------------------------------------------------------------    2570
Strongylocentrotus.purpuratus       VNTRRNIFSSLNKNSNSKSSSKSSLDSSKSGGKSTPKMTSKQKHESEAKPEEKKDKKSEK    2853
Danio.rerio                         ------------------------------------------------------------    2754
Xenopus.tropicalis                  ------------------------------------------------------------    2833
Xenopus.laevis                      ------------------------------------------------------------    2829
Ornithorhynchus.anatinus            ------------------------------------------------------------    2844
Monodelphis.domestica               ------------------------------------------------------------    2842
Mus.musculus                        ------------------------------------------------------------    2842
Rattus.norvegicus                   ------------------------------------------------------------    2842
Sorex.araneus                       ------------------------------------------------------------    2837
Octodon.degus                       ------------------------------------------------------------    2841
Heterocephalus                      ------------------------------------------------------------    2839
Jaculus.jaculus                     ------------------------------------------------------------    2841
Loxodonta.africana                  ------------------------------------------------------------    2836
Trichechus.manatus.latirostris      ------------------------------------------------------------    2838
Otolemur.garnettii                  ------------------------------------------------------------    2843
Callithrix.jacchus                  ------------------------------------------------------------    2842
Macaca.mulatta                      ------------------------------------------------------------    2843
Nomascus.leucogenys                 ------------------------------------------------------------    2844
Pongo.abelii                        ------------------------------------------------------------    2844
```

```
Homo.sapiens                        ------------------------------------------------------------   2843
Gorilla.gorilla                     ------------------------------------------------------------   2844
Pan.troglodytes                     ------------------------------------------------------------   2844
Pan.paniscus                        ------------------------------------------------------------   2844
Canis.lupus.familiaris              ------------------------------------------------------------   2844
Bos.taurus                          ------------------------------------------------------------   2851
Ovis.aries                          ------------------------------------------------------------   2852
Orcinus.orca                        ------------------------------------------------------------   2847
Sus.scrofa                          ------------------------------------------------------------   2847
Ceratotherium.simum.simum           ------------------------------------------------------------   2845
Equus.caballus                      ------------------------------------------------------------   2845
Felis.catus                         ------------------------------------------------------------   2845
Ailuropoda.melanoleuca              ------------------------------------------------------------   2844
Mustela.putorius.furo               ------------------------------------------------------------   2844
Odobenus.rosmarus.divergens         ------------------------------------------------------------   2844
Taeniopygia.guttata                 ------------------------------------------------------------   2837
Anolis.carolinensis                 ------------------------------------------------------------   2843
Gallus.gallus                       ------------------------------------------------------------   2849


Ciona.intestinalis                  ------------------------------------------------------------   2570
Strongylocentrotus.purpuratus       KRFSFLKLKIGGKKSDSDSEGKGKSKGFFSKKEKKSPKNLKVQTKSRSESDSMAEHVLPS   2913
Danio.rerio                         ------------------------------------------------------------   2754
Xenopus.tropicalis                  ------------------------------------------------------------   2833
Xenopus.laevis                      ------------------------------------------------------------   2829
Ornithorhynchus.anatinus            ------------------------------------------------------------   2844
Monodelphis.domestica               ------------------------------------------------------------   2842
Mus.musculus                        ------------------------------------------------------------   2842
Rattus.norvegicus                   ------------------------------------------------------------   2842
Sorex.araneus                       ------------------------------------------------------------   2837
Octodon.degus                       ------------------------------------------------------------   2841
Heterocephalus                      ------------------------------------------------------------   2839
Jaculus.jaculus                     ------------------------------------------------------------   2841
Loxodonta.africana                  ------------------------------------------------------------   2836
Trichechus.manatus.latirostris      ------------------------------------------------------------   2838
Otolemur.garnettii                  ------------------------------------------------------------   2843
Callithrix.jacchus                  ------------------------------------------------------------   2842
Macaca.mulatta                      ------------------------------------------------------------   2843
Nomascus.leucogenys                 ------------------------------------------------------------   2844
Pongo.abelii                        ------------------------------------------------------------   2844
Homo.sapiens                        ------------------------------------------------------------   2843
Gorilla.gorilla                     ------------------------------------------------------------   2844
Pan.troglodytes                     ------------------------------------------------------------   2844
Pan.paniscus                        ------------------------------------------------------------   2844
Canis.lupus.familiaris              ------------------------------------------------------------   2844
Bos.taurus                          ------------------------------------------------------------   2851
Ovis.aries                          ------------------------------------------------------------   2852
Orcinus.orca                        ------------------------------------------------------------   2847
Sus.scrofa                          ------------------------------------------------------------   2847
Ceratotherium.simum.simum           ------------------------------------------------------------   2845
Equus.caballus                      ------------------------------------------------------------   2845
Felis.catus                         ------------------------------------------------------------   2845
Ailuropoda.melanoleuca              ------------------------------------------------------------   2844
Mustela.putorius.furo               ------------------------------------------------------------   2844
Odobenus.rosmarus.divergens         ------------------------------------------------------------   2844
Taeniopygia.guttata                 ------------------------------------------------------------   2837
Anolis.carolinensis                 ------------------------------------------------------------   2843
Gallus.gallus                       ------------------------------------------------------------   2849


Ciona.intestinalis                  ------------------------------------------------------------   2570
Strongylocentrotus.purpuratus       PPLAELEPDLNMSGADLNFDRHLDELEAWNSDNDIDNEVMPDEFVEWDSGSDIFKEEMPE   2973
Danio.rerio                         ------------------------------------------------------------   2754
Xenopus.tropicalis                  ------------------------------------------------------------   2833
Xenopus.laevis                      ------------------------------------------------------------   2829
```

```
Ornithorhynchus.anatinus            ------------------------------------------------------------   2844
Monodelphis.domestica               ------------------------------------------------------------   2842
Mus.musculus                        ------------------------------------------------------------   2842
Rattus.norvegicus                   ------------------------------------------------------------   2842
Sorex.araneus                       ------------------------------------------------------------   2837
Octodon.degus                       ------------------------------------------------------------   2841
Heterocephalus                      ------------------------------------------------------------   2839
Jaculus.jaculus                     ------------------------------------------------------------   2841
Loxodonta.africana                  ------------------------------------------------------------   2836
Trichechus.manatus.latirostris      ------------------------------------------------------------   2838
Otolemur.garnettii                  ------------------------------------------------------------   2843
Callithrix.jacchus                  ------------------------------------------------------------   2842
Macaca.mulatta                      ------------------------------------------------------------   2843
Nomascus.leucogenys                 ------------------------------------------------------------   2844
Pongo.abelii                        ------------------------------------------------------------   2844
Homo.sapiens                        ------------------------------------------------------------   2843
Gorilla.gorilla                     ------------------------------------------------------------   2844
Pan.troglodytes                     ------------------------------------------------------------   2844
Pan.paniscus                        ------------------------------------------------------------   2844
Canis.lupus.familiaris              ------------------------------------------------------------   2844
Bos.taurus                          ------------------------------------------------------------   2851
Ovis.aries                          ------------------------------------------------------------   2852
Orcinus.orca                        ------------------------------------------------------------   2847
Sus.scrofa                          ------------------------------------------------------------   2847
Ceratotherium.simum.simum           ------------------------------------------------------------   2845
Equus.caballus                      ------------------------------------------------------------   2845
Felis.catus                         ------------------------------------------------------------   2845
Ailuropoda.melanoleuca              ------------------------------------------------------------   2844
Mustela.putorius.furo               ------------------------------------------------------------   2844
Odobenus.rosmarus.divergens         ------------------------------------------------------------   2844
Taeniopygia.guttata                 ------------------------------------------------------------   2837
Anolis.carolinensis                 ------------------------------------------------------------   2843
Gallus.gallus                       ------------------------------------------------------------   2849


Ciona.intestinalis                  ------------------------------------------------------------   2570
Strongylocentrotus.purpuratus       EFIESCSEVDTDPATPLSPGVTTGPGGGNIQIDPNGQRGGFSPHSETGSLSPNGLSGDCM   3033
Danio.rerio                         ------------------------------------------------------------   2754
Xenopus.tropicalis                  ------------------------------------------------------------   2833
Xenopus.laevis                      ------------------------------------------------------------   2829
Ornithorhynchus.anatinus            ------------------------------------------------------------   2844
Monodelphis.domestica               ------------------------------------------------------------   2842
Mus.musculus                        ------------------------------------------------------------   2842
Rattus.norvegicus                   ------------------------------------------------------------   2842
Sorex.araneus                       ------------------------------------------------------------   2837
Octodon.degus                       ------------------------------------------------------------   2841
Heterocephalus                      ------------------------------------------------------------   2839
Jaculus.jaculus                     ------------------------------------------------------------   2841
Loxodonta.africana                  ------------------------------------------------------------   2836
Trichechus.manatus.latirostris      ------------------------------------------------------------   2838
Otolemur.garnettii                  ------------------------------------------------------------   2843
Callithrix.jacchus                  ------------------------------------------------------------   2842
Macaca.mulatta                      ------------------------------------------------------------   2843
Nomascus.leucogenys                 ------------------------------------------------------------   2844
Pongo.abelii                        ------------------------------------------------------------   2844
Homo.sapiens                        ------------------------------------------------------------   2843
Gorilla.gorilla                     ------------------------------------------------------------   2844
Pan.troglodytes                     ------------------------------------------------------------   2844
Pan.paniscus                        ------------------------------------------------------------   2844
Canis.lupus.familiaris              ------------------------------------------------------------   2844
Bos.taurus                          ------------------------------------------------------------   2851
Ovis.aries                          ------------------------------------------------------------   2852
Orcinus.orca                        ------------------------------------------------------------   2847
Sus.scrofa                          ------------------------------------------------------------   2847
Ceratotherium.simum.simum           ------------------------------------------------------------   2845
```

```
Equus.caballus                     ------------------------------------------------------------    2845
Felis.catus                        ------------------------------------------------------------    2845
Ailuropoda.melanoleuca             ------------------------------------------------------------    2844
Mustela.putorius.furo              ------------------------------------------------------------    2844
Odobenus.rosmarus.divergens        ------------------------------------------------------------    2844
Taeniopygia.guttata                ------------------------------------------------------------    2837
Anolis.carolinensis                ------------------------------------------------------------    2843
Gallus.gallus                      ------------------------------------------------------------    2849


Ciona.intestinalis                 ------------------------------------------------------------    2570
Strongylocentrotus.purpuratus      SPVDEGDLSMEAGEQYFYSENRLNSFIRLDDTKGETSDTFGEGELSISRNVQNQRSRSLS    3093
Danio.rerio                        ------------------------------------------------------------    2754
Xenopus.tropicalis                 ------------------------------------------------------------    2833
Xenopus.laevis                     ------------------------------------------------------------    2829
Ornithorhynchus.anatinus           ------------------------------------------------------------    2844
Monodelphis.domestica              ------------------------------------------------------------    2842
Mus.musculus                       ------------------------------------------------------------    2842
Rattus.norvegicus                  ------------------------------------------------------------    2842
Sorex.araneus                      ------------------------------------------------------------    2837
Octodon.degus                      ------------------------------------------------------------    2841
Heterocephalus                     ------------------------------------------------------------    2839
Jaculus.jaculus                    ------------------------------------------------------------    2841
Loxodonta.africana                 ------------------------------------------------------------    2836
Trichechus.manatus.latirostris     ------------------------------------------------------------    2838
Otolemur.garnettii                 ------------------------------------------------------------    2843
Callithrix.jacchus                 ------------------------------------------------------------    2842
Macaca.mulatta                     ------------------------------------------------------------    2843
Nomascus.leucogenys                ------------------------------------------------------------    2844
Pongo.abelii                       ------------------------------------------------------------    2844
Homo.sapiens                       ------------------------------------------------------------    2843
Gorilla.gorilla                    ------------------------------------------------------------    2844
Pan.troglodytes                    ------------------------------------------------------------    2844
Pan.paniscus                       ------------------------------------------------------------    2844
Canis.lupus.familiaris             ------------------------------------------------------------    2844
Bos.taurus                         ------------------------------------------------------------    2851
Ovis.aries                         ------------------------------------------------------------    2852
Orcinus.orca                       ------------------------------------------------------------    2847
Sus.scrofa                         ------------------------------------------------------------    2847
Ceratotherium.simum.simum          ------------------------------------------------------------    2845
Equus.caballus                     ------------------------------------------------------------    2845
Felis.catus                        ------------------------------------------------------------    2845
Ailuropoda.melanoleuca             ------------------------------------------------------------    2844
Mustela.putorius.furo              ------------------------------------------------------------    2844
Odobenus.rosmarus.divergens        ------------------------------------------------------------    2844
Taeniopygia.guttata                ------------------------------------------------------------    2837
Anolis.carolinensis                ------------------------------------------------------------    2843
Gallus.gallus                      ------------------------------------------------------------    2849


Ciona.intestinalis                 ------------------------------------------------------------    2570
Strongylocentrotus.purpuratus      DTAVEQSPHRPQRLKLPQKPFGFHQNDASPSSNNVVPVLVSPYNYSPNPNRKGEIDTNYS    3153
Danio.rerio                        ------------------------------------------------------------    2754
Xenopus.tropicalis                 ------------------------------------------------------------    2833
Xenopus.laevis                     ------------------------------------------------------------    2829
Ornithorhynchus.anatinus           ------------------------------------------------------------    2844
Monodelphis.domestica              ------------------------------------------------------------    2842
Mus.musculus                       ------------------------------------------------------------    2842
Rattus.norvegicus                  ------------------------------------------------------------    2842
Sorex.araneus                      ------------------------------------------------------------    2837
Octodon.degus                      ------------------------------------------------------------    2841
Heterocephalus                     ------------------------------------------------------------    2839
Jaculus.jaculus                    ------------------------------------------------------------    2841
Loxodonta.africana                 ------------------------------------------------------------    2836
Trichechus.manatus.latirostris     ------------------------------------------------------------    2838
```

```
Otolemur.garnettii                    ------------------------------------------------------------    2843
Callithrix.jacchus                    ------------------------------------------------------------    2842
Macaca.mulatta                        ------------------------------------------------------------    2843
Nomascus.leucogenys                   ------------------------------------------------------------    2844
Pongo.abelii                          ------------------------------------------------------------    2844
Homo.sapiens                          ------------------------------------------------------------    2843
Gorilla.gorilla                       ------------------------------------------------------------    2844
Pan.troglodytes                       ------------------------------------------------------------    2844
Pan.paniscus                          ------------------------------------------------------------    2844
Canis.lupus.familiaris                ------------------------------------------------------------    2844
Bos.taurus                            ------------------------------------------------------------    2851
Ovis.aries                            ------------------------------------------------------------    2852
Orcinus.orca                          ------------------------------------------------------------    2847
Sus.scrofa                            ------------------------------------------------------------    2847
Ceratotherium.simum.simum             ------------------------------------------------------------    2845
Equus.caballus                        ------------------------------------------------------------    2845
Felis.catus                           ------------------------------------------------------------    2845
Ailuropoda.melanoleuca                ------------------------------------------------------------    2844
Mustela.putorius.furo                 ------------------------------------------------------------    2844
Odobenus.rosmarus.divergens           ------------------------------------------------------------    2844
Taeniopygia.guttata                   ------------------------------------------------------------    2837
Anolis.carolinensis                   ------------------------------------------------------------    2843
Gallus.gallus                         ------------------------------------------------------------    2849


Ciona.intestinalis                    ----------------------------   2570
Strongylocentrotus.purpuratus         GMGFHKDDSSRSSSEVVSPTGSTRVTTV   3181
Danio.rerio                           ----------------------------   2754
Xenopus.tropicalis                    ----------------------------   2833
Xenopus.laevis                        ----------------------------   2829
Ornithorhynchus.anatinus              ----------------------------   2844
Monodelphis.domestica                 ----------------------------   2842
Mus.musculus                          ----------------------------   2842
Rattus.norvegicus                     ----------------------------   2842
Sorex.araneus                         ----------------------------   2837
Octodon.degus                         ----------------------------   2841
Heterocephalus                        ----------------------------   2839
Jaculus.jaculus                       ----------------------------   2841
Loxodonta.africana                    ----------------------------   2836
Trichechus.manatus.latirostris        ----------------------------   2838
Otolemur.garnettii                    ----------------------------   2843
Callithrix.jacchus                    ----------------------------   2842
Macaca.mulatta                        ----------------------------   2843
Nomascus.leucogenys                   ----------------------------   2844
Pongo.abelii                          ----------------------------   2844
Homo.sapiens                          ----------------------------   2843
Gorilla.gorilla                       ----------------------------   2844
Pan.troglodytes                       ----------------------------   2844
Pan.paniscus                          ----------------------------   2844
Canis.lupus.familiaris                ----------------------------   2844
Bos.taurus                            ----------------------------   2851
Ovis.aries                            ----------------------------   2852
Orcinus.orca                          ----------------------------   2847
Sus.scrofa                            ----------------------------   2847
Ceratotherium.simum.simum             ----------------------------   2845
Equus.caballus                        ----------------------------   2845
Felis.catus                           ----------------------------   2845
Ailuropoda.melanoleuca                ----------------------------   2844
Mustela.putorius.furo                 ----------------------------   2844
Odobenus.rosmarus.divergens           ----------------------------   2844
Taeniopygia.guttata                   ----------------------------   2837
Anolis.carolinensis                   ----------------------------   2843
Gallus.gallus                         ----------------------------   2849
```
