# Supplementary material for: Curated multiple sequence alignment for the Adenomatous Polyposis Coli (APC) gene and accuracy of in silico pathogenicity predictions
Source: PLoS One. 2020 Aug 4;15(8):e0233673. doi: 10.1371/journal.pone.0233673 (PMC7402488; doi:10.1371/journal.pone.0233673)
Supplement: S3 Fig — PMSA was generated from the program Clustal Omega. Annotation as per S1 Fig. (PDF) [file pone.0233673.s003.pdf]

APC Alignment – 9 species with edits (2018)

Exon
Dimerization
Armadillo Repeat
Beta Catenin and SAMP Repeats (one)(two three)
PHA03307
MAP65_ASE1 Microtubule Association Family (basic region for microtubule binding)
EB1 Binding Site
DLG (Axin) Binding Site
Basic Region

CLUSTAL O(1.2.4) multiple sequence alignment

XP_783363.3 – *Strongylocentrotus purpuratus*
NP_001137312.1 – *Danio rerio*
NP_001084351.1 – *Xenopus laevis*
XP_004949340.1 – *Gallus gallus*
XP_007497871.1 – *Monodelphis domestica*
NP_031488.2 – *Mus musculus*
NP_001069454.2 – *Bos taurus*
XP_014996065.1 – *Macaca mulatta*
AAA03586.1 – *Homo sapiens*

```
XP_783363.3             --MSSYDQLLHQVESLKAENSHLKMELKDNSSQLNKLDSVVGHLQSGKP---GMGEDDLR      55
NP_001137312.1          MAAASYDQLLKQVEALKMENSNLRQELEDNSNHLNKLETEASNMKEVLKQLQGSIDEDSK      60
NP_001084351.1          MAAASYDQLVKQVEALTMENTNLRQELEDNSNHLTKLETEATNMKEVLKQLQGSIEDEAM      60
XP_004949340.1          MAAASYDQLLKQVEALKMENSNLRQELEDNSNHLTKLETEASNMKEVLKQLQGSIEDEAI      60
XP_007497871.1          MAAASYDQLLKQVEALKMENSNLRQELEDNSNHLTKLETEASNMKEVLKQLQGSIEDEAM      60
NP_031488.2             MAAASYDQLLKQVEALKMENSNLRQELEDNSNHLTKLETEASNMKEVLKQLQGSIEDETM      60
NP_001069454.2          MAAASYDQLLKQVEALKMENSNLRQELEDNSNHLTKLETEASNMKEVLKQLQGSIEDEAM      60
XP_014996065.1          MAAASYDQLLKQVEALKMENSNLRQELEDNSNHLTKLETEASNMKEVLKQLQGSIEDEAM      60
AAA03586.1              MAAASYDQLLKQVEALKMENSNLRQELEDNSNHLTKLETEASNMKEVLKQLQGSIEDEAM      60
                           :*****::***:*. **::*: **:***.:*.**:: . :::.      *

XP_783363.3             AQITIHGLAMTTAAASATSSSAATSG---AGPASSFQVNGGAMLPGKPHSLPTS      100
NP_001137312.1          DSQGQIEFLERIKEMSLDPSGFSGVKLRSKASLQGSA---------DSSPSPSP      105
NP_001084351.1          ASSGPIDLLERFKDLNLDSSNIPAGKARPKMSMRSYGSREGSLSGHSGECSPVP      114
XP_004949340.1          ASSGQIDLLERLKELNLESTSFPGVKLRQKVSVRSYGSREGSVSSRSGECSPVP      114
XP_007497871.1          ASSGQIDLLERLKELNLDSSNFPGVKLRPKMSIRSYGSREGSVSSRSGECSPVP      114
NP_031488.2             T-SGQIDLLERLKEFNLD-SNFPGVKLRSKMSLRSYGSREGSVSSRSGECSPVP      112
NP_001069454.2          ASSGQIDLLERLKELNLDSSNFPGVKLRSKMSLRSYGSREGSVSSRSGECSPVP      114
XP_014996065.1          ASSGQIDLLERLKELNLDSSNFPGVKLRSKMSLRSYGSREGSVSSRSGECSPVP      114
AAA03586.1              ASSGQIDLLERLKELNLDSSNFPGVKLRSKMSLRSYGSREGSVSSRSGECSPVP      114
                          *  ::  ::.  .*  :   .                .           .    *

XP_783363.3             LGTSGTDGSLRQTKDHFIRHQELERDRLLILNEQAKEENLRSQCLAQIQSLTRRIEDLPI      166
NP_001137312.1          VSSCPRRGASSGGRDSAGYLEELEKERSLLVAELEKEEKEKDWYYAQLQNLTKRIDSLPL      165
NP_001084351.1          VGSFQRRGLLNGSRESAGYMEELEKERLLLIAEHEKEEKEKRWYYAQLQNLTKRIDSLPL      174
XP_004949340.1          MGSFPRRGFMNGSRESTGYLEELEKERSLLLAELEKEEKEKDWYYAQLQNLTKRIDSLPL      174
XP_007497871.1          MGSFSRRGFMNGSRESTGYLEELEKERSLLLAELEKEEKEKDWYYAQLQNLTKRIDSLPL      174
NP_031488.2             MGSFPRRTFVNGSRESTGYLEELEKERSLLLADLDKEEKEKDWYYAQLQNLTKRIDSLPL      172
NP_001069454.2          MGSFPRRGFVNGSRENTGYLEELEKERSLLLADLDKEEKEKDWYYAQLQNLTKRIDSLPL      174
XP_014996065.1          MGSFPRRGFVNGSRESTGYLEELEKERSLLLADLDKEEKEKDWYYAQLQNLTKRIDSLPL      174
AAA03586.1              MGSFPRRGFVNGSRESTGYLEELEKERSLLLADLDKEEKEKDWYYAQLQNLTKRIDSLPL      174
                        :.:          ::     :***::* *:: :  ***: :    **:*.**:**:.**:

XP_783363.3             TDNYSLQTDMSRRQLEYEARQLRELMQERLGSMEDIAARHRMRMQRLSAIEYELRQVQEQ      226
NP_001137312.1          TENFSLQTDMTRRQLEYEARQIRAAMEDQLGTCQDMEKRAQGRVARIQQIEKDMLRIRTR      225
NP_001084351.1          TENFSMQTDMTRRQLEYEARQIRAAMEEQLGTCQDMEKRVQTRVGKIHQIEEEILRIRQL      234
XP_004949340.1          TENFSLQTDMTRRQLEYEARQIRAAMEEQLGTCQDMEKRAQVRVARIQQIEKDILRIRQL      234
XP_007497871.1          TENFSLQTDMTRRQLEYEARQIRAAMEEQLGTCQDMEKRAQLRVARIQQIEKDILRIRQL      234
NP_031488.2             TENFSLQTDMTRRQLEYEARQIRAAMEEQLGTCQDMEKRAQRRIARIQQIEKDILRVRQL      232
NP_001069454.2          TENFSLQTDMTRRQLEYEARQIRVAMEEQLGTCQDMEKRAQRRITRIQQIEKDILRIRQL      234
XP_014996065.1          TENFSLQTDMTRRQLEYEARQIRVAMEEQLGTCQDMEKRAQRRIARIQQIEKDILRIRQL      234
AAA03586.1              TENFSLQTDMTRRQLEYEARQIRVAMEEQLGTCQDMEKRAQRRIARIQQIEKDILRIRQL      234
                        *:*:*:****:**********:*  *:::**: :*:  * : *: ::  ** :: :::

XP_783363.3             QQQHQQQQQQQQQQQQQQQQQMLAKVWEQGAQ----------------------------      259
NP_001137312.1          LQAQSAESESSGKR-YRERVKHEPLSQTEGSHAAGDAGAAAAASVCSQGSASRVDHDSAS      284
NP_001084351.1          LQSQVAEAAERTPQSKHDAGSRDAEKLPDGQGTSE--ITASGNVGSGQGSSSRADHDTTS      292
XP_004949340.1          LQSQAAE-AERAPQGKHDAASHDTERQSEGQGAPE--ISMS-TSNTGQGSAARMDHETAS      290
XP_007497871.1          LQSQPAE-AERASQSKPDHASHEAERPNEGPGAAD--VSVA-PPAGSQGSVAQVDQETAS      290
```

```
NP_031488.2         LQSQAAE-AERSSQSRHDAASHEAGRQHEGHGVAE--SNTA-ASSSGQSPATRVDHETAS     288
NP_001069454.2      LQSQATE-AERSSQSKHEAGSHEAERQNEGQGVAE--INMA-TSGSGQGSTTRIDHETAS     290
XP_014996065.1      LQSQATE-AERSSQNKHETGSHDAERQNEGQGVAE--INMA-TSGNGQGSTTRMDHETAS     290
AAA03586.1          LQSQATE-AERSSQNKHETGSHDAERQNEGQGVGE--INMA-TSGNGQGSTTRMDHETAS     290
                     * :  :  .                  :

XP_783363.3         ---------------------VGFVYSLLSMLGSHDRDDMASTLLMMSRSADSCLAMRQS     287
NP_001137312.1      EMSSAGSYSVPRRLTSHLGTKVEMVYSLLSMLGTHDKDDMSRTLLAMSSSQDSCIAMRQS     344
NP_001084351.1      VMSSNSTYSVPRRLTSHLGTKVEMVYSLLSMLGTHDKDDMSRTLLAMSSSQDSCIAMRQS     352
XP_004949340.1      VMSSSNNYSVPRRLTSHLGTKVEMVYSLLSMLGTHDKDDMSRTLLAMSSSQDSCIAMRQS     350
XP_007497871.1      GGSANGAYSVPRRLTSHLGTKVEMVYSLLSMLGTHDKDDMSRTLLAMSSSQDSCISMRQS     350
NP_031488.2         VLSSSGTHSAPRRLTSHLGTKVEMVYSLLSMLGTHDKDDMSRTLLAMSSSQDSCISMRQS     348
NP_001069454.2      VLSSSSTHSAPRRLTSHLGTKVEMVYSLLSMLGTHDKDDMSRTLLAMSSSQDSCISMRQS     350
XP_014996065.1      VLSSSSTHSAPRRLTSHLGTKVEMVYSLLSMLGTHDKDDMSRTLLAMSSSQDSCISMRQS     350
AAA03586.1          VLSSSSTHSAPRRLTSHLGTKVEMVYSLLSMLGTHDKDDMSRTLLAMSSSQDSCISMRQS     350
                                  . . *::* :*********:**:***: *** ** * ***::****

XP_783363.3         GCIPLLIHILHGTDQESV-LGNFRGSQKARDCASTALHNIVHLNPDEKRRKQEGRVLRLL     357
NP_001137312.1      GCLPLLIQLLHGNDKDSVLLGNSRGSKEARARASAALHNIIHSQPDDKRGRREIRVLHLL     404
NP_001084351.1      GCLPLLIQLLHGNDKDSVLLGNSRGSKEARASGSAALDNIIHSQPDDKRGRREIRVLHLL     412
XP_004949340.1      GCLPLLIQLLHGNDKDSVLLGNSRGSKEARARASAALHNIIHSQPDDKRGRREIRVLHLL     410
XP_007497871.1      GCLPLLIQLLHGNDKDSVLLGNSRGSKEARARASAALHNIIHSQPDDKRGRREIRVLHLL     410
NP_031488.2         GCLPLLIQLLHGNDKDSVLLGNSRGSKEARARASAALHNIIHSQPDDKRGRREIRVLHLL     408
NP_001069454.2      GCLPLLIQLLHGNDKDSVLLGNSRGSKEARARASAALHNIIHSQPDDKRGRREIRVLHLL     410
XP_014996065.1      GCLPLLIQLLHGNDKDSVLLGNSRGSKEARARASAALHNIIHSQPDDKRGRREIRVLHLL     410
AAA03586.1          GCLPLLIQLLHGNDKDSVLLGNSRGSKEARARASAALHNIIHSQPDDKRGRREIRVLHLL     410
                    **:****::***.*::** *** ***::**  .*:**.**:* :**:** ::* ***:**

XP_783363.3         EQIRTYCDSLVEAETKES-----ASSQNALPIDHNPGPAMAALMKLSFDEEHRSAICHLG     412
NP_001137312.1      EQIRAYCETCWEWQESHERGVDQDKNPMPSPVEHQICPAVCVLMKLSFDEEHRHAMNELG     464
NP_001084351.1      EQIRAYCETCWEWQEAHEQGMDQDKNPMPAPVDHQICPAVCVLMKLSFDEEHRHAMNELG     472
XP_004949340.1      EQIRAYCETCWEWQEAHEQGMDQDKNPMPAPVDHQICPAVCVLMKLSFDEEHRHAMNELG     470
XP_007497871.1      EQIRAYCETCWQWQEAHEQGMDQDKNPMPAPVEHQICPAVCVLMKLSFDEEHRHAMNELG     470
NP_031488.2         EQIRAYCETCWEWQEAHEQGMDQDKNPMPAPVEHQICPAVCVLMKLSFDEEHRHAMNELG     468
NP_001069454.2      EQIRAYCETCWEWQEAHEQGMDQDKNPMPAPVEHQICPAVCVLMKLSFDEEHRHAMNELG     470
XP_014996065.1      EQIRAYCETCWEWQEAHEQGMDQDKNPMPAPVEHQICPAVCVLMKLSFDEEHRHAMNELG     470
AAA03586.1          EQIRAYCETCWEWQEAHEPGMDQDKNPMPAPVEHQICPAVCVLMKLSFDEEHRHAMNELG     470
                    ****:**::  :  :   ..     ..    *::*:  **:..*********** *: .**

XP_783363.3         GLHAIAELLQVDYEVHGSSNDQYTVTLRRYAGMALTNLTFGDVTNKALLCSMKGCMKALV     472
NP_001137312.1      GLQAIGELLQVDCEIYGLTNDHYSVTLRRYAGMALTNLTFGDVANKATLCSMKGCMRAMV     524
NP_001084351.1      GLQAIAELLQVDCEMYGLINDHYSVTLRRYAGMALTNLTFGDVANKATLCSMKSCMRALV     532
XP_004949340.1      GLQAIAELLQVDCEMYGLTNDHYSVTLRRYAGMALTNLTFGDVANKATLCSMKGCMRALV     530
XP_007497871.1      GLQAIAELLQVDCEMYGLTSDHYSVTLRRYAGMALTNLTFGDVANKATLCSMKGCMRALV     530
NP_031488.2         GLQAIAELLQVDCEMYGLTNDHYSVTLRRYAGMALTNLTFGDVANKATLCSMKGCMRALV     528
NP_001069454.2      GLQAIAELLQVDCEMYGLTNDHYSITLRRYAGMALTNLTFGDVANKATLCSMKGCMRALV     530
XP_014996065.1      GLQAIAELLQVDCEMYGLTNDHYSITLRRYAGMALTNLTFGDVANKATLCSMKGCMRALV     530
AAA03586.1          GLQAIAELLQVDCEMYGLTNDHYSITLRRYAGMALTNLTFGDVANKATLCSMKGCMRALV     530
                    **:**.****** *::*  .*:*::******************:*** *****.**:*:*

XP_783363.3         ALLSAESEDLRQVAASVLRNLSWRADMASKKALREAGAVVALMTCSLEVKKESTLKSVLS     532
NP_001137312.1      AQLKSESEDLQQVIASVLRNLSWRADVNSKKILREVGSVRALMECALEVQKESTLKSVLS     584
NP_001084351.1      AQLKSESEDLQQVIASVLRNLSWRADVNSKKTLREVGSVKALMECALDVKKESTLKSVLS     592
XP_004949340.1      AQLKSESEDLQQVIASVLRNLSWRADVNSKKTLREVGSVKALMECALEVKKESTLKSVLS     590
XP_007497871.1      AQLKSESEDLEQVIASVLRNLSWRADVNSKKTLREVGSVKALMECALEVKKESTLKSVLS     590
NP_031488.2         AQLKSESEDLQQVIASVLRNLSWRADVNSKKTLREVGSVKALMECALEVKKESTLKSVLS     588
NP_001069454.2      AQLQSESEDLQQVIASVLRNLSWRADVNSKKTLREVGSVKALMECALEVKKESTLKSVLS     590
XP_014996065.1      AQLKSESEDLQQVIASVLRNLSWRADVNSKKTLREVGSVKALMECALEVKKESTLKSVLS     590
AAA03586.1          AQLKSESEDLQQVIASVLRNLSWRADVNSKKTLREVGSVKALMECALEVKKESTLKSVLS     590
                    * *.:*****.** ************: *** ***.*:* *** *:*:*:**********

XP_783363.3         ALWNLSAHCTENKADICAVNGALEFLVSSLTYRSPTRNSAVVENGGGILRNVSSHIATSE     592
NP_001137312.1      ALWNLSAHCTENKADICTVPGALAFLVSTLTYRSQTNTLAIIESGGGILRNVSSLIATNE     644
NP_001084351.1      ALWNLSAHCTENKADICSVDGALAFLVSTLTYRSQTNTLAIIESGGGILRNVSSLIATNE     652
XP_004949340.1      ALWNLSAHCTENKADICAVDGALAFLVGTLTYRSQTNTLAIIESGGGILRNVSSLIATNE     650
XP_007497871.1      ALWNLSAHCTENKADICAVDGALAFLVGTLTYRSQTNTLAIIESGGGILRNVSSLIATNE     650
NP_031488.2         ALWNLSAHCTENKADICAVDGALAFLVGTLTYRSQTNTLAIIESGGGILRNVSSLIATNE     648
NP_001069454.2      ALWNLSAHCTENKADICAVDGALAFLVGTLTYRSQTNTLAIIESGGGILRNVSSLIATNE     650
XP_014996065.1      ALWNLSAHCTENKADICAVDGALAFLVGTLTYRSQTNTLAIIESGGGILRNVSSLIATNE     650
AAA03586.1          ALWNLSAHCTENKADICAVDGALAFLVGTLTYRSQTNTLAIIESGGGILRNVSSLIATNE     650
                    *****************:* *** ***.:***** *.. *::*.********** ***.*
```

```
XP_783363.3         KYRQLLRKHNCLQILLHHLKSSSLTIVSNACGTLWNLSARNKADQDLLWELGAVSMLKNL     652
NP_001137312.1      EHRQILRENSCLQTLLQHLKSHSLTIVSNACGTLWNLSARNAKDQEALWDMGAVSMLKNL     704
NP_001084351.1      DHRQILRENNCLQTLLQHLKSHSLTIVSNACGTLWNLSARNAKDQEGLWDMGAVSMLKNL     712
XP_004949340.1      DHRQILRENSCLQTLLQHLKSHSLTIVSNACGTLWNLSARNAKDQEALWDMGAVSMLKNL     710
XP_007497871.1      DHRQILRENSCLQTLLQHLKSHSLTIVSNACGTLWNLSARNTKDQEALWDMGAVSMLKNL     710
NP_031488.2         DHRQILRENNCLQTLLQHLKSHSLTIVSNACGTLWNLSARNPKDQEALWDMGAVSMLKNL     708
NP_001069454.2      DHRQILRENNCLQTLLQHLKSHSLTIVSNACGTLWNLSARNPKDQEALWDMGAVSMLKNL     710
XP_014996065.1      DHRQILRENNCLQTLLQHLKSHSLTIVSNACGTLWNLSARNPKDQEALWDMGAVSMLKNL     710
AAA03586.1          DHRQILRENNCLQTLLQHLKSHSLTIVSNACGTLWNLSARNPKDQEALWDMGAVSMLKNL     710
                    .:**:**::.*** **:**** ******************   **: **::*********

XP_783363.3         ISSKHKMIAMGSSAALRNLMASRPDVLATADGQK--EGTPGLHVRKQRALQAEIDKN-LK     709
NP_001137312.1      IHSKHKMIAMGSAAALRNLMANRPAKYKDANIMSPGSSLPSLHVRKQKALIEELDAQHLS     764
NP_001084351.1      IHSKHKMIAMGSAAALRNLMANRPAKYKDANIMSPGSSVPSLHVRKQKALEAELDAQHLS     772
XP_004949340.1      IHSKHKMIAMGSAAALRNLMANRPAKYKDTNIMSPGSSLPSLHVRKQKALEAELDAQHLS     770
XP_007497871.1      IHSKHKMIAMGSAAALRNLMANRPAKYKDANIMSPGSSLPSLHVRKQKALEAELDAQHLS     770
NP_031488.2         IHSKHKMIAMGSAAALRNLMANRPAKYKDANIMSPGSSLPSLHVRKQKALEAELDAQHLS     768
NP_001069454.2      IHSKHKMIAMGSAAALRNLMANRPAKYKDANIMSPGSSLPSLHVRKQKALEAELDAQHLS     770
XP_014996065.1      IHSKHKMIAMGSAAALRNLMANRPAKYKDANIMSPGSSLPSLHVRKQKALEAELDAQHLS     770
AAA03586.1          IHSKHKMIAMGSAAALRNLMANRPAKYKDANIMSPGSSLPSLHVRKQKALEAELDAQHLS     770
                    * **********:********.**      ::  .  .. *.******:**  *:* : *.

XP_783363.3         DTYAEMEGRTDQHGLLQSQQRASLRNRGHRSRQQQHGP---DYSPV-------PQRIPIW     759
NP_001137312.1      ETFDNIDNLSPKA--------------SHRVKPRHKHNVYGDY--------DAVCRSDGY     802
NP_001084351.1      ETFDNIDNLSPKT--------------THRNKQRHKQNLCSEYALDSSRHDDSICRSDNF     818
XP_004949340.1      ETFDNIDNLSPKA--------------SHRNKQRHKQNIYGEYVLDSSRHDDGVCRTESF     816
XP_007497871.1      ETFDNIDNLSPKT--------------SHRPKQRHKQSVYGEYALDASRHDDS--RPDAF     814
NP_031488.2         ETFDNIDNLSPKA--------------SHRSKQRHKQNLYGDYAFDANRHDDS--RSDNF     812
NP_001069454.2      ETFDNIDNLSPKA--------------SHRSKQRHKQNLYGDYVFDTNRHDDN--RSDNF     814
XP_014996065.1      ETFDNIDNLSPKA--------------SHRSKQRHKQSLYGDYVFDTNRHEDN--RSDNF     814
AAA03586.1          ETFDNIDNLSPKA--------------SHRSKQRHKQSLYGDYVFDTNRHDDN--RSDNF     814
                    :*: :::. : :                ** : :::    :*            *   :

XP_783363.3         NPNATPLPDSLMNSQQRAHSPSNRHHGNMINSPETCSDDQSFQEGKTKD-SEGQSTSSQP     818
NP_001137312.1      NPNGVGVRSPYMNTPVLSSPSSRDNRGNAESVRA--ERDRSLDRERRGFLPD--------     852
NP_001084351.1      SIGNLTVLSPYINTTVLPGSS-S-PRPTMDGSRP--EK----DRERTAGLGNYHSTTESS     870
XP_004949340.1      NTGNMTVLSPYLNSTVLPGSA-SSSRGNIENCLS--EKDRSLDRDRAVGLNAYHPATENS     873
XP_007497871.1      STGNLTVLSPYLNSTVLPGSS--SSRTSLESSRS--EKDRSLDRERAVALSTFHPAADSP     870
NP_031488.2         NTGNMTVLSPYLNTTVLPSSS--SSRGSLDSSRS--EKDRSLERERGIGLSAYHPTTENA     868
NP_001069454.2      NTGNMTVLSPYLNTTVLPSSS--SSRGSLDSSRS--EKDRSLERERGISLGNYHPATENP     870
XP_014996065.1      NAGNMTVLSPYLNTTVLPSSS--SSRGSLDSSRS--EKDRSLERERGIGLGNYHPATENP     870
AAA03586.1          NTGNMTVLSPYLNTTVLPSSS--SSRGSLDSSRS--EKDRSLERERGIGLGNYHPATENP     870
                    . .   : .  :*:           : .  .     .     :. :

XP_783363.3         GSVE--NSPGRPHGASRIAQIMQEVAQDGLPTDSS-SGSESPRRESLESRLQSRNGHQQK     875
NP_001137312.1      -GEAAKRMMQIPTSAAQIAVVMEEVQNMHLGMDDRSAGSTPDPHSVQDD--MIRRQ---T     906
NP_001084351.1      GNSSKRIGIQLSTT-AQISKVMDEVSNIHLVQENRSSGSASEMHCMSDERNSQRKP---S     926
XP_004949340.1      GNSSKRIGMQISTAAAQIAKVMEEVTSMHIPQEDRSSGSTSEMHCLTEDRNTTRRA---A     930
XP_007497871.1      GNPSKRLGMQLSTTTAQIAKVMEEVSAIHAQ-EDQSSASTTDLHCVAEERSTLRRA---S     926
NP_031488.2         GTSSK-RGLQITTTAAQIAKVMEEVSAIHTSQDDRSSASTTEFHCVADDRSAARRS---S     924
NP_001069454.2      GTSSK-RGLQISTTAAQIAKVMEEVSAIHTSQEDRSSGSTTELHCGTDERNALRRS---S     926
XP_014996065.1      GTSSK-RGLQISTTAAQIAKVMEEVSAIHTSQEDRSSGSTTELHCVTDERNALRRS---S     926
AAA03586.1          GTSSK-RGLQISTTAAQIAKVMEEVSAIHTSQEDRSSGSTTELHCVTDERNALRRS---S     926
                                   ::*: :*:**       :.  :.*    :   :.    *.

XP_783363.3         PTCPRSSSFTHMPPEGSNLSSRSNSYCFGFDGGHGLVARRSSTESINSISSDIFPAGIHE     935
NP_001137312.1      AVHGHQNIYSYSKTDPSG------RPCPM--PKL--EY-RASNDSLNSVNSTD---GYG-     951
NP_001084351.1      SNHPQSNPFTFTKAESST------RGCPV--AFMKMEYKMASNDSLNSVSSTE---GYG-     974
XP_004949340.1      TAHTHSNTY-FPKSENSS------RPCPV--PYTKMEYKRASNDSLNSVSSSD---GYG-     977
XP_007497871.1      AAHAHSNTYNFPKPDNSN------RTCAM--PYAKVEYKRSSNDSLNSVSSSD---GYG-     974
NP_031488.2         ASHTHSNTYNFTKSENSN------RTCSM--PYAKVEYKRSSNDSLNSVTSSD---GYG-     972
NP_001069454.2      TTHTHANTYNFTKSENSN------RTCPI--PYAKVEYKRSSNDSLNSVSSSD---GYG-     974
XP_014996065.1      AAHTHSNTYNFTKSENSN------RTCSM--PYAKLEYKRSSNDSLNSVSSSD---GYG-     974
AAA03586.1          AAHTHSNTYNFTKSENSN------RTCSM--PYAKLEYKRSSNDSLNSVSSSD---GYG-     974
                        : . : .   : *         *             :*.:*:**:.*     *

XP_783363.3         RLAQNRSQMDHSQ---SADSSLNMHGTGRSLQNTTALVHSAD--EAFGTNMDSTTNYSLK     990
NP_001137312.1      ----KRGQMKPSVDSYSEDDEGKCCVYRKYPADLAHKIHNANHMEDDNGDLDTPINYSLK     1007
NP_001084351.1      ----KRGQVKPSVESYSEDDESKFFSYGQYPAGLAHKIQSANHMDDNDTELDTPINYSLK     1030
XP_004949340.1      ----KRGQMKPSIESYSEDDESKFCSYGQYPADLAHKIHSANHMDDNDGELDTPINYSLK     1033
XP_007497871.1      ----KRGQMKPSIESYSEDDESKFCSYGQYPADLAHKIHSANHMDDNDEELDTPINYSLK     1030
NP_031488.2         ----KRGQMKPSVESYSEDDESKFCSYGQYPADLAHKIHSANHMDDNDGELDTPINYSLK     1028
NP_001069454.2      ----KRGQMKPSIESYSEDDESKFCSYGQYPADLAHKIHSANHMDDNDGELDTPINYSLK     1030
```

```
XP_014996065.1          ----KRGQMKPSIESYSEDDESKFCSYGQYPADLAHKIHSANHMDDNDGELDTPINYSLK     1030
AAA03586.1              ----KRGQMKPSIESYSEDDESKFCSYGQYPADLAHKIHSANHMDDNDGELDTPINYSLK     1030
                            :*.*:.  *    * *.. :     :   . :  ::.*:  :  . ::*:  *****

XP_783363.3             YSEEDLPPGMHSPKRTAPVEH---RCKDGNSQH-----------SSVDGEQSEEG-----     1031
NP_001137312.1          YSDEQLNSGRQSPSQNERWARPK-LLDDEMKRPDQKPPRSQSPGYPMYTEGSSEGEDKPK     1066
NP_001084351.1          YSDEQLNSGRQSPTQNERWSRPKHIIDSEMKQSEQRQPRTTKTTYSSYTEN---KEEKHK     1087
XP_004949340.1          YSDEQLNSGRQSPSQNERWARPKHIIDDEMKQNDQRQSRSQSATYPVYTES---GDDKHM     1090
XP_007497871.1          YSDEQLNSGRQSPSQNDRWARPKHVIEDEIKQNEQRQARGQNTPFSAYSES---TDDKHM     1087
NP_031488.2             YSDEQLNSGRQSPSQNERWARPKHVIEDEIKQNEQRQARSQNTSYPVYSEN---TDDKHL     1085
NP_001069454.2          YSDEQLNSGRQSPSQNERWARPKHILEDEIKPNEQRQSRSQSTAYPVYPES---TDDKHL     1087
XP_014996065.1          YSDEQLNSGRQSPSQNERWARPKHIIEDEIKQSEQRQSRSQSTTYPVYTES---TDDKHL     1087
AAA03586.1              YSDEQLNSGRQSPSQNERWARPKHIIEDEIKQSEQRQSRNQSTTYPVYTES---TDDKHL     1087
                        **:*:*  * :**.:.    :      ..  .                  *

XP_783363.3             ---LSQPCQKCNSPHRPREGLAIGNSYPASQQQRPFPQQYSDQSVSSHHNVID-------     1081
NP_001137312.1          KYQPRFVQQD-LPAFRSR----GSNEQ---------------ISSGSSHGLNKKISQTIC     1106
NP_001084351.1          KFPPHFNQSENVPAYTRSRGANNQVDQ---------------SRVSSNLSNNSKASKPHC     1132
XP_004949340.1          KYQSPFGQQDCVPSFRSR-GS-NGSDQ---------------NRVGSTLGINQKVNQSLC     1133
XP_007497871.1          KFQSRFGQQECVSPYRSR-GA-SGSEQ---------------NRVSSGHGINQKVNQSLC     1130
NP_031488.2             KFQPHFGQQECVSPYRSR-GT-SGSET---------------NRMGSSHAINQNVNQSLC     1128
NP_001069454.2          KFQPHFGQQECVSPYRSR-AA-NGSET---------------NRVGSNHGISQNVNQSLC     1130
XP_014996065.1          KFQPHFGQQECVSPYRSR-GA-NGSET---------------NRVGSNHGINQNVSQSLC     1130
AAA03586.1              KFQPHFGQQECVSPYRSR-GA-NGSET---------------NRVGSNHGINQNVSQSLC     1130
                                ..    .          .                    .*     .

XP_783363.3             ---------PMQGSSMYSNMNDHE---------------PFAEEDERPTDFSQRYANDMS     1117
NP_001137312.1          SVDDYADDKPTNYSERYSEEEQLEEQ---TPSYSMK--YTEDHHVEQPIDYSLKYSEA--     1159
NP_001084351.1          QVDDYDDDKTTNFSERYSEEEQQEDETERQNKYNIKAYASEEHHGEQPIDYSRKYSTDVP     1192
XP_004949340.1          QVDDYDDDKPTNYSERYSEEEQHEE-EDRPTNYSIK-YNEEEHQVDQPIDYSLKYSTEVP     1191
XP_007497871.1          HEDDYDEDKPTNYSERYSEEEQHEE-EDRPTNYSMK-YNEEEHHADQPIDYSLKYAADIT     1188
NP_031488.2             QEDDYEDDKPTNYSERYSEEEQHEEEEERPTNYSIK-YNEEKHHVDQPIDYSLKYATDI-     1186
NP_001069454.2          QEDDYEDDKPTNYSERYSEEGQHEE-EERPTNYSIK-YSEEKHHVDQPIDYSLKYTTDI-     1187
XP_014996065.1          QEDDYEDDKPTNYSERYSEEEQHEE-EERPTNYSIK-YNEEKHHVDQPIDYSLKYATDI-     1187
AAA03586.1              QEDDYEDDKPTNYSERYSEEEQHEE-EERPTNYSIK-YNEEKRHVDQPIDYSLKYATDI-     1187
                                   :  *. **:  : *                    .. ::* *:* :*:

XP_783363.3             HGDDAESFGFVQTSLETNTGGTVYMSSEEPDVRDSSYNASSNQEIPSSVVSQFGSEHGGS     1177
NP_001137312.1          -PSKKGMFSHSKTSSAQSSAKEHLSQDSSSSVASLKNQGRQKQLHPSSAQSRSGPT----     1214
NP_001084351.1          SSAQKPSFPYSNNSSKQKPKKEQVSSNS-NTPTPSPNSNRQNQLHPNSAQSRPGLN----     1247
XP_004949340.1          PSSQKPSFTFSKTSSVQSTKTDHISSSSGNTSAPSAGSKRQNQLHPSSAQSRGGHA----     1247
XP_007497871.1          PSSQKPSFSFSKSSSVQSNKTGHISSGG-NTSTAPASTKRQNQLLPSSAQNRSGHT----     1243
NP_031488.2             SSSQKPSFSFSKNSSAQSTKPEHLSPSSENTAVPPSNAKRQNQLRPSSAQRN-GQT----     1241
NP_001069454.2          PSSQKPAFSFSKNSSGQSTKTEHISSSSENTSTTSSNAKRQNQLHPSSAQSRSGQT----     1243
XP_014996065.1          PSSQKQSFSFSKSSSGQSTKTEHISSSSENTSTPSSNAKRQNQLHPSSAQSRSGQT----     1243
AAA03586.1              PSSQKQSFSFSKSSSGQSSKTEHMSSSSENTSTPSSNAKRQNQLHPSSAQSRSGQP----     1243
                           .   * . :.*   .        .            .:*  *.*.  . *

XP_783363.3             RHSIASSHHEDEEEPPCSHDDNTKTYCVEGTPGPISRCSSLSSLDLNEELDEVEKTKDDS     1237
NP_001137312.1          -RA---VQKNPTCKAPTINQETLQTYCVEDTPTCFSRGSSLSSLSSEEDEMESCKRNVNS     1270
NP_001084351.1          -R-----PKQIPNKPPSINQETIQTYCVEDTPICFSRGSSLSSLSSAEDEIEGRERNSRG     1301
XP_004949340.1          -------QKTASCKTPSINQETIQTYCVEDTPICFSRCSSLSSLSSAEDEI-GRDQSTRV     1299
XP_007497871.1          -------QKTASCKAPSINQETIQTYCVEDTPICFSRCSSLSSLSSAEDEI-GRDQTTRG     1295
NP_031488.2             -------QKGTTCKVPSINQETIQTYCVEDTPICFSRCSSLSSLSSADDEI-GCDQTTQE     1293
NP_001069454.2          -PK----ATSSSCKVPSINQETIQTYCVEDTPICFSRCSSLSSLSSAEDEV-GCDQTTQE     1297
XP_014996065.1          -QK----A--ATCKVSSINQETIQTYCVEDTPICFSRCSSLSSLSSAEDEI-GCDQTTQE     1295
AAA03586.1              -QK----A--ATCKVSSINQETIQTYCVEDTPICFSRCSSLSSLSSAEDEI-GCNQTTQE     1295
                                     :    :::. :*****.**  :** ******.  ::     . .

XP_783363.3             AHSPSNDLQSTTPPNEP--------------TPEKVTN------------------KQVL     1265
NP_001137312.1          A-SNYPTLPISEKQSTNN-VAADQRTSESQSSVHYVRAKPPRHHL--GHGDGSRHHKTVE     1326
NP_001084351.1          Q-ESNNTLQITEPKEISA-VSKDGAVNETRSSVHHTRTKNNRLQTSNISPSDSSRHKSVE     1359
XP_004949340.1          T-DTNATLQISELKENSGALSAEAAVSEITSTSQHIRTKSSRLPTSSLSPSESSRHKAVE     1358
XP_007497871.1          S-ETTNTLQIAELKENSASVSTGDTGSEVPSTSQHIRTKANRLQTTSLSPSDSTRHKAVE     1354
NP_031488.2             A-DSANTLQTAEVKENDVTRSAEDPATEVPAVSQNARAKPSRLQASGLSSESTRHNKAVE     1352
NP_001069454.2          A-ESANTLQIAEIKDNSGPRSNEDSVSKVPAGSQHIRTKSSRLQASGLSSESARH-KAVE     1355
XP_014996065.1          A-DSANTLQIAEIKDKIGTRSTEDPVSEVPAVSQHTRTKSSRLQGSSLSSESTRH-KAVE     1353
AAA03586.1              A-DSANTLQIAEIKEKIGTRSAEDPVSEVPAVSQHPRTKSSRLQGSSLSSESARH-KAVE     1353
                          .    *  :   .                  .                      * *


XP_783363.3             ------------LQLPSDVHHHDEVQETPLVFSRCSSVCSLSSDDVPDICDDVSSIYTNS     1313
NP_001137312.1          FSSGAKSPSKSGAQTP-KSPPEHYVQETPLMFSRCTSVSSLDSFESHSIASSVQSEP-CS     1384
```

```
NP_001084351.1        FSSGAKSPSKSGAQTP-KSPPEHYVQETPLMFSRCTSGSSLDSFESHSIASSIASSVASE      1418
XP_004949340.1        FSSGAKSPSKSGAQTP-KSPPEHYVQETPLMFSRCTSVSSLDSFESRSIASSVQSEP-CS      1416
XP_007497871.1        FSSGAKSPAKSGAQTP-KSPPEHYVQETPLMFSRCTSVSSLDSFESRSIASSVQSEP-CS      1412
NP_031488.2           FSSGAKSPSKSGAQTP-KSPPEHYVQETPLVFSRCTSVSSLDSFESRSIASSVQSEP-CS      1410
NP_001069454.2        FSSGAKSPSKSGAQTP-KSPPEHYVQETPLMFSRCTSVSSLDSFESRSIASSVQSEP-CS      1413
XP_014996065.1        FSSGAKSPSKSGAQTP-KSPPEHYVQETPLMFSRCTSVSSLDSFESRSIASSVQSEP-CS      1411
AAA03586.1            FSSGAKSPSKSGAQTP-KSPPEHYVQETPLMFSRCTSVSSLDSFESRSIASSVQSEP-CS      1411
                                   * * .   .. ******:****:* .**.* :  .*...: *    .

XP_783363.3           RAASGFVSPSELPDSPSDTMPPSPRRTSQS---------NVKVDL---------------      1349
NP_001137312.1        GMVSGIISPSDLPDSPGQTMPPSRSKTPPPPPPRSTS---VKQKV--TVPPHTEKHDLAP      1439
NP_001084351.1        HMISGIISPSDLPDSPGQTMPPSRSKTPP-----PPQTVQAKKDGSKPIVPDEERG--KV      1471
XP_004949340.1        GMVSGIISPSDLPDSPGQTMPPSRSKTPP-----PAQGVQVKRDVTKGKVPTAEKREPGP      1471
XP_007497871.1        GMVSGIISPSDLPDSPGQTMPPSRSKTPP-----PPQTGQTKREATKSKLPSAEKRESGP      1467
NP_031488.2           GMVSGIISPSDLPDSPGQTMPPSRSKTPP----PPPQTVQAKREVPKSKVPAAEKRESGP      1466
NP_001069454.2        GMVSGIISPSDLPDSPGQTMPPSRSKTPPPPPPPPPQTVQTKQEVPKNKAPSAEKRESGP      1473
XP_014996065.1        GIVSGIISPSDLPDSPGQTMPPSRSKTPPP----PPQTAQTKREVPKNKTPTAEKRESGP      1467
AAA03586.1            GMVSGIISPSDLPDSPGQTMPPSRSKTPPP----PPQTAQTKREVPKNKAPTAEKRESGP      1467
                         **::***:*****.:*****  :*              .* .

XP_783363.3           ---------LNAPKLPEMEEKPTMYATEGTPIDNSCATSLSALTIDGDVK-ATVAKRLLP      1399
NP_001137312.1        RHAVVSAAVQKVQVLPD-NDTLLHFATESTPDGFSCASSLSALSLDEPFIQKDVELKIMP      1498
NP_001084351.1        AKTAVHSAIQRVQVLQE-ADTLLHFATESTPDGFSCASSLSALSLDEPYIQKDVQLKIMP      1530
XP_004949340.1        RQAAVNAAVQRVQVLPD-ADTLLHFATESTPDGFSCSSSLSALSLDEPFIQKDVELRIMP      1530
XP_007497871.1        RQAAVNAAVQRVQVLPD-ADTLLHFATESTPDGFSCSSSLSALSLDEPFIQKDVELRLMP      1526
NP_031488.2           KQTAVNAAVQRVQVLPD-VDTLLHFATESTPDGFSCSSSLSALSLDEPFIQKDVELRIMP      1525
NP_001069454.2        KQAAVNAAVQRVQVLPE-ADTLLHFATESTPDGFSCSSSLSALSLDEPFIQKDVELRIMP      1532
XP_014996065.1        KQAAVNAAVQRVQVLPD-ADTLLHFATESTPDGFSCSSSLSALSLDEPFIQKDVELRIMP      1526
AAA03586.1            KQAAVNAAVQRVQVLPD-ADTLLHFATESTPDGFSCSSSLSALSLDEPFIQKDVELRIMP      1526
                                .. * :  :.   :***.** . **::*****::*        *  :::*

XP_783363.3           PKGNEGSENVGSEDKE-----ITDEKNVEPS-------GNEFSEQEQSLLDECINAAMPV      1447
NP_001137312.1        PVHEDDHSIEAEPEMEDMHEPKVQEKSPATSEAAKDILDDSDDDDDTEILNACINSAMPT      1558
NP_001084351.1        PVLENDQGNKAEPEKE--FIDNKAKKEDKRSEQEKDMLDDT--DDDIDILEECIISAMPR      1586
XP_004949340.1        PVHENEHGNEAEPEQSDETKDNQENKAEKPSEAEKDILDDSD-DDDIEILEECIISAMPT      1589
XP_007497871.1        PVQENEHGNETEPEQLEDVSEVKEKKAEKPTESEKDILDDSD-DDDIEILEECIISAMPT      1585
NP_031488.2           PVQENDNGNETESEQPEESNENQDKEVEKP-DSEKDLLDDSD-DDDIEILEECIISAMPT      1583
NP_001069454.2        PVQENDNGNETESEQPEESNENQEKEAEKPTDSEKDLLDESD-DDDIEILEECIISAMPT      1591
XP_014996065.1        PVQENDNGNETESEQPKESNENQEKEAEKTIDSEKDLLDDSD-DDDIEILEECIISAMPT      1585
AAA03586.1            PVQENDNGNETESEQPKESNENQEKEAEKTIDSEKDLLDDSD-DDDIEILEECIISAMPT      1585
                      *  ::      . :          ::              .:   ::: .:*: ** :***

XP_783363.3           RRKGIPKPT------------KSKSKREITSRKIPVSHKPSSSKHVSFEKSNAAPPTTMQ      1495
NP_001137312.1        KSSRKPKKQ---STSRIPPPVACKPSQLPVYKLLPPQNRGQPQKHVALA--------HGE      1607
NP_001084351.1        KPSRKNKKVPQPTPGKPPPPVARKPSQLPVYKLLSSQNRLQTQKHVNFT--------HSD      1638
XP_004949340.1        KSSRKAKKPSQASAPKIPPPVARKPSQLPVYKLLPSQSRLQSQKHVSFT--------PGD      1641
XP_007497871.1        KSSRKAKKPSQA-ASKIPPPVARKPSQLPVYKLLPSQNRLQAQKHVSFT--------PGD      1636
NP_031488.2           KSSRKAKKLAQT-ASKLPPPVARKPSQLPVYKLLPAQNRLQAQKHVSFT--------PGD      1634
NP_001069454.2        KSSRKAKKPAQT-TSKLPPPVARKPSQLPVYKLLPSQNRLQAQKHVSFT--------PGD      1642
XP_014996065.1        KSSRKAKKPAQT-ASKLPPPVARKPSQLPVYKLLPSQNRLQPQKHVSFT--------PGD      1636
AAA03586.1            KSSRKAKKPAQT-ASKLPPPVARKPSQLPVYKLLPSQNRLQPQKHVSFT--------PGD      1636
                      : .   *                *  .:  . : :  . :  . .*** :            :

XP_783363.3           DLPKPFCTEDTPVNFSAATSLSDLSIDDIEIESSSDGKLMIANPDRSALEKPSGIESDNQ      1555
NP_001137312.1        DMPRVYCVEGTPINFSTATSLSDLTIDSPPNELAGMESSA-PHVEA-SGQRRDTL-----      1660
NP_001084351.1        DMPRVYCVEGTPINFSTATSLSDLTIESPPSEPTND-QPN-TDSLSTDLEKRDTI-----      1691
XP_004949340.1        DMPRVYCVEGTPINFSTATSLSDLTIESPPNELANVDSVG-AGAESGEFEKRDTI-----      1695
XP_007497871.1        DVPRVYCVEGTPINFSTATSLSDLTIESPPNELAGVEGTS-TGALLGDFEKRDTI-----      1690
NP_031488.2           DVPRVYCVEGTPINFSTATSLSDLTIESPPNELATGDGVR-AGIQSGEFEKRDTI-----      1688
NP_001069454.2        DMPRVYCVEGTPINFSTATSLSDLTIESPPNELAAGEGVR-AGAQSSEFEKRDTI-----      1696
XP_014996065.1        DMPRVYCVEGTPINFSTATSLSDLTIESPPNELAAGEGVR-AGAQSGEFEKRDTI-----      1690
AAA03586.1            DMPRVYCVEGTPINFSTATSLSDLTIESPPNELAAGEGVR-GGAQSGEFEKRDTI-----      1690
                      *:*: :*.*.**:***:*******:*:.    * :              :: . :
XP_783363.3           VRTSGDLLHCMDDAR-----SETNSVTKEAEDSMLQECINSAIPRSKSCKPRSSLLAKHR      1610
NP_001137312.1        -PE-GKSAEAKETGLSPPM-QSA--LAENEGDDILAECINSAMPKSKIHKPFRVQKMP--      1713
NP_001084351.1        -PTEGRSTDDTDASKPLNP-TTVLDEDKAEEGDILAECIHSAMPKGKSHKPYRVKKIM--      1747
XP_004949340.1        -PTEGRSTDDTQRAKSITVTGPGLDDDKTEEGDILAECINSAMPKGKSHKPFRVKKIM--      1752
XP_007497871.1        -PTEGRSTDDIQIGKSSNVNTSAFDDNKTEEGEILAECINSAMPKGKSHKPFRVKKIM--      1747
NP_031488.2           -PTEGRSTDDAQRGKISSIVTPDLDDNKAEEGDILAECINSAMPKGKSHKPFRVKKIM--      1745
NP_001069454.2        -PTEGRSTDEAQRGKASSVTVPELDDSKTEEGDILAECINSAMPKGKSHKPFRVKKIM--      1753
XP_014996065.1        -PTEGRSTDEAQGGKTSSVTIPELDDNKAEEGDILAECINSAMPKGKSHKPFRVKKIM--      1747
AAA03586.1            -PTEGRSTDEAQGGKTSSVTIPELDDNKAEEGDILAECINSAMPKGKSHKPFRVKKIM--      1747
                          *   .  : .             :   ..:* ***:**:*:.*  **
```

```
XP_783363.3          SPRQIGKFRSPKGVAPRKRLPSADVRHKSPSPIPEMSSDTRSTSSQERGQDWGNNPSPPP      1670
NP_001137312.1       --DQAQHPSTATGS---LV-QQDLEKKKPTSPVKPMPQSSEYRARMLKRPEANNS----L      1763
NP_001084351.1       --DQINHTSAATSSGNSRS-MQETDKNKPTSPVKPMPQSIGFKERLKKNTELKLN----P      1800
XP_004949340.1       --DQIQQASTS---LNNKN-QPEGEKKKPTSPVKPVPQNSEYRARVRKNTESKSQ----I      1802
XP_007497871.1       --DQIQQASAS-SSGNSKN-PLDSEKKKPTSPVKPMPQSAEYRTRIRKNAESK-N----V      1798
NP_031488.2          --DQVQQASST-SSGANKN-QVDTKKKKPTSPVKPMPQNTEYRTRVRKNTDSKVN----V      1797
NP_001069454.2       --DQVQQASMS-SSGTNKN-QLDGKTKKPTSPVKPIPQNTEYRTRVRKNTDSKNN----L      1805
XP_014996065.1       --DQVQQASAS-SSATNKN-QLDGKKKKPTSPVKPIPQNTEYRTRIRKNADSKNN----L      1799
AAA03586.1           --DQVQQASAS-SSAPNKN-QLDGKKKKPTSPVKPIPQNTEYRTRVRKNADSKNN----L      1799
                        *  :                   :*  **:  : ..        :  :   .

XP_783363.3          DTVTTYCMEGTPGISNATSLSDLTSPIDSEDVNGNITSQNLPQNRSTLGVGVSRGSTPSD      1730
NP_001137312.1       ADPATYPDKNKETR-------KQEP-----KVVIRDFADKPSNAE-----ERTRPGFAFD      1806
NP_001084351.1       NSENQYCDP-R----------KPSS-----KKPSKVANEKIPNNE-----ERTKG-FAFD      1838
XP_004949340.1       NNERSYPEN-RDAK-------KQNL-----KNNSRDFNDKLPNNE-----ERVRGSFTFD      1844
XP_007497871.1       NVERSYS-D-KDSK-------KLSL-----KNNARDFLDKMPNNE-----DRVRGSFTFD      1839
NP_031488.2          NTEETFSDN-KDSK-------KPSL-----QTNAKAFNEKLPNNE-----DRVRGSFALD      1839
NP_001069454.2       NAERNFSEN-KDSK-------KQHL-----KNNSKDFNDKLPNNE-----DRVRGSFTFD      1847
XP_014996065.1       NAERVFSDN-KDSK-------KQNL-----KNNSKDFNDKLPNNE-----DRVRGSFAFD      1841
AAA03586.1           NAERVFSDN-KDSK-------KQNL-----KNNSKDFNDKLPNNE-----DRVRGSFAFD      1841
                          :               .        .   .   ::  : .        :     *

XP_783363.3          TPR-VFAVEGTPINFSCNGSLSSLSCDEDA-ELTEAKAQMKEVSKRNQAGGGMSRQQTVI      1788
NP_001137312.1       SPHHYTPIEGTPYCFSRNDSLSSLDFEDEDLDFSKEKAVLRKDKEQRKVPL------LK-      1859
NP_001084351.1       SPHHYTPIEGTPYCFSRNDSLSSLDFEDDDIDLSKEKAELRKEKGTKDTDQ------KV-      1891
XP_004949340.1       SPHHYTPIEGTPYCFSRNDSLSSLDFDDDDVDLSREKAELRKGKEAKEVET------KD-      1897
XP_007497871.1       SPHHYTPIEGTPYCFSRNDSLSSLDFDDDDVDLSREKAEL-KGKEAKETEA------KV-      1891
NP_031488.2          SPHHYTPIEGTPYCFSRNDSLSSLDFDDDDVDLSREKAELRKGKESKDSEA------KV-      1892
NP_001069454.2       SPHHYTPIEGTPYCFSRNDSLSSLDFDDDDVDLSREKAELRKGKENKESEA------KV-      1900
XP_014996065.1       SPHHYTPIEGTPYCFSRNDSLSSLDFDDDDVDLSREKAELRKAKENKESEA------KV-      1894
AAA03586.1           SPHHYTPIEGTPYCFSRNDSLSSLDFDDDDVDLSREKAELRKAKENKESEA------KV-      1894
                     :*:    :****  ** *.*****. :::  :::.  ** : : .  ..

XP_783363.3          PKQRASFSNPIEQVQSPENKYYSMDDYHNAEASKSAMSPLDSPRVFAVEGTPGIISRADS      1848
NP_001137312.1       -------CS-VEQ---PANTNM---------VSTFQT----------APTKP--L-----      1882
NP_001084351.1       -------KYKHEN---RAINPM---------GKQDQT----------GPKSL--GGRDQ-      1919
XP_004949340.1       -------CPNVEQ---PSGQQP---------SNRTQV----------CQKHP--TSRSQ-      1925
XP_007497871.1       -------SNHLEL---TSNQQS---------ANRAQI----------CAKHP--VERGQ-      1919
NP_031488.2          -------TCRPEP---NSSQQA---------ASKSQA----------SIKHP--ANRAQ-      1920
NP_001069454.2       -------TNHTEL---TSNQQS---------ASKTPA----------VTKQP--INRGQ-      1928
XP_014996065.1       -------TSHTEL---TSNQQS---------ASKTQA----------IAKHP--INRGQ-      1922
AAA03586.1           -------TSHTEL---TSNQQS---------ANKTQA----------IAKQP--INRGQ-      1922
                                *                    .

XP_783363.3          LSSLSCDEEDASPAVEKSAKERLAEKTRTSRISGSRMNHLGEGSMAMRRLSSEEAPLSYA      1908
NP_001137312.1       ----------------QK--------T-V--F------PQAPKEN---TVVVCDEKQKFS      1906
NP_001084351.1       ----------PKALVQKP--------T-S--F------SSAAKGTQDRGGATDEKMENFA      1952
XP_004949340.1       ----------SK----------------T--F------CQPSKDIPDRGAATDEKMQNFA      1951
XP_007497871.1       ----------SKPLLQKQ--------S-T--F------PQSSKDMPDRVAATDEKLQNFA      1952
NP_031488.2          ----------SKPVLQKQ--------P-T--F------PQSSKDGPDRGAATDEKLQNFA      1953
NP_001069454.2       ----------SKPVLQKQ--------S-T--F------PQSSKDIPDRGAATDEKLQNFA      1961
XP_014996065.1       ----------LKPILQKQ--------S-T--F------PQSSKDIPDRGAATDEKLHNFA      1955
AAA03586.1           ----------PKPILQKQ--------S-T--F------PQSSKDIPDRGAATDEKLQNFA      1955
                                                    :                     :   .::

XP_783363.3          VEDTPTCFSHNSSLSALSDNDEEERPDLTEQW-HDDEESQPQRSSGGANGINKDSRRVFA      1967
NP_001137312.1       IEDTPVCFSRNSSLSSLSDIDQENNNKDCSHK-----DDVTQ-MEAPRPQASGYAPKAFH      1960
NP_001084351.1       IENTPVCFSRNSSLSSLSDIDQENN-NKETEPLKQTGTSETQ-LGLRRPQTSGYAPKSFH      2010
XP_004949340.1       IENTPVCFSRNSSLSSLSDIDQENNNNKEGEPVKRTEAPDSQ-IESSRPQTSGYAPKSFH      2010
XP_007497871.1       IENTPVCFSRNSSLSSLSDIDQENNNNKESEPTKETEPPDSQ-GEPNRPQTSGYAPKSFH      2011
NP_031488.2          IENTPVCFSRNSSLSSLSDIDQENNNNKESEPIKEAEPANSQ-GEPSKPQASGYAPKSFH      2012
NP_001069454.2       IENTPVCFSRNSSLSSLSDIDQENNNNKENEPVKETEPPASQ-GEPGKPQASGYAPKSFH      2020
XP_014996065.1       IENTPVCFSHNSSLSSLSDIDQENNNNKENEPIKETEPPDSQ-GEPSKPQASGYAPKSFH      2014
AAA03586.1           IENTPVCFSHNSSLSSLSDIDQENN-NKENEPIKETEPPDSQ-GEPSKPQASGYAPKSFH      2013
                     :*:**.***:*****:*** *:*:. .   .          *         .  :  :  *

XP_783363.3          TEDTPVCFSRNSSLSSLDAESDGDAASEQALLDECITSGMPQSKVKKPVKRI--------      2019
NP_001137312.1       VEDTPVCFSRNSSLSSLSIDS------EDDLLQECISSAMPKKKKQTPRSKTEESGVKEE      2014
NP_001084351.1       VEDTPVCFSRNSSLSSLSIDS------EDDLLQECISSAMPKKRKPSKIKNE----VGKS      2060
XP_004949340.1       VEDTPVCFSRNSSLSSLSIDS------EDDLLQECISSAMPKKKKPSRIKSE----SEKS      2060
XP_007497871.1       VEDTPVCFSRNSSLSSLSIDS------EDDLLQECISSAMPKKKRPSRFKGD----DEKP      2061
NP_031488.2          VEDTPVCFSRNSSLSSLSIDS------EDDLLQECISSAMPKKKRPSRLKSE----SEKQ      2062
```

```
NP_001069454.2      VEDTPVCFSRNSSLSSLSIDS------EDDLLQECISSAMPKKKKPSRLKPD----NEKH      2070
XP_014996065.1      VEDTPVCFSRNSSLSSLSIDS------EDDLLQECISSAMPKKKKPSRLKGD----NEKH      2064
AAA03586.1          VEDTPVCFSRNSSLSSLSIDS------EDDLLQECISSAMPKKKKPSRLKGD----NEKH      2063
                    .****************. :*      *: **:***:*.**:.:  .  .

XP_783363.3         ----NGKIISGGPSSM--------------------------------------------      2031
NP_001137312.1      KSMMADGILSEEPDLILDLTDTHSPISEQALSPDSESFDWKAIQEGANSIVSSLHQAA--      2072
NP_001084351.1      RSNSVGGILAEEPDLTLDLRDIQSPDSENAFSPDSENFDWKAIQEGANSIVSRLHQAA-A      2119
XP_004949340.1      NSRNIGGMLAE--DLTLDLREIQRPDSEHGFSPDSENFDWKAIQEGANSIVSSLHQAA-A      2117
XP_007497871.1      SPRNMDGILAE--DLTLDLRDIQRPDSEHGFSPDSENFDWKAIQEGANSIVSSLHQAA-A      2118
NP_031488.2         SPRKVGGILAE--DLTLDLKDLQRPDSEHAFSPDSENFDWKAIQEGANSIVSSLHQAAAA      2120
NP_001069454.2      SPRNMGGILAE--DLTLDLKDIQRPDSEHGLSPDSENFDWKAIQEGANSIVSSLHQAA-A      2127
XP_014996065.1      SPRNMGGMLAE--DLTLDLKDIQRPDSEHGLSPDSENFDWKAIQEGANSIVSSLHQAA-A      2121
AAA03586.1          SPRNMGGILGE--DLTLDLKDIQRPDSEHGLSPDSENFDWKAIQEGANSIVSSLHQAA-A      2120
                         . ::.   .

XP_783363.3         ------------------------EDDDSSPANQENEPKPVRKGPRITKPSASVEEKSVN      2067
NP_001137312.1      -ASLSRQGSSDSDSILSLKSGISIGSPFHLPLNQDDKPA-PNKGPRILKPGEKSSIEAKK      2130
NP_001084351.1      AGSLSRQGSSDSDSILSLKSGISLGSPFHLTLDKEEKTITSNKGPKILKPAEKSALENKK      2179
XP_004949340.1      AASLSRQASSDSDSILSLKSGISLGSPFHLTPDQEEKPFTSNKGPRILKPGEKSTLESKK      2177
XP_007497871.1      AACLSRQASSDSDSILSLKSGISLGSPFHLTPDQEEKPFTSNKGPRILKPGEKSTLETKK      2178
NP_031488.2         AACLSRQASSDSDSILSLKSGISLGSPFHLTPDQEEKPFTSNKGPRILKPGEKSTLEAKK      2180
NP_001069454.2      AACLSRQASSDSDSILSLKSGISLGSPFHLTPDQEEKPFTSNKGPRILKPGEKSTLETKK      2187
XP_014996065.1      AACLSRQASSDSDSILSLKSGISLGSPFHLTPDQEEKPFTSNKGPRILKPGEKSTLETKK      2181
AAA03586.1          AACLSRQASSDSDSILSLKSGISLGSPFHLTPDQEEKPFTSNKGPRILKPGEKSTLETKK      2180
                                             .      :::::    .***:* **. .   :  :

XP_783363.3         EEEEGPKGVKGGKKIYRSPITGKIRSITPPKSVLPPKSPSSTRGGLAKGSPTTSRGRGAI      2127
NP_001137312.1      KEEETAKSLKGGKKVYKSLITGKPRPSLES--MAS--------QHRQAQAPVISRGRTMV      2180
NP_001084351.1      TEE-EPKGIKGGKKVYKSLITGKSRSSSDFSSHCK--------QSVQTNMPSISRGRTMI      2230
XP_004949340.1      VES-ESKGIKGGKRVYKSIITGKARSNSEVSSQIK--------QPQQTSVPSISRGRTMI      2228
XP_007497871.1      IES-ENKGIKGGKKVYKSLITGKVRSNSEVSGQLK--------QPLPTNMPSISRGRTMI      2229
NP_031488.2         IES-ENKGIKGGKKVYKSLITGKIRSNSEISSQMK--------QPLPTNMPSISRGRTMI      2231
NP_001069454.2      IES-ENKGIKGGKKVYKSLITGKVRSNSEISSQMK--------QPLQTNMPSISRGRTMI      2238
XP_014996065.1      IES-ESKGIKGGKKVYKSLITGKVRSNSEISGQMK--------QPLQANMPSISRGRTMI      2232
AAA03586.1          IES-ESKGIKGGKKVYKSLITGKVRSNSEISGQMK--------QPLQANMPSISRGRTMI      2231
                     *.   *.:****::*:* **** *                        *  ****   :

XP_783363.3         RGARGGFARSASTTPPRSSTPTGRGTPPRTTTPPRTTSPMTAGRTTTPPRTTSPRTTSPR      2187
NP_001137312.1      HVP--GVRSSSPSTSPV-----PKKPPPRG--------QMSKPPSQAPGAGSSPRTMKVP      2225
NP_001084351.1      HIP--GVRASSPSTSPV-----SKKGPVFKN-------VPSKGSNENPSSSSSPKGTKPL      2276
XP_004949340.1      HIP--GVRNSSSSTSPV-----SKKGPPFKN-------TNSKSPSEGQSSASSPRGVKSS      2274
XP_007497871.1      HIP--GIRNSSSSTSPV-----SKKGPSLKT-------PTSKSPSEGPTSSTSPRGAKPS      2275
NP_031488.2         HIP--GLRNSSSSTSPV-----SKKGPPLKT-------PASKSPSEGPGATTSPRGTKPA      2277
NP_001069454.2      HIP--GVRNSSSSTSPV-----SKKGPPLKT-------PASKSPSEGQPATTSPRGTKPS      2284
XP_014996065.1      HIP--GVRNSSSSTSPV-----SKKGPPLKT-------PASKSPSEGQTATTSPRGAKPS      2278
AAA03586.1          HIP--GVRNSSSSTSPV-----SKKGPPLKT-------PASKSPSEGQTATTSPRGAKPS      2277
                    :    *.  *: :* *       :  *             :   .     :**:   .

XP_783363.3         SGTPPKPKGSIA---NKSITPVRNATNGARSITPPRPVRKSSVDSQKDGDAKSETSSR--      2242
NP_001137312.1      PSSEPSPASG--PPSSQGGSSKASSRSGSRDSTPSRPVQQSLTRPMQSPGRASVSPGRNG      2283
NP_001084351.1      KS-ELVY--GSRPSSTPGGSSKGNSRSGSRDSASSRPSPQPLSRPLQSPGRNSISPGKNG      2333
XP_004949340.1      VKPEPAPVTRQLSGLNQGGSSKGPSRSGSRDSTPSRPQQQPLSRPLQSPGRNSISPGRNG      2334
XP_007497871.1      VKSELSPVTRQTS--QPGGSSKGPSRSGSRDSTPSRPSQQPLSRPMQSPGRNSISPGRNG      2333
NP_031488.2         GKSELSPITRQTS--QISGSNKGSSRSGSRDSTPSRPTQQPLSRPMQSPGRNSISPGRNG      2335
NP_001069454.2      VKSELSPVTRQAS--QTAGSNKGPSRSGSRDSTPSRPAQQPLSRPMQSPGRNSISPGRNG      2342
XP_014996065.1      VKSELSPVARQTS--QIGGSSKAPSRSGSRDSTPSRPAQQPLSRPIQSPGRNSISPGRNG      2336
AAA03586.1          VKSELSPVARQTS--QIGGSSKAPSRSGSRDSTPSRPAQQPLSRPIQSPGRNSISPGRNG      2335
                                     . :    : .*:*. :  **  :      :. .  * : .:

XP_783363.3         RSSKESISSIP-KPRQIKPQSTQTKTSPNSSTSPKPPTGRGSPAGKPPLSTSRSSSPKSK      2301
NP_001137312.1      LSPSNKLSQLPQLPRTASPSASSTKSS-----------GS----GRMAY-----TSPGRQ      2323
NP_001084351.1      ISPPNKFSQ---LPRTTSPSTASTKSS-----------GS----GRMSY-----TSPGRQ      2370
XP_004949340.1      ISPPNKLSQ---LPRTSSPSTASTKSS-----------SS----GRMSY-----TSPGRQ      2371
XP_007497871.1      ISPPNKLSQ---LPRTSSPSTSSTKSS-----------GS----GKISY-----TSPGRQ      2370
NP_031488.2         ISPPNKLSQ---LPRTSSPSTASTKSS-----------GS----GKMSY-----TSPGRQ      2372
NP_001069454.2      ISPPNKLSQ---LPRTSSPSTASTKSS-----------GS----GKMSY-----TSPGRQ      2379
XP_014996065.1      ISPPNKLSQ---LPRTSSPSTASTKSS-----------GS----GKMSY-----TSPGRQ      2373
AAA03586.1          ISPPNKLSQ---LPRTSSPSTASTKSS-----------GS----GKMSY-----TSPGRQ      2372
                     *  :.:*.    **  .*.::.**:*           .     *:        :**  :
XP_783363.3         MPVPSRSNSPSTKSSGSVTTPRKPLNKSPSSTKARDES-PTDAEEVTSQMKSMHLESNGS      2360
NP_001137312.1      LVQPTP-----TKQSGLPR-STSGIPRSESASKILNQC----GPSKKAELSRM-SSTKSS      2372
NP_001084351.1      LSQPNL-----SKQSGLPK-THSSIPRSESASKSLNQNVNT-GSNKKVELSRM-SSTKSS      2422
```

```
XP_004949340.1      MSQQNL-----TKQTALTK-NTSSIPRSESASKGLNQILGSGASNKKTDLSRM-SSAKSS      2424
XP_007497871.1      MSQQNL-----TKQTGLSK-NTSNIPRSESASKGLNQISNSNGTNKKVELSRM-SSTKSS      2423
NP_031488.2         LSQQNL-----TKQASLSK-NASSIPRSESASKGLNQMSNGNGSNKKVELSRM-SSTKSS      2425
NP_001069454.2      MSQQNL-----TKQTGLSK-NGSGIPRSESASKGLNQMSNSNSNGSNKKVELSRM-SSTKSS   2432
XP_014996065.1      MSQQNL-----TKQTGLSK-NASSIPRSESASKGLNQVNNGNGANKKVELSRM-SSTKSS      2426
AAA03586.1          MSQQNL-----TKQTGLSK-NASSIPRSESASKGLNQMNNGNGANKKVELSRM-SSTKSS      2425
                    :   .      :*.:.       . : :* *::*  ::     . . . ::. *  .::.*

XP_783363.3         SPDLDDGDRPVLLKQSTFTKDSASLPEQTP-----EMNPVSGD--------EKVLSPVQS      2407
NP_001137312.1      GSESDRSEKPGLVRQSTFIKEAPSPTLKRKLEESASFESLSPSST------SQSQTPVSS      2426
NP_001084351.1      GSESDRSERPALVRQSTFIKEAPSPTLRRKLEESASFETLSSSSRADSPPRSQTQTPALS      2482
XP_004949340.1      GSESDRSERPVLVRQSTFIKEAPSPTLRRKLEESASFESLSP-SRPDSPTRSQLQTPVLS      2483
XP_007497871.1      GSESDRSERPVLVRQSTFIKETPSPTLRRKLEESASLESLSSSSRPDSPTKSQVQTPILS      2483
NP_031488.2         GSESDRSERPALVRQSTFIKEAPSPTLRRKLEESASFESLSPSSRPDSPTRSQAQTPVLS      2485
NP_001069454.2      GSESDRSERPVLVRQSTFIKEAPSPTLRRKLEESASFESLSPSSRPDSPTRSQAHTPVLS      2492
XP_014996065.1      GSESDRSERPVLVRQSTFIKEAPSPTLRRKLEESASFESLSPSSRPASPTRSQAQTPVLS      2486
AAA03586.1          GSESDRSERPVLVRQSTFIKEAPSPTLRRKLEESASFESLSPSSRPASPTRSQAQTPVLS      2485
                     . : *  .::* *::**** *:: *    :       .:: :*           .:  :*  *

XP_783363.3         EEAPSVTESESSVSLSKGGWKKTGSGIQGSQESKKSSTGN-KPVSK--TTGVSKITPRRT      2464
NP_001137312.1      PSLPDMSL-SLP--YQGSGWTKAPQSQN-SAENGDGKSLKRHDISRSHSESPSRLPINRT      2482
NP_001084351.1      PSLPDMAL-STHS-IQAGGWRKMPPNLNPAAEH--GDSRRRHDISRSHSESPSRLPITRS      2538
XP_004949340.1      PSLPDMSL-STHSTAQTSGWRKLPPNLSPSVEY-DGRPAKRHDIARSHSESPSRLPINRS      2541
XP_007497871.1      PSLPDMSL-STHSSIQTGSWRKLPPNLNPSIEFNDGRSTKRHDIARSHSESPSRLPVNRS      2542
NP_031488.2         PSLPDMSL-STHPSVQAGGWRKLPPNLSPTIEYNDGRPTKRHDIARSHSESPSRLPINRA      2544
NP_001069454.2      PSLPDMSL-STHSSLQSGGWRKLPPNLSPTIEYNDGRPVKRHDIARSHSESPSRLPINRS      2551
XP_014996065.1      PSLPDMSL-STHSSVQAGGWRKLPPNLSPTIEYNDGRPAKRHDIARSHSESPSRLPINRS      2545
AAA03586.1          PSLPDMSL-STHSSVQAGGWRKLPPNLSPTIEYNDGRPAKRHDIARSHSESPSRLPINRS      2544
                     . *.::  .      . ..* *    . . : *    .    . : :::  : . *::   *:

XP_783363.3         GSPGLRTPAARSSSPGQRVNTPARRSESPSRMSSTSQRSESPSRASTTSQSSVSKQRTTP      2524
NP_001137312.1      GTWK--REHSKHSSSLPRVGTWKRTGSSSSILSASSESSEKG---RS----EDERQP-TN      2532
NP_001084351.1      GTWK--REHSKHSSSLPRVSTWRRTGSSSSILSASSESSEKA---KS----EDEKQQ-VC      2588
XP_004949340.1      GTWK--REHSKHSSSLPRVSTWRRTGSSSSILSASSESSEKA---KS----EDEKQH-GS      2591
XP_007497871.1      GTWK--REHSKHSSSLPRVSTWRRTGSSSSILSASSESSEKA---KS----EDEKHV--S      2591
NP_031488.2         GTWK--REHSKHSSSLPRVSTWRRTGSSSSILSASSESSEKA---KS----EDERHV--S      2593
NP_001069454.2      GTWK--REHSKHSSSLPRVSTWRRTGSSSSILSASSESSEKA---KS----EDEKQV--N      2600
XP_014996065.1      GTWK--REHSKHSSSLPRVSTWRRTGSSSSILSASSESSEKA---KS----EDEKHV--N      2594
AAA03586.1          GTWK--REHSKHSSSLPRVSTWRRTGSSSSILSASSESSEKA---KS----EDEKHV--N      2593
                    *:        :: **   **.*  * ..* * :*::*: **.      :    . .::

XP_783363.3         QTKGPASTQRNNSATSKINTGLQKSGGMSKRSASPSGVKTNPNVNGTHASTPPKSTASRP      2584
NP_001137312.1      --PPQKSGKEG---------GLERKGTWRKAK--GSETSYAPM-----------------      2562
NP_001084351.1      SFPGPRS--EC---------SSSAKGTWRKIK--ESEILETPSNGSSST-----------      2624
XP_004949340.1      SLSGQKQSKES---------QAPAKGTWRKIK--ENEIPQIMNDPQ-HP-----------      2628
XP_007497871.1      SLLGTKQTKEN---------QGPAKGTWRKIK--ESEMTPISNVSQ-VT-----------      2628
NP_031488.2         SMPAPRQMKEN---------QVPTKGTWRKIK--ESDISPTGMASQ-SA-----------      2630
NP_001069454.2      SISGSKQTKEN---------QVSTKGTWRKIK--ESEISPTNSTSQ-TT-----------      2637
XP_014996065.1      SISGTKQSKEN---------QVSAKGTWRKIK--ENEISPTNSTSQ-TV-----------      2631
AAA03586.1          SISGTKQSKEN---------QVSAKGTWRKIK--ENEFSPTNSTSQ-TV-----------      2630
                          .  .              .*    * .   .

XP_783363.3         NTRPTKSGNAGVRTGPPTTRVASASPKPSGASARAVRNKPASRPSSAEGSRSLSGNNSSS      2644
NP_001137312.1      -------------SLDLQDQTGDAMSKSEDVWV-RIEDCPIN--------------NPRS      2594
NP_001084351.1      ----IAESNCSLESKTLVYQMAPAVSKTEDVWV-RIEDCPIN--------------NPRS      2665
XP_004949340.1      ----SSSATSSSDSKTLIYQMAPAVSKTEDVWV-RIEDCPIN--------------NPRS      2669
XP_007497871.1      ----SSGTTNGADSKTLIYQMAPAVSKTEDVWV-RIEDCPIN--------------NPRS      2669
NP_031488.2         ----SSGAASGAESKPLIYQMAPPVSKTEDVWV-RIEDCPIN--------------NPRS      2671
NP_001069454.2      ----SSGAANGAESKTLIYQMAPAVSKTEDVWV-RIEDCPIN--------------NPRS      2678
XP_014996065.1      ----SSGATNGAESKTLIYQMAPAVSKTEDVWV-RIEDCPIN--------------NPRS      2672
AAA03586.1          ----SSGATNGAESKTLIYQMAPAVSKTEDVWV-RIEDCPIN--------------NPRS      2671
                                 :     : .     * ... .  :.: * .              *  *

XP_783363.3         KETPSPQTPSKFGTFTKK-----------------------------------KAQSSVET      2670
NP_001137312.1      SKSPTASTPPVIDSLPIKGLACDRDSSESHSKLMSENA---AMSHLGSETNLNLLRSSES      2651
NP_001084351.1      GRSPTGNSPPVIDNVLDQGQKEE-AAKDCHTRHNSGNGN-----VPLLENRQKSFIKVDG      2719
XP_004949340.1      GRSPTGNTPPVIDSVSEKGVVNGKDSKEIQEKQNPGNGS-VPVRTIGLENRLNSFFQMDS      2728
XP_007497871.1      GRSPTGNTPPVIDNVAEKVSSGNKESKDNQGKPNGGNGSSAPARTVGLENRLNSFIQIDS      2729
NP_031488.2         GRSPTGNTPPVIDSVSEKGSSSIKDSKDTHGKQSVGSG--SPVQTVGLETRLNSFVQVEA      2729
NP_001069454.2      GRSPTGNTPPVIDTVSEKGNPNPKDSKDNQGKQNVSNGS-APTRTMGLENRLNSFIQVDP      2737
XP_014996065.1      GRSPTGNTPPVIDSVSEKGNPN-KDSKDNQAKQNVGNGS-VPMRTVGLENRLNSFIQVDA      2730
AAA03586.1          GRSPTGNTPPVIDSVSEKANPNIKDSKDNQAKQNVGNGS-VPMRTVGLENRLNSFIQVDA      2730
                     .:*: .:*  :...  :                                    :   . :
```

```
XP_783363.3         YDKKETERNSGESDSVS------------KSDLSSD-----DQKSSQLKAK---------     2704
NP_001137312.1      LDKKVTDIKPAPS-NPNIGPELHEFPVSERTPFSSTNSSKHSSPSGAVAARVSPFNYTPS     2710
NP_001084351.1      LDTKGTDPKSLINN----QQETNENTVAERTAFSSSSSSKHSSPSGTVAARVTPFNYNPS     2775
XP_004949340.1      PDKKGNETKPLQT-NPVPAPENNESTVSERTPFSSSSSSKHNSPIGAVAARVTPFNYNPS     2787
XP_007497871.1      PDKKAAETKSGQV-NLVPAPETSETSVAERTPFSSTSSSKHSSPSGTVAARVTPFNYNPS     2788
NP_031488.2         PEQKGTEAKPGQS-NPVSIAETAETCIAERTPFSSSSSSKHSSPSGTVAARVTPFNYNPS     2788
NP_001069454.2      PDQKGTETKPGHSNNPVPASETSESSIAERTPFSSSSSSKHSSPSGTVAARVSPFNYNPS     2797
XP_014996065.1      PDQKGTETKPGQ-NNPVPVSETNESSIVERTPFSSSSSSKHSSPSGTVAARVTPFNYNPS     2789
AAA03586.1          PDQKGTEIKPGQ-NNPVPVSETNESSIVERTPFSSSSSSKHSSPSGTVAARVTPFNYNPS     2789
                     : *  : :                    :: :**      ..  . : *:

XP_783363.3         ----------DLCKELNIPVKNCGGKIVSPTRETAEGDGIWMKRPEDEVKDCASVSIHSS     2754
NP_001137312.1      PRKSSADGSTPRPSQIPTPISSNAKK------------------RDTKGDTT--------     2744
NP_001084351.1      PRKSNGENSTSRPSQIPTPVTNSTKK------------------RDSKTETTDSSGSQSP     2817
XP_004949340.1      RRKSSVDNSSARPSQIPTPVNNSTKK------------------RDSKSENTDSSGTQSP     2829
XP_007497871.1      PRKSSADSSAARPSQIPTPVNNSTKK------------------RDSKTENTESSGTQSP     2830
NP_031488.2         PRKSSADSTSARPSQIPTPVSTNTKK------------------RDSKTDSTESSGAQSP     2830
NP_001069454.2      PRKSSTDGTSARPSQIPTPVSNNTKK------------------RDSKPDSTEPSGTQSP     2839
XP_014996065.1      PRKSSADSTSARPSQIPTPVNNNTKK------------------RDSKTDSTESSGTQSP     2831
AAA03586.1          PRKSSADSTSARPSQIPTPVNNNTKK------------------RDSKTDSTESSGTQSP     2831
                                 .::  *:..   *                   : : .

XP_783363.3         SRHSSSYSLSSISTNHQVGSLSRQKSAQPELPSSKKSPAVNTRRNIFSSLNKNSNSKSSS     2814
NP_001137312.1      --ESGSYIVTSV------------------------------------------------     2754
NP_001084351.1      KRHSGSYLVTSV------------------------------------------------     2829
XP_004949340.1      KRHSGSYLVTSV------------------------------------------------     2841
XP_007497871.1      KRHSGSYLVTSV------------------------------------------------     2842
NP_031488.2         KRHSGSYLVTSV------------------------------------------------     2842
NP_001069454.2      KRHSGSYLVTSV------------------------------------------------     2851
XP_014996065.1      KRHSGSYLVTSV------------------------------------------------     2843
AAA03586.1          KRHSGSYLVTSV------------------------------------------------     2843
                      .*.** ::*:

XP_783363.3         KSSLDSSKSGGKSTPKMTSKQKHESEAKPEEKKDKKSEKKRFSFLKLKIGGKKSDSDSEG     2874
NP_001137312.1      ------------------------------------------------------------     2754
NP_001084351.1      ------------------------------------------------------------     2829
XP_004949340.1      ------------------------------------------------------------     2841
XP_007497871.1      ------------------------------------------------------------     2842
NP_031488.2         ------------------------------------------------------------     2842
NP_001069454.2      ------------------------------------------------------------     2851
XP_014996065.1      ------------------------------------------------------------     2843
AAA03586.1          ------------------------------------------------------------     2843

XP_783363.3         KGKSKGFFSKKEKKSPKNLKVQTKSRSESDSMAEHVLPSPPLAELEPDLNMSGADLNFDR     2934
NP_001137312.1      ------------------------------------------------------------     2754
NP_001084351.1      ------------------------------------------------------------     2829
XP_004949340.1      ------------------------------------------------------------     2841
XP_007497871.1      ------------------------------------------------------------     2842
NP_031488.2         ------------------------------------------------------------     2842
NP_001069454.2      ------------------------------------------------------------     2851
XP_014996065.1      ------------------------------------------------------------     2843
AAA03586.1          ------------------------------------------------------------     2843

XP_783363.3         HLDELEAWNSDNDIDNEVMPDEFVEWDSGSDIFKEEMPEEFIESCSEVDTDPATPLSPGV     2994
NP_001137312.1      ------------------------------------------------------------     2754
NP_001084351.1      ------------------------------------------------------------     2829
XP_004949340.1      ------------------------------------------------------------     2841
XP_007497871.1      ------------------------------------------------------------     2842
NP_031488.2         ------------------------------------------------------------     2842
NP_001069454.2      ------------------------------------------------------------     2851
XP_014996065.1      ------------------------------------------------------------     2843
AAA03586.1          ------------------------------------------------------------     2843

XP_783363.3         TTGPGGGNIQIDPNGQRGGFSPHSETGSLSPNGLSGDCMSPVDEGDLSMEAGEQYFYSEN     3054
NP_001137312.1      ------------------------------------------------------------     2754
NP_001084351.1      ------------------------------------------------------------     2829
XP_004949340.1      ------------------------------------------------------------     2841
XP_007497871.1      ------------------------------------------------------------     2842
NP_031488.2         ------------------------------------------------------------     2842
NP_001069454.2      ------------------------------------------------------------     2851
XP_014996065.1      ------------------------------------------------------------     2843
AAA03586.1          ------------------------------------------------------------     2843
```

```
XP_783363.3         RLNSFIRLDDTKGETSDTFGEGELSISRNVQNQRSRSLSDTAVEQSPHRPQRLKLPQKPF      3114
NP_001137312.1      ------------------------------------------------------------      2754
NP_001084351.1      ------------------------------------------------------------      2829
XP_004949340.1      ------------------------------------------------------------      2841
XP_007497871.1      ------------------------------------------------------------      2842
NP_031488.2         ------------------------------------------------------------      2842
NP_001069454.2      ------------------------------------------------------------      2851
XP_014996065.1      ------------------------------------------------------------      2843
AAA03586.1          ------------------------------------------------------------      2843

XP_783363.3         GFHQNDASPSSNNVVPVLVSPYNYSPNPNRKGEIDTNYSGMGFHKDDSSRSSSEVVSPTG      3174
NP_001137312.1      ------------------------------------------------------------      2754
NP_001084351.1      ------------------------------------------------------------      2829
XP_004949340.1      ------------------------------------------------------------      2841
XP_007497871.1      ------------------------------------------------------------      2842
NP_031488.2         ------------------------------------------------------------      2842
NP_001069454.2      ------------------------------------------------------------      2851
XP_014996065.1      ------------------------------------------------------------      2843
AAA03586.1          ------------------------------------------------------------      2843

XP_783363.3         STRVTTV  3181
NP_001137312.1      -------  2754
NP_001084351.1      -------  2829
XP_004949340.1      -------  2841
XP_007497871.1      -------  2842
NP_031488.2         -------  2842
NP_001069454.2      -------  2851
XP_014996065.1      -------  2843
AAA03586.1          -------  2843
```
