# Supplementary material for: Curated multiple sequence alignment for the Adenomatous Polyposis Coli (APC) gene and accuracy of in silico pathogenicity predictions
Source: PLoS One. 2020 Aug 4;15(8):e0233673. doi: 10.1371/journal.pone.0233673 (PMC7402488; doi:10.1371/journal.pone.0233673)
Supplement: S4 Fig — PMSA was generated from the program Clustal Omega. Annotation as per S1 Fig. (PDF) [file pone.0233673.s004.pdf]

APC Alignment - 9 species with edits (2018)

Exon
Dimerization
Armadillo Repeat
Beta Catenin and SAMP Repeats (one)(two three)
PHA03307
MAP65_ASE1 Microtubule Association Family (basic region for microtubule binding)
EB1 Binding Site
DLG (Axin) Binding Site
Basic Region

CLUSTAL O(1.2.4) multiple sequence alignment

NP_001137312.1 – *Danio rerio*
NP_001084351.1 – *Xenopus laevis*
XP_004949340.1 – *Gallus gallus*
XP_007497871.1 – *Monodelphis domestica*
NP_031488.2 – *Mus musculus*
NP_001069454.2 – *Bos taurus*
XP_014996065.1 – *Macaca mulatta*
AAA03586.1 – *Homo sapiens*

```
NP_001137312.1      MAAASYDQLLKQVEALKMENSNLRQELEDNSNHLNKLETEASNMKEVLKQLQGSIEDEDSK      60
NP_001084351.1      MAAASYDQLVKQVEALTMENTNLRQELEDNSNHLTKLETEATNMKEVLKQLQGSIEDEAM      60
XP_004949340.1      MAAASYDQLLKQVEALKMENSNLRQELEDNSNHLTKLETEASNMKEVLKQLQGSIEDEAI      60
XP_007497871.1      MAAASYDQLLKQVEALKMENSNLRQELEDNSNHLTKLETEASNMKEVLKQLQGSIEDEAM      60
NP_031488.2         MAAASYDQLLKQVEALKMENSNLRQELEDNSNHLTKLETEASNMKEVLKQLQGSIEDETM      60
NP_001069454.2      MAAASYDQLLKQVEALKMENSNLRQELEDNSNHLTKLETEASNMKEVLKQLQGSIEDEAM      60
XP_014996065.1      MAAASYDQLLKQVEALKMENSNLRQELEDNSNHLTKLETEASNMKEVLKQLQGSIEDEAM      60
AAA03586.1          MAAASYDQLLKQVEALKMENSNLRQELEDNSNHLTKLETEASNMKEVLKQLQGSIEDEAM      60
                    *********:******.***:*************.******:*************::::

NP_001137312.1      DSQGQIEFLERIKEMSLDPSGFSGVKLRSKASLQGSA---------DSSPSPSPVSSCPR      111
NP_001084351.1      ASSGPIDLLERFKDLNLDSSNIPAGKARPKMSMRSYGSREGSLSGHSGECSPVPVGSFQR      120
XP_004949340.1      ASSGQIDLLERLKELNLESTSFPGVKLRQKVSVRSYGSREGSVSSRSGECSPVPMGSFPR      120
XP_007497871.1      ASSGQIDLLERLKELNLDSSNFPGVKLRPKMSIRSYGSREGSVSSRSGECSPVPMGSFSR      120
NP_031488.2         T-SGQIDLLERLKEFNLD-SNFPGVKLRSKMSLRSYGSREGSVSSRSGECSPVPMGSFPR      118
NP_001069454.2      ASSGQIDLLERLKELNLDSSNFPGVKLRSKMSLRSYGSREGSVSSRSGECSPVPMGSFPR      120
XP_014996065.1      ASSGQIDLLERLKELNLDSSNFPGVKLRSKMSLRSYGSREGSVSSRSGECSPVPMGSFPR      120
AAA03586.1          ASSGQIDLLERLKELNLDSSNFPGVKLRSKMSLRSYGSREGSVSSRSGECSPVPMGSFPR      120
                      .* *::***:*::.*: :.: . * * * *::. .         ... ** *:.*  *

NP_001137312.1      RGASSGGRDSAGYLEELEKERSLLVAELEKEEKEKDWYYAQLQNLTKRIDSLPLTENFSL      171
NP_001084351.1      RGLLNGSRESAGYMEELEKERLLLIAEHEKEEKEKRWYYAQLQNLTKRIDSLPLTENFSM      180
XP_004949340.1      RGFMNGSRESTGYLEELEKERSLLLAELEKEEKEKDWYYAQLQNLTKRIDSLPLTENFSL      180
XP_007497871.1      RGFMNGSRESTGYLEELEKERSLLLAELEKEEKEKDWYYAQLQNLTKRIDSLPLTENFSL      180
NP_031488.2         RTFVNGSRESTGYLEELEKERSLLLADLDKEEKEKDWYYAQLQNLTKRIDSLPLTENFSL      178
NP_001069454.2      RGFVNGSRENTGYLEELEKERSLLLADLDKEEKEKDWYYAQLQNLTKRIDSLPLTENFSL      180
XP_014996065.1      RGFVNGSRESTGYLEELEKERSLLLADLDKEEKEKDWYYAQLQNLTKRIDSLPLTENFSL      180
AAA03586.1          RGFVNGSRESTGYLEELEKERSLLLADLDKEEKEKDWYYAQLQNLTKRIDSLPLTENFSL      180
                    *   .*.*:.:**:******* **:*: :******  **********************:

NP_001137312.1      QTDMTRRQLEYEARQIRAAMEDQLGTCQDMEKRAQGRVARIQQIEKDMLRIRTRLQAQSA      231
NP_001084351.1      QTDMTRRQLEYEARQIRAAMEEQLGTCQDMEKRVQTRVGKIHQIEEEILRIRQLLQSQVA      240
XP_004949340.1      QTDMTRRQLEYEARQIRAAMEEQLGTCQDMEKRAQVRVARIQQIEKDILRIRQLLQSQAA      240
XP_007497871.1      QTDMTRRQLEYEARQIRAAMEEQLGTCQDMEKRAQLRVARIQQIEKDILRIRQLLQSQPA      240
NP_031488.2         QTDMTRRQLEYEARQIRAAMEEQLGTCQDMEKRAQRRIARIQQIEKDILRVRQLLQSQAA      238
NP_001069454.2      QTDMTRRQLEYEARQIRVAMEEQLGTCQDMEKRAQRRITRIQQIEKDILRIRQLLQSQAT      240
XP_014996065.1      QTDMTRRQLEYEARQIRVAMEEQLGTCQDMEKRAQRRIARIQQIEKDILRIRQLLQSQAT      240
AAA03586.1          QTDMTRRQLEYEARQIRVAMEEQLGTCQDMEKRAQRRIARIQQIEKDILRIRQLLQSQAT      240
                    *****************.***:***********.* *: :*:***:::**:*  **:* :

NP_001137312.1      ESESSGKR-YRERVKHEPLSQTEGSHAAGDAGAAAAASVCSQGSASRVDHDSASEMSSAG      290
NP_001084351.1      EAAERTPQSKHDAGSRDAEKLPDGQGTSE--ITASGNVGSGQGSSSRADHDTTSVMSSNS      298
XP_004949340.1      E-AERAPQGKHDAASHDTERQSEGQGAPE--ISMS-TSNTGQGSAARMDHETASVMSSSN      296
XP_007497871.1      E-AERASQSKPDHASHEAERPNEGPGAAD--VSVA-PPAGSQGSVAQVDQETASGGSANG      296
NP_031488.2         E-AERSSQSRHDAASHEAGRQHEGHGVAE--SNTA-ASSSGQSPATRVDHETASVLSSSG      294
NP_001069454.2      E-AERSSQSKHEAGSHEAERQNEGQGVAE--INMA-TSGSGQGSTTRIDHETASVLSSSS      296
XP_014996065.1      E-AERSSQNKHETGSHDAERQNEGQGVAE--INMA-TSGNGQGSTTRMDHETASVLSSSS      296
AAA03586.1          E-AERSSQNKHETGSHDAERQNEGQGVGE--INMA-TSGNGQGSTTRMDHETASVLSSSS      296
                    *  .   :   :  .::     :*  .       :     .*.  :: *::::*  *: .
NP_001137312.1      SYSVPRRLTSHLGTKVEMVYSLLSMLGTHDKDDMSRTLLAMSSSQDSCIAMRQSGCLPLL      350
```

```
NP_001084351.1      TYSVPRRLTSHLGTKVEMVYSLLSMLGTHDKDDMSRTLLAMSSSQDSCIAMRQSGCLPLL     358
XP_004949340.1      NYSVPRRLTSHLGTKVEMVYSLLSMLGTHDKDDMSRTLLAMSSSQDSCIAMRQSGCLPLL     356
XP_007497871.1      AYSVPRRLTSHLGTKVEMVYSLLSMLGTHDKDDMSRTLLAMSSSQDSCISMRQSGCLPLL     356
NP_031488.2         THSAPRRLTSHLGTKVEMVYSLLSMLGTHDKDDMSRTLLAMSSSQDSCISMRQSGCLPLL     354
NP_001069454.2      THSAPRRLTSHLGTKVEMVYSLLSMLGTHDKDDMSRTLLAMSSSQDSCISMRQSGCLPLL     356
XP_014996065.1      THSAPRRLTSHLGTKVEMVYSLLSMLGTHDKDDMSRTLLAMSSSQDSCISMRQSGCLPLL     356
AAA03586.1          THSAPRRLTSHLGTKVEMVYSLLSMLGTHDKDDMSRTLLAMSSSQDSCISMRQSGCLPLL     356
                     :*.*********************************************:**********

NP_001137312.1      IQLLHGNDKDSVLLGNSRGSKEARARASAALHNIIHSQPDDKRGRREIRVLHLLEQIRAY     410
NP_001084351.1      IQLLHGNDKDSVLLGNSRGSKEARASGSAALDNIIHSQPDDKRGRREIRVLHLLEQIRAY     418
XP_004949340.1      IQLLHGNDKDSVLLGNSRGSKEARARASAALHNIIHSQPDDKRGRREIRVLHLLEQIRAY     416
XP_007497871.1      IQLLHGNDKDSVLLGNSRGSKEARARASAALHNIIHSQPDDKRGRREIRVLHLLEQIRAY     416
NP_031488.2         IQLLHGNDKDSVLLGNSRGSKEARARASAALHNIIHSQPDDKRGRREIRVLHLLEQIRAY     414
NP_001069454.2      IQLLHGNDKDSVLLGNSRGSKEARARASAALHNIIHSQPDDKRGRREIRVLHLLEQIRAY     416
XP_014996065.1      IQLLHGNDKDSVLLGNSRGSKEARARASAALHNIIHSQPDDKRGRREIRVLHLLEQIRAY     416
AAA03586.1          IQLLHGNDKDSVLLGNSRGSKEARARASAALHNIIHSQPDDKRGRREIRVLHLLEQIRAY     416
                    ************************* .****.****************************

NP_001137312.1      CETCWEWQESHERGVDQDKNPMPSPVEHQICPAVCVLMKLSFDEEHRHAMNELGGLQAIG     470
NP_001084351.1      CETCWEWQEAHEQGMDQDKNPMPAPVDHQICPAVCVLMKLSFDEEHRHAMNELGGLQAIA     478
XP_004949340.1      CETCWEWQEAHEQGMDQDKNPMPAPVDHQICPAVCVLMKLSFDEEHRHAMNELGGLQAIA     476
XP_007497871.1      CETCWQWQEAHEQGMDQDKNPMPAPVEHQICPAVCVLMKLSFDEEHRHAMNELGGLQAIA     476
NP_031488.2         CETCWEWQEAHEQGMDQDKNPMPAPVEHQICPAVCVLMKLSFDEEHRHAMNELGGLQAIA     474
NP_001069454.2      CETCWEWQEAHEQGMDQDKNPMPAPVEHQICPAVCVLMKLSFDEEHRHAMNELGGLQAIA     476
XP_014996065.1      CETCWEWQEAHEQGMDQDKNPMPAPVEHQICPAVCVLMKLSFDEEHRHAMNELGGLQAIA     476
AAA03586.1          CETCWEWQEAHEPGMDQDKNPMPAPVEHQICPAVCVLMKLSFDEEHRHAMNELGGLQAIA     476
                    *****:***:** *:********:**:********************************.

NP_001137312.1      ELLQVDCEIYGLTNDHYSVTLRRYAGMALTNLTFGDVANKATLCSMKGCMRAMVAQLKSE     530
NP_001084351.1      ELLQVDCEMYGLINDHYSVTLRRYAGMALTNLTFGDVANKATLCSMKSCMRALVAQLKSE     538
XP_004949340.1      ELLQVDCEMYGLTNDHYSVTLRRYAGMALTNLTFGDVANKATLCSMKGCMRALVAQLKSE     536
XP_007497871.1      ELLQVDCEMYGLTSDHYSVTLRRYAGMALTNLTFGDVANKATLCSMKGCMRALVAQLKSE     536
NP_031488.2         ELLQVDCEMYGLTNDHYSVTLRRYAGMALTNLTFGDVANKATLCSMKGCMRALVAQLKSE     534
NP_001069454.2      ELLQVDCEMYGLTNDHYSITLRRYAGMALTNLTFGDVANKATLCSMKGCMRALVAQLQSE     536
XP_014996065.1      ELLQVDCEMYGLTNDHYSITLRRYAGMALTNLTFGDVANKATLCSMKGCMRALVAQLKSE     536
AAA03586.1          ELLQVDCEMYGLTNDHYSITLRRYAGMALTNLTFGDVANKATLCSMKGCMRALVAQLKSE     536
                    ********:*** .****:****************************.****:****:**

NP_001137312.1      SEDLQQVIASVLRNLSWRADVNSKKILREVGSVRALMECALEVQKESTLKSVLSALWNLS     590
NP_001084351.1      SEDLQQVIASVLRNLSWRADVNSKKTLREVGSVKALMECALDVKKESTLKSVLSALWNLS     598
XP_004949340.1      SEDLQQVIASVLRNLSWRADVNSKKTLREVGSVKALMECALEVKKESTLKSVLSALWNLS     596
XP_007497871.1      SEDLEQVIASVLRNLSWRADVNSKKTLREVGSVKALMECALEVKKESTLKSVLSALWNLS     596
NP_031488.2         SEDLQQVIASVLRNLSWRADVNSKKTLREVGSVKALMECALEVKKESTLKSVLSALWNLS     594
NP_001069454.2      SEDLQQVIASVLRNLSWRADVNSKKTLREVGSVKALMECALEVKKESTLKSVLSALWNLS     596
XP_014996065.1      SEDLQQVIASVLRNLSWRADVNSKKTLREVGSVKALMECALEVKKESTLKSVLSALWNLS     596
AAA03586.1          SEDLQQVIASVLRNLSWRADVNSKKTLREVGSVKALMECALEVKKESTLKSVLSALWNLS     596
                    ****:******************** *******:*******:*:****************

NP_001137312.1      AHCTENKADICTVPGALAFLVSTLTYRSQTNTLAIIESGGGILRNVSSLIATNEEHRQIL     650
NP_001084351.1      AHCTENKADICSVDGALAFLVSTLTYRSQTNTLAIIESGGGILRNVSSLIATNEDHRQIL     658
XP_004949340.1      AHCTENKADICAVDGALAFLVGTLTYRSQTNTLAIIESGGGILRNVSSLIATNEDHRQIL     656
XP_007497871.1      AHCTENKADICAVDGALAFLVGTLTYRSQTNTLAIIESGGGILRNVSSLIATNEDHRQIL     656
NP_031488.2         AHCTENKADICAVDGALAFLVGTLTYRSQTNTLAIIESGGGILRNVSSLIATNEDHRQIL     654
NP_001069454.2      AHCTENKADICAVDGALAFLVGTLTYRSQTNTLAIIESGGGILRNVSSLIATNEDHRQIL     656
XP_014996065.1      AHCTENKADICAVDGALAFLVGTLTYRSQTNTLAIIESGGGILRNVSSLIATNEDHRQIL     656
AAA03586.1          AHCTENKADICAVDGALAFLVGTLTYRSQTNTLAIIESGGGILRNVSSLIATNEDHRQIL     656
                    ***********:* *******.********************************:*****

NP_001137312.1      RENSCLQTLLQHLKSHSLTIVSNACGTLWNLSARNAKDQEALWDMGAVSMLKNLIHSKHK     710
NP_001084351.1      RENNCLQTLLQHLKSHSLTIVSNACGTLWNLSARNAKDQEGLWDMGAVSMLKNLIHSKHK     718
XP_004949340.1      RENSCLQTLLQHLKSHSLTIVSNACGTLWNLSARNAKDQEALWDMGAVSMLKNLIHSKHK     716
XP_007497871.1      RENSCLQTLLQHLKSHSLTIVSNACGTLWNLSARNTKDQEALWDMGAVSMLKNLIHSKHK     716
NP_031488.2         RENNCLQTLLQHLKSHSLTIVSNACGTLWNLSARNPKDQEALWDMGAVSMLKNLIHSKHK     714
NP_001069454.2      RENNCLQTLLQHLKSHSLTIVSNACGTLWNLSARNPKDQEALWDMGAVSMLKNLIHSKHK     716
XP_014996065.1      RENNCLQTLLQHLKSHSLTIVSNACGTLWNLSARNPKDQEALWDMGAVSMLKNLIHSKHK     716
AAA03586.1          RENNCLQTLLQHLKSHSLTIVSNACGTLWNLSARNPKDQEALWDMGAVSMLKNLIHSKHK     716
                    ***.******************************* ****.*******************

NP_001137312.1      MIAMGSAAALRNLMANRPAKYKDANIMSPGSSLPSLHVRKQKALIEELDAQHLSETFDNI     770
NP_001084351.1      MIAMGSAAALRNLMANRPAKYKDANIMSPGSSVPSLHVRKQKALEAELDAQHLSETFDNI     778
XP_004949340.1      MIAMGSAAALRNLMANRPAKYKDTNIMSPGSSLPSLHVRKQKALEAELDAQHLSETFDNI     776
XP_007497871.1      MIAMGSAAALRNLMANRPAKYKDANIMSPGSSLPSLHVRKQKALEAELDAQHLSETFDNI     776
```

```
NP_031488.2          MIAMGSAAALRNLMANRPAKYKDANIMSPGSSLPSLHVRKQKALEAELDAQHLSETFDNI     774
NP_001069454.2       MIAMGSAAALRNLMANRPAKYKDANIMSPGSSLPSLHVRKQKALEAELDAQHLSETFDNI     776
XP_014996065.1       MIAMGSAAALRNLMANRPAKYKDANIMSPGSSLPSLHVRKQKALEAELDAQHLSETFDNI     776
AAA03586.1           MIAMGSAAALRNLMANRPAKYKDANIMSPGSSLPSLHVRKQKALEAELDAQHLSETFDNI     776
                     ***********************:********:***********  **************

NP_001137312.1       DNLSPKASHRVKPRHKHNVYGDY--------DAVCRSDGYNPNGVGVRSPYMNTPVLSSP     822
NP_001084351.1       DNLSPKTTHRNKQRHKQNLCSEYALDSSRHDDSICRSDNFSIGNLTVLSPYINTTVLPGS     838
XP_004949340.1       DNLSPKASHRNKQRHKQNIYGEYVLDSSRHDDGVCRTESFNTGNMTVLSPYLNSTVLPGS     836
XP_007497871.1       DNLSPKTSHRPKQRHKQSVYGEYALDASRHDDS--RPDAFSTGNLTVLSPYLNSTVLPGS     834
NP_031488.2          DNLSPKASHRSKQRHKQNLYGDYAFDANRHDDS--RSDNFNTGNMTVLSPYLNTTVLPSS     832
NP_001069454.2       DNLSPKASHRSKQRHKQNLYGDYVFDTNRHDDN--RSDNFNTGNMTVLSPYLNTTVLPSS     834
XP_014996065.1       DNLSPKASHRSKQRHKQSLYGDYVFDTNRHEDN--RSDNFNAGNMTVLSPYLNTTVLPSS     834
AAA03586.1           DNLSPKASHRSKQRHKQSLYGDYVFDTNRHDDN--RSDNFNTGNMTVLSPYLNTTVLPSS     834
                     ******::** * ***:.: .:*        *   * : :. ..: * ***:*: ** .

NP_001137312.1       SSRDNRGNAESVRAERDRSLDRERRGFLPD---------GEAAKRMMQIPTSAAQIAVVM     873
NP_001084351.1       S-S-PRPTMDGSRPEK----DRERTAGLGNYHSTTESSGNSSKRIGIQLSTT-AQISKVM     891
XP_004949340.1       A-SSSRGNIENCLSEKDRSLDRDRAVGLNAYHPATENSGNSSKRIGMQISTAAAQIAKVM     895
XP_007497871.1       S--SSRTSLESSRSEKDRSLDRERAVALSTFHPAADSPGNPSKRLGMQLSTTTAQIAKVM     892
NP_031488.2          S--SSRGSLDSSRSEKDRSLERERGIGLSAYHPTTENAGTSSK-RGLQITTTAAQIAKVM     889
NP_001069454.2       S--SSRGSLDSSRSEKDRSLERERGISLGNYHPATENPGTSSK-RGLQISTTAAQIAKVM     891
XP_014996065.1       S--SSRGSLDSSRSEKDRSLERERGIGLGNYHPATENPGTSSK-RGLQISTTAAQIAKVM     891
AAA03586.1           S--SSRGSLDSSRSEKDRSLERERGIGLGNYHPATENPGTSSK-RGLQISTTAAQIAKVM     891
                     :    * . :.   *:    :*:*   *             :    :*: *: ***: **

NP_001137312.1       EEVQNMHLGMDDRSAGSTPDPHSVQDD--MIRRQTAVHGHQNIYSYSKTDPSGRPCPMPK     931
NP_001084351.1       DEVSNIHLVQENRSSGSASEMHCMSDERNSQRKPSSNHPQSNPFTFTKAESSTRGCPVAF     951
XP_004949340.1       EEVTSMHIPQEDRSSGSTSEMHCLTEDRNTTRRAATAHTHSNTY-FPKSENSSRPCPVPY     954
XP_007497871.1       EEVSAIHAQ-EDQSSASTTDLHCVAEERSTLRRASAAHAHSNTYNFPKPDNSNRTCAMPY     951
NP_031488.2          EEVSAIHTSQDDRSSASTTEFHCVADDRSAARRSSASHTHSNTYNFTKSENSNRTCSMPY     949
NP_001069454.2       EEVSAIHTSQEDRSSGSTTELHCGTDERNALRRSSTTHTHANTYNFTKSENSNRTCPIPY     951
XP_014996065.1       EEVSAIHTSQEDRSSGSTTELHCVTDERNALRRSSAAHTHSNTYNFTKSENSNRTCSMPY     951
AAA03586.1           EEVSAIHTSQEDRSSGSTTELHCVTDERNALRRSSAAHTHSNTYNFTKSENSNRTCSMPY     951
                     :**  :*   :::*:.*: : *.  ::    *: :: * : * : : * : * * * :

NP_001137312.1       L--EY-RASNDSLNSVNSTDGYGKRGQMKPSVDSYSEDDEGKCCVYRKYPADLAHKIHNA     988
NP_001084351.1       MKMEYKMASNDSLNSVSSTEGYGKRGQVKPSVESYSEDDESKFFSYGQYPAGLAHKIQSA     1011
XP_004949340.1       TKMEYKRASNDSLNSVSSSDGYGKRGQMKPSIESYSEDDESKFCSYGQYPADLAHKIHSA     1014
XP_007497871.1       AKVEYKRSSNDSLNSVSSSDGYGKRGQMKPSIESYSEDDESKFCSYGQYPADLAHKIHSA     1011
NP_031488.2          AKVEYKRSSNDSLNSVTSSDGYGKRGQMKPSVESYSEDDESKFCSYGQYPADLAHKIHSA     1009
NP_001069454.2       AKVEYKRSSNDSLNSVSSSDGYGKRGQMKPSIESYSEDDESKFCSYGQYPADLAHKIHSA     1011
XP_014996065.1       AKLEYKRSSNDSLNSVSSSDGYGKRGQMKPSIESYSEDDESKFCSYGQYPADLAHKIHSA     1011
AAA03586.1           AKLEYKRSSNDSLNSVSSSDGYGKRGQMKPSIESYSEDDESKFCSYGQYPADLAHKIHSA     1011
                        **  :********.*::*******:***::*******.*   * :***.*****:.*

NP_001137312.1       NHMEDDNGDLDTPINYSLKYSDEQLNSGRQSPSQNERWARPK-LLDDEMKRPDQKPPRSQ     1047
NP_001084351.1       NHMDDNDTELDTPINYSLKYSDEQLNSGRQSPTQNERWSRPKHIIDSEMKQSEQRQPRTT     1071
XP_004949340.1       NHMDDNDGELDTPINYSLKYSDEQLNSGRQSPSQNERWARPKHIIDDEMKQNDQRQSRSQ     1074
XP_007497871.1       NHMDDNDEELDTPINYSLKYSDEQLNSGRQSPSQNDRWARPKHVIEDEIKQNEQRQARGQ     1071
NP_031488.2          NHMDDNDGELDTPINYSLKYSDEQLNSGRQSPSQNERWARPKHVIEDEIKQNEQRQARSQ     1069
NP_001069454.2       NHMDDNDGELDTPINYSLKYSDEQLNSGRQSPSQNERWARPKHILEDEIKPNEQRQSRSQ     1071
XP_014996065.1       NHMDDNDGELDTPINYSLKYSDEQLNSGRQSPSQNERWARPKHIIEDEIKQSEQRQSRSQ     1071
AAA03586.1           NHMDDNDGELDTPINYSLKYSDEQLNSGRQSPSQNERWARPKHIIEDEIKQSEQRQSRNQ     1071
                     ***:*:: :***********************:**:**:*** :::.*:*  :*:  *

NP_001137312.1       SPGYPMYTEGSSEGEDKPKKYQPRFVQQD-LPAFRSR----GSNEQISSGSSHGLNKKIS     1102
NP_001084351.1       KTTYSSYTEN---KEEKHKKFPPHFNQSENVPAYTRSRGANNQVDQSRVSSNLSNNSKAS     1128
XP_004949340.1       SATYPVYTES---GDDKHMKYQSPFGQQDCVPSFRSR-GS-NGSDQNRVGSTLGINQKVN     1129
XP_007497871.1       NTPFSAYSES---TDDKHMKFQSRFGQQECVSPYRSR-GA-SGSEQNRVSSGHGINQKVN     1126
NP_031488.2          NTSYPVYSEN---TDDKHLKFQPHFGQQECVSPYRSR-GT-SGSETNRMGSSHAINQNVN     1124
NP_001069454.2       STAYPVYPES---TDDKHLKFQPHFGQQECVSPYRSR-AA-NGSETNRVGSNHGISQNVN     1126
XP_014996065.1       STTYPVYTES---TDDKHLKFQPHFGQQECVSPYRSR-GA-NGSETNRVGSNHGINQNVS     1126
AAA03586.1           STTYPVYTES---TDDKHLKFQPHFGQQECVSPYRSR-GA-NGSETNRVGSNHGINQNVS     1126
                     .  :  * *.    ::*  *:   * *.: :  :       .  :    .*  . ..: .

NP_001137312.1       QTICSVDDYADDKPTNYSERYSEEEQLEEQ---TPSYSMK--YTEDHHVEQPIDYSLKYS     1157
NP_001084351.1       KPHCQVDDYDDDKTTNFSERYSEEEQQEDETERQNKYNIKAYASEEHHGEQPIDYSRKYS     1188
XP_004949340.1       QSLCQVDDYDDDKPTNYSERYSEEEQHEE-EDRPTNYSIK-YNEEEHQVDQPIDYSLKYS     1187
XP_007497871.1       QSLCHEDDYDEDKPTNYSERYSEEEQHEE-EDRPTNYSMK-YNEEEHHADQPIDYSLKYA     1184
NP_031488.2          QSLCQEDDYEDDKPTNYSERYSEEEQHEEEEERPTNYSIK-YNEEKHHVDQPIDYSLKYA     1183
NP_001069454.2       QSLCQEDDYEDDKPTNYSERYSEEGQHEE-EERPTNYSIK-YSEEKHHVDQPIDYSLKYT     1184
XP_014996065.1       QSLCQEDDYEDDKPTNYSERYSEEEQHEE-EERPTNYSIK-YNEEKHHVDQPIDYSLKYA     1184
```

```
AAA03586.1           QSLCQEDDYEDDKPTNYSERYSEEEQHEE-EERPTNYSIK-YNEEKRHVDQPIDYSLKYA      1184
                     :  *   *** :** **:******* * *:      .*.:*    *.:: :****** **:

NP_001137312.1       EA---PSKKGMFSHSKTSSAQSSAKEHLSQDSSSSVASLKNQGRQKQLHPSSAQSRSGPT      1214
NP_001084351.1       TDVPSSAQKPSFPYSNNSSKQKPKKEQVSSNS-NTPTPSPNSNRQNQLHPNSAQSRPGLN      1247
XP_004949340.1       TEVPPSSQKPSFTFSKTSSVQSTKTDHISSSSGNTSAPSAGSKRQNQLHPSSAQSRGGHA      1247
XP_007497871.1       ADITPSSQKPSFSFSKSSSVQSNKTGHISSGG-NTSTAPASTKRQNQLLPSSAQNRSGHT      1243
NP_031488.2          TDI-SSSQKPSFSFSKNSSAQSTKPEHLSPSSENTAVPPSNAKRQNQLRPSSAQRN-GQT      1241
NP_001069454.2       TDI-PSSQKPAFSFSKNSSGQSTKTEHISSSSENTSTTSSNAKRQNQLHPSSAQSRSGQT      1243
XP_014996065.1       TDI-PSSQKQSFSFSKSSSGQSTKTEHISSSSENTSTPSSNAKRQNQLHPSSAQSRSGQT      1243
AAA03586.1           TDI-PSSQKQSFSFSKSSSGQSSKTEHMSSSSENTSTPSSNAKRQNQLHPSSAQSRSGQP      1243
                           ::*   *  .*:.** *.    ::*  .. .: .    .   **:**  *.***  .  *

NP_001137312.1       RAVQKNPTCKAPTINQETLQTYCVEDTPICFSRGSSLSSLSSEEDEMESCKRNVNSASNY      1274
NP_001084351.1       R--PKQIPNKPPSINQETIQTYCVEDTPICFSRGSSLSSLSSAEDEIEGRERNSRGQESN      1305
XP_004949340.1       ---QKTASCKTPSINQETIQTYCVEDTPICFSRCSSLSSLSSAEDEI-GRDQSTRVTDTN      1303
XP_007497871.1       ---QKTASCKAPSINQETIQTYCVEDTPICFSRCSSLSSLSSAEDEI-GRDQTTRGSETT      1299
NP_031488.2          ---QKGTTCKVPSINQETIQTYCVEDTPICFSRCSSLSSLSSADDEI-GCDQTTQEADSA      1297
NP_001069454.2       PK-ATSSSCKVPSINQETIQTYCVEDTPICFSRCSSLSSLSSAEDEV-GCDQTTQEAESA      1301
XP_014996065.1       QK-A--ATCKVSSINQETIQTYCVEDTPICFSRCSSLSSLSSAEDEI-GCDQTTQEADSA      1299
AAA03586.1           QK-A--ATCKVSSINQETIQTYCVEDTPICFSRCSSLSSLSSAEDEI-GCNQTTQEADSA      1299
                              *   :*****:************** ******** :**:  .  .:.  .   ..

NP_001137312.1       PTLPISEKQSTNN-VAADQRTSESQSSVHYVRAKPPRHHL--GHGDGSRHHKTVEFSSGA      1331
NP_001084351.1       NTLQITEPKEISA-VSKDGAVNETRSSVHHTRTKNNRLQTSNISPSDSSRHKSVEFSSGA      1364
XP_004949340.1       ATLQISELKENSGALSAEAAVSEITSTSQHIRTKSSRLPTSSLSPSESSRHKAVEFSSGA      1363
XP_007497871.1       NTLQIAELKENSASVSTGDTGSEVPSTSQHIRTKANRLQTTSLSPSDSTRHKAVEFSSGA      1359
NP_031488.2          NTLQTAEVKENDVTRSAEDPATEVPAVSQNARAKPSRLQASGLSSESTRHNKAVEFSSGA      1357
NP_001069454.2       NTLQIAEIKDNSGPRSNEDSVSKVPAGSQHIRTKSSRLQASGLSSESARH-KAVEFSSGA      1360
XP_014996065.1       NTLQIAEIKDKIGTRSTEDPVSEVPAVSQHTRTKSSRLQGSSLSSESTRH-KAVEFSSGA      1358
AAA03586.1           NTLQIAEIKEKIGTRSAEDPVSEVPAVSQHPRTKSSRLQGSSLSSESARH-KAVEFSSGA      1358
                      **   :* :.      :      .:  :  :  *:*   *         .  :  :  *:*******

NP_001137312.1       KSPSKSGAQTPKSPPEHYVQETPLMFSRCTSVSSLDSFESHSIASSVQSEP-CSGMVSGI      1390
NP_001084351.1       KSPSKSGAQTPKSPPEHYVQETPLMFSRCTSGSSLDSFESHSIASSIASSVASEHMISGI      1424
XP_004949340.1       KSPSKSGAQTPKSPPEHYVQETPLMFSRCTSVSSLDSFESRSIASSVQSEP-CSGMVSGI      1422
XP_007497871.1       KSPAKSGAQTPKSPPEHYVQETPLMFSRCTSVSSLDSFESRSIASSVQSEP-CSGMVSGI      1418
NP_031488.2          KSPSKSGAQTPKSPPEHYVQETPLVFSRCTSVSSLDSFESRSIASSVQSEP-CSGMVSGI      1416
NP_001069454.2       KSPSKSGAQTPKSPPEHYVQETPLMFSRCTSVSSLDSFESRSIASSVQSEP-CSGMVSGI      1419
XP_014996065.1       KSPSKSGAQTPKSPPEHYVQETPLMFSRCTSVSSLDSFESRSIASSVQSEP-CSGIVSGI      1417
AAA03586.1           KSPSKSGAQTPKSPPEHYVQETPLMFSRCTSVSSLDSFESRSIASSVQSEP-CSGMVSGI      1417
                     ***:*******************:****** ********:*****: *.   .. ::***

NP_001137312.1       ISPSDLPDSPGQTMPPSRSKTPPPPPPPRSTS---VKQKV--TVPPHTEKHDLAPRHAVVS      1445
NP_001084351.1       ISPSDLPDSPGQTMPPSRSKTPP-----PPQTVQAKKDGSKPIVPDEERG--KVAKTAVH      1477
XP_004949340.1       ISPSDLPDSPGQTMPPSRSKTPP-----PAQGVQVKRDVTKGKVPTAEKREPGPRQAAVN      1477
XP_007497871.1       ISPSDLPDSPGQTMPPSRSKTPP-----PPQTGQTKREATKSKLPSAEKRESGPRQAAVN      1473
NP_031488.2          ISPSDLPDSPGQTMPPSRSKTPP----PPPQTVQAKREVPKSKVPAAEKRESGPKQTAVN      1472
NP_001069454.2       ISPSDLPDSPGQTMPPSRSKTPPPPPPPPPQTVQTKQEVPKNKAPSAEKRESGPKQAAVN      1479
XP_014996065.1       ISPSDLPDSPGQTMPPSRSKTPPP----PPQTAQTKREVPKNKTPTAEKRESGPKQAAVN      1473
AAA03586.1           ISPSDLPDSPGQTMPPSRSKTPPP----PPQTAQTKREVPKNKAPTAEKRESGPKQAAVN      1473
                     ***********************       .    .*:.      *   *:      ::.*

NP_001137312.1       AAVQKVQVLPDNDTLLHFATESTPDGFSCASSLSALSLDEPFIQKDVELKIMPPVHEDDH      1505
NP_001084351.1       SAIQRVQVLQEADTLLHFATESTPDGFSCASSLSALSLDEPYIQKDVQLKIMPPVLENDQ      1537
XP_004949340.1       AAVQRVQVLPDADTLLHFATESTPDGFSCSSSLSALSLDEPFIQKDVELRIMPPVHENEH      1537
XP_007497871.1       AAVQRVQVLPDADTLLHFATESTPDGFSCSSSLSALSLDEPFIQKDVELRLMPPVQENEH      1533
NP_031488.2          AAVQRVQVLPDVDTLLHFATESTPDGFSCSSSLSALSLDEPFIQKDVELRIMPPVQENDN      1532
NP_001069454.2       AAVQRVQVLPEADTLLHFATESTPDGFSCSSSLSALSLDEPFIQKDVELRIMPPVQENDN      1539
XP_014996065.1       AAVQRVQVLPDADTLLHFATESTPDGFSCSSSLSALSLDEPFIQKDVELRIMPPVQENDN      1533
AAA03586.1           AAVQRVQVLPDADTLLHFATESTPDGFSCSSSLSALSLDEPFIQKDVELRIMPPVQENDN      1533
                     :*:*:****  : *****************:***********:*****:*::****  *:::

NP_001137312.1       SIEAEPEMEDMHEPKVQEKSPATSEAAKDILDDSDDDDDTEILNACINSAMPTKSSRKPK      1565
NP_001084351.1       GNKAEPEKE--FIDNKAKKEDKRSEQEKDMLDDT--DDDIDILEECIISAMPRKPSRKNK      1593
XP_004949340.1       GNEAEPEQSDETKDNQENKAEKPSEAEKDILDDSD-DDDIEILEECIISAMPTKSSRKAK      1596
XP_007497871.1       GNETEPEQLEDVSEVKEKKAEKPTESEKDILDDSD-DDDIEILEECIISAMPTKSSRKAK      1592
NP_031488.2          GNETESEQPEESNENQDKEVEKP-DSEKDLLDDSD-DDDIEILEECIISAMPTKSSRKAK      1590
NP_001069454.2       GNETESEQPEESNENQEKEAEKPTDSEKDLLDESD-DDDIEILEECIISAMPTKSSRKAK      1598
XP_014996065.1       GNETESEQPKESNENQEKEAEKTIDSEKDLLDDSD-DDDIEILEECIISAMPTKSSRKAK      1592
AAA03586.1           GNETESEQPKESNENQEKEAEKTIDSEKDLLDDSD-DDDIEILEECIISAMPTKSSRKAK      1592
                     . ::* *          ::     :  **:**::  *** :**: ** **** * *** *
```

```
NP_001137312.1      KQ---STSRIPPPVACKPSQLPVYKLLPPQNRGQPQKHVALAHGEDMPRVYCVEGTPINF     1622
NP_001084351.1      KVPQPTPGKPPPPVARKPSQLPVYKLLSSQNRLQTQKHVNFTHSDDMPRVYCVEGTPINF     1653
XP_004949340.1      KPSQASAPKIPPPVARKPSQLPVYKLLPSQSRLQSQKHVSFTPGDDMPRVYCVEGTPINF     1656
XP_007497871.1      KPSQA-ASKIPPPVARKPSQLPVYKLLPSQNRLQAQKHVSFTPGDDVPRVYCVEGTPINF     1651
NP_031488.2         KLAQT-ASKLPPPVARKPSQLPVYKLLPAQNRLQAQKHVSFTPGDDVPRVYCVEGTPINF     1649
NP_001069454.2      KPAQT-TSKLPPPVARKPSQLPVYKLLPSQNRLQAQKHVSFTPGDDMPRVYCVEGTPINF     1657
XP_014996065.1      KPAQT-ASKLPPPVARKPSQLPVYKLLPSQNRLQPQKHVSFTPGDDMPRVYCVEGTPINF     1651
AAA03586.1          KPAQT-ASKLPPPVARKPSQLPVYKLLPSQNRLQPQKHVSFTPGDDMPRVYCVEGTPINF     1651
                    *       : ***** ***********  *.* * **** :: .:*:*************

NP_001137312.1      STATSLSDLTIDSPPNELAGMESSAPHVEA-SGQRRDTLPE-GKSAEAKETGLSPPM-QS     1679
NP_001084351.1      STATSLSDLTIESPPSEPTND-QPNTDSLSTDLEKRDTIPTEGRSTDDTDASKPLNP-TT     1711
XP_004949340.1      STATSLSDLTIESPPNELANVDSVGAGAESGEFEKRDTIPTEGRSTDDTQRAKSITVTGP     1716
XP_007497871.1      STATSLSDLTIESPPNELAGVEGTSTGALLGDFEKRDTIPTEGRSTDDIQIGKSSNVNTS     1711
NP_031488.2         STATSLSDLTIESPPNELATGDGVRAGIQSGEFEKRDTIPTEGRSTDDAQRGKISSIVTP     1709
NP_001069454.2      STATSLSDLTIESPPNELAAGEGVRAGAQSSEFEKRDTIPTEGRSTDEAQRGKASSVTVP     1717
XP_014996065.1      STATSLSDLTIESPPNELAAGEGVRAGAQSGEFEKRDTIPTEGRSTDEAQGGKTSSVTIP     1711
AAA03586.1          STATSLSDLTIESPPNELAAGEGVRGGAQSGEFEKRDTIPTEGRSTDEAQGGKTSSVTIP     1711
                    ***********:***.* :            . ::***:*  *:*::  : .

NP_001137312.1      A--LAENEGDDILAECINSAMPKSKIHKPFRVQKMPDQAQHPSTATGS---LVQQDLEKK     1734
NP_001084351.1      VLDEDKAEEGDILAECIHSAMPKGKSHKPYRVKKIMDQINHTSAATSSGNSRSMQETDKN     1771
XP_004949340.1      GLDDDKTEEGDILAECINSAMPKGKSHKPFRVKKIMDQIQQASTS---LNNKNQPEGEKK     1773
XP_007497871.1      AFDDNKTEEGEILAECINSAMPKGKSHKPFRVKKIMDQIQQASAS-SSGNSKNPLDSEKK     1770
NP_031488.2         DLDDNKAEEGDILAECINSAMPKGKSHKPFRVKKIMDQVQQASST-SSGANKNQVDTKKK     1768
NP_001069454.2      ELDDSKTEEGDILAECINSAMPKGKSHKPFRVKKIMDQVQQASMS-SSGTNKNQLDGKTK     1776
XP_014996065.1      ELDDNKAEEGDILAECINSAMPKGKSHKPFRVKKIMDQVQQASAS-SSATNKNQLDGKKK     1770
AAA03586.1          ELDDNKAEEGDILAECINSAMPKGKSHKPFRVKKIMDQVQQASAS-SSAPNKNQLDGKKK     1770
                         : * .:******:*****.* ***:**:*: ** :: * :          : ..:

NP_001137312.1      KPTSPVKPMPQSSEYRARMLKRPEANNSLADPATYPDKNKETRKQEPKVVIRDFADKPSN     1794
NP_001084351.1      KPTSPVKPMPQSIGFKERLKKNTELKLNPNSENQYCDP-R---KPSSKKPSKVANEKIPN     1827
XP_004949340.1      KPTSPVKPVPQNSEYRARVRKNTESKSQINNERSYPEN-RDAKKQNLKNNSRDFNDKLPN     1832
XP_007497871.1      KPTSPVKPMPQSAEYRTRIRKNAESK-NVNVERSYS-D-KDSKKLSLKNNARDFLDKMPN     1827
NP_031488.2         KPTSPVKPMPQNTEYRTRVRKNTDSKVNVNTEETFSDN-KDSKKPSLQTNAKAFNEKLPN     1827
NP_001069454.2      KPTSPVKPIPQNTEYRTRVRKNTDSKNNLNAERNFSEN-KDSKKQHLKNNSKDFNDKLPN     1835
XP_014996065.1      KPTSPVKPIPQNTEYRTRIRKNADSKNNLNAERVFSDN-KDSKKQNLKNNSKDFNDKLPN     1829
AAA03586.1          KPTSPVKPIPQNTEYRTRVRKNADSKNNLNAERVFSDN-KDSKKQNLKNNSKDFNDKLPN     1829
                    ********:**.  :: *: *. : : .      :    :   *   :   :   :*  *

NP_001137312.1      AEERTRPGFAFDSPHHYTPIEGTPYCFSRNDSLSSLDFEDEDLDFSKEKAVLRKDKEQRK     1854
NP_001084351.1      NEERTKG-FAFDSPHHYTPIEGTPYCFSRNDSLSSLDFEDDDIDLSKEKAELRKEKGTKD     1886
XP_004949340.1      NEERVRGSFTFDSPHHYTPIEGTPYCFSRNDSLSSLDFDDDDVDLSREKAELRKGKEAKE     1892
XP_007497871.1      NEDRVRGSFTFDSPHHYTPIEGTPYCFSRNDSLSSLDFDDDDVDLSREKAEL-KGKEAKE     1886
NP_031488.2         NEDRVRGSFALDSPHHYTPIEGTPYCFSRNDSLSSLDFDDDDVDLSREKAELRKGKESKD     1887
NP_001069454.2      NEDRVRGSFTFDSPHHYTPIEGTPYCFSRNDSLSSLDFDDDDVDLSREKAELRKGKENKE     1895
XP_014996065.1      NEDRVRGSFAFDSPHHYTPIEGTPYCFSRNDSLSSLDFDDDDVDLSREKAELRKAKENKE     1889
AAA03586.1          NEDRVRGSFAFDSPHHYTPIEGTPYCFSRNDSLSSLDFDDDDVDLSREKAELRKAKENKE     1889
                     *:*.:  *::***************************:*:*:*:*:*** * * *  :.

NP_001137312.1      VPLLKCS-VEQPANTNMVSTFQTAPTKPL----------QKTVFPQAPKEN---TVVVCD     1900
NP_001084351.1      TDQKVKYKHENRAINPMGKQDQTGPKSLGGRDQPKALVQKPTSFSSAAKGTQDRGGATDE     1946
XP_004949340.1      VETKDCPNVEQPSGQQPSNRTQVCQKHPTSRSQSK-------TFCQPSKDIPDRGAATDE     1945
XP_007497871.1      TEAKVSNHLELTSNQQSANRAQICAKHPVERGQSKPLLQKQSTFPQSSKDMPDRVAATDE     1946
NP_031488.2         SEAKVTCRPEPNSSQQAASKSQASIKHPANRAQSKPVLQKQPTFPQSSKDGPDRGAATDE     1947
NP_001069454.2      SEAKVTNHTELTSNQQSASKTPAVTKQPINRGQSKPVLQKQSTFPQSSKDIPDRGAATDE     1955
XP_014996065.1      SEAKVTSHTELTSNQQSASKTQAIAKHPINRGQLKPILQKQSTFPQSSKDIPDRGAATDE     1949
AAA03586.1          SEAKVTSHTELTSNQQSANKTQAIAKQPINRGQPKPILQKQSTFPQSSKDIPDRGAATDE     1949
                             *  :     .      .                 * .  *       .. :

NP_001137312.1      EKQKFSIEDTPVCFSRNSSLSSLSDIDQENNNKDCSHK-----DDVTQMEAPRPQASGYA     1955
NP_001084351.1      KMENFAIENTPVCFSRNSSLSSLSDIDQENN-NKETEPLKQTGTSETQLGLRRPQTSGYA     2005
XP_004949340.1      KMQNFAIENTPVCFSRNSSLSSLSDIDQENNNNKEGEPVKRTEAPDSQIESSRPQTSGYA     2005
XP_007497871.1      KLQNFAIENTPVCFSRNSSLSSLSDIDQENNNNKESEPTKETEPPDSQGEPNRPQTSGYA     2006
NP_031488.2         KLQNFAIENTPVCFSRNSSLSSLSDIDQENNNNKESEPIKEAEPANSQGEPSKPQASGYA     2007
NP_001069454.2      KLQNFAIENTPVCFSRNSSLSSLSDIDQENNNNKENEPVKETEPPASQGEPGKPQASGYA     2015
XP_014996065.1      KLHNFAIENTPVCFSHNSSLSSLSDIDQENNNNKENEPIKETEPPDSQGEPSKPQASGYA     2009
AAA03586.1          KLQNFAIENTPVCFSHNSSLSSLSDIDQENN-NKENEPIKETEPPDSQGEPSKPQASGYA     2008
                    : .:*:**:******:*************** :.  .         :*    :**:****

NP_001137312.1      PKAFHVEDTPVCFSRNSSLSSLSIDSEDDLLQECISSAMPKKKKQTPRSKTEESGVKEEK     2015
NP_001084351.1      PKSFHVEDTPVCFSRNSSLSSLSIDSEDDLLQECISSAMPKKRKPSKIKNE----VGKSR     2061
```

```
XP_004949340.1      PKSFHVEDTPVCFSRNSSLSSLSIDSEDDLLQECISSAMPKKKKPSRIKSE----SEKSN      2061
XP_007497871.1      PKSFHVEDTPVCFSRNSSLSSLSIDSEDDLLQECISSAMPKKKRPSRFKGD----DEKPS      2062
NP_031488.2         PKSFHVEDTPVCFSRNSSLSSLSIDSEDDLLQECISSAMPKKKRPSRLKSE----SEKQS      2063
NP_001069454.2      PKSFHVEDTPVCFSRNSSLSSLSIDSEDDLLQECISSAMPKKKKPSRLKPD----NEKHS      2071
XP_014996065.1      PKSFHVEDTPVCFSRNSSLSSLSIDSEDDLLQECISSAMPKKKKPSRLKGD----NEKHS      2065
AAA03586.1          PKSFHVEDTPVCFSRNSSLSSLSIDSEDDLLQECISSAMPKKKKPSRLKGD----NEKHS      2064
                    **:***************************************:: :  .        :

NP_001137312.1      SMMADGILSEEPDLILDLTDTHSPISEQALSPDSESFDWKAIQEGANSIVSSLHQAA---      2072
NP_001084351.1      SNSVGGILAEEPDLTLDLRDIQSPDSENAFSPDSENFDWKAIQEGANSIVSRLHQAA-AA      2120
XP_004949340.1      SRNIGGMLAE--DLTLDLREIQRPDSEHGFSPDSENFDWKAIQEGANSIVSSLHQAA-AA      2118
XP_007497871.1      PRNMDGILAE--DLTLDLRDIQRPDSEHGFSPDSENFDWKAIQEGANSIVSSLHQAA-AA      2119
NP_031488.2         PRKVGGILAE--DLTLDLKDLQRPDSEHAFSPDSENFDWKAIQEGANSIVSSLHQAAAAA      2121
NP_001069454.2      PRNMGGILAE--DLTLDLKDIQRPDSEHGLSPDSENFDWKAIQEGANSIVSSLHQAA-AA      2128
XP_014996065.1      PRNMGGMLAE--DLTLDLKDIQRPDSEHGLSPDSENFDWKAIQEGANSIVSSLHQAA-AA      2122
AAA03586.1          PRNMGGILGE--DLTLDLKDIQRPDSEHGLSPDSENFDWKAIQEGANSIVSSLHQAA-AA      2121
                        .*:*.*  ** *** : : * **:.:*****.*************** *****

NP_001137312.1      ASLSRQGSSDSDSILSLKSGISIGSPFHLPLNQDDKPA-PNKGPRILKPGEKSSIEAKKK      2131
NP_001084351.1      GSLSRQGSSDSDSILSLKSGISLGSPFHLTLDKEEKTITSNKGPKILKPAEKSALENKKT      2180
XP_004949340.1      ASLSRQASSDSDSILSLKSGISLGSPFHLTPDQEEKPFTSNKGPRILKPGEKSTLESKKV      2178
XP_007497871.1      ACLSRQASSDSDSILSLKSGISLGSPFHLTPDQEEKPFTSNKGPRILKPGEKSTLETKKI      2179
NP_031488.2         ACLSRQASSDSDSILSLKSGISLGSPFHLTPDQEEKPFTSNKGPRILKPGEKSTLEAKKI      2181
NP_001069454.2      ACLSRQASSDSDSILSLKSGISLGSPFHLTPDQEEKPFTSNKGPRILKPGEKSTLETKKI      2188
XP_014996065.1      ACLSRQASSDSDSILSLKSGISLGSPFHLTPDQEEKPFTSNKGPRILKPGEKSTLETKKI      2182
AAA03586.1          ACLSRQASSDSDSILSLKSGISLGSPFHLTPDQEEKPFTSNKGPRILKPGEKSTLETKKI      2181
                    ..****.***************:******  ::::*    ****:****.***::* **

NP_001137312.1      EEETAKSLKGGKKVYKSLITGKPRPSLES--MASQHRQAQAPVISRGRTMVHVPGVRSSS      2189
NP_001084351.1      EE-EPKGIKGGKKVYKSLITGKSRSSSDFSSHCKQSVQTNMPSISRGRTMIHIPGVRASS      2239
XP_004949340.1      ES-ESKGIKGGKRVYKSIITGKARSNSEVSSQIKQPQQTSVPSISRGRTMIHIPGVRNSS      2237
XP_007497871.1      ES-ENKGIKGGKKVYKSLITGKVRSNSEVSGQLKQPLPTNMPSISRGRTMIHIPGIRNSS      2238
NP_031488.2         ES-ENKGIKGGKKVYKSLITGKIRSNSEISSQMKQPLPTNMPSISRGRTMIHIPGLRNSS      2240
NP_001069454.2      ES-ENKGIKGGKKVYKSLITGKVRSNSEISSQMKQPLQTNMPSISRGRTMIHIPGVRNSS      2247
XP_014996065.1      ES-ESKGIKGGKKVYKSLITGKVRSNSEISGQMKQPLQANMPSISRGRTMIHIPGVRNSS      2241
AAA03586.1          ES-ESKGIKGGKKVYKSLITGKVRSNSEISGQMKQPLQANMPSISRGRTMIHIPGVRNSS      2240
                    *.   *.:****:****:**** * . :     .*   :. * *******:*:**:* **

NP_001137312.1      PSTSPVPKKPPPRG-QMSKPPSQAPGAGSSPRTMKVPPSSEPSPASG--PPSSQGGSSKA      2246
NP_001084351.1      PSTSPVSKKGPVFKNVPSKGSNENPSSSSSPKGTKPLKS-ELVY--GSRPSSTPGGSSKG      2296
XP_004949340.1      SSTSPVSKKGPPFKNTNSKSPSEGQSSASSPRGVKSSVKPEPAPVTRQLSGLNQGGSSKG      2297
XP_007497871.1      SSTSPVSKKGPSLKTPTSKSPSEGPTSSTSPRGAKPSVKSELSPVTRQTS--QPGGSSKG      2296
NP_031488.2         SSTSPVSKKGPPLKTPASKSPSEGPGATTSPRGTKPAGKSELSPITRQTS--QISGSNKG      2298
NP_001069454.2      SSTSPVSKKGPPLKTPASKSPSEGQPATTSPRGTKPSVKSELSPVTRQAS--QTAGSNKG      2305
XP_014996065.1      SSTSPVSKKGPPLKTPASKSPSEGQTATTSPRGAKPSVKSELSPVARQTS--QIGGSSKA      2299
AAA03586.1          SSTSPVSKKGPPLKTPASKSPSEGQTATTSPRGAKPSVKSELSPVARQTS--QIGGSSKA      2298
                     ***** ** *      **  .:   : :**:  *   . *             .**.*.

NP_001137312.1      SSRSGSRDSTPSRPVQQSLTRPMQSPGRASVSPGRNGLSPSNKLSQLPQLPRTASPSASS      2306
NP_001084351.1      NSRSGSRDSASSRPSPQPLSRPLQSPGRNSISPGKNGISPPNKFSQ---LPRTTSPSTAS      2353
XP_004949340.1      PSRSGSRDSTPSRPQQQPLSRPLQSPGRNSISPGRNGISPPNKLSQ---LPRTSSPSTAS      2354
XP_007497871.1      PSRSGSRDSTPSRPSQQPLSRPMQSPGRNSISPGRNGISPPNKLSQ---LPRTSSPSTSS      2353
NP_031488.2         SSRSGSRDSTPSRPTQQPLSRPMQSPGRNSISPGRNGISPPNKLSQ---LPRTSSPSTAS      2355
NP_001069454.2      PSRSGSRDSTPSRPAQQPLSRPMQSPGRNSISPGRNGISPPNKLSQ---LPRTSSPSTAS      2362
XP_014996065.1      PSRSGSRDSTPSRPAQQPLSRPIQSPGRNSISPGRNGISPPNKLSQ---LPRTSSPSTAS      2356
AAA03586.1          PSRSGSRDSTPSRPAQQPLSRPIQSPGRNSISPGRNGISPPNKLSQ---LPRTSSPSTAS      2355
                     ********: ***  * *:**:***** *:***:**:** **:**   ****:***::*

NP_001137312.1      TKSSGSGRMAYTSPGRQLVQPTPTKQSGLPRSTSGIPRSESASKILNQC----GPSKKAE      2362
NP_001084351.1      TKSSGSGRMSYTSPGRQLSQPNLSKQSGLPKTHSSIPRSESASKSLNQNVNT-GSNKKVE      2412
XP_004949340.1      TKSSSSGRMSYTSPGRQMSQQNLTKQTALTKNTSSIPRSESASKGLNQILGSGASNKKTD      2414
XP_007497871.1      TKSSGSGKISYTSPGRQMSQQNLTKQTGLSKNTSNIPRSESASKGLNQISNSNGTNKKVE      2413
NP_031488.2         TKSSGSGKMSYTSPGRQLSQQNLTKQASLSKNASSIPRSESASKGLNQMSNGNGSNKKVE      2415
NP_001069454.2      TKSSGSGKMSYTSPGRQMSQQNLTKQTGLSKNGSGIPRSESASKGLNQMSNSNGSNKKVE      2422
XP_014996065.1      TKSSGSGKMSYTSPGRQMSQQNLTKQTGLSKNASSIPRSESASKGLNQVNNGNGANKKVE      2416
AAA03586.1          TKSSGSGKMSYTSPGRQMSQQNLTKQTGLSKNASSIPRSESASKGLNQMNNGNGANKKVE      2415
                    ****.**:::*******: * . :**:.* :. *.********* ***     . .**.:

NP_001137312.1      LSRMSSTKSSGSESDRSEKPGLVRQSTFIKEAPSPTLKRKLEESASFESLSPSST-----      2417
NP_001084351.1      LSRMSSTKSSGSESDRSERPALVRQSTFIKEAPSPTLRRKLEESASFESLSSSSRADSPP      2472
XP_004949340.1      LSRMSSAKSSGSESDRSERPVLVRQSTFIKEAPSPTLRRKLEESASFESLSP-SRPDSPT      2473
XP_007497871.1      LSRMSSTKSSGSESDRSERPVLVRQSTFIKETPSPTLRRKLEESASLESLSSSSRPDSPT      2473
NP_031488.2         LSRMSSTKSSGSESDRSERPALVRQSTFIKEAPSPTLRRKLEESASFESLSPSSRPDSPT      2475
```

```
NP_001069454.2      LSRMSSTKSSGSESDRSERPVLVRQSTFIKEAPSPTLRRKLEESASFESLSPSSRPDSPT    2482
XP_014996065.1      LSRMSSTKSSGSESDRSERPVLVRQSTFIKEAPSPTLRRKLEESASFESLSPSSRPASPT    2476
AAA03586.1          LSRMSSTKSSGSESDRSERPVLVRQSTFIKEAPSPTLRRKLEESASFESLSPSSRPASPT    2475
                    ******:***********:* **********:*****:********:****   *

NP_001137312.1      -SQSQTPVSSPSLPDMSLSLP--YQGSGWTKAPQSQN-SAENGDGKSLKRHDISRSHSES    2473
NP_001084351.1      RSQTQTPALSPSLPDMALSTHS-IQAGGWRKMPPNLNPAAEH--GDSRRRHDISRSHSES    2529
XP_004949340.1      RSQLQTPVLSPSLPDMSLSTHSTAQTSGWRKLPPNLSPSVEY-DGRPAKRHDIARSHSES    2532
XP_007497871.1      KSQVQTPILSPSLPDMSLSTHSSIQTGSWRKLPPNLNPSIEFNDGRSTKRHDIARSHSES    2533
NP_031488.2         RSQAQTPVLSPSLPDMSLSTHPSVQAGGWRKLPPNLSPTIEYNDGRPTKRHDIARSHSES    2535
NP_001069454.2      RSQAHTPVLSPSLPDMSLSTHSSLQSGGWRKLPPNLSPTIEYNDGRPVKRHDIARSHSES    2542
XP_014996065.1      RSQAQTPVLSPSLPDMSLSTHSSVQAGGWRKLPPNLSPTIEYNDGRPAKRHDIARSHSES    2536
AAA03586.1          RSQAQTPVLSPSLPDMSLSTHSSVQAGGWRKLPPNLSPTIEYNDGRPAKRHDIARSHSES    2535
                     ** :**  *******:**     * ..* * * . . : *   *   :****:******

NP_001137312.1      PSRLPINRTGTWKREHSKHSSSLPRVGTWKRTGSSSSILSASSESSEKGRSEDERQPTN-    2532
NP_001084351.1      PSRLPITRSGTWKREHSKHSSSLPRVSTWRRTGSSSSILSASSESSEKAKSEDEKQQVCS    2589
XP_004949340.1      PSRLPINRSGTWKREHSKHSSSLPRVSTWRRTGSSSSILSASSESSEKAKSEDEKQHGSS    2592
XP_007497871.1      PSRLPVNRSGTWKREHSKHSSSLPRVSTWRRTGSSSSILSASSESSEKAKSEDEKHV-SS    2592
NP_031488.2         PSRLPINRAGTWKREHSKHSSSLPRVSTWRRTGSSSSILSASSESSEKAKSEDERHV-SS    2594
NP_001069454.2      PSRLPINRSGTWKREHSKHSSSLPRVSTWRRTGSSSSILSASSESSEKAKSEDEKQV-NS    2601
XP_014996065.1      PSRLPINRSGTWKREHSKHSSSLPRVSTWRRTGSSSSILSASSESSEKAKSEDEKHV-NS    2595
AAA03586.1          PSRLPINRSGTWKREHSKHSSSLPRVSTWRRTGSSSSILSASSESSEKAKSEDEKHV-NS    2594
                    *****:.*:****************.**:*****************.:****::

NP_001137312.1      -PPQKSGKEGGLERKGTWRKAKGSETSYAPM---------------SLDLQDQTGDAMSK    2576
NP_001084351.1      FPGPRS--ECSSSAKGTWRKIKESEILETPSNGSSSTIAESNCSLESKTLVYQMAPAVSK    2647
XP_004949340.1      LSGQKQSKESQAPAKGTWRKIKENEIPQIMNDPQ-HPSSSATSSSDSKTLIYQMAPAVSK    2651
XP_007497871.1      LLGTKQTKENQGPAKGTWRKIKESEMTPISNVSQ-VTSSGTTNGADSKTLIYQMAPAVSK    2651
NP_031488.2         MPAPRQMKENQVPTKGTWRKIKESDISPTGMASQ-SASSGAASGAESKPLIYQMAPPVSK    2653
NP_001069454.2      ISGSKQTKENQVSTKGTWRKIKESEISPTNSTSQ-TTSSGAANGAESKTLIYQMAPAVSK    2660
XP_014996065.1      ISGTKQSKENQVSAKGTWRKIKENEISPTNSTSQ-TVSSGATNGAESKTLIYQMAPAVSK    2654
AAA03586.1          ISGTKQSKENQVSAKGTWRKIKENEFSPTNSTSQ-TVSSGATNGAESKTLIYQMAPAVSK    2653
                        :.  *     ****** * .:                    *  *  *  .  :**

NP_001137312.1      SEDVWVRIEDCPINNPRSSKSPTASTPPVIDSLPIKGLACDRDSSESHSKLMSENA---A    2633
NP_001084351.1      TEDVWVRIEDCPINNPRSGRSPTGNSPPVIDNVLDQGQKEE-AAKDCHTRHNSGNGN---    2703
XP_004949340.1      TEDVWVRIEDCPINNPRSGRSPTGNTPPVIDSVSEKGVVNGKDSKEIQEKQNPGNGS-VP    2710
XP_007497871.1      TEDVWVRIEDCPINNPRSGRSPTGNTPPVIDNVAEKVSSGNKESKDNQGKPNGGNGSSAP    2711
NP_031488.2         TEDVWVRIEDCPINNPRSGRSPTGNTPPVIDSVSEKGSSSIKDSKDTHGKQSVGSG--SP    2711
NP_001069454.2      TEDVWVRIEDCPINNPRSGRSPTGNTPPVIDTVSEKGNPNPKDSKDNQGKQNVSNGS-AP    2719
XP_014996065.1      TEDVWVRIEDCPINNPRSGRSPTGNTPPVIDSVSEKGNPN-KDSKDNQAKQNVGNGS-VP    2712
AAA03586.1          TEDVWVRIEDCPINNPRSGRSPTGNTPPVIDSVSEKANPNIKDSKDNQAKQNVGNGS-VP    2712
                    :*****************.:***..:*****.:   :        :.: : :    ..

NP_001137312.1      MSHLGSETNLNLLRSSESLDKKVTDIKPAPS-NPNIGPELHEFPVSERTPFSSTNSSKHS    2692
NP_001084351.1      --VPLLENRQKSFIKVDGLDTKGTDPKSLINN----QQETNENTVAERTAFSSSSSSKHS    2757
XP_004949340.1      VRTIGLENRLNSFFQMDSPDKKGNETKPLQT-NPVPAPENNESTVSERTPFSSSSSSKHN    2769
XP_007497871.1      ARTVGLENRLNSFIQIDSPDKKAAETKSGQV-NLVPAPETSETSVAERTPFSSTSSSKHS    2770
NP_031488.2         VQTVGLETRLNSFVQVEAPEQKGTEAKPGQS-NPVSIAETAETCIAERTPFSSSSSSKHS    2770
NP_001069454.2      TRTMGLENRLNSFIQVDPPDQKGTETKPGHSNNPVPASETSESSIAERTPFSSSSSSKHS    2779
XP_014996065.1      MRTVGLENRLNSFIQVDAPDQKGTETKPGQ-NNPVPVSETNESSIVERTPFSSSSSSKHS    2771
AAA03586.1          MRTVGLENRLNSFIQVDAPDQKGTEIKPGQ-NNPVPVSETNESSIVERTPFSSSSSSKHS    2771
                          *.. : : . :  : *  : *           *  *  : *** ***:.****.

NP_001137312.1      SPSGAVAARVSPFNYTPSPRKSSADGSTPRPSQIPTPISSNAKKRDTKGDTT--------    2744
NP_001084351.1      SPSGTVAARVTPFNYNPSPRKSNGENSTSRPSQIPTPVTNSTKKRDSKTETTDSSGSQSP    2817
XP_004949340.1      SPIGAVAARVTPFNYNPSRRKSSVDNSSARPSQIPTPVNNSTKKRDSKSENTDSSGTQSP    2829
XP_007497871.1      SPSGTVAARVTPFNYNPSPRKSSADSSAARPSQIPTPVNNSTKKRDSKTENTESSGTQSP    2830
NP_031488.2         SPSGTVAARVTPFNYNPSPRKSSADSTSARPSQIPTPVSTNTKKRDSKTDSTESSGAQSP    2830
NP_001069454.2      SPSGTVAARVSPFNYNPSPRKSSTDGTSARPSQIPTPVSNNTKKRDSKPDSTEPSGTQSP    2839
XP_014996065.1      SPSGTVAARVTPFNYNPSPRKSSADSTSARPSQIPTPVNNNTKKRDSKTDSTESSGTQSP    2831
AAA03586.1          SPSGTVAARVTPFNYNPSPRKSSADSTSARPSQIPTPVNNNTKKRDSKTDSTESSGTQSP    2831
                    ** *:*****:****.** ***. :.:: ********:...:****:* :.*
NP_001137312.1      --ESGSYIVTSV      2754
NP_001084351.1      KRHSGSYLVTSV      2829
XP_004949340.1      KRHSGSYLVTSV      2841
XP_007497871.1      KRHSGSYLVTSV      2842
NP_031488.2         KRHSGSYLVTSV      2842
NP_001069454.2      KRHSGSYLVTSV      2851
XP_014996065.1      KRHSGSYLVTSV      2843
AAA03586.1          KRHSGSYLVTSV      2843
                      .****:****
```
