# Supplementary material for: Curated multiple sequence alignment for the Adenomatous Polyposis Coli (APC) gene and accuracy of in silico pathogenicity predictions
Source: PLoS One. 2020 Aug 4;15(8):e0233673. doi: 10.1371/journal.pone.0233673 (PMC7402488; doi:10.1371/journal.pone.0233673)
Supplement: Attachment — Submitted filename: Response to Reviewers.pdf [file pone.0233673.s005.pdf]

Karabachev et al, Curated multiple sequence alignment for the Adenomatous Polyposis Coli (APC) gene and accuracy of In Silico pathogenicity predictions

We thank the reviewers and editor for their helpful comments. We are very happy that Reviewer 2 took such a positive view of the paper. Reviewer 1 and the editor each make one specific suggestion, which we have addressed as below. Also, we now provide the "data not shown".

1. Reviewer 1 couldn't follow the conclusion that "Our current analyses of APC variants suggest that the likelihood that a missense APC variant is pathogenic is far lower than 1%." We have modified this statement slightly, and provided the rationale, which is already in the manuscript on pages 11 and 12, and in Table 3. We think we have made this more clear:

"Our current analyses of APC variants suggest that the likelihood that a missense APC variant is pathogenic is very low, perhaps lower than 1%. We base this conclusion on the observation that only 2 of 1924 missense variants in Clinvar (0.10%) are classified as pathogenic (Table 3) after decades of analysis by testing labs and researchers."

2. The specific comment from the editor is slightly puzzling, but we think that we have found the source of the comment and edited the manuscript to clarify: "The authors have showed that with PMSA they have reassigned 5/21 to be pathogenic, they should mention those 5 variants and discuss how this differs from the others and the location and consequences of the variants in addition to addressing the points raised by reviewer 1."

We believe that the reviewer is referring to the sentence "When applied to 21 variants reported in ClinVar as Benign, the five methods ranged in accuracy from 76.2-100%" in the Abstract. We have clarified that we consider the variants to be securely classified as Benign. These variants are described in the manuscript on pages 12, 13, and 14, and in Tables 4, 5A, and 5B. We have edited sentences in the Abstract and in the text (p.13, line 292-294) to clarify that we consider these to be securely classified as Benign, and the discrepancy is due to the methods, not due to misclassification, as follows:

Abstract: "When applied to 21 missense variants reported in ClinVar and securely classified as Benign, the five methods ranged in accuracy from 76.2-100%."

Manuscript text: "For the n=21 variants securely classified in ClinVar as B/LB based on non-computational data, the prediction algorithms showed good concordance with each other and with the ClinVar classifications (Table 5A). "

3. Data Not Shown

The "Data not shown" were the 8 sequence and 9 sequence alignments. These are now included as Supporting Figure 3 and 4.

4. We have edited the Competing Interests section using the language provided in the Review.
